# Screening for chlamydia and/or gonorrhea in primary health care: systematic reviews on effectiveness and patient preferences

**DOI:** 10.1186/s13643-021-01658-w

**Published:** 2021-04-19

**Authors:** Jennifer Pillay, Aireen Wingert, Tara MacGregor, Michelle Gates, Ben Vandermeer, Lisa Hartling

**Affiliations:** Alberta Research Centre for Health Evidence, Faculty of Medicine and Dentistry, University of Alberta, 11405 87 Avenue, Edmonton, Alberta T6G 1C9 Canada

**Keywords:** Systematic review, Chlamydia, Gonorrhea, Screening, Sexually transmitted infections, Guideline, Patient values and preferences

## Abstract

**Background:**

We conducted systematic reviews on the benefits and harms of screening compared with no screening or alternative screening approaches for *Chlamydia trachomatis* (CT) and *Neisseria gonorrhoeae* (NG) in non-pregnant sexually active individuals, and on the relative importance patients’ place on the relevant outcomes. Findings will inform recommendations by the Canadian Task Force on Preventive Health Care.

**Methods:**

We searched five databases (to January 24, 2020), trial registries, conference proceedings, and reference lists for English and French literature published since 1996. Screening, study selection, and risk of bias assessments were independently undertaken by two reviewers, with consensus for final decisions. Data extraction was conducted by one reviewer and checked by another for accuracy and completeness. Meta-analysis was conducted where appropriate. We used the GRADE approach to rate the certainty of the evidence. The Task Force and content experts provided input on determining thresholds for important effect sizes and on interpretation of findings.

**Results:**

Of 41 included studies, 17 and 11 reported on benefits and harms of screening, respectively, and 14 reported on patient preferences. Universal screening for CT in general populations 16 to 29 years of age, using population-based or opportunistic approaches achieving low screening rates, may make little-to-no difference for a female’s risk of pelvic inflammatory disease (PID) (2 RCTs, *n*=141,362; 0.3 more in 1000 [7.6 fewer to 11 more]) or ectopic pregnancy (1 RCT, *n*=15,459; 0.20 more per 1000 [2.2 fewer to 3.9 more]). It may also not make a difference for CT transmission (3 RCTs, *n*=41,709; 3 fewer per 1000 [11.5 fewer to 6.9 more]). However, benefits may be achieved for reducing PID if screening rates are increased (2 trials, *n*=30,652; 5.7 fewer per 1000 [10.8 fewer to 1.1 more]), and for reducing CT and NG transmission when intensely screening high-prevalence female populations (2 trials, *n*=6127; 34.3 fewer per 1000 [4 to 58 fewer]; NNS 29 [17 to 250]). Evidence on infertility in females from CT screening and on transmission of NG in males and both sexes from screening for CT and NG is very uncertain. No evidence was found for cervicitis, chronic pelvic pain, or infertility in males from CT screening, or on any clinical outcomes from NG screening. Undergoing screening, or having a diagnosis of CT, may cause a small-to-moderate number of people to experience some degree of harm, mainly due to feelings of stigmatization and anxiety about future infertility risk. The number of individuals affected in the entire screening-eligible population is likely smaller. Screening may make little-to-no difference for general anxiety, self-esteem, or relationship break-up. Evidence on transmission from studies comparing home versus clinic screening is very uncertain. Four studies on patient preferences found that although utility values for the different consequences of CT and NG infections are probably quite similar, when considering the duration of the health state experiences, infertility and chronic pelvic pain are probably valued much more than PID, ectopic pregnancy, and cervicitis. How patients weigh the potential benefits versus harms of screening is very uncertain (1 survey, 10 qualitative studies); risks to reproductive health and transmission appear to be more important than the (often transient) psychosocial harms.

**Discussion:**

Most of the evidence on screening for CT and/or NG offers low or very low certainty about the benefits and harms. Indirectness from use of comparison groups receiving some screening, incomplete outcome ascertainment, and use of outreach settings was a major contributor to uncertainty. Patient preferences indicate that the potential benefits from screening appear to outweigh the possible harms. Direct evidence about which screening strategies and intervals to use, which age to start and stop screening, and whether screening males in addition to females is necessary to prevent clinical outcomes is scarce, and further research in these areas would be informative. Apart from the evidence in this review, information on factors related to equity, acceptability, implementation, cost/resources, and feasibility will support recommendations made by the Task Force.

**Systematic review registration:**

International Prospective Register of Systematic Reviews (PROSPERO), registration number CRD42018100733.

**Supplementary Information:**

The online version contains supplementary material available at 10.1186/s13643-021-01658-w.

## Background

### Impact of the infections

*Chlamydia trachomatis* (CT) and *Neisseria* g*onorrhoeae* (NG) are the most commonly reported bacterial sexually transmitted infections (STIs) in Canada [1, 2]. In 2017, CT was reported for 0.6–1.3% of males and 1.1–2.5% of females 15–29 years old, and for <0.4% for those above 30 years old [3]. NG rates are about one-tenth of CT [1, 2]. These annual reported cases are thought to underestimate actual rates by at least 70% [4], likely because the infections are largely asymptomatic, often treated using syndromic management, and are incompletely reported [4]. Additionally, without widespread testing of extragenital sites, reported rates generally reflect genital infections whereas oropharyngeal and rectal CT and NG infections can be as high as 20% and often occur in the absence of a genital infection [5–7]. Lastly, lower reported rates in 15–19 year olds compared with 20–24 year olds, and in males, are attributed to lower testing rates rather than true differences in prevalence. Screening studies have reported much higher rates in 15–19 year olds and similar rates between sexes [8, 9], which aligns with knowledge about behavioral (e.g., condomless sex [10]) and biological (e.g., cervical ectopy in adolescent females [11]) risk factors. Several sexual behaviors such as inconsistent condom use, multiple sexual partners, and partner(s) having concurrent partners place one at higher risk. Although the total number of cases is relatively low compared with other provinces and populations, the highest population-based prevalence rates in Canada are observed in Nunavut and the Northwest Territories and for Indigenous peoples [4]. These rates may reflect the relatively high impacts on several social determinants of health such as socioeconomic status, geography, demographics (younger median age), and other aspects of social vulnerability including colonialism [12]. There is also concern about the lack of access to screening as well as to culturally safe care especially in rural and remote regions. Some individuals (e.g., MSM, transgender) are disproportionally impacted because of a delay or avoidance of seeking STI-related information, care, and services as a result of anticipated homophobia, transphobia, ignorance, and insensitivity [13]. Having other STIs is also a risk factor. Up to 20 to 40% of individuals infected with NG are co-infected with CT, although fewer people (0.6–10%) with CT also have NG [4, 14–18].

Rates of CT and NG increased three-fold and five-fold, respectively, between 1997 and 2017 in Canada, with steady increases in CT and more accelerated increases in NG over the last 5 years [3]. These rises to some degree reflect increases in case finding, from the use of highly sensitive (86–98% [19]) nucleic acid amplification tests [1, 2], the availability of urine and self-swab sample collection, and increased screening of extragenital sites. There is also a hypothesis that the increased rates of CT are paradoxically due to increased reinfection rates following aggressive control efforts “seek and treat”, due to an “arrested immune state” associated with early initiation of treatment resulting in interruption of naturally acquired immunity [20].

The mean duration of CT is 1.4 years [21] and of NG is about 6 months [22]. The infections will resolve spontaneously if not treated, but while active, they can initiate inflammatory and immunological processes leading to several complications [23]. In females, CT and NG are important causes of pelvic inflammatory disease (PID), with CT implicated in one-fifth to one-third of all PID cases [24–26]. PID can be asymptomatic, resolve spontaneously, or lead to the sequelae of chronic pelvic pain, ectopic pregnancy, and infertility. It may also be possible for the infections to cause ectopic pregnancy and infertility without first causing PID [27]. Best estimates of the rates of complications in untreated CT, from longitudinal cohorts and control arms of representative trials, are 10–16% for PID [28, 29], 3–8% for chronic pelvic pain [27, 30], 0.02–2% for ectopic pregnancy, and 0.1–4.6% for infertility [27]. Infection with NG results in more severe manifestations and increases the risks of PID and its sequelae [31]. The duration and severity of these outcomes will vary [32]. In males, reproductive system complications include epididymitis, with or without orchitis, and, rarely [33], infertility. Other complications occur in both reproductive (e.g., urethritis, cervicitis) and non-reproductive sites (e.g., reactive arthritis, pharyngitis, proctitis) for both sexes. An uncommon complication of NG is disseminated gonococcal infection, thought to occur in <1% of those infected and with the rare sequelae of endocarditis [34]. CT and NG may increase susceptibility to the human immunodeficiency virus (HIV), although findings from longitudinal studies examining the associations between STIs and HIV acquisition have inconsistent findings, due to poor accounting for actual HIV contact/exposure and adjustment for confounders; moreover, trials have failed to demonstrate that STI control interventions can reduce HIV incidence [4, 5, 35–38]. Reinfection with CT or NG increases the risk for complications [27, 39–41]. A meta-analysis of 38 studies found median reinfection rates for CT of 13.9% (follow-up 2–60 months) and for NG of 11.7% (follow-up 3–20 months) [39]. Little is known about the reproductive consequences from single-site extragenital CT infections, although oropharyngeal infection can be transmitted to the genitals [42], and infection of the genitals may occur through contiguous spread from extragenital sites [43]. Current treatment regimens for uncomplicated urogenital CT and NG are over 95% effective [44–48], if adhered to, although antimicrobial resistance is becoming a major issue for NG [49].

### Screening for CT and NG

Because of the largely asymptomatic nature of the infections, screening may be necessary to reduce the clinical consequences discussed above related to the natural course of infection. Screening refers to systematically offering a test to detect an infection in those asymptomatic or not purposively seeking care for symptoms. It includes the associated follow-up including treatment and partner notification, as well as possibly re-testing for re-infection and counseling on future STI prevention. At a population level, the aim of screening is also to reduce transmission of the infections. However, screening might lead to negative physical (e.g., serious adverse drug effects from treatment) or psychosocial (e.g., stigma, anxiety) consequences. Possible benefits from reducing CT-related consequences relative to harms from the procedure need to be considered during decision making about implementing and participating in screening.

Different screening approaches are available with several considerations required related to their advantages and disadvantages. The relative priority between aims to prevent complications in individuals and to reduce transmission in the population may influence to whom, how often, and where screening if offered. Frequent and targeted screening of a specific proportion of the population may enable overall reduction of transmission in the larger population [50].Screening to reduce clinical complications in individuals may focus on opportunistic screening at visits to clinician offices or other health care sites including school-based health centers, STI clinics, pharmacies [51], or emergency departments [52]. Other detection strategies may focus on hard-to-reach individuals using outreach to non-health community settings such as gathering sites at colleges, bars, sex venues, or mobile vans [53–55]. Considerations for targeting individuals at increased risk of infection, based on sexual behaviors or group membership, include underreporting, possible stigmatization, practical considerations (e.g., addition of pre-screen to identify those at risk), and awareness that many cases may be missed. Conversely, screening the general/entire population that will on average have a lower prevalence of infection will increase rates of false positives and may lead to some unintended harm. The availability of non-invasive diagnostic tests (urine, self-swabs) may reduce the likelihood of people experiencing discomfort or embarrassment during the procedure and make screening easier to implement. However, the lower sensitivity for urine tests in females needs consideration [56]. Although at much lower prevalence than CT, consideration of whether to also screen for NG arises because of the availability of laboratory tests that can evaluate both organisms from a single sample and test and because current combined first-line treatment for NG (regardless of CT presence) can in most uncomplicated cases also treat CT [57].

Since 2010, national guidance from the Public Health Agency of Canada has recommended screening for CT in at-risk groups of any age and in all sexually active females and males under 25 years of age and pregnant women [58]. The 24-year age limit aligns with the statistics used from 2004, in which the highest reported cases of CT were among those aged 15–24 years. The inclusion of males is due to their being a source for infections and reinfections of their female partners, for which the consequences were considered more clinically significant. Screening annually is recommended for those under 25 years old and for gay, bisexual, and other MSM and transgender populations; screening and repeat screening, of unclear frequency, is recommended for people ≥25 with risk factors. Guidance also extends to case finding and partner notification as critical for controlling NG, but a specific definition (e.g., active seeking of signs and symptoms in at-risk individuals) or methods for case finding are not described. The current Canadian guidance was not based on a systematic review. Further, rates of CT have increased over time for those aged 25–29, and there are reports from screening trials completed after 2010 that would not have been considered by the guideline panel [8, 9, 28, 59, 60].

Preferences for or against a screening strategy are influenced by the relative importance people place on the expected or experienced outcomes incurred [61–63]. Evidence on how people weigh the relevant outcomes is important to inform guideline panels when considering the balance of benefits and harms and determining whether this balance might vary across different individuals [64].

### Purpose of review

To examine evidence on the effectiveness (impact of screening on critical/important benefits and harms) and comparative effectiveness of screening for CT and NG infections and on the relative importance people place on the relevant outcomes (patient preferences) from screening, to inform the Canadian Task Force on Preventive Health Care when making recommendations on screening to support primary health care providers in delivering preventive care. Existing reviews on screening effectiveness (e.g., [19, 65]) were considered out-of-date with knowledge of at least one new trial [8] and had different eligibility criteria than the Task Force’s. We are not aware of any existing reviews covering the full scope of the question on patient preferences. Several factors provided rationale for this review and its associated recommendations, as described in the additional files of the protocol [66].

## Methods

The review was undertaken following a peer-reviewed protocol [66] and is reported following current standards for systematic reviews [67]. The methods are outlined briefly here, focusing on the eligibility criteria and any deviations or new methods developed after the protocol. Methods for the review on the relative importance placed on the outcomes from screening (values and preferences) align with those used by members of Grading of Recommendations Assessment, Development and Evaluation (GRADE) [62].

A working group of the Task Force, with input from four topic experts, developed the key questions (KQs) and inclusion/exclusion criteria for the review (Additional file 1). The Task Force and topic experts rated the outcomes according to methods of GRADE [64]. Outcomes with final ratings as critical (7–9 on 9-point scale) by Task Force consensus were the following potential benefits (with reductions in): transmission of CT and NG via reduced incidence or prevalence of the infections over time, cervicitis, PID, chronic pelvic pain in females, ectopic pregnancy, and infertility in females and males. Two harm-related outcomes were rated as important (4–6 on scale): serious adverse drug reactions and negative psychosocial impact of screening or diagnosis. The ratings of outcomes were not changed after findings from an outcome rating exercise and focus groups with a sample of sexually active individuals in Canada conducted by an independent group with expertise in knowledge translation from St. Michael’s Hospital in Toronto, Ontario. Stakeholder organizations reviewed the KQs and inclusion/exclusion criteria (*n*=14) and a draft version of this report (*n*=15). All comments were taken into consideration, and no substantive changes were made to the conclusions.

### Key questions

The key questions (KQs) of interest were as follows:

- KQ1: What is the effectiveness of screening compared with no screening for chlamydia and/or gonorrhea in non-pregnant sexually active individuals?
- KQ2: What is the comparative effectiveness of different screening approaches for chlamydia and/or gonorrhea in non-pregnant sexually active individuals?
- KQ3: What is the relative importance that people place on the potential outcomes from screening for chlamydia and/or gonorrhea?

### Eligibility criteria

#### Key questions 1 and 2

The population of interest for KQs 1 and 2 was non-pregnant sexually active individuals of any age, who were not seeking care for symptoms. We excluded studies focusing on pregnant persons, but not those that may have included individuals who were pregnant. Studies that included more than 25% of individuals seeking care for symptoms at baseline were excluded. We also excluded studies enrolling individuals already known to have recent CT and/or NG infections, except when capturing the outcomes of interest related to psychosocial harms of a diagnosis from undergoing screening.

Interventions of interest included any screening approach that included testing and management for individuals who tested positive. We excluded studies using point-of-care tests because these tests are not approved for use in Canada. We included studies on screening for CT and/or NG along with any other STI(s) because the outcomes of interest are attributed to CT and NG. The comparisons of interest were no screening (KQ1) or a screening approach differing from the intervention (KQ2) by the main variables of interest (Additional file 1 Table 1).

The outcomes of interest were those rated as critical or important for decision making by the Task Force, as described above. Infection transmission and infertility required at least 3 and 12 month’s follow-up, respectively. Chronic pelvic pain was defined as being of at least 6 month’s duration. Treatment rates in the study populations were considered as a proxy for transmission.

Randomized (RCTs) and non-randomized controlled clinical trials (CCTs), as well as retrospective and prospective controlled cohort studies, were included for all outcomes. As defined in the protocol, the decision to accept uncontrolled studies for the outcome of negative psychosocial impact was based on the lack of evidence from controlled studies. We did not have a minimum threshold for a study’s risk of bias but considered the risk of bias when interpreting the findings. We included studies published in English or French, on or after 1996 aligning with the introduction of most relevant NAAT tests, and conducted in high or very high Human Development Index countries [68] to achieve a similar epidemiologic and healthcare context as Canada.

#### Key question 3

For assessing the relative importance of the outcomes, all participants could have had symptoms or a recent diagnosis of CT and/or NG. Participants may not have experienced screening or testing for CT or NG but could have experienced or been presented with information about the relevant clinical outcomes. Post hoc, we included studies where the participants (e.g., caregivers, clinical experts) were serving as a proxy for the eligible population. The exposures of interest were (i) experience with any screening program for CT and/or NG, (ii) experience with an infection or one of the critical outcome(s) of interest, or (iii) exposure to scenarios about the possible outcomes of screening. A comparator of no screening was not relevant because the focus was on the relative importance of the different possible outcomes. Unlike when assessing harms from screening or a diagnosis in KQ1, in KQ3 studies with data on harms, participants did not have to have experience with screening, there did not have to be data for comparison from before the intervention/diagnosis or with people without these experiences, and there needed to be a comparison with benefits. We also used qualitative findings for this question but not for KQ1. Outcomes/data of interest included (i) health-state utility values or other utility values, (ii) non-utility, quantitative information on relative importance of benefits versus harms, and (iii) qualitative information indicating the relative importance between benefits and harms. Any experimental, descriptive, or qualitative study design met inclusion criteria, including surveys, qualitative studies, stated and revealed preference studies, and studies measuring health-state utility weights (Additional file 1 Table 2). Criteria related to language, publication date, and country were the same as for KQs 1 and 2.

### Searching the literature and selecting studies

Our research librarian conducted comprehensive, peer-reviewed, searches in relevant bibliographic databases on June 5, 2018, with an update on January 24, 2020: Ovid Medline, Ovid Embase, Wiley Cochrane Library, CINAHL via EBSCOhost, and Ovid PsycINFO (searches in Additional File 7 in protocol [66]). The search was comprehensive for all KQs, with the exception of studies for KQ3 measuring health-state utility values for which we updated the search of an existing systematic review from 2013 to January 26, 2020 [32]. Additional sources of literature for all KQs were ClinicalTrials.gov (inception–2018), meeting abstracts via the Conference Proceedings Citation Index–Science edition (Clarivate Analytics; 1996–2018), and reference lists of included studies and relevant systematic reviews. We also searched for reports of research using websites of several organizations: Centers for Disease Control and Prevention, BC Centre for Disease Control, College of Registered Nurses of British Columbia, International Union Against Sexually Transmitted Infections, Pan American Health Organization, Public Health Agency of Canada, and the World Health Organization. Independent review by two reviewers with consensus or third reviewer involvement was used for screening and final selection of studies.

### Data extraction and analysis

One reviewer extracted data and another verified all data for accuracy and completeness. Study and population characteristics were extracted based on a priori variables (Additional file 1) and were tabulated. As described in Additional file 1, the definitions for some outcomes were refined after study selection but before analysis. In particular, for psychosocial harms, we received clinical input to determine which of many reported outcomes aligned with the outcome categories of interest. Further, for KQ3 when using utility values because of large variation in the duration of the different health states (e.g., PID typically has a much shorter duration than chronic pelvic pain), we multiplied the utility values by an estimated duration of effect using the range of durations applied in various cost-utility analyses [32]. This generated an estimated range of the quality-adjusted life year losses (QALY loss) for each state. Using these QALY loss estimates, we then determined a rank order of importance of the relevant outcomes and reported this in addition to the main outcome of the utility value for each health state. Assumptions relevant to this approach are described in Additional file 2.

We intended to assess risk status by participant reports of sexual behaviors and/or other factors increasing risk, but due to lack of reporting or use of risk factors for inclusion, we needed to rely on CT or NG baseline prevalence in the studies to categorize studies as enrolling populations, versus individuals, at general or high risk. Based on the baseline prevalence in the trials of general populations (4–6%), consideration that Canadian statistics (of about 1–2.5% CT) represent underreporting possibly by 70%, and after input from the Task Force and content experts, greater than 7% CT prevalence at baseline was used as the threshold for an increased risk study population.

When meta-analysis was possible and appropriate, due to similarity in populations, outcomes, and interventions, we used the DerSimonian Laird random effects model using Review Manager Version 5.3 (The Cochrane Collaboration, Copenhagen, Denmark). When results were not combined using meta-analysis due lack of common measurement (e.g., harms data), we used narrative descriptions of each study for our analysis and interpretation. We then compared and contrasted study findings by study methodology, populations, outcome presentations provided to participants (for KQ3), and analysis. With qualitative studies in KQ3 on the relative importance between harms and benefits when making decisions about screening, there was often numerical data from content analysis to use for the analysis, and in other cases, we used data on the frequency of comments/quotes related to our critical outcomes and interpreted the strength of the preference based on the language in quotes and narratives from the authors.

For studies using cluster design but not appropriately accounting for this in their analysis, we adjusted the findings using an interclass correlation coefficient of 0.028 [69]. For dichotomous outcomes, we report relative risks (RR) or odds ratios (OR) between groups with corresponding 95% confidence intervals (95% CI). When ORs were used for the analysis, we calculated RR using the control event rate. We also calculated the absolute risk reduction and risk differences, based on GRADE guidance [70]. In addition to using the study control event rates (medians when multiple studies were in the analysis) for calculating absolute effects, we also made calculations to estimate—relying on natural history parameters (see Additional file 2)—assumed/illustrative effects for both general and high-risk (i.e., prevalence) populations for the PID outcome.

We had several population and intervention variables of interest for performing potential subgroup/stratified analysis for the outcomes where meta-analysis was performed and indicated heterogeneity (Additional file 1), but because of including few studies in all meta-analyses, no subgroup analyses were conducted. Several sensitivity analyses were conducted, based on risk of bias, study design, or our need to make assumptions during data analysis. If there had been at least eight studies of varying size in a meta-analysis, we would have analyzed for publication bias both visually using the funnel plot and quantitatively using Egger’s test [71].

### Risk of bias assessments

We used several methods and tools for assessing risk of bias, for RCTs and CCTs [72, 73], cohorts [74], surveys/cross sectional studies [75], and qualitative studies [76]. We relied on recent guidance from GRADE for assessing risk of bias of studies in KQ3 measuring utilities and adapted the Newcastle-Ottawa tool for cohort studies [74], as described in Additional file 2. Risk of bias ratings for all studies contributing to each analysis were used during our assessments of the certainty of evidence.

### Assessing the certainty of the evidence on outcomes across the studies

For KQs 1 and 2 on effectiveness, we did separate GRADE assessments for trials (starting at high certainty) and observational (starting at low certainty) study designs and relied on guidance from GRADE [64, 77–79]. For the KQ3 on patient preferences, we relied on GRADE guidance published after the protocol publication [61, 63]. We did not rely on RCTs for obtaining high certainty evidence for this KQ because causation from the intervention is not relevant to valuation of outcomes.

Our GRADE assessments were based on absolute rather than relative effects and considered thresholds for minimally important effects that were developed (see Additional file 2) for several outcomes: PID, 2.5 fewer or more cases per 1000 (e.g., reflecting a 25–32% relative reduction in estimated 0.8–1% CT-related PID); ectopic pregnancy and infertility, 1 fewer or more per 1000; CT and NG transmission, 5 fewer or more per 1000 (10 fewer or more per 1000 was determined to be a moderate effect) when using prevalence data, and 20 more or fewer per 1000 when using treatment rates as a proxy for transmission. We did not base our assessments of precision on the null/statistical significance but rather the estimates of effects and the 95% CIs in relation to the thresholds. Assessments and findings are presented narratively and using tables including GRADE Evidence Profiles and Summary of Findings tables.

### Interpretations

We chose to use standard wording to describe the level of certainty of each finding. For findings of high, moderate, and low certainty evidence, we use “will,” “probably/likely,” and “may/appears to,” respectively, in our textual descriptions when discussing the results [80]. For very low certainty findings, we either use “may (make little-to-no difference/reduce/increase), but the evidence is very uncertain” or “the evidence is very uncertain,” reflecting a continuum of our certainty (from a small amount to none) within this category [80].

## Results

**Fig. 1:**
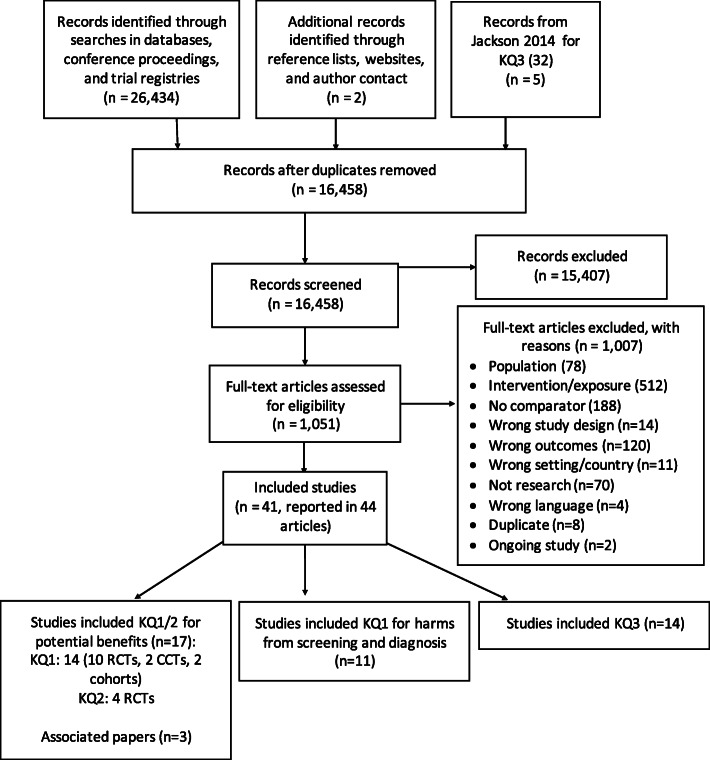
Literature flow diagram for all key questions. *One RCT [109] was included for KQs 1 and 2, and another RCT [8] was included for KQ1 benefits and harms

Our searches retrieved 16,458 unique citations, and after screening of abstracts (when available) or titles, 15,407 were excluded as irrelevant. After reviewing 1051 full texts, we included 41 studies [8, 9, 12, 28, 59, 60, 81–115] with three additional associated publications [116–118] (Fig. 1). The 1007 studies excluded based on full text review are listed, with reason, in Additional file 3. Many studies reporting on harms from a CT or NG diagnosis were excluded because the diagnosis was not attributed to a screening intervention and/or more than 25% of participants reported symptoms before the testing. Further details on the studies are included in the below sections based on KQ and outcome.

### Effectiveness of screening versus no screening (key question 1)

For KQ1, we included 14 studies that reported on potential benefits: 10 RCTs [8, 9, 12, 28, 59, 60, 98, 105, 108, 109], 2 CCTs [88, 89], and 2 retrospective cohorts [101, 111]. One RCT [8] and 10 uncontrolled cohort studies [81, 86, 92–95, 97, 100, 104, 114] were included for harms of the screening process or a diagnosis of CT from a screening program.

#### Benefits

##### Study characteristics

**Fig. 2:**
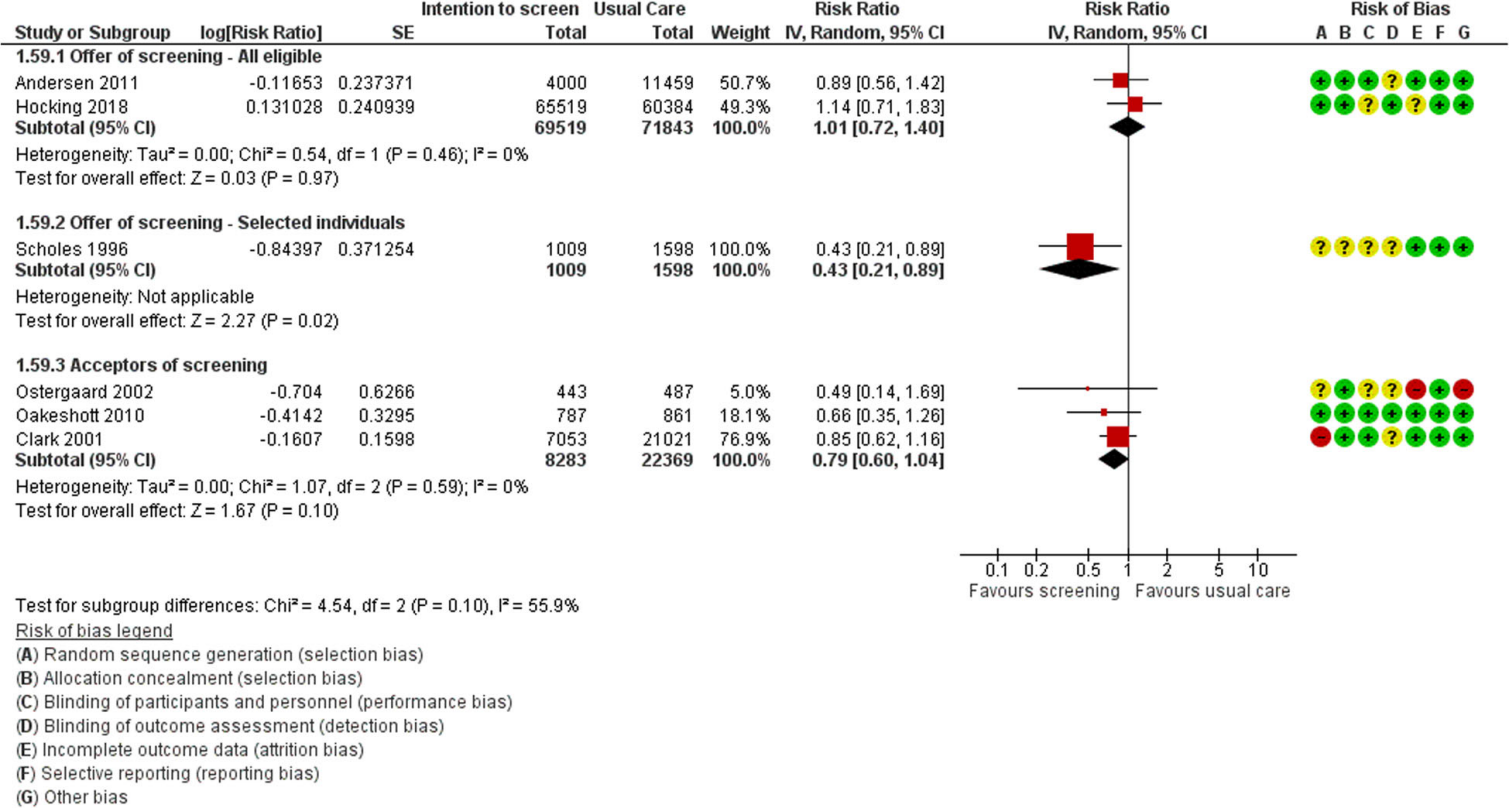
Meta-analyses and findings on relative effects* from trials reporting on pelvic inflammatory disease, grouped by screening approaches. *See Table 2 for findings using absolute rather than relative effects

Table 1 and Additional file 4 include the characteristics of studies addressing the benefits from screening versus no screening. Ratings of risk of bias by study are included in Table 1, Fig. 2, and Additional file 5. We did not locate any trials where the comparator was no screening; all trials had usual care controls which were described as including some form of ad hoc screening, regardless of further contamination bias from lack of participant blinding. We included these in a post hoc manner as indirectly relevant to KQ1. Although there was a small amount of overlap, studies naturally formed groups based on whether they focused on the more clinical (individual) outcomes or on transmission (population). None of the studies only screened for NG. All studies employed a universal approach with all enrolled participants offered or undertaking the screening; we did not locate any studies employing a risk-based strategy whereby outcomes in an entire general population were assessed based on only screening those deemed to be at higher risk.

**Table 1:** Study characteristics for studies reporting on benefit outcomes for screening versus no screening (key question 1)

Author, year Country	Design, intensity, sample size, risk of bias	Screening rates in intervention group (IG), rates of testing outside of study (IG; control group [CG])	Sex and age	Baseline CT positivity of tested, all sexually active (Y/N)	Recruitment	Screening approach; CT vs CT and NG; location, test, and person testing; re-testing; co-interventions	Outcome assessment Incidence of PID in CG Follow-up durations
**Randomized controlled trials**							
Andersen et al. 2011 [59] Denmark	RCT 1 screen offered 15,459 ROB: unclear (detection bias)	29% IG 9.0% vs CG 9.4% during 3 mos study period	F 21–24 yrs	7.1%; N	Population-based via mailed kits	Universal; CT; home-collected vaginal pipette, with NAAT; 70% re-tested; none	**PID**: hospital discharge ICD codes or doxycycline prescriptions (only used for PID in Denmark; 33% by GPs). Incidence in CG 0.65%. Follow-up duration, 1 yr **EP and infertility**: hospital discharge ICD codes. Follow-up duration, 9 yrs
Garcia et al. 2012 [60] Brazil	RCT cluster Screening offered every 8 wks over 3 yrs 20 cities with >50,000 inhabitants; follow-up survey using sampling at random sites at baseline (3732) and after 4 yrs (4156) ROB: unclear (performance bias)	NR (interviewed FSWs 48,207 times during 20 8-wk cycles) NR	F >14 yrs Mean 24.5 yrs	15.5% CT, 2.4% NG; Y	Outreach via mobile teams	Universal; CT and NG (and other STIs); mobile site self-collected vaginal swabs with NAAT; re-testing NR but frequent visits; multi-faceted syndromic management in general population and clients of FSWs; condom promotion with motivational interviewing and free condoms; peer education	**Estimated population prevalence** in FSWs using surveys (>99% of eligible enrolled) at random FSW sites. Follow-up duration, 3.5 yrs
Hocking et al. 2018 [8] Rural Australia	RCT cluster 3 annual screens offered 52 clusters (130 clinics of >500 16–29 yr olds) ROB: unclear (performance and attrition biases)	24% ≥1 times over 3 yrs (8.2% pre-trial yr to 20% at 25–36 mos) IG NR; CG rates increased from 8.2% pre-trial to 12.9% (stable over trial)	F and M 16–29 yrs	10% in those testing; 4.8% (4.5% females and 5.5% males) in prevalence surveys; Y	Primary care (clinic attenders)	Opportunistic; CT; in-clinic patient-collected vaginal or urine with NAAT; approx. 25% re-testing each year; multi-faceted with provider reminders, incentives, education, payments and feedback, and patient recall systems	**Clinic PID**: cumulative incidence in clinics for women 16–33 with at least one clinic visit during intervention period. Criteria provided to all providers but not blinded. Incidence in CG 0.4%. **Hospital PID**: ICD codes for all 15–34 yr olds living in each cluster. Incidence in CG 0.4%. Not used for main analysis because of low ascertainment and difference in trial and hospitalized populations **Estimated population prevalence (in clinic attenders)**: Using surveys of consecutive clinic attenders (70% response) before randomization and at end of trial (difference in change from baseline) Follow-up duration for all outcomes: 3 yrs
Hodgins et al. 2002 [12] Nunavik region in Northern Quebec	RCT cluster 1 screen offered 12 communities in Nunavut (2320, 15–39 years) ROB: high (incomplete outcome data with reported rates based on low uptake; multiple unclear domains)	31% NR	F and M All; focus on 15–39 yrs	7%; Y	Outreach via community	Universal CT; home urine sampling with PCR; re-testing NR; intensive community health education program	**Estimated population prevalence** via reported rates over past yr in communities. Follow-up duration, 1 yr
Klovstad et al. 2013 [98] Norway	RCT 1 screen offered 41,519 (10,000 IG) ROB: low	IG 14% (85% of 16.5% testing via study or healthcare system) IG 2.5%, CG 3.4%	F and M 18–25 yrs	IG 6.3% vs 11.6%; N	Population-based register via mailed invitations with screening kits (no reminders)	Universal; CT; home urine sampling via mailed kit via NAAT; N; N	**Treatment for CT:** national prescription database (filled at least one prescription for (azithromycin, doxycycline, erythromycin, lymecyklin, amoxicillin) within 30 days following a positive test result. Follow-up duration, 3 mos
Oakeshott et al. 2010 [28] London, UK	RCT 1 screen 2529 ROB: low	100% 22% both groups (43% of those CT+ in CG)	F 16–27 (mean 21) yrs	5.4%; Y	Outreach at common rooms, lecture theaters, and student bars at universities and further education colleges in London	Universal; CT; outreach site self-collected vaginal swabs with NAAT; re-testing NR; informed of risks of CT infection	**PID**: Any report by participants or their providers about signs and symptoms or dx, looked to medical records in general practitioners, hospitals, family planning clinics, and genitourinary medicine clinics. Used criteria for all cases, but medical records sometimes incomplete. Incidence in CG 1.8%. Follow-up duration, 1 yr
Ostergaard et al. 2000 [105] Denmark	RCT cluster 1 screen 17 schools (IG 928 vs CG 833) ROB: high (attrition [>50%] and lack of cluster analysis), unclear for other domains except allocation concealment	IG 93% vs CG 7.5% IG 29% and CG 36% (*p*=0.04)	F ≥15 yrs in high school (9% ≥19 yrs)	IG 5% vs CG 7.9%; Y	Outreach in schools with provision of home kits or invitation/reminder to go to general practitioner or STI clinics	Universal; CT; home sampling using vaginal pipette and NAAT vs. in-clinic swab with EIA; re-testing NR; information about consequences	**PID**: Self-reported at follow-up questionnaire, with confirmation in registration for prescriptions. Incidence in CG 4.1%. Follow-up duration, 1 yr
Scholes et al. 1996 [108] Washington, USA	RCT 1 screen offered to selected females 2607 ROB: unclear (selection, performance and detection biases)	64% NR	F 18–34 yrs; 81% ≤24 yrs	7%; Y	Primary care using telephone recruitment with questionnaire for high-risk considering race, douching, and ≥2 sexual partners in the preceding 12 months; married women excluded	Universal; CT; in-clinic; clinician-collected cervical swabs (EIA or culture); re-testing NR; none	**PID**: Self-report signs, symptoms, dx; medical records, hospital discharge, and pharmacy records. Dx had to be recorded and considered “clinical” (37 of 142 reported PID confirmed) but no specific criteria provided. Incidence in CG 2%. Follow-up duration, 1 yr
Senok et al. 2005 [109] UK	RCT postal and opportunistic vs usual care over 4-month period 476 ROB: high for attrition bias; unclear for selection and detection	Opportunistic 21%; postal 48%; UC 0%; NR	F 16–30 yrs; mean 24 yrs	Opportunistic 14% Postal 5% Usual care NR; N	Letters from general practice lists	Universal opportunistic and postal; CT; NR; no; incentives to providers at practices	**Treatment for CT:** clinic records. Follow-up duration, 4 mos
van den Broek et al. 2012 [9] Netherlands	RCT stepped-wedged cluster 3 annual offered 190 clusters with 317,304 people in three regions ROB: unclear for performance and detection biases and incomplete outcome data (prevalence); high for incomplete outcome data (positivity)	16% (1st round), 10% (3rd round) (vs 13% in controls) NR	F and M 16–29 yrs	4.3% (7.1% in <20 yr olds); NR	Population-based with postal invite to request sampling kit via internet	Universal; CT; home with urine for males and vaginal swab or urine for females; kits for re-testing sent 6 mos after CT+ (uptake NR); none	**Estimated population prevalence** using data from positivity with extrapolation to sexually active population of same ages in communities. Follow-up duration, 2 yrs
**Controlled clinical trials**							
Clark et al. 2001 [88] USA (Army recruits to South Carolina)	CCT 1 screen 28,074 ROB: high (selection and detection bias)	100% NR	F 17-39 yrs; 88% ≤25yrs	9.1%; N (93% of IG but higher and unknown for CG)	Community outreach via non-health Army training examination center	Universal; CT; on-site self-collected urine with NAAT; re-testing NR; education on STDs	**PID, EP, infertility**: Hospital discharge records. Incidence in CG 5.1/1000 PY (0.8%), 1.9/1000 PY and <0.01/1000 PY. Follow-up duration: mean 1.5 yrs
Cohen et al. 1999 [89] Louisiana, USA	CCT Bi-annual screening offered over 2.5 years (CG invited in 3rd year with 1 test period) 5907 from 3 IG and 5 CG schools ROB: high for selection, performance (11–53% testing outside of trial), attrition and other (no cluster adjustment) biases	83% at least once; annually, 52 to 65% IG and CG 11% grade 9 and 53% grade 12, males ~20%	F and M Grades 9–12	CT 11.5% females (8.7% in grade 9s vs 14% in grade 12s), 6.2% males NG 2.5% females, 1.2% males (similar across ages) N	Community health in high school health centers	Universal; CT and NG; on-site urine with NAAT; re-testing NR but bi-annual testing; information about risks and consequences	**CT positivity in screening eligible students**, who participated in screening, in IG after year 2 (5 tests offered) and CG (offered screening after year 2 in IG); Follow-up duration, 2.5 yrs **NG positivity in screening eligible students**, who participated in screening, in IG after year 1 (3 tests offered) and CG (offered screening after 1 year of NG testing added in 2nd year of study). Follow-up duration, 1.5 yrs
**Observational studies**							
Sufrin et al. 2012 [111] California, USA	Retrospective cohort 1 screen 57,728 ROB: moderate (selection bias with some adjustment)	100% NA	F 14–49 yrs; mean 32 yrs (non-screened 7 yrs older)	NR; Y	Primary care	Unknown but assume some form of risk assessment for screening same day or up to 1 year before IUD insertion; CT; in-clinic but unknown test and methods; re-testing NR; none	**PID after IUD insertion**: Health Maintenance Organization database ICD plus antibiotic pharmacy dispensed, with record review in 10% random sample if discordant; closed system. No specific criteria. Incidence in CG 0.36%. Follow-up duration, 3–15 mos (3 mos after IUD insertion but up to 15 mos from screen)
Low et al. 2006 [101] Sweden	Retrospective cohort 48% screened once, 22% twice, 30% ≥3 tests over 10 yr 43,715 ROB: moderate (selection bias with some adjustment)	100% NA	F 15–24 yrs	NR (11.5% at some time during follow-up); N	Population-based	Opportunistic used in county; CT; in-clinic sampling NR with culture; none	**PID, EP, infertility**: hospital discharge in-(all yrs) and out-(last 6 yrs) patient. No criteria. Incidence in CG 2.9%, 1.9%, 3.1% over 10 yrs. Follow-up duration, 10 yrs

*Abbreviations*: *CCT* controlled clinical trial, *CG* control group, *CT Chlamydia trachomatis*, *Dx* diagnosis, *F* females, *FSW* female sex workers, *ICD* International Classification of Diseases, *IG* intervention group, *IUD* intrauterine device, *M* males, *mos* months, *NAAT* nucleic acid amplification test, *NG Neisseria gonorrhoeae*, *NR* not reported, *PID* pelvic inflammatory disease, *RCT* randomized controlled trial, *ROB* risk of bias, *wks* weeks, *yrs* years

Five RCTs [8, 28, 59, 105, 108], one CCT [88], and two cohorts [101, 111] provided data for one or more of the clinical outcomes of PID, ectopic pregnancy, or infertility in females from CT screening. No study reported on cervicitis, chronic pelvic pain in females, or infertility in males. The three trials by Hocking et al. [8], Andersen et al. [59], and Scholes et al. [108] used an intention-to-screen design, whereby universal screening was offered and results were captured in the entire population regardless of screening attendance. Hocking and Andersen both enrolled general populations in healthcare clinics or via population-based registers, respectively, meeting their age criteria. Other facets of the intervention in Hocking included provider reminders, education, payments and feedback, and patient recall systems. Scholes enrolled females recruited by telephone with a questionnaire to enroll those with an increased risk for CT and who were willing to set up a primary care clinic appointment to have the clinician collect a cervical swab [108]. This trial was considered to enroll a select population interested in screening. Screening rates in Hocking and Andersen were fairly low (24–29%), whereas those in Scholes were high [64%]. The females in all three RCTs were considered to be at general risk (≤7% CT at baseline), despite the attempt in Scholes to obtain a high-risk sample. Hocking reported on PID diagnosed in clinics and hospitals at 3-year follow-up, Andersen reported on PID at 1-year follow-up and infertility and ectopic pregnancies after 9 years, and Scholes reported on PID at 1 year. Few data on risk factors were reported; in Hocking, 32% reported two or more sexual partners in the past 12 months, and in Andersen, the participant characteristics only included marital (e.g., 20% were married) and employment status.

The RCTs by Ostergaard et al. and Oakeshott et al. [28, 105], the CCT by Clark et al. [88], and the two cohort studies by Low et al. and Sufrin et al. [101, 111] were considered to use *acceptors of screening* design rather than an intention-to-screen approach because of only enrolling females submitting a sample or having very high uptake (93%) [105] of the offer of a test in the screening group. All three of the trials used outreach recruitment in non-healthcare settings within high schools [105], universities [28], or military training centers [88]. Ostergaard and Oakeshott both reported on PID at 1-year follow-up. Ostergaard did not account in their analysis for clustering, so we adjusted their findings. The CCT by Clark compared rates of PID, ectopic pregnancy, and infertility at mean follow-up duration of about 1.5 years. On average, the females in Ostergaard and Oakeshott were at general risk, while those in Clark had an increased risk (9% CT at baseline). The cohort study by Low examined rates of PID, ectopic pregnancy, and infertility in females over 10 years, linking data from a population register to hospital discharge codes in a Swedish county where opportunistic screening had been routinely undertaken [101]. Sufrin investigated rates of PID development 3 months after insertion of an intrauterine device in primary care, based on screening status in the 1 year prior to the insertion [111].

The trials reporting on transmission using estimated population prevalence (4 RCTs [8, 9, 12, 60]) or participant positivity at follow-up (1 CCT [89]) employed cluster designs with intention-to-screen approaches as appropriate for this outcome. The RCTs by Hocking et al. [8], van den Broek et al. [9], and Hodgins et al. [12] were similar in that they enrolled general-risk populations and offered low-intensity interventions with annual screening tests for 1 to 3 years. Screening rates were quite low (16–31%) across these trials. Two trials, by Garcia and Cohen, focused on high-risk populations (>11% CT at baseline) with more intensive CT and NG screening programs using outreach programs in communities for female sex workers (FSWs) in Garcia and high schools in Cohen. In the trial by Garcia, other interventions within the general populations of these clusters involved multifaceted syndromic management in the general population and clients of FSWs, condom promotion with motivational interviewing and free condoms, and peer education. We adjusted for the effects of clustering in trials by Hodgins and Cohen.

Two RCTs reported on treatment rates in populations offered a screening test/visit through population-based home sampling using mailed kits [98] or either on-site or via mailed kits from a general practice clinic [109]. Screening rates were between 14 and 48%, with higher rates from invitations from health clinics compared with a population-based registry.

##### Pelvic inflammatory disease

Figure 2 shows the study findings and analyses of trial data for PID. Table 2 summarizes the main findings for the KQ1 benefit outcomes, and Additional file 6 contains all of the review’s GRADE Evidence Profiles and Summary of Findings Tables including explanations for all ratings. We did not pool results across all six trials reporting on PID because of substantial differences between screening approaches and settings.

**Table 2:** Summary of findings on benefit outcomes for screening versus no screening (key question 1)

Outcome	Study approach	Assumed population risk^**a**^	Studies; sample size^**b**^	Follow-up (yrs)	Absolute effects	Certainty^**c**^	What happens?
PID MID 2.5 fewer or more per 1000	Offer of universal CT screening, annually for 1–3 years	Study data	2 RCTs; 141,362 [8, 59]	1–3	0.1 more in 1000 (2.1 fewer to 1.5 more)	Low to moderate	Little-to-no difference
		General			0.3 more in 1000 (7.6 fewer to 11 more)	Very low	Little-to-no difference
		High			0.5 more in 1000 (13.1 fewer to 18.7 more)	Very low	Little-to-no difference
	Offer of 1 universal CT screen—selected population	Study data	1 RCT; 2607 [108]	1	11.8 fewer per 1000 (2.3 to 16.3 fewer)	Low to moderate	Small reduction
		General			15.4 fewer per 1000 (3 to 21.3 fewer)	Low to moderate	Small reduction
		High			26.8 fewer per 1000 (5.2 to 37.1 fewer)	Low	Moderate reduction
	Acceptors of 1 universal CT screen	Study data	2 RCTs [28, 105], 1 CCT [88]; 30,652	1	3.7 fewer per 1000 (7.1 fewer to 0.7 more)	Low	Small reduction
		General			5.7 fewer per 1000 (10.8 fewer to 1.1 more)	Low	Small reduction
		High			9.9 fewer per 1000 (18.8 fewer to 1.9 more)	Very low to low	Moderate reduction
Ectopic pregnancy MID 1 fewer or more per 1000	Offer of 1 universal CT screen	General	1 RCT; 15,459 [59]	9	0.20 more per 1000 (2.2 fewer to 3.9 more)	Very low	Little-to-no difference
	Acceptors of 1 universal CT screen	High	1 CCT; 28,074 [88]	1.5	0.63 more per 1000 (0.76 fewer to 2.8 more)	Very low	Little-to-no difference
Infertility—female MID 1 fewer or more per 1000	Offer of 1 universal CT screen	General	1 RCT; 15,459 [59]	9	4.2 more per 1000 (1.7 fewer to 11.2 more)	Very low	Very uncertain
	Acceptors of 1 universal CT screen	High	1 CCT; 28,074 [88]	1.5	0.15 fewer per 1000 (0.37 fewer to 0.88 more)	Very low	Very uncertain
Transmission of CT—prevalence MID 5 and 10 fewer or more per 1000	Offer of universal CT screening—annually for 1–3 years	General—both sexes	3 cluster RCTs; 41,709 [8, 9, 12]	1–3	3 fewer per 1000 (11.5 fewer to 6.9 more)	Low (5 fewer per 1000) Low to moderate (10 fewer per 1000)	Little to no difference
		General— females	2 cluster RCTs; 27,300 [8, 9]	2–3	4.1 fewer (13.5 fewer to 9.75 more)	Very low to low (5 fewer per 1000) Low (10 fewer per 1000)	Little-to-no difference
		General— males	2 cluster RCTs; 11,749 [8, 9]	2-3	7.2 fewer (18.9 fewer to 8.6 more)	Very low (5 fewer per 1000) Very low to low (10 fewer per 1000)	Very uncertain
	Offer of universal CT and NG screening—frequent screening over 2.5–4 years	High—both sexes	1 cluster CCT; 3803 [89]	2.5	26 fewer cases in 1000 (65 fewer to 68 more)	Very low (both thresholds)	Very uncertain
		High—females	1 RCT [60]; 1 CCT [89]; 6127	2.5–4	34.3 fewer per 1000 (4 to 58 fewer); NNS 29 (17 to 250)	Low to moderate (both thresholds)	Moderate reduction
		High—males	1 CCT; 1830 [89]	2.5	31 fewer per 1000 (55 fewer to 52 more)	Very low (both thresholds)	Very uncertain
Transmission of NG—prevalence MID 5 and 10 fewer or more per 1000	Offer of universal CT and NG screening—frequent screening over 2.5–4 years	High—both sexes	1 CCT; 3765 [89]	2.5	3 fewer in 1000 (5 fewer to 87 more)	Very low	Very uncertain
		High—females	1 RCT [60], 1 CCT [89]; 6000	2.5–4	5–7 fewer per 1000	Very low to low	Moderate reduction
		High—males	1 CCT; 1826 [89]	2.5	0.4 fewer in 1000 (10 fewer to 117 more)	Very low	Very uncertain

*Abbreviations*: *CCT* controlled clinical trial, *CT Chlamydia trachomatis*, *MID* minimally important difference threshold, *NG Neisseria gonorrhoeae*, *NNS* number needed to screen, *RCT* randomized controlled trial, *RR* relative risks

^a^ For PID, the absolute effects and certainty assessment the in Study Data rows used the control event rates from the studies, applying the median value when more than one study contributed to the analysis. The general-risk and high-risk assumed population risk estimates were calculated using the relative effects (RR) from the studies together with an estimate of the risk without screening, based on the natural history parameters of CT (see Additional files 3 and 6). The studies reporting on PID enrolled samples considered to be at general risk. For the other outcomes, the (median) control event rate of the studies was used for the calculations, but the level of risk of the study population (general risk ≤7% vs. high risk >7% CT prevalence at baseline) is indicated

^b^ Sample sizes for the outcome of transmission are for those participants who had this outcome assessed (e.g., through surveys), not the number enrolled in the trials. All findings from observational studies are described in Additional file 6 and provided very low certainty evidence

^c^ Reasons for ratings are explained in Additional file 6

Offering screening for CT universally, via opportunistic [8] or population-based [59] approaches achieving low screening rates, to females 16–29 years old may make little-to-no difference in risk of all-cause PID over 1- to 3-year follow-up when using assumed risks in general or high-prevalence populations. The evidence is very uncertain due to imprecision and serious indirectness from the following: use of comparison groups receiving some screening, reliance on recruitment from population registers in one trial which may not reflect primary care, and lack of complete outcome ascertainment [8, 59]. When applying an assumed population risk from the median control event rate in the studies, the evidence of this trivial effect is of higher certainty (low-to-moderate rather than very low) because of no concerns about imprecision when event rates are low (PID in 0.4–0.65% of the study control group vs. 2.7% in estimates for the general population based on natural history of CT). The data from the Hocking RCT for this analysis was based on clinic records for all patients attending the clinics during the trial period and accounted for most (approximately 80%) of the PID cases in the trial. Using hospital data for all people within the eligible age range residing in the clusters captured about 20% of the PID cases (if assuming hospital cases were not recorded in clinic charts) and indicated that there may be a reduction in PID hospitalizations (general-risk estimate 10.8 fewer per 1000 [16.2 to 0 fewer]; high-risk estimate 18.8 fewer per 1000 [28.2 to 0 fewer]), but the evidence is very uncertain.

The RCT by Scholes et al. [108] indicated that there appears to be a reduction in PID over 1-year follow-up for general-risk females showing interest in screening. Three other trials indicated that screening may reduce the risk for PID over 1 year for females 15–29 years of age who accept and undergo one CT screen in outreach settings [28, 88, 105]. When assuming a high-risk population for the effects from these studies of either selected individuals or screening acceptors, the magnitude of effects may be greater, but there is more uncertainty because of reliance for these calculations on the RR and baseline estimates of PID that were generated from data in general-risk populations. Sensitivity analyses removing the CCT by Clark did not impact findings. We used rates from patients with data at follow-up in Ostergaard because of large attrition rates in this trial; sensitivity analysis using data from all females randomized (intention-to-treat analysis) did not impact findings. Overall, the findings from these four trials are considered indirect to the main interests of the Task Force, to determine what would occur by offering primary-care based screening to unselected populations.

The evidence from observational studies [101, 111] is very uncertain about the effects of being screened for CT on PID, due to some concerns about risk of bias, serious inconsistency, and some indirectness.

##### Ectopic pregnancy

Offering a single CT screen to general-risk females, aged 21 to 24 years, may make little-to-no difference in rates of ectopic pregnancy over 9 years, but the evidence is very uncertain from reliance on a single RCT with concerns about indirectness from only using hospital diagnoses and imprecision [59]. Findings are similar from one CCT with 1.5 years follow-up for high-risk females accepting a screen [88]. The evidence from one cohort study [101] is very uncertain.

##### Infertility

The evidence is very uncertain about the effects on infertility from offering (1 RCT [59]) or for acceptors of CT screening (1 CCT [88], 1 cohort [101]). There was inconsistency between studies and serious concerns about indirectness from use of hospital data and the use of usual care comparisons. Data from the RCT was also imprecise, and that from the CCT had additional indirectness based on short-term follow-up (1.5 years) and use of an outreach setting.

##### Transmission of CT

Based on estimated population prevalence rates, offering screening to both sexes, 15–29 years old at general-risk for CT, annually for 1 to 3 years may make little-to-no difference (<5 fewer infections per 1000) in the transmission of CT when considering both sexes together [8, 9, 12]. Sensitivity analysis removing the trial [12] where we had to assume similar intervention and control group sample sizes and that reported rates of infections in the community were applicable to the population-based sample did not affect findings. There is more certainty (moderate-to-low compared with low) of the trivial effect when applying the higher threshold of 10 fewer infections per 1000 screened. Findings were similar for transmission in general-risk females only, except for having very low certainty because of more imprecision. The evidence for males is very uncertain. The one trial [8] that performed subgroup analysis based on age found no interaction effects (*p*=0.75). Findings from studies reporting on treatment rates as a proxy for transmission are similar, with low certainty that offering a single CT screen will make little-to-no difference in transmission.

Frequent offers of screening for CT and NG in high-risk females (e.g., CT prevalence 11–15%), 15–29 years old, appears to reduce CT prevalence to a moderate extent (>10 fewer per 1000) in these females [60, 89]. The evidence is of moderate-to-low certainty due to some concern about risk of bias and serious indirectness from the outreach approaches, use of usual care comparisons having some screening, and co-interventions provided in the Garcia trial. The evidence about screening high-risk populations for CT and NG on transmission of CT when considering prevalence in both sexes (1 CCT; *n*=5907) and in males only (1 CTT; *n*=1830) is very uncertain [89].

##### Transmission of NG

Frequent offers of screening for CT and NG in high-risk females (e.g., NG prevalence 2.5%) may reduce NG transmission in these females to a moderate extent (>5 fewer per 1000) [60, 89]. The evidence is very uncertain about the effects on transmission of NG across both sexes or in males, from the CCT where screening for CT and NG was offered to both sexes at high-risk [89].

#### Harms

##### Study characteristics

The Hocking RCT reported on serious adverse events from treatment through passive surveillance methods. Of the 10 uncontrolled cohort studies [81, 86, 92–95, 97, 100, 104, 114], seven reported on harms from undertaking screening and seven reported on harms from a positive diagnosis of CT after screening (Additional file 4). Four of the seven studies on screening harms only enrolled CT-negative individuals, not also those with infections but unaware of the results, such that the effects in the entire population eligible for screening may be different. Five studies enrolled about 60% females, four enrolled only females, and one did not report the sex distribution. Half of the studies enrolled what was considered a high-risk sample, either because of ≥ 7% prevalence of CT and/or a moderate or high proportion of participants reporting risk behaviors such as multiple sexual partners or previous STIs. None reported on the number identifying with groups disproportionally affected by social or other factors (e.g., FSWs, MSM, injection drug users). Mean age across studies ranged between 18 and 25 years. Two studies assessed harms in a longitudinal manner: Gottlieb et al. [94] at the testing visit and 4–6 weeks later, and Campbell et al. [86] before the invitation, during testing, and after receiving the negative results. Other studies relied on comparisons between CT positive and negative individuals. Studies were mostly lacking in long-term follow-up, with the exception of one study [104] reporting on partner break-up or violence at 1 year after diagnosis. Two studies examined harms from screening using outreach approaches [92, 100], one focused on a population-based register program [95], and the remaining seven were based on screening in primary care. Details for risk of bias by study and outcome are included in Additional file 5. Ratings of low or unclear risk of bias were given to all outcomes with the exception of data on general anxiety from a CT diagnosis in one study [81] that was at high risk due to lack of comparison with individuals without CT and inadequate data and follow-up duration.

Detailed findings, analyses, and reasons for certainty ratings for the harm outcomes are included in Additional files 4 and 6.

##### Serious adverse events from treatment during a screening intervention

The effects from screening on serious adverse events from treatment are very uncertain (1 RCT with no reports of events in 4574 receiving a CT diagnosis), due to serious concerns about risk of bias from lack of active harm surveillance and very serious concerns about imprecision due to the small sample for this very rare event [8].

##### Anxiety from screening

Over the short-term, screening for CT may make little-to-no difference in general anxiety (2 studies, *n*=2139; low certainty) [86, 94] or anxiety about one’s sexual aspects of life (2 studies, *n*=1937; low certainty for high-risk and very low certainty for general-risk individuals) [94, 97]. It may cause a small-to-moderate (50 to 400 per 1000) number of individuals to feel some degree of anxiety about their or their partner’s infertility (2 studies, *n*=450; very low certainty), although findings were inconsistent and indirect [95, 97]. Screening may make a small-to-moderate [10–46%] number of people feel some concern or anxiety about CT (based in single items on questionnaires) (2 studies, *n*=2307; very low certainty); this evidence is uncertain, particularly for men and those without risk factors for CT. Feelings of concern and worry about CT may persist after receiving a negative result.

##### Shame/stigma from screening

Over the short-term, screening for CT may make little-to-no difference in stigma manifested as low levels of overall self-esteem (2 studies, *n*=1990, low certainty) [86, 94]. One or more feelings related to stigmatization (mainly related to embarrassment and disapproval by one’s social environment) may be experienced by a small-to-moderate (60 to 300 per 1000) number of individuals (5 studies, *n*=1823, low certainty) [92, 95, 97, 100, 114], although the severity of these symptoms are unknown.

##### Relationship distress from screening

No studies reported on partner violence from screening for CT. In high-risk individuals, there may be little-to-no effect on relationship break-up as a direct consequence of undergoing screening (2 studies, *n*=445, low certainty) [94, 97]. Findings on the effects from CT screening on general relationship distress are very uncertain but suggest that responses from partners about screening may not be very negative and may be better than anticipated (2 studies, *n*=1000; very low certainty) [97, 114].

All studies reporting on psychosocial harms from screening enrolled individuals undergoing screening, who may not represent all individuals eligible to be offered a screening test. The data may therefore overestimate what will happen in the overall population eligible for a screening intervention.

##### Anxiety from a diagnosis

A CT diagnosis may make little-to-no difference in symptoms of general anxiety (2 studies, *n*=277, very low certainty) [81, 86]. A moderate-to-large (400–600 per 1000) number of individuals (mainly females) diagnosed may feel some degree of anxiety about infertility (6 studies, *n*=428, low certainty) [81, 93–95, 97, 114], and a small-to-moderate number [100–300 per 1000] may feel anxious about one’s sexual aspects of life (3 studies, *n*=359, very low certainty) [81, 94, 97]. Receiving a diagnosis of CT may cause one or more symptoms related to anxiety for a moderate-to-large (40–80%) proportion of people (3 studies, *n*=292, very low certainty), but the evidence is uncertain and duration of effects unknown.

##### Shame/stigma from a diagnosis

A CT diagnosis may make little-to-no difference for stigma manifested as low self-esteem (1 study, n=149, very low certainty) [94] but may lead to one or more stigma-related symptoms (e.g., feeling dirty, shame, embarrassment) for a moderate number (200–500 per 1000) of those diagnosed (6 studies, *n*=506, low certainty) [81, 93–95, 97, 114].

##### Relationship distress from a diagnosis

The effects on relationship violence from a diagnosis of CT are uncertain (1 study, *n*=298, very low certainty) [104], but a diagnosis may lead to relationship break-up for a small proportion (about 5–10%) of people in high-prevalence populations (4 studies, *n*=994, low certainty) [81, 94, 97, 104]. A CT diagnosis may cause some relationship distress for a small-to-moderate [100–500 per 1000] number of those diagnosed (5 studies, *n*=553, low certainty) [81, 94, 95, 97, 114].

The proportion of people within an entire screening-eligible population experiencing the harms from a diagnosis will be substantially lower (<2 to 5%).

### Comparative effectiveness of different screening strategies (key question 2)

For KQ2, we included four RCTs that compared home versus clinic sampling for screening [90, 106, 109, 115]. Study characteristic tables are in Additional file 4, and risk of bias assessment is in Additional file 5. Detailed findings, analyses, and reasons for certainty ratings for these outcomes are included in Additional file 6.

#### Study characteristics

One small RCT (*n*=205) measured incidence of CT and NG in a high-risk (17% CT) population of females after treating cases at baseline and then offering three screens over 18 months, in an outreach setting with provision of home testing kits (via mail or pick-up) or an invitation for clinic testing [90]. Three RCTs measured treatment rates in general-risk populations after various forms of recruitment: outreach via community promotion and websites [115], outreach via health clinic and community promotion [106], and postal invitations from general practice clinics [109]. All compared offers of screening at home (with mailed samples) versus screening in a primary care clinic. One of these three RCTs offered screening for CT and NG in both sexes [106], another offered screening for CT and NG in males, and another screened for CT in females [109]. Although screening was conducted at home in the intervention arms, participants had to attend clinics for treatment. All RCTs had unclear risk of bias, due to possible selection [90, 109], performance [90, 106, 109, 115], and detection biases [106, 109].

#### Transmission of CT and NG

The evidence on the effects on transmission of CT and NG from incidence rates after moderate-intensity screening using home versus clinic sampling is very uncertain (1 RCT, *n*=205, very low certainty) [90]. Findings for treatment rates of CT or NG across both sexes (1 RCT; *n*=2063; 1.9 more treated per 1000 [1.7 less to 16.3 more]) indicated that home versus clinic sampling may make little to no difference in transmission of these infections, but the evidence is uncertain [115]. The effects on transmission of CT and NG in males are very uncertain (1 RCT, *n*=200) [106], as are the effects for transmission of CT in females (1 study, *n*=260) [109]. In these studies, the rates of screening were higher for home (38–72%) versus clinic (19–48%) sampling.

### Patient values and preferences: relative importance of outcomes (key question 3)

Detailed study characteristics, risk of bias assessments, findings, analyses, and reasons for certainty ratings for this KQ3 are included in Additional files 4, 5 and 6. Table 3 summarizes the findings.

**Table 3:** Summary of findings on patient preferences (key question 3)

Study designs	Outcome Studies; sample size	Findings	Certainty of the evidence^**a**^	What does the evidence say?
Preference-based using direct (*n*=3) and indirect (*n*=1) methods to derive health-state utilities	Infertility HSUV 3; 461 [96, 110, 113]	TTO range 0.76–0.91 (SD 0.25–0.34); VAS range 0.53–0.68 (SD 0.24–0.29); indirect 0.82 **Best estimate** of utility value = 0.80 (range 0.76–0.91)	Moderate	Based on utility values, the potential benefits from screening are probably of similar importance to people.
	Chronic pelvic pain HSUV 4; 733 [96, 99, 110, 113]	TTO range 0.69–0.85 (SD 0.29–0.38); VAS range 0.45–0.61 (SD 0.29–0.38); indirect 0.60 **Best estimate** = 0.76 (range 0.69–0.85)	Moderate	
	Ectopic pregnancy HSUV 3; 461 [96, 110, 113]	TTO range 0.79–0.91 (SD 0.26–034); VAS range 0.55–0.73 (SD 0.21–0.25); indirect: out-patient 0.58 vs in-patient 0.23 with recuperation 0.60 (added to PID health state) **Best estimate** = 0.83 (range 0.79–0.91)	Low to moderate	
	PID HSUV 3; 461 [96, 110, 113]	PID out-patient: TTO range 0.82–0.90 (SD 0.22–0.33); VAS range 0.62–0.76 (SD 0.17–0.24); indirect 0.63 PID in-patient: TTO range 0.82–0.88 (SD 0.27–0.36); VAS range 0.60–0.74 (SD 0.20–0.25); indirect IPNS 0.57 vs IPS 0.46 with OPAIP 0.83 **Best estimate** (majority treated as outpatient) = 0.86 (range 0.82–0.90)	Low to moderate	
	Cervicitis HSUV 1; NR [96]	Indirect methods 0.90 (no measure of variance)	Low to moderate	
	Rank order of outcomes based on QALY loss 4; 733 [96, 99, 110, 113]	**Infertility > chronic pelvic pain >> ectopic pregnancy = PID = cervicitis** Based on range of QALY losses ((1− best estimate of utility) × duration in years) for each health state: Infertility (0.20 × 10–30 years) = 2–6 QALY loss > chronic pelvic pain (0.24 × 5–10 years = 1.2–2.4 QALY loss) >> ectopic pregnancy (0.17 for 4 weeks =0.013 QALY loss) = cervicitis (0.10 × 4 weeks = 0.008 QALY loss) = PID (0.14 × 10–12 days = 0.004 QALY loss)	Low to moderate	Infertility and chronic pelvic pain may be considerably more important to females than ectopic pregnancy, PID, and cervicitis.
Survey (*n*=1) and qualitative studies (*n*=9) providing non-utility data	Relative importance of benefits vs harms Patients mainly considering rather than undergoing CT and NG screening 777 (7 studies)	Two studies of general-risk populations found that harms from stigma of a diagnosis and (less so) anxiety from testing may outweigh the potential benefits on their reproductive health (unspecified outcomes) and transmission [82, 83]. One study’s findings indicated that a fine balance may exist between a large potential for reduced transmission and several harms, from stigma from testing, anxiety about CT, and relationship distress [84]. The remaining four studies suggested that the potential benefits from reduced transmission and (less so) improved future reproductive health will outweigh the harms from anxiety and stigma when making decisions about screening [85, 87, 107, 112]. The relative importance placed on benefits may be higher for women.	Very low	Patients considering screening (mainly females) may place more importance on the potential benefits than on the harms from screening, but the evidence is very uncertain with indication of variability. Transmission as the only benefit considered may still lead to the same assessment, as would consideration of both transmission and future reproductive health.
	Relative importance of benefits vs harms Patients who have undergone CT screening 77 (3 studies)	The potential benefits for reducing infertility and/or transmission may outweigh any (transient and mild) harms from anxiety or stigma experienced from screening, except in those getting a diagnosis where the stigma (e.g., about transmitting to others in social network) and anxiety about infertility will likely become relatively more important [91, 102, 103]. It is unclear if the harms from a diagnosis would deter people in these studies from future screening. Because of being told about the uncertain course of CT infections and duration required to cause infertility [91, 102], many women who tested positive in two studies were significantly concerned about the possibility of being infertile and distressed by their unanswered questions. One of the studies found that the harm from stigma after a diagnosis (or an anticipated one) was the main driver for regular repeat testing, to alleviate the feelings [103].	Very low	Patients who have undergone screening, and are not diagnosed with CT, may place more importance on the benefits than on the harms, but the evidence is very uncertain.

*Abbreviations*: *CT Chlamydia trachomatis*, *HSUV* health-state utility value, *IPNS* in-patient nonsurgical, *IPS* in-patient surgical, *NG Neisseria gonorrhoeae*, *OPAIP* out-patient after in-patient, *PID* pelvic inflammatory disease, *QALY* quality-adjusted life year, *SD*standard deviation, *TTO* time trade off, *VAS* visual analog scale

^a^ Reasons for ratings are explained in Additional file 6

#### Study characteristics

Four studies measured utilities for the health states of interest [96, 99, 110, 113]. Two groups of authors [110, 113] directly measured utilities for PID (treated as both in- and out-patients), ectopic pregnancy, infertility, and chronic pelvic pain, using both time trade-off (TTO) and visual analog scale (VAS) instruments and similar clinical scenarios about the outcomes’ symptoms, treatment options, complication risks (e.g., small chance of infertility from PID), and functional limitations. Smith et al. recruited 206 females (mean age 29 years) with and without a history of PID [110], whereas Trent et al. recruited adolescents (12–19 years) and their caregivers (*n*=255), most not experienced with any outcome, from medical and school health clinics [113]. Kupperman et al. used a TTO to directly measure the utility of chronic pelvic pain in females seeking care for noncancerous pelvic problems (*n*=272) [99]. A committee of the Institute of Medicine (IOM) studying priorities for vaccine development used topic expert input to indirectly measure utilities, by developing scenarios using the components of the Health Utilities Index Mark 2 tool, for several health conditions, including PID (out- and in-patient), cervicitis, chronic pelvic pain, ectopic pregnancy, and infertility [96]. The utilities were calculated using weighting from preferences of the general population in the USA. The main concerns with risk of bias across studies and outcomes were for (i) ectopic pregnancy in three studies [96, 110, 113] where the severity of the condition was thought to be underrepresented in the scenarios, (ii) PID in two studies [110, 113] from concerns about using TTO methods for temporary health states (i.e., the method assumes death follows the health state which is unrealistic for temporary states [119], and (iii) for all outcomes in the study by the IOM [96] from the use of experts rather than patients and from lack of reporting any measurement of variance in the findings.

Ten studies provided non-utility information on the relative importance of benefits and harms. Seven studies enrolled populations mainly considering rather than undertaking screening [82–85, 87, 107, 112]. Various settings were used for recruitment, including general practitioners’ offices [82], universities or vocational colleges [83–85], an emergency department [107], and STI or community health clinics [87, 112]. Five studies enrolled both sexes [83–85, 107, 112], aged between 16–29 years (one included adolescents 14–21 years old [107]), and four focused on high-risk populations [84, 85, 87, 107]. Six studies (*n*=23 to 192) [82–84, 87, 107, 112] used semi-structured or open-ended questionnaires that focused on or included questions on beliefs about benefits and harms of screening, reasons for screening/factors that influenced decision making, and/or anticipations about screening. All studies analyzed their data using qualitative methodologies. One study (*n*=278) [85] used a questionnaire based on the Theory of Planned Behavior and quantitative analysis including the correlations of beliefs and attitudes with intentions to screen. Three other qualitative studies (*n*=15 to 45) recruited participants, ages ranging from 16 to 39 years, of population-based postal [102] or primary care (STI and family planning clinics) screening programs [91, 103], in one case only including those recently diagnosed with CT [91]. Most risk of bias domains were rated low risk of bias; main concerns were that several studies lacked descriptions of how much the perceived outcomes were thought to influence screening intentions or behaviors, and that most studies did not provide an accurate representation of the realistic risks for the outcomes—for example about the rare risk for infertility—to inform participant responses (Additional file 5).

#### Health-state utilities and rank order of benefits

Values at the mid-range within values provided by the TTO methods were chosen as the best estimates of the utilities for each health state, with the exception of cervicitis where we only had the utility data from the indirect methods of the IOM study (Table 3). Variations between studies in the populations and methods did not help determine one best estimate within the range of TTO values (Additional file 6).

All of the health states probably have quite similar utility for females, without consideration of their duration. Based on estimates of QALY losses from using TTO utilities, infertility and chronic pelvic pain may be valued considerably more by females than ectopic pregnancy, PID, and cervicitis (Table 2 rank order outcome). As described in Additional file 2, there are several assumptions that need to be made when using QALY losses, including that the utilities from TTO method do not already account for duration. The rank order was similar when sensitivity analysis was applied using QALY losses based on the utility values from the IOM study’s indirect methods that estimate utility values without scenarios including a duration component.

#### Qualitative findings on relative importance of benefits versus harms

Analysis of findings indicated that more comments and stronger concerns related to the potential benefits of reducing transmission “It benefits everybody...running around spreading it” [107] and reproductive complications “To stop long term effects so I can have babies” [87] than the potential harms, mainly from anxiety “The worry of having chlamydia” [84] and stigma “I would feel a bit ashamed...didn’t pay attention...haven’t been safe” [112] from screening or a diagnosis. This evidence is very uncertain due to serious risk of bias, inconsistency, and indirectness because most harms were anticipated rather than experienced, and studies did not consider specific benefit outcomes and imprecision in three small studies of those undergoing screening. Most studies did not present participants with any estimates of the risks for health consequences such that concern and anxiety over these may be based on misperceptions, of for example overestimated risks of infertility.

## Discussion

We found that universal screening for CT, offered annually for 1 to 3 years in general populations 16 to 29 years of age using population-based (mailed invitation to screen) or opportunistic approaches in primary care, may make little-to-no difference in a females’ risk of PID (< 2.5 fewer or more cases per 1000) or ectopic pregnancy (< 1 fewer or more cases per 1000), although the evidence is very uncertain. These same approaches may make little-to-no difference for transmission (< 5 fewer or more cases per 1000) when considering both sexes together (low certainty) or for females only (low to very low certainty); evidence for transmission in males is very uncertain. Findings from studies only enrolling females interested in or accepting screening suggest that important reductions in PID (>2.5 fewer per 1000) may be attained in these scenarios. Intensive screening, at least biannually for 2–4 years, for CT and NG in high-prevalence (e.g., >11% CT and >2.4% NG) female populations may reduce transmission of CT and NG to a moderate extent (>10 fewer per 1000) within these high-risk populations, but there was no evidence on whether a risk-based approach with screening only in high-risk individuals (e.g., based on some screen for risk factors) will impact transmission in the overall population eligible for screening. Across all KQs, our assessments and interpretations of effects in general and high-risk populations were based on the baseline prevalence of infection in the study populations or on estimates of the effects when assuming different prevalence rates, with the threshold for high-risk of 7% for CT based on study, epidemiological data, and clinical input. The effects reflect a population perspective, and there was no evidence identified to directly inform one or more effective ways to choose specific individuals at increased risk to screen. Evidence was of very low certainty about the effects on transmission of CT and/or NG when considering both sexes or in males alone, or on infertility in females from offering a single CT screen. Evidence was not found for the outcomes of cervicitis, chronic pelvic pain (females), or infertility in males. No study reported on any of the clinical complications from screening for NG alone. The screening procedure, or receiving a diagnosis from screening, may cause a small proportion of the eligible population to experience harms of an uncertain duration and severity, mainly from feelings of stigmatization and anxiety especially about future risk for infertility. Offering patients screening conducted at home compared with at a clinic may make little-to-no difference in transmission, although the findings are very uncertain largely because of having to rely on treatment rates as a proxy for transmission. When using health-state utility data and accounting for the durations of each health state for the critical benefits of interest, we have low-to-moderate certainty that infertility and chronic pelvic pain are valued much more by female patients than are PID, ectopic pregnancy, and cervicitis. How patients weigh the potential benefits versus harms of screening is very uncertain, due to study limitations and inconsistency in findings, but there is some indication that risks to reproductive health and transmission are more important than the (often transient) harms of anxiety and stigmatization.

To some extent, the primary findings for PID from studies offering screening to the general population (whether accepted or not) may underestimate what could happen in practice care, mainly due to the studies’ (i) usual care comparisons involving some screening, (ii) low screening rates, and (iii) assessment of PID in the entire source population, in which some people may not be sexually active. When considering the positive effects for this outcome from studies of those interested or accepting screening, it appears that benefits may be realized if higher rates of screening are achieved. The rates of CT testing for females in the Canadian province Ontario in 2011 were 21% (15–19 years), 39% (20–24 years), and 35% (25–29 years) [120]. These numbers fell by about 5% in the year after changes to cervical cancer screening recommendations were released in 2012 by the cancer care agency in Ontario and Task Force, where the recommended starting age for screening increased to 21 and 25 years, respectively, and the frequency of screening was reduced to every 3 years [120]. The 2011 screening rates could likely be maintained or possibly surpassed if providers considered offering screening for CT during visits in addition to those involving a Papanicolaou (Pap) test [120, 121] and if they were aware of the higher than reported prevalence of CT, particularly in adolescents.

It is likely that the lower than expected screening rates in the Hocking trial were due to factors related to both providers and patients. Rates of screening completion if requested by a provider were 80%; less likely to follow through and be tested for CT were males, people aged 16–19 years, those living in areas of greater socioeconomic disadvantage, and those attending clinics without on-site pathology collection [122]. Whether a provider requested a test was likely influenced by numerous factors, including but not limited to whether a Pap smear was also provided, whether they felt comfortable or thought the context was suitable for questioning patients about sexual activity, or whether nurses as well as physicians were included in the process [123, 124]. The test positivity rates (about 10% CT) during screening in the trial were twice as high as the general prevalence rate at baseline measured through surveys; this suggests an informal selection process by health care providers for screening rather than the universal approach of the protocol, although this method did not appear to find enough cases to impact PID or prevalence [7].

The findings for effectiveness of screening are largely applicable to a broad age range from 15 to 29 years. Only one trial [8] performed subgroup analysis for differing effects on prevalence by age and found no differences. More evidence on whether the effects vary by age would be useful to help determine the best ages to start and stop universal screening.

The thresholds for an important effect, used for interpreting the magnitude and certainty of the evidence for the benefit outcomes in KQ1, were created by working group clinicians and topic experts and may not accurately reflect the patient perspective. Had different thresholds been used, the conclusions for some outcomes would be different. For example, a higher threshold for PID (e.g., ≥6 or 7 fewer cases per 1000 for a minimally important difference) would lead to findings of little-to-no difference (still having low certainty) for acceptors of screening at general-risk, and to a higher certainty of little-to-no difference for universal offering of screening to the general-risk populations. The thresholds were developed using estimates of CT prevalence in the general Canadian population and data on the natural course of the infections, recognizing there are limitations particularly from difficulties and challenges in obtaining long-term data on women with untreated infections [27].

We found very few studies comparing the effectiveness of different screening approaches (KQ2) and none that compared strategies differing by intensity. The body of evidence in KQ1 (screening versus no screening) suggests that a reduction in PID may be attainable for females interested and/or accepting one screening test with 1-year follow-up, indicates that annual testing may be sufficient. Some data suggest that more frequent testing may be more beneficial. The one trial in KQ1 that tested for CT at baseline in both study arms (freezing the samples in the control arm until study completion) found that while fewer females had PID in the screening versus control arm at 1-year follow-up, most episodes of PID (79%) in the study population occurred in females who tested negative for CT at baseline [28]. Some cases of PID likely arose in the women acquiring a CT infection over the year after screening; others were likely caused by other organisms (e.g., *Mycoplasma genitalium*, microorganisms associated with bacterial vaginosis, and respiratory and enteric pathogens) [26, 31]. Likewise, the two trials [60, 89] showing benefit for transmission also suggest that more than annual screening may be necessary. Because the study populations in these trials were at high risk for CT and NG infections (e.g., multiple sexual partners) and screening was in outreach populations, it is difficult to determine whether the effects may be attributed more to the intensity of screening or to the target population and/or setting. Duration of screening may be a key determinant that was not directly addressed in the trials. For screening programs in general, and especially when considering transmission effects, there is a lag time expected before seeing the full effect of the major outcomes averted as a result of screening [125]. After harmonization of disease-specific parameters across three modeling studies [125–127], substantial reductions in CT prevalence may require sustaining screening at low-to-moderate rates (20%) in the general population for at least 5 to 10 years [50]. Lastly, because of the transmission dynamics and sexual transmissibility of this infection, screening males may be critical to prevent CT (and its complications) in females. The screening rates for males were approximately half of those for females in the trials reporting no reduction in PID or transmission of CT [8, 9, 12, 59].

Our results about CT screening effectiveness differ somewhat from other recent systematic reviews, and much of this may have resulted from differences in inclusion criteria and analytic approach. A 2016 Cochrane review by Low et al. [65] and another review commissioned by the European Centre for Disease Prevention and Control led by the same author [27] found 32% and 34% relative reductions in PID (95% CIs 6 to 51% and 10 to 55%), respectively, from pooling four RCTs [28, 59, 105, 108] that we chose not to pool because of methodological differences (i.e., offer-to-screen versus acceptors of screening). We also included the more recent results from the Hocking RCT, which were considered the most direct of the evidence to inform the Task Force that prioritized an offer-to-screen approach. Further, although the statistical heterogeneity (*I*^2^ value 11%) was found to be low (in support of pooling the four RCTs), the differences between the magnitudes in effect when using absolute effects and when compared with a threshold (as we relied upon for assessing inconsistency) would be substantial. These authors’ findings for transmission in general populations (low certainty for little-to-no difference) were similar to ours, despite that we included the Hocking RCT results and an additional RCT [12], which did not meet their inclusion criteria. We have slightly more certainty (low-to-moderate versus their low certainty) about the findings for CT prevalence in high prevalence female populations because of the consistency found between the two studies we included [60, 89] versus their inclusion of a single study [60]. Only including RCTs, the Low et al. review did not find evidence on harms of CT screening. Neither of these reviews considered the effectiveness of screening for NG. The most recent published systematic review on this topic (2014) conducted for the United States Preventive Services Task Force focused on screening for both CT and NG in asymptomatic people [19]. This population differed from ours in that we considered eligible people not seeking testing for symptoms, and we did not require confirmation of their asymptomatic status, which can be considered a screening test in itself. Although the USPSTF review authors mention the results from two RCTs included in the previous USPSTF review (Scholes and Ostergaard, neither excluding females with symptoms), their conclusions that screening may reduce PID focused on results in a subset of the participants without symptoms at baseline in the Oakeshott RCT (relative risk, 0.39 [95% CI, 0.14 to 1.08]; received from author contact). Further, although uncontrolled studies were newly eligible for harms from screening in this update, several were excluded (*n*=4) based on the population not being asymptomatic. Many other reviews have been published, although typically focus on particular settings and/or comparisons [56, 128, 129]. The 2015 Cochrane review [128] on home versus clinic specimen collection for CT screening came to similar conclusions as ours for KQ2, when looking at case management (i.e., identification and treatment of cases) for which there may be little-to-no difference. These authors also looked at the number of persons tested using each approach, with findings varying widely across the studies, ranging from 30 to 96% in the home group and 6 to 97% in the clinic group (low-quality evidence).

Systematic reviews are threatened by risks of selective reporting bias (e.g., studies only reporting positive outcomes), publication bias—whereby unexpectedly strong results from large trials are selectively published, and selection bias. Our comprehensive search, independent review for study selection, and negative findings from several studies suggest that these factors were likely not relevant [130, 131]. Several studies had either trial registration or published protocols to help assess selective reporting and/or missing outcomes. Effect sizes in language-restricted reviews have shown to not differ significantly from those without restrictions [130, 131]. Many trials had methodological limitations introducing some risk of bias, and several aspects of the study populations, setting, interventions, and control groups introduced indirectness. Our findings for the absolute effects on PID in high-risk populations should be interpreted with caution; we are uncertain whether the RR from studies in a general-risk population, used in calculations for the absolute effects, applies to the high-risk population. Our main conclusion of very low certainty from the data on PID in trials offering screening to general-risk populations is based on absolute effects calculated using estimated control PID event rates, based on evidence on the natural history of PID and assumed CT prevalence rates, rather than the study data. Those preferring to use study data for these conclusions should note our low-to-moderate certainty of little to no difference in this situation.

Apart from the limitations of having very low to low certainty evidence across most outcomes and comparisons, the studies included in this review do not provide much if any insight on whether or how to target screening to individuals at higher risk (e.g., based on behavioral risk factors or identification with groups at disproportionate risk such as MSM or transgender people) where the benefits may be realized at an individual and population level. Studies on the benefits and harms of screening specific to these populations in primary care settings would be particularly informative, as would studies directly comparing different screening intervals and target ages, and comparing screening in both sexes versus only females.

## Conclusions

For screening benefits, most of the evidence examined about screening for CT and/or NG offers low or very low certainty about the effects on outcomes and comparisons critical for decision making about offering screening in primary care. Indirectness from use of comparison groups receiving some screening, from lack of complete outcome ascertainment, and from use of outreach settings is a major contributor to uncertainty. Prevalence of the infections and screening rates appear to be important moderators of effect on the benefits, but direct evidence on the impact on the general population from targeting screening to high-risk individuals was not found. For screening harms, although the evidence suggested screening may cause a small-to-moderate number of people to experience some degree of harm, mainly due to feelings of stigmatization and anxiety about future infertility risk, there is uncertainty about the extent, severity, and duration of harms from screening when considering the overall population eligible for screening. If preventing clinical consequences from CT infection is a priority, it appears that screening in primary care may have benefits for reducing PID and, through its natural course, long-term sequelae. The magnitude of the effects expected from screening in primary care in Canada, though, is uncertain and may require some speculation as may the degree to which the benefits outweigh the harms. Direct evidence about which screening strategies and intervals to use, which age to start and stop screening, and whether screening males in addition to females is necessary to prevent clinical outcomes is scarce, and further research in these areas would be informative. For patient preferences, the evidence indicates that the potential benefits from screening appear to outweigh the possible harms, although there may be some variability between patients. Apart from the evidence in this review, information on factors related to equity, acceptability, implementation, cost/resources, and feasibility in the Canadian context will support recommendations made by the Task Force.

## Supplementary Information

**Additional file 1.** Eligibility criteria.

**Additional file 2.** Detailed methods.

**Additional file 3.** Excluded studies.

**Additional file 4.** Study characteristics tables.

**Additional file 5.** Risk of bias assessments.

**Additional file 6.** Evidence tables.

## Data Availability

All data generated or analyzed during this study are included in this published article and its additional files.

## Abbreviations

CCT: Controlled clinical trial
CI: Confidence interval
CT: *Chlamydia trachomatis*
FSWs: Female sex workers
GRADE: Grading of Recommendations Assessment, Development and Evaluation
HIV: Human immunodeficiency virus
ICC: Intraclass correlation coefficient
KQ: Key question
MSM: Men who have sex with men
NG: *Neisseria gonorrhoeae*
OR: Odds ratio
PID: Pelvic inflammatory disease
PHAC: Public Health Agency of Canada
RCT: Randomized controlled trial
RR: Relative risk
STI: Sexually transmitted infection
