# Supplementary material for: Screening for chlamydia and/or gonorrhea in primary health care: systematic reviews on effectiveness and patient preferences
Source: Syst Rev. 2021 Apr 19;10:118. doi: 10.1186/s13643-021-01658-w (PMC8056106; doi:10.1186/s13643-021-01658-w)
Supplement: Supplementary file 1 — Additional file 1. Eligibility criteria. [file 13643_2021_1658_MOESM1_ESM.docx]

**Additional file 1: Eligibility criteria**

Note: Changes after the protocol was published are in red font. All changes occurred before study results were reviewed.

**Table 1. Eligibility criteria using PICOTS for Key Questions 1 and 2: Effectiveness and comparative effectiveness of screening approaches**

| **Criteria** | **Inclusion** | **Exclusion** |
| --- | --- | --- |
| Population | **KQ 1 & 2**: Non-pregnant sexually active individuals; studies were not excluded if all participants were not clearly identified as being sexually active  Population subgroups:   1. Population recruitment/identification strategy: clinician office (family doctor or pediatrician) vs. community health site (e.g., emergency room, school health clinic, pharmacy, sexual health/abortion/fertility clinics) vs. outreach program (e.g., field visits to homes, sex venues, bathhouses, homeless shelters, mobile vans, recreational or educational settings, online) vs. population register-based program not affiliated with health setting 2. Demographics: age (10-14, 15-19, 20-24, 25-29, 30-49, 50+ years), sex (female vs male) 3. Asymptomatic only (as determined by primary study authors) vs. all people not presenting with symptoms 4. High risk individuals based on sexual behaviours and/or other factors, as defined by authors of primary studies | - Studies focusing on pregnant people; we will not exclude studies including pregnant people. - Focus of study is on retesting cases or partner notification, where all participants have recent diagnosis (≤12 months) of chlamydia or gonorrhea - Studies with >25% presenting with STI symptoms (e.g. need to have >75% screening population) |
| Intervention | **KQ 1 & 2**: Any screening approach  Intervention subgroup: Screening for chlamydia vs. gonorrhea vs. chlamydia and gonorrhea  Screening may use any diagnostic test and treatment process for positive tests (e.g., referral to doctor, direct prescription), and may (but not necessarily) include partner notification/treatment and retesting of cases. If risk-based intervention strategy, may use any method to identify high-risk people.  Sample may be collected by clinician or patient, and either on-site or at home. Postal delivery may be used for receiving or submitting screening tests. | - If focus is on re-testing/screening or testing partners - We will not exclude studies screening for CT and/or NG as well other STIs. - Point-of-care screening tests. |
| Comparator | **KQ1**: No screening (i.e. people are not presenting with symptoms and seeking testing); usual care where some (ad hoc) screening occurs (considered indirect for comparison)  **KQ2:** Any screening comparison differing from the intervention by the following factors:     1. Universal vs. risk-based testing 2. Health care setting only: sample collection location (i.e., clinic/health care setting vs. home) 3. Outreach screening only: offered through street-based (e.g. mobile van) vs. other venues (e.g. bars, community services, bath houses, sporting events) 4. Sample collection method (i.e., NAAT vs culture; invasive [urethral or cervical swab] vs non-invasive [urine or self-collected vaginal swab]; genital vs. genital and extragenital [e.g., as determined suitable]) 5. Sample collection personnel (i.e., self vs. health care provider) 6. Screening interval (i.e., one-time vs. annual vs. other) 7. Case management approaches (i.e., retesting cases, method for partner notification/treatment)   Studies from KQ1 may be used to help answer (indirectly) KQ2, for example when effectiveness appears to differ between different studies using different screening interventions compared with no screening. |  |
| Outcomes | **KQ 1 and 2:**  **Primary Outcomes***   - 1. Chlamydia/gonorrhea infection transmission: hierarchy using (i) incidence [# new cases during follow up/#population or person-years], (ii) prevalence [# positive tests/# in population at follow up time point (if large proportion of eligible population was screened); otherwise estimated population prevalence via surveys of random/representative samples or other means)], then (iii) index case management (as reported; could include # cases receiving treatment/total population (not # cases), or also include partner notification [females and males]   2. Cervicitis [females]   3. Pelvic inflammatory disease [PID; females];   4. Ectopic pregnancy [females]   5. Chronic pelvic pain (≥6 months duration) [females]   6. Infertility: unable to conceive with unprotected sex for 12 months or longer [females and males]   7. KQ2 only: Repeat infection/reinfection (proportion having positive test ≥3 months after the index infection; measurement may not distinguish between infection due to new exposure following treatment, treatment failure/nonadherence, false positives, or lack of initial treatment)   8. Negative psychosocial impact (i.e., anxiety, sexual relationship distress including partner violence, stigmatization/shame [removed blame]) from screening procedure, or based on results a positive diagnostic test or presumptive diagnosis (i.e., regardless of test results in those with symptoms or considered at very high risk due to partner diagnosis)   9. Serious** adverse drug reaction from antibiotic treatment (e.g., anaphylaxis, QTc interval prolongation/cardiac arrhythmias, severe colitis from Clostridium Difficile, hepatic toxicity, thrombocytopenia, hemolytic anemia; requiring hospitalization)   For outcome b-f: did not have to be attributed to CT (e.g. all-cause PID included)  **Secondary Outcomes:** Factors related to feasibility, acceptability, cost and process (from studies also reporting on one or more primary outcomes)   - Feasibility/screening rates (# tests returned/# invited) - Costs - Acceptability (testing process safe, valued, preference for type of provider, sampling, setting etc.) - Barriers to testing (any reason for not completing the testing procedure) - Treatment adherence: proportion cases not initiating or completing treatment as prescribed) - Partner treatment rate: proportion of index case sex partners treated - Retesting rate |  |
| Timing | - Follow-up duration: Any duration, with exception of infection transmission and repeat infection (both ≥3 months), and as defined for outcomes of incidence of chronic pelvic pain (≥6 months) and infertility (≥12 months*)* - Study publication: 1996 – present (post NAATs) |  |
| Setting | - Any setting (indirectness to primary health care will be considered for studies where participant recruitment/identification strategy is undertaken in non-health care settings, but not criteria for inclusion) - High and Very High Human Development Index countries <http://hdr.undp.org/en/composite/HDI> |  |
| Study Design | - RCTs - Non-randomized experimental studies (i.e., studies with intervention by investigators but without randomized allocation, e.g. quasi-randomized allocation) - Controlled cohorts (prospective, retrospective, non-concurrent), controlled before-after studies, interrupted time series - If feasible and if no or very low quality evidence from first 3 design categories for outcomes ‘h’ to ‘i’, we will look for evidence for these outcomes from uncontrolled cohorts or before-after studies with ≥ 30 participants or descriptive (e.g., qualitative, surveys) studies where participants have all had experience of screening.   Reliance on controlled studies for outcomes ‘a’ to ‘g’ because of their relation to the natural history of the infections and therefore multiple potential confounders (e.g., multiple other causes of outcome) unaccounted for without a control group. | - Studies only published/available as conference proceedings or other grey literature (e.g., trial registry sites, government reports), unless information on study design (e.g., eligibility criteria, intervention and population description) is available (accessible online or via author contact) and sufficient for assessing quality/risk of bias. |
| Language | - English - French | - Non-English/French articles |

*An explanation of the process for rating outcomes for inclusion is in the protocol.

**Results in death or is life-threatening (i.e., requires inpatient hospitalization or results in prolongation of existing hospitalization; results in persistent or significant disability/incapacity; is a congenital anomaly/birth defect; is a medically important event or reaction) see https://www.ich.org/fileadmin/Public_Web_Site/ICH.../E2A/.../E2A_Guideline.pdf

**Table 2. Eligibility criteria using PICOTS for Key Question 3: Outcome valuation**

| **Criteria** | **Inclusion** | **Exclusion** |
| --- | --- | --- |
| Population | Non-pregnant sexually active individuals*  Population subgroups:   1. Population recruitment/identification strategy: clinician office (family doctor, pediatrician, nurse) vs. community health site (e.g., emergency room, school health clinic, pharmacy, sexual health/abortion/fertility clinics) vs. outreach program (e.g., field visits to homes, sex venues, bathhouses, homeless shelters, mobile vans, recreational or educational settings, online) vs. population register-based program not affiliated with health setting 2. Current or previous infection with chlamydia or gonorrhea vs. not; current or previous experience of a primary outcome vs. not 3. Demographics: age (10-14, 15-19, 20-24, 25-29, 30-49, 50+ years), sex (female vs male) 4. Asymptomatic only (as determined by authors) vs. all people not presenting with symptoms 5. High risk individuals based on sexual behaviours and/or other factors, as defined by authors of primary studies | - Pregnant women |
| Exposure | Experience with any screening program for chlamydia and/or gonorrhea; experience with infection or outcomes of interest; exposure to scenarios about screening process and possible outcomes of screening (benefits and harms)  Focus of study is on consideration of possible, or assessment of definite, outcomes from screening. Studies of patients with outcomes (e.g., pelvic inflammatory disease) do not have to exclusively enroll patients with current or history of STIs. |  |
| Comparison | Depending on study design, comparator may be no screening or another form of screening, or the study may not have a comparator. When only one arm (e.g. screening) of a comparative study is included in the assessment of patient preferences, this study will be classified as a non-comparative study. |  |
| Outcomes | - Utilities/health state valuations or other utility data (e.g., probability trade–offs, preference weights from conjoint analysis studies) - Non-utility, quantitative information on relative importance of benefits and harms (e.g., willingness to be screened, screening uptake, relative ratings/rankings) - Qualitative information indicating relative importance between benefits and harms   All outcomes will only be in relation to the primary outcomes for KQ 1 and 2. |  |
| Timing | - Follow-up duration: any or none - Published: 1996 – present (after widespread use of nucleic acid amplification test NAATs) |  |
| Setting | - Any setting - High and Very High Human Development Index countries <http://hdr.undp.org/en/composite/HDI> |  |
| Study Design | - Any experimental or qualitative study design (e.g., stated and revealed preference studies [e.g. contingent analysis or valuation studies including discrete choice experiments, willingness to pay], studies directly [e.g., time-trade-off, standard gamble] or indirectly [mapping of health status instruments to utilities using population weights/preferences] measuring health-state utility values, surveys, qualitative studies) | - Studies only published/available as conference proceedings or other grey literature (e.g., government reports), unless information on study design (e.g., eligibility criteria, participant characteristics, presentation of scenarios) is available (accessible online or via author contact) and sufficient to assess methodological quality. |
| Language | - English - French | - Non- English/French articles |

*Studies that are reporting on health state values for people with experience of the outcomes of interest (e.g., PID) that may have been caused by another infectious source do not have to only include sexually active individuals. Studies may use proxies (parents) or others (e.g. healthcare providers) to provide an estimate from the patient perspective.
