# Supplementary material for: Screening for chlamydia and/or gonorrhea in primary health care: systematic reviews on effectiveness and patient preferences
Source: Syst Rev. 2021 Apr 19;10:118. doi: 10.1186/s13643-021-01658-w (PMC8056106; doi:10.1186/s13643-021-01658-w)
Supplement: Supplementary file 2 — Additional file 2. Detailed methods. [file 13643_2021_1658_MOESM2_ESM.docx]

**Additional file 2: Detailed methods**

**Refinement of outcomes**

Three outcomes underwent further definition at the data extraction and/or analysis stage. For the outcome of transmission, the ideal measurement of prevalence in the study population at follow-up (via positivity rates from testing all enrolled participants) was usually either not undertaken or based on a small proportion of the sample, due to low screening rates. Therefore, we preferentially relied on study estimates of prevalence at follow-up, measured in people who represented the source population of each study (e.g., survey of representative or random sample of those eligible). Where population prevalence was not reported or measured in this way, we accepted positivity rates reported for a portion of the study sample but rated this outcome as being at high risk for bias when screening rates were below 50% because representation of the source population may have been poor. When using treatment rates as a proxy for transmission, we used the number of cases treated divided by the number enrolled in each arm, rather than by the number of cases.

After the protocol was published and with input from the Task Force, the original outcome of negative psychosocial impact, including blame, anxiety, stigma, relationship distress, was further defined. Blame (towards others) was changed to (self-directed) shame, and stigma and shame were combined into one outcome because of difficulties distinguishing between the two when individual symptoms were described that could fit into either category (e.g., feeling dirty or guilty). Further, after listing all of the measurement scales and individual psychosocial symptoms reported in all studies included for harms, but before analysis or presentation to the Task Force on the findings, we further defined symptoms of interest (e.g., shame included feelings of being separate or apart from others such as feeling dirty but not feeling badly) and formed sub-groupings within each main outcome (e.g., anxiety included general anxiety [using validated scales], anxiety about sexuality, anxiety about infertility, and other single symptoms of anxiety [e.g., feeling fear, worry, anxious]).

Finally, for KQ3 the outcome of health-state utilities was refined in terms of what instrument was relied upon to provide a best estimate of the utility; we also transformed the utilities to account for the duration of each health state. When available, we chose to rely on the direct measurement of utilities from TTO or standard gamble instruments rather than from VAS. Utilities from using a VAS are numerically lower than those obtained by other measures, because the VAS does not involve valuation against an external metric, such as time or risk of death. As such, the VAS is less well accepted for decision analysis (1). TTO scores have a stronger theoretical basis and are generally accepted as “true” utilities (99, 100). Because of their indirectness when following GRADE guidance, because of their incorporation of values from people other than the study participants (2), values provided from indirect methods (e.g., using health-related quality of life instruments) were extracted but only relied upon when they were the only available measure for a critical outcome. Further, because of large variation in the duration of the different health states (e.g., PID has a much shorter duration than chronic pelvic pain), we multiplied the utility values by an estimated duration of effect (using the range of durations applied in various cost-utility analyses (3)) to generate an estimated range of the quality-adjusted life year losses (QALY loss) for each state. Using these QALY loss estimates, we then determined a rank order of importance of the relevant outcomes (in this case only the different potential benefits). Our estimates of QALY losses for each health state are based on a few assumptions: 1) utility will be the same for entire duration of health state; 2) those without health state have perfect health (i.e. actual QALY loss may be higher); 3) TTO method is not affected by the duration applied to the scenario (constant proportional trade-off holds) and the value of each health state is not affected by the states that come before or after it (additive separability holds) (4).

**Analysis**

*A priori*, we did not limit inclusion to only studies designed or analyzed using an intention-to-screen approach (e.g., offering an invitation to eligible individuals to screen with analysis of the entire sample regardless of screening participation). Studies only reporting a per protocol analysis or using a design only enrolling those individuals actually screened (e.g., *acceptors* of screening) were also included, but this distinction was accounted for in the analysis and interpretation of the data.

When meta-analysis was possible and appropriate, due to similarity in populations, outcomes, and interventions, we used a DerSimonian Laird random effects model using Review Manager Version 5.3 (The Cochrane Collaboration, Copenhagen, Denmark). We did not use the Peto OR method for rare events (<1-2%) because control groups were of unequal sizes or there was a large magnitude of effect; in these cases the Mantel-Haenszel method without correction factor was used (5). When results were not combined using meta-analysis due lack of common measurement (e.g., harms data), we used narrative descriptions of each study for our analysis and interpretation. For studies reporting on harms in KQ1 and for KQ3, we tabulated all findings and compared and contrasted study findings by study methodology (e.g., timing of outcome measurement), populations, outcome presentations provided to participants (relevant only for KQ3), and analysis. Findings based on key differences between studies were also created; for example, KQ3 findings from non-utility studies were separated based on whether participants were considering or had already participated in a screening program.

For dichotomous outcomes, we report relative risks (RR) or odds ratios (OR) between groups with corresponding 95% confidence intervals (95% CI). When ORs were used for the analysis we calculated RR using the control event rate. We also calculated the absolute risk reduction and risk differences, based on GRADE guidance (6). For trials not accounting for clustering effects in their analysis, we adjusted for the effect of the cluster design using an interclass correlation coefficient of 0.028 (7). For one RCT (8) that used reported rates of CT in the communities one year after the intervention for prevalence, we assumed that the population contributing to the reported rates was the same as that included in the population-based sample invited to screen (all 15-39 year olds living in the community; number provided for each intervention cluster); the authors did not report the sample size in the control clusters, but reported matching communities based on size, so we assumed equal sample sizes. We preferred to use intention-to-screen analyses (i.e., using the number of people allocated to each group as the denominator), although for one RCT where there was large (>50%) attrition we used the number reporting presence or absence of the outcome (self-reported PID) for the denominator (9).

Variables thought to possibly influence the effectiveness of screening for different sub-populations, based on clinical input, included: recruitment strategy (clinician office vs. community health centre vs. outreach vs. population register-based), age (10-14, 15-19, 20-24, 25-29, 30-49, ≥50 years), sex, proportion asymptomatic, and risk status (general vs. high/elevated risk for CT and NG). We intended to assess risk status by participant reports of sexual behaviors and/or other risk factors, but we needed to rely on CT or NG baseline prevalence in the studies to categorize studies as enrolling populations at general or high-risk. Based on the baseline prevalence in the trials of general populations (4-6%), consideration that Canadian statistics (of about 1-2.5% CT) represent underreporting by about 70%, and after input from the Task Force and content experts, greater than 7% CT prevalence at baseline was used as the threshold for an increased-risk study population. As anticipated at the protocol stage, in addition to using the study control event rates (medians when multiple studies were in the analysis) for calculating absolute effects, we also made calculations to estimate—relying on natural history parameters—the effects (assumed/illustrative risks) for both general- and high-risk (prevalence) populations for the PID outcome. The number of PID cases without screening for the general-risk population (2.7%) assumed that approximately 6% of the female population would have CT (baseline prevalence), that between 10-16% (about 13%) of these females would develop PID (10), and that approximately 25-30% (11) of all cases of PID are attributed to CT. The baseline risks for the high-risk population (4.7%) assumed a higher (12%) CT prevalence, and slightly higher (33%) attribution to CT. We did not calculate any estimates based on differing risk status for the other reported outcomes of ectopic pregnancy or infertility because of much more variability and uncertainty about the natural history parameters. For the outcome of transmission, the studies naturally separated into groups based on risk status/CT prevalence when also considering the differences in the interventions based on intensity (frequency and duration) of screening. There was initial consideration of pooling data from studies with high screening rates from an offer-to-screen design with studies only enrolling acceptors of screening, based on the studies all having “implausible” rates of screening (i.e., ≥ 50% screening rate based on input from topic experts and looking at evidence used in economic analysis), but upon further scrutiny it was decided that there was too much clinical and methodological heterogeneity between the studies of an offer versus acceptors of screening.

Sensitivity analysis was conducted based on risk of bias and study design. If there had been at least eight studies of varying size in a meta-analysis, we would have analyzed for publication bias both visually using the funnel plot and quantitatively using Egger’s test (12).

**Assessing certainty using thresholds**

Before presenting findings, the Working Group and content experts created thresholds, based on their clinical judgement and natural history parameters, for a minimally/small importance difference (MID) in effect (to indicate either an important benefit [fewer cases] or harm [more cases]) for several outcomes: PID, 2.5 fewer or more cases per 1000; ectopic pregnancy and infertility, 1 fewer or more per 1000; CT and NG transmission, 5 fewer or more per 1000 (10 fewer or more per 1000 was determined to be a moderate effect) when using prevalence rates, and 20 more or fewer per 1000 when using treatment rates as a proxy for transmission. For clinical outcomes, natural history parameters (see Background) were used to develop the thresholds: we assumed that, without screening, 6% of the general population would have CT and about 13% of the those with untreated CT would experience PID, which corresponds to about 0.8 to 1% (8 to 10 per 1000) of the screening population getting PID. The Working Group agreed that about a 25-30% reduction in this value (i.e., 2.5 fewer per 1000) would be important. Similar exercises were undertaken for ectopic pregnancy and infertility (both with roughly 3% in untreated CT), except that somewhat larger relative effects were used (about 50% reduction) due to the rare nature of these outcomes.

In general, we used the location of our best estimate of the absolute effect (point estimate if meta-analysis was undertaken) relative to the threshold to determine what we were rating our certainty for (e.g., a small effect if exceeding the threshold versus a trivial effect/little-to-no difference if below the threshold). We then used the 95% CI of the estimate relative to the threshold to assess the precision of the effect. We did not base our assessments of precision on the null/statistical significance. When there were multiple studies in an analysis, the magnitude of the absolute effects of individual studies with respect to the threshold was also used when deciding whether to rate down for study limitations/risk of bias (e.g., when removing a high risk-of-bias study changed conclusions) and/or for inconsistency (e.g., from studies with outlying effects possibly from clinical or methodological differences, per our predefined subgroups). We considered several factors for rating down for indirectness, including the recruitment method (outreach being indirect to the primary care population), use of usual care (some ad hoc screening vs. no screening) in the control arms of trials, poor outcome ascertainment (e.g., use of hospital records failing to capture many cases of PID or ectopic pregnancy), and the use of the natural history parameters (i.e., risk of PID in untreated CT and proportion of PID attributable to CT) and relative effects from studies in general-risk populations to calculate estimates for high-risk populations (for PID outcome). None of these reasons were considered in the assessment of risk of bias as they were considered instead for indirectness. For KQ3, we relied on GRADE guidance published after the protocol publication (2, 13). For the KQ3 findings, because causation from the intervention is not relevant to valuation of outcomes, we did not rely on RCTs for obtaining high certainty evidence.

**References**

1. Smith KJ, Tsevat J, Ness RB, Wiesenfeld HC, Roberts MS. Quality of life utilities for pelvic inflammatory disease health states. Sex Transm Dis. 2008;35:307-11.

2. Zhang Y, Alonso-Coello P, Guyatt GH, Yepes-Nunez JJ, Akl EA, Hazlewood G, et al. GRADE Guidelines: 19. Assessing the certainty of evidence in the importance of outcomes or values and preferences-risk of bias and indirectness. J Clin Epidemiol. 2019;111:94-104.

3. Jackson LJ, Auguste P, Low N, Roberts TE. Valuing the health states associated with Chlamydia trachomatis infections and their sequelae: a systematic review of economic evaluations and primary studies. Value Health. 2014;17:116-30.

4. Tsuchiya A, Dolan P. The QALY model and individual preferences for health states and health profiles over time: a systematic review of the literature. Med Decis Making. 2005;25(4):460-7.

5. Fu R, Gartlehner G, Grant M, Shamliyan T, Sedrakyan A, Wilt TJ, et al. Conducting quantitative synthesis when comparing medical interventions: AHRQ and the Effective Health Care Program. J Clin Epidemiol. 2011;64(11):1187-97.

6. Schunemann H, Brozek J, Guyatt G, Oxman A (Eds): GRADE Handbook 2013. <https://gdt.gradepro.org/app/handbook/handbook.html#h.wsfivfhuxv4r>. Accessed 14 Sept 2020.

7. Glassman JR, Potter SC, Baumler ER, Coyle KK. Estimates of intraclass correlation coefficients from longitudinal group-randomized trials of adolescent HIV/STI/Pregnancy Prevention Programs. Health Educ Behav. 2015;42(4):545-53.

8. Hodgins S, Peeling RW, Dery S, Bernier F, LaBrecque A, Proulx JF, et al. The value of mass screening for chlamydia control in high prevalence communities. Sex Transm Infect. 2002;78 Suppl 1:i64-8.

9. Ostergaard L, Andersen B, Moller JK, Olesen F. Home sampling versus conventional swab sampling for screening of Chlamydia trachomatis in women: a cluster-randomized 1-year follow-up study. Clin Infect Dis. 2000;31(4):951-7.

10. Price MJ, Ades AE, De Angelis D, Welton NJ, Macleod J, Soldan K, et al. Risk of pelvic inflammatory disease following Chlamydia trachomatis infection: analysis of prospective studies with a multistate model. Am J Epidemiol. 2013;178(3):484-92.

11. Davies B, Turner KM, Leung S, Yu BN, Frolund M, Benfield T, et al. Comparison of the population excess fraction of Chlamydia trachomatis infection on pelvic inflammatory disease at 12-months in the presence and absence of chlamydia testing and treatment: systematic review and retrospective cohort analysis. PLoS ONE. 2017;12(2):e0171551.

12. Egger M, Davey Smith G, Schneider M, Minder C. Bias in meta-analysis detected by a simple, graphical test. BMJ. 1997;315(7109):629-34.

13. Zhang Y, Coello PA, Guyatt GH, Yepes-Nunez JJ, Akl EA, Hazlewood G, et al. GRADE guidelines: 20. Assessing the certainty of evidence in the importance of outcomes or values and preferences-inconsistency, imprecision, and other domains. J Clin Epidemiol. 2019;111:83-93.
