# Supplementary material for: Screening for chlamydia and/or gonorrhea in primary health care: systematic reviews on effectiveness and patient preferences
Source: Syst Rev. 2021 Apr 19;10:118. doi: 10.1186/s13643-021-01658-w (PMC8056106; doi:10.1186/s13643-021-01658-w)
Supplement: Supplementary file 3 — Additional file 3. Excluded studies. [file 13643_2021_1658_MOESM3_ESM.docx]

**Additional file 3: Excluded studies**

**POPULATION**

1. Adams EJ, Turner KM, Edmunds WJ. The cost effectiveness of opportunistic chlamydia screening in England. Sex Transm Infect. 2007;83:267-74.

2. Allen-Davis JT, Parker R, McGregor J, Beck A, McClatchey MW. Assessment of vulvovaginal complaints: agreement between phone and office management. Prim Care Update Ob Gyns. 1998;5:152.

3. Andersen B, Ostergaard L, Moller JK, Olesen F. Home sampling versus conventional contact tracing for detecting Chlamydia trachomatis infection in male partners of infected women: randomised study. BMJ. 1998;316:350-1.

4. Angel G, Horner PJ, O'Brien N, Sharp M, Pye K, Priestley C, et al. An observational study to evaluate three pilot programmes of retesting chlamydia-positive individuals within 6 months in the South West of England. BMJ Open. 2015;5:E007455.

5. Artis SM. Routine chlamydia screening in community health centers: Mixed methods approach. PhD [dissertation]. Washington: George Washington University; 2013. Available from: Dissertation Abstracts International: Section B: The Sciences and Engineering.

6. Banerjee P, Thorley N, Radcliffe K. A service evaluation comparing home-based testing to clinic-based testing for Chlamydia and gonorrhoea in Birmingham and Solihull. Int J STD AIDS. 2018;29:974-9.

7. Bell G, Kernec M. Workload, costs and outcomes for managing positive results and partner notification for chlamydia by telephone. HIV Med. 2010;11:85.

8. Blake M. "Undesirable": The impact of incurable STIs on single, young women. PhD [dissertation]. 2007. Available from: Dissertation Abstracts International: Section B: The Sciences and Engineering.

9. Byrne R, Cooper F, Appleby T, Chislett L, Freeman L, Kershaw E, et al. Can express treatment reduce onward transmission? Sex Transm Infect. 2015;91:A7.

10. Cassell JA, Dodds J, Estcourt C, Llewellyn C, Lanza S, Richens J, et al. The relative clinical effectiveness and cost-effectiveness of three contrasting approaches to partner notification for curable sexually transmitted infections: a cluster randomised trial in primary care. Health Technol Assess. 2015;19(5):1-115, vii-viii.

11. Centers for Disease Control and Prevention. The Participant Agreement for Contact Tracing (PACT) Study: Enhancing Partner Notification Services. 2005. ClinicalTrial.gov registration number: NCT00207493.

12. Chan KHN, Ho KM, Lo KK. Partner notification for gonorrhoea in Hong Kong. Hong Kong J Dermatol Venereol. 2008;16:5-11.

13. Cheserem E, Stevenson J, Evason R, Brady M. Gonorrhoea test of cure: Outcomes in a large urban sexual health service. Sex Transm Infect. 2012;88:A1-2.

14. Christianson M, Boman J, Essen B. 'Let men into the pregnancy'--men's perceptions about being tested for chlamydia and HIV during pregnancy. Midwifery. 2013;29:351-8.

15. Christianson M, Boman J, Essen B. "Men don't think that far" - Interviewing men in Sweden about chlamydia and HIV testing during pregnancy from a discursive masculinities construction perspective. Sex Reprod Healthc. 2017;12:107-15.

16. Clark JL, Segura ER, Oldenburg C, Rios J, Montano SM, Salvatierra J, et al. Patient-delivered partner therapy (PDPT) increases the frequency of partner notification among MSM in Lima, Peru: A randomised clinical trial. Sex Transm Infect. 2015;91(Suppl 2):A48-9.

17. Connolly NB, Hamer S, Ward M, Mellor J, McQuillan O. Improving management of pelvic inflammatory disease by using a simple tick-box sticker. Sex Transm Infect. 2012;88(Suppl 1):A29-30.

18. Deak J, Nagy E, Vereb I, Meszaros G, Kovacs L, Nyari T, et al. Prevalence of Chlamydia trachomatis infection in a low-risk population in Hungary. Sex Transm Dis. 1997;24:538-42.

19. Deihl TE, Updike GM, Nagle W, Wiesenfeld HC. Std tracker reminder system increases repeat testing following treatment for chlamydia or gonorrhea. Sex Transm Dis. 2016;43(10 Supplement 2):S127.

20. Dennehy D, Whitlock G, Patel S, McOwan A, Nwokolo N. Gonorrhoea test-of-cure by post maintains return rate. Sex Transm Infect. 2015;91(Suppl 1):A9.

21. Desai M, Burns F, Mercey D, Nardone A, Muniina P, Sharp T, et al. Active recall of men who have sex with men (MSM) for an HIV/STI testing: A feasible and effective strategy? HIV Med. 2014;15(Suppl 3):109.

22. Estcourt C, Sutcliffe L, Mercer C, Copas A, Muniia P, Rait G, et al. Can accelerated partner therapy (APT) improve outcomes of partner notification for women diagnosed with genital chlamydia in primary care settings: A pilot randomized controlled trial in general practice and community sexual health services. Sex Transm Dis. 2014;41(Suppl 1):S32-3.

23. Fagan P. Sexual health service provision in remote Aboriginal and Torres Strait Islander settings in Far North Queensland: Sexual health symptoms and some outcomes of partner notification. Venereology. 2001;14:55-61.

24. Failor CM, Budrys NM, Shain RN, Robinson RD. Contraception use and the risk of recurrent sexually transmitted infection in high risk, minority women. Fertil Steril. 2013;100:S316.

25. Falk L, Hegic S, Wilson D, Wirehn AB. Home-sampling as a tool in the context of Chlamydia trachomatis partner notification: a randomized controlled trial. Acta Derm Venereol. 2014;94:72-4.

26. Frye JC, Wallace L, Chavez RS, Luce DA. Quality assessment of Chlamydia trachomatis screening and treatment in a juvenile detention center. J Correct Health Care. 2008;14:99-108.

27. Gannon-Loew KE, Holland-Hall C, Bonny AE. Expedited partner therapy: Adolescents' acceptance of a partner treatment method. J Adolesc Health. 2018;62:S83.

28. Gannon-Loew KE, Holland-Hall C, Lange HLH, Bonny AE. Chlamydia and Trichomoniasis Reinfection Rates in Adolescents: Evidence of Continued Need for Intervention. J Pediatr Adolesc Gynecol. 2017;30:318-9.

29. Garcia CM, Ptak SJ, Stelzer EB, Harwood EM, Brady SS. "I connect with the ringleader:" Health professionals' perspectives on promoting the sexual health of adolescent males. Res Nurs Health. 2014;37:454-65.

30. Gift T, Kissinger P, Mohammed H, Leichliter J, Hogben M, Golden M. The cost of expedited partner therapy compared to the cost of standard partner referral for the treatment of chlamydia or gonorrhoea. Sex Transm Infect. 2011;87(Suppl 1):A62.

31. Gift TL, Pate MS, Hook EW, Kassler WJ. The rapid test paradox: when fewer cases detected lead to more cases treated - a decision analysis of tests for Chlamydia trachomatis Sex Transm Dis. 1999;26:232-40.

32. Gilbert M, Salway Hottes T, Chabot C, Haag D, Shoveller J, Ogilvie G. "There are a million scenarios to consider": Health care provider perspectives on internet-based testing for sexually transmitted infections, HIV, and hepatitis C in British Columbia. Sex Transm Infect. 2013;89:A350.

33. Gillespie GL, Reed J, Holland CK, Munafo JK, Ekstrand R, Britto MT, et al. Pediatric emergency department provider perceptions of universal sexually transmitted infection screening. Adv Emerg Nurs J. 2013;35:76-86.

34. Ginocchio RH, Veenstra DL, Connell FA, Marrazzo JM. The clinical and economic consequences of screening young men for genital chlamydial infection Sex Transm Dis. 2003; 30:99-106.

35. Golden M, Kerani R, Stenger M, Hughes J, Aubin M, Malinski C, et al. Uptake and population-level impact of expedited partner therapy (EPT) on Chlamydia trachomatis and Neisseria gonorrhoeae: the Washington State community-level randomized trial of EPT. PLoS Med. 2015;12:E1001777.

36. Hocking JS, Parker RM, Pavlin N, Fairley CK, Gunn JM. What needs to change to increase chlamydia screening in general practice in Australia? The views of general practitioners. BMC Public Health. 2008;8:425.

37. Jayaweera R, Loke W. Audit of the diagnosis and management of gonorrhoea in a genitourinary medicine clinic. HIV Med. 2014;15(Suppl 3):133.

38. Kerns J, Jones H, Fratarelli L, Pressman E, Tiezzi L, Westhoff C. Implementing patient-delivered partner therapy for chlamydia infection at an Urban family planning clinic. Contraception. 2009;80:222.

39. Kissinger PJ, Reilly K, Taylor SN, Leichliter JS, Rosenthal S, Martin DH. Early repeat Chlamydia trachomatis and Neisseria gonorrhoeae infections among heterosexual men. Sex Transm Dis. 2009;36:498-500.

40. Knapper C, Murphy M, Collett M, Browning M. Problematic partner notification for gonorrhoea in a city GUM clinic. HIV Med. 2010;11(Suppl 1):91.

41. Lemmers M, Verschoor MAC, Oude Rengerink K, Naaktgeboren C, Bossuyt PM, Huirne JAF, et al. MisoREST: Surgical versus expectant management in women with an incomplete evacuation of the uterus after misoprostol treatment for miscarriage: A cohort study. Eur J Obstet Gynecol Reprod Biol. 2017;211:83-9.

42. Lorch R, Hocking J, Guy R, Vaisey A, Wood A, Donovan B, et al. Do Australian general practitioners believe practice nurses can take a role in chlamydia testing? A qualitative study of attitudes and opinions. BMC Infect Dis. 2015;15:31.

43. Lorch R, Hocking J, Guy R, Vaisey A, Wood A, Lewis D, et al. Practice nurse chlamydia testing in Australian general practice: a qualitative study of benefits, barriers and facilitators. BMC Fam Pract. 2015;16:36.

44. Low N, McCarthy A, Roberts TE, Huengsberg M, Sanford E, Sterne JA, et al. Partner notification of chlamydia infection in primary care: randomised controlled trial and analysis of resource use. BMJ. 2006;332:14-9.

45. Maastricht University Medical Center. Transmission of Genital and Extra-genital Chlamydia Trachomatis Infections in Women (FemCure). 2016. ClinicalTrials.gov registration number: NCT02694497.

46. McNulty CAM, Freeman E, Howell-Jones R, Hogan A, Randall S, Ford-Young W, et al. Overcoming the barriers to chlamydia screening in general practice-a qualitative study. Fam Pract. 2010;27:291-302.

47. Nack AL. Damaged goods: The sexual self-transformations of women with chronic STDs. PhD [dissertation]. Colorado: University of Colorado; 2001. Available from: Dissertation Abstracts International Section A: Humanities and Social Sciences.

48. Ong JJ, Peng M, Zhu S, Lo YJ, Fairley CK, Kidd MR, et al. Opportunities and barriers to STI testing in community health centres in China: a nationwide survey. Sex Transm Infect. 2017;93:566-71.

49. Ostergaard L, Andersen B, Moller JK, Olesen F, Worm AM. Managing partners of people diagnosed with Chlamydia trachomatis: a comparison of two partner testing methods. Sex Transm Infect. 2003;79:358-61.

50. Paavonen J, Puolakkainen M, Paukku M, Sintonen H. Cost-benefit analysis of first-void urine Chlamydia trachomatis screening program. Obstet Gynecol. 1998;92:292-8.

51. Percy L, Langley K, Harrison E, Sankar N, Michell L. Extra-genital chlamydia testing in heterosexual patients. Is it worth it? Sex Transm Infect. 2015;91:A24.

52. Percy L, Langley K, Harrison E, Sankar N, Mitchell L. Extra-genital gonorrhoea testing in heterosexual patients. Is it worth it? Sex Transm Infect. 2015;91:A24-5.

53. Pickett ML, Melzer-Lange MD, Miller MK, Menon S, Visotcky AM, Drendel AL. Perceived Patient Preference and Clinical Testing for Chlamydia and Gonorrhea in Females: How Closely Are These Aligned? Clin Pediatr (Phila). 2018;57:106-8.

54. Reed JL, Huppert JS, Gillespie GL, Taylor RG, Holland CK, Alessandrini EA, et al. Adolescent patient preferences surrounding partner notification and treatment for sexually transmitted infections. Acad Emerg Med. 2015;22:61-6.

55. Rosenfeld EA, Marx J, Terry MA, Stall R, Pallatino C, Miller E. Healthcare providers' perspectives on expedited partner therapy for chlamydia: a qualitative study. Sex Transm Infect. 2015;91:407-11.

56. Scott C, Teague A, Menon-Johanssen A, Jones R, Sullivan A. A study to assess acceptability of partner notification via Short Message Service text messaging (SMS). HIV Med. 2010;11:49.

57. Smith KJ, Cook RL, Roberts MS. Time from sexually transmitted infection acquisition to pelvic inflammatory disease development: influence on the cost-effectiveness of different screening intervals. Value Health. 2007;10:358-66.

58. Smith KS, Guy R, Danielewski J, Tabrizi SN, Fairley CK, McNulty AM, et al. Biological and Behavioral Factors Associated With Positive Chlamydia Retests. Sex Transm Dis. 2017;44:417-22.

59. Smith KS, Hocking JS, Wand H, Chen M, Fairley CK, Bradshaw CS, et al. Home-based sample collection increases chlamydia retesting and detects additional repeat positive tests: A randomised controlled trial in three risk groups. Sex Trans Infect. 2013;89(Suppl 1):A70-1.

60. Smock L, Barker K, Hsu K. Expedited partner therapy for chlamydia infection is underreported and underutilized, Massachusetts 2012. Sex Transm Dis. 2014:S19-20.

61. Sparks R, Helmers JR, Handsfield HH, Totten PA, Holmes KK, Wroblewski JK, et al. Rescreening for gonorrhea and chlamydial infection through the mail: a randomized trial. Sex Transm Dis. 2004;31:113-6.

62. Tomnay JE, Gebert RL, Fairley CK. A survey of partner notification practices among general practitioners and their use of an internet resource for partner notification for Chlamydia trachomatis. Sex Health. 2006;3:217-20.

63. Tomnay JE, Pitts MK, Fairley CK. General practitioners' use of internet-based patient materials for partner notification. Sex Transm Dis. 2007;34:613-6.

64. University of Bristol. Partner Notification for Chlamydia in Primary Care. 2005. ClinicalTrials.gov registration number: NCT00112255.

65. University of Maryland. Use of a Rapid Turnaround Test for NG/CT to Improve Treatment of Women Presenting With Possible STIs. 2017. ClinicalTrials.gov registration number: NCT03098394.

66. Vacca SH. Patient Delivered Expedited Partner Therapy for Chlamydia Trachomatis among Adolescent Females Using School Based Health Centers. PhD [dissertation]. Pennsylvania: Villanova University; 2017. <https://pqdtopen.proquest.com/doc/1904950809.html?FMT=ABS>. Accessed 10 Sept 2020.

67. de Vries R, Bergen JE, Jong-van dBLT, Postma MJ. Cost-utility of repeated screening for chlamydia trachomatis Value Health. 2008;11:272-4.

68. Walleser S, Salkeld G, Donovan B. The cost effectiveness of screening for genital Chlamydia trachomatis infection in Australia. Sex Health. 2006;3:225-34.

69. Wanje GH, Masese L, Avuvika E, Omoni G, Baghazal A, McClelland RS. Parents' and teachers' views on adolescents' sexual health to inform the development of a screening intervention for sexually transmitted infections: A qualitative study. Sex Transm Infect. 2015;2:A95.

70. Welte R, Kretzschmar M, Leidl R, Hoek A, Jager JC, Postma MJ. Cost-effectiveness of screening programs for Chlamydia trachomatis: a population-based dynamic approach. Sex Transm Dis. 2000;27:518-29.

71. Whitlock G, Byrne R, Cooper F, McOwan A. A novel model of care incorporating self-directed care and rapid results management successfully reaches high-risk men who have sex with men. Int J STD AIDS. 2015;1:88.

72. Wilson SR, Brown NL, Leyden WA, Manos MM, Chin V, Levin D, et al. Healthcare utilization by women in a comprehensive managed care population subsequent to diagnosis of a sexually transmitted disease. Sex Transm Dis. 2002;29:678-88.

73. Wilson TE, Uuskula A, Feldman J, Holman S, Dehovitz J. A case-control study of beliefs and behaviors associated with sexually transmitted disease occurrence in Estonia. Sex Transm Dis. 2001;28:624-9.

74. Wiskin C, Roberts L, Roalfe A. The impact of discussing a sexual history in role-play simulation teaching on pre-clinical student attitudes towards people who submit for STI testing. Med Teach. 2011;33:E324-32.

75. Xu F, Stoner B, Taylor S, Mena L, Tian L, Papp J, et al. Rescreening for chlamydial infection using home-based, self-obtained vaginal swabs: A randomised controlled trial in family planning clinic clients. Sex Transm Infect. 2011;87:A75-6.

76. Xu F, Stoner BP, Taylor SN, Mena L, Tian LH, Papp J, et al. Use of home-obtained vaginal swabs to facilitate rescreening for Chlamydia trachomatis infections: two randomized controlled trials. Obstet Gynecol. 2011;118(2 Pt 1):231-9.

77. Yeung A, Hocking J, Vaisey A, Lorch R, Guy R, Fairley CK, et al. "It opened my eyes"-examining the impact of the australian chlamydia control effectiveness pilot (ACCEPt) on chlamydia testing practices of general practitioners. Sex Transm Infect. 2015;91(Suppl 2):A101-2.

78. Zogg NA. Effect of follow-up mail reminders on increased timely detection of chlamydia trachomatis persistent or recurrent infections. PhD [dissertation]. Minnesota: Walden University; 2013. Available from: Dissertation Abstracts International: Section B: The Sciences and Engineering.

**INTERVENTION/EXPOSURE**

1. Adam PC, de Wit JB, Bourne CP, Knox D, Purchas J. Promoting regular testing: an examination of HIV and STI testing routines and associated socio-demographic, behavioral and social-cognitive factors among men who have sex with men in New South Wales, Australia. Aids Behav. 2014;18:921-32.
2. Ahmad FA, Jeffe DB, Plax K, Schechtman KB, Doerhoff DE, Garbutt JM, et al. Characteristics of youth agreeing to electronic sexually transmitted infection risk assessment in the emergency department. Emerg Med J. 2018;35:46-51.
3. Ahmad FA, Plax K, Collins KK, Jeffe DB, Schechtman KB, Garbutt J, et al. Computer-assisted self-interviews improve testing for chlamydia and gonorrhea in the pediatric emergency department. Acad Emerg Med. 2012;19(Suppl 1):S352
4. Aicken C, Mercer C, Keane F, Estcourt C, Brook G, Armstrong N, et al. Care pathways to GUM: Is general practice now helping or hindering? Evidence from the MSTIC (Maximising STI Control in local populations) study. HIV Med. 2010;11(Suppl 1):95.
5. Al-Mousa H, Abouelhoda M, Monies DM, Al-Tassan N, Al-Ghonaium A, Al-Saud B, et al. Unbiased targeted next-generation sequencing molecular approach for primary immunodeficiency diseases. J Allergy Clin Immunol. 2016;137:1780-7.
6. Alfonsi G, Eggert J, Edel M, Bell D, Mettenbrink C, Shlay J. Provision of intrauterine devices in an STD clinic setting. Sex Transm Dis. 2014;41:S7.
7. Ali H, Guy RJ, Fairley CK, Wand H, Chen MY, Dickson B, et al. Understanding trends in genital Chlamydia trachomatis can benefit from enhanced surveillance: Findings from Australia. Sex Transm Infect. 2012;88:552-7.
8. Alicea-Alvarez N, Hellier SD, Jack LW, Lundberg GG. A pilot study of chlamydia screening among high school girls. J Nurse Pract. 2011;7:25-8
9. Allan S. Improving clinical standards in GU medicine: A retrospective audit of Neisseria gonorrhoea 2007-2015. Sex Transm Infect. 2016;92(Suppl 1):A37-8.
10. Allsworth J, Graseck A, Secura G, Madden T, Peipert J. Experiences of discrimination and STI testing in white and black women. Contraception. 2010;82:210
11. American Academy of Pediatrics. AAP Section on Emergency Medicine Scientific Abstracts and Posters National Conference and Exhibition 2013. Proceedings of the Pediatric Emergency Care Conference; 2013 Oct 25-29; Orlando FL. Illinois: AAP; 2013.
12. Andrade RF, Araujo MA, Vieira LJ, Reis CB, Miranda AE. Intimate partner violence after the diagnosis of sexually transmitted diseases. Rev Saude Publica. 2015;49:3.
13. Apostoli A, Carvalho A, Odolini S, Benedetti S, Dal Zoppo S, Izzo I, et al. Incidence of C. trachomatis genital infections in a cohort of HIV infected women. Infection. 2011;39:S43-4.
14. Atherly A, Blake SC. Efforts by commercial health plans to increase Chlamydia trachomatis screening among their members. Sex Transm Dis. 2013;40:55-60.
15. Atherton H, Banks D, Harbit R, Long L, Chadd F, Hay P, et al. Recruitment of young women to a trial of chlamydia screening - as easy as it sounds? Trials. 2007;8:41
16. Atherton H, Oakeshott P, Aghaizu A, Hay P, Kerry S. Use of an online questionnaire for follow-up of young female students recruited to a randomised controlled trial of chlamydia screening. J Epidemiol Community Health. 2010;64:580-4.
17. Azzopardi PS, Kennedy EC, Brown AD. Online chlamydia testing: an innovative approach that appeals to young people. Med J Aust. 2012;197:621.
18. Backonja U, Royer HR, Lauver DR. Young women's reasons to seek sexually transmitted infection screening. Public Health Nurs. 2014;31:395-404.
19. Baird J, Merchant RC. A randomized controlled trial of the effects of a brief intervention to increase chlamydia and gonorrhea testing uptake among young adult female emergency department patients. Acad Emerg Med. 2014;21:1512-20.
20. Balfe M, Brugha R. What prompts young adults in Ireland to attend health services for STI testing? BMC Public Health. 2009;9:311.
21. Balfe M, Brugha R. Disclosure of STI testing activities by young adults: the influence of emotions and social networks. Sociol Health Illn. 2010;32:1041-58
22. Balfe M, Brugha R, E OC, McGee H, D OD. Where do young Irish women want Chlamydia-screening services to be set up? A qualitative study employing Goffman's impression management framework. Health Place. 2010;16:16-24.
23. Banikarim C, Chacko MR, Wiemann CM, Smith PB. Gonorrhea and chlamydia screening among young women: stage of change, decisional balance, and self-efficacy. J Adolesc Health. 2003;32:288-95.
24. Barbee L, King I, Dombrowski JC, Golden M. Chlamydia positivity and cost per case detected in asymptomatic women over 25 years in an STD clinic. Sex Transm Dis. 2014;41(Suppl 1):S17.
25. Barral R, Desai R, Gold M, Sucato G, Pletcher J, Bass D, et al. Analysis of human T cell immune responses in adolescents at risk for Chlamydia trachomatis infection. J Adolesc Health. 2011;48:S66-67.
26. Bartelsman M, Straetemans M, Vaughan K, Alba S, van Rooijen MS, Faber WR, et al. Comparison of two Gram stain point-of-care systems for urogenital gonorrhoea among high-risk patients: diagnostic accuracy and cost-effectiveness before and after changing the screening algorithm at an STI clinic in Amsterdam. Sex Transm Infect. 2014;90:358-62.
27. Benner TA. What Could You Do? Interactive video intervention to reduce adolescent females' STI risk. In: Benner TA, Card, J, editors. Model programs for adolescent sexual health: Evidence-based HIV, STI, and pregnancy prevention interventions. New York, NY, US: Springer Publishing Co; 2008. p.227-34.
28. Benner TA. FOCUS: Preventing sexually transmitted infections and unwanted pregnancies among young women. In: Benner TA, Card J, editors. Model programs for adolescent sexual health: Evidence-based HIV, STI, and pregnancy prevention interventions. New York, NY, US: Springer Publishing Co; 2008. p.217-25.
29. Bernstein KT, Chow JM, Ruiz J, Schachter J, Horowitz E, Bunnell R, et al. Chlamydia trachomatis and Neisseria gonorrhoeae infections among men and women entering California prisons. Am J Public Health. 2006;96:1862-6.
30. Bernstein KT, Mehta SD, Rompalo AM, Erbelding EJ. Cost-effectiveness of screening strategies for Gonorrhea among females in private sector care. Obstet Gynecol. 2006;107:813-21.
31. Berry SA, Ghanem KG, Page KR, Thio CL, Moore RD, Gebo KA. Gonorrhoea and chlamydia testing rates of HIV-infected men: low despite guidelines. Sex Transm Infect. 2010;86:481-4.
32. Betournay R, Paparello J, Menon-Johansson A. Real-time partner notification feedback. HIV Med. 2017;18 (Supplement 1):25-6.
33. Blandford JM, Gift TL. Productivity losses attributable to untreated chlamydial infection and associated pelvic inflammatory disease in reproductive-aged women. Sex Transm Dis. 2006;33:S117-21.
34. Bogler T, Farber A, Wijayasinghe S, Glazier R, Guiang C. Effect of the 2012 Ontario cervical screening guidelines on sexually transmitted infection screening. Can Fam Physician. 2015;61(2 Suppl 1):S31.
35. Bolu OO, Lindsey C, Kamb ML, Kent C, Zenilman J, Douglas JM, et al. Is HIV/sexually transmitted disease prevention counseling effective among vulnerable populations? A subset analysis of data collected for a randomized, controlled trial evaluating counseling efficacy (Project RESPECT). Sex Transm Dis. 2004;31:469-74.
36. Booth AR, Norman P, Goyder E, Harris PR, Campbell MJ. Pilot study of a brief intervention based on the theory of planned behaviour and self-identity to increase chlamydia testing among young people living in deprived areas. Br J Health Psychol. 2014;19:636-51.
37. Boudewyns V, Paquin RS. Intentions and beliefs about getting tested for STDs: implications for communication interventions. Health Commun. 2011;26:701-11.
38. Brady M, Baraitser P, Collander-Brown K, Gleisner Z, Barnes D, Pearce V, et al. DIY sexual health care: The user experience. HIV Med. 2010;11(Suppl 1):92.
39. Braun RA, Provost JM. Bridging the gap: using school-based health services to improve chlamydia screening among young women. Am J Public Health. 2010;100:1624-9.
40. Brook G, Burton J, McSorley J, Murphy S. The effectiveness of SMS texts for reminding patients at high risk of STIs and HIV to return for testing. Sex Transm Infect. 2013;89:A139.
41. Brook G, McSorley J, Shaw A. Retrospective study of the effect of enhanced systematic sexually transmitted infection screening, facilitated by the use of electronic patient records, in an HIV-infected cohort. HIV Med. 2013;14:347-53.
42. Brown B, Blas MM, Heidari O, Carcamo C, Halsey NA. Reported changes in sexual behaviour and human papillomavirus knowledge in Peruvian female sex workers following participation in a human papillomavirus vaccine trial. Int J STD AIDS. 2013;24:531-5.
43. Brunahl CA, Riegel B, Hoink J, Kutup A, Eichelberg E, Lowe B. Psychosomatic aspects of chronic pelvic pain syndrome. Psychometric results from the pilot phase of an interdisciplinary outpatient clinic. Schmerz. 2014;28:311-8.
44. Buhrer-Skinner M, Muller R, Bialasiewicz S, Sloots TP, Debattista J, Gordon R, et al. The check is in the mail: Piloting a novel approach to Chlamydia trachomatis testing using self-collected, mailed specimen. Sex Health. 2009;6:163-9.
45. Burchell AN, Allen V, Grewal R, Moravan V, Remis RS, Gardner S, et al. Gonorrhea co-infection among gay and bisexual men in HIV care in Ontario : Trends in testing and diagnosis, 2008-2011. Can J Infect Dis Med Microbiol. 2013;24(Suppl A):60A-1A.
46. Carey MP, Senn TE, Vanable PA, Coury-Doniger P, Urban MA. Brief and intensive behavioral interventions to promote sexual risk reduction among STD clinic patients: results from a randomized controlled trial. Aids Behav. 2010;14:504-17.
47. Carty JN, Tomakowsky J, Lumley MA, Carrico DJ, Peters K. Social constraints and emotional processing as unique correlates of health status in women with chronic pelvic pain. Psychosom Med. 2014;76:A111.
48. Catarino R, Cherkaoui A, Trellu LT, Yaron M. Chlamydia trachomatis screening in Switzerland: The experience of the Geneva University Hospitals. Eur J Contracept Reprod Health Care. 2018;23(Suppl 1):139-40.
49. Caul EO, Horner PJ, Leece J, Crowley T, Paul I, Davey-Smith G. Population-based screening programmes for Chlamydia trachomatis. Lancet. 1997;349:1070-1.
50. Cavalcante EG, Miranda MC, Carvalho AZ, Lima IC, Galvao MT. Partner notification for sexually transmitted infections and perception of notified partners. Rev Esc Enferm USP. 2016;50:450-7.
51. Celentano DD, Dilorio C, Hartwell T, Kelly J, Magana R, Maibach E, et al. The NIMH multisite HIV prevention trial: Reducing HIV sexual risk behavior. Science. 1998;280:1889-94.
52. Celentano DD, Mayer KH, Pequegnat W, Abdala N, Green AM, Handsfield HH, et al. Prevalence of sexually transmitted diseases and risk behaviors from the NIMH Collaborative HIV/STD Prevention Trial. Int J Sex Health. 2010;22:272-84.
53. Chacko M, Markham C, Crandall S, Thiel M, Torres J. Can adolescents participating in a school-based research project receive STI treatment in off-campus and non-clinic settings? J Adolesc Health. 2011;48:S60-S1.
54. Champion JD. Behavioural interventions and abuse: secondary analysis of reinfection in minority women. Int J STD AIDS. 2007;18:748-53.
55. Chen MY, Karvelas M, Sundararajan V, Hocking JS, Fairley CK. Evidence for the effectiveness of a chlamydia awareness campaign: increased population rates of chlamydia testing and detection. Int J STD AIDS. 2007;18:239-43.
56. Chiao C, Morisky DE, Ksobiech K, Masson CL, Malow RM. Clinic appointment attendance for sexually transmitted infection screening among Filipina sex workers: a multilevel analysis. AIDS Care. 2007;19:1166-70.
57. Clark J, Chavez-Gomez S, Castaneda-Huerta A, Passaro R, Gonzales-Saavedra W, Cachay E, et al. Personalized cognitive counseling (PCC) to reduce HIV risk following rectal gonorrhea/ chlamydia diagnosis among MSM in PERU. Sex Transm Infect. 2019;95:A254.
58. Clarke J, White K, Turner K. Efficacy of chlamydia control: Optimal combinations of partner notification and screening in a pair approximation model. Int J STD AIDS. 2013;24(Suppl 1):16.
59. Cohen AC. Evaluating an online patient engagement platform and smartphone application that notifies clients of sexually transmitted infection test results. PhD [dissertation]. Los Angeles: University of California; 2017. Available in: Dissertation Abstracts International: Section B: The Sciences and Engineering.
60. Cohen S, Vittinghoff E, Philip SS, Doblecki-Lewis S, Bacon O, Chege W, et al. Quarterly STI screening optimizes STI detection among prep users in the demo project. Top Antivir Med. 2016;24:368-9.
61. Coll P, Andreu A, Jane M, Meulbroek M, Cruz I, Mansilla R, et al. Prevalence study of asymptomatic sexually transmitted infection among men who have sex with men in a non-clinical setting in Barcelona. Int J STD AIDS. 2015;26:71.
62. Coppus SFPJ, Land JA, Opmeer BC, Steures P, Eijkemans MJC, Hompes PGA, et al. Chlamydia trachomatis IgG seropositivity is associated with lower natural conception rates in ovulatory subfertile women without tubal pathology. Hum Reprod. 2009;24:i100-1.
63. Cunha-Oliveira A, Cunha-Olivera J, Cardoso S, Pita J. Behavioral interventions for prevention of sexually transmitted infections in university students over 36 months. Atencion Primaria. 2014;46(Suppl 5):28.
64. Cunningham SD. The social context and public health implications of stigma associated with sexually transmitted infections. PhD [dissertation]. Maryland: Johns Hopkins University; 2007. Available from: Dissertation Abstracts International: Section B: The Sciences and Engineering.
     Cunningham SD, Kerrigan DL, Jennings JM, Ellen JM. Relationships between perceived STD-related stigma, STD-related shame and STD screening among a household sample of adolescents. Perspect Sex Reprod Health. 2009;41:225-30.
65. Currie S, Mercer C, Dunbar K, Saunders J, Woodhall S. Young adults' chlamydia testing patterns and awareness of guidance: Results from a clinic-based survey. HIV Med. 2018;19(Suppl 2):S66.
66. Dakshina S, Cowdy C, Dave J, Sampson M, Sarner L, Tong W. Confirmatory tests for oropharyngeal gonorrhoea in GUM clinics. HIV Med. 2014;15(Suppl 3):104-5.
67. Dave S, Kerry SR, Oakeshott P, McGregor F, Cannon E, Stephenson JM. Women's health study of Mycoplasma genitalium: A feasibility study. Sex Transm Infect. 2012;88(Suppl 1):A23.
68. Davies B, Day S, Ward H. Estimating the incidence of PID following chlamydia infection in sex workers. Sex Transm Infect. 2011;87:A158-9.
69. Davies B, Turner KME, Benfield T, Frolund M, Andersen B, Westh H, et al. Pelvic inflammatory disease risk following negative results from chlamydia nucleic acid amplification tests (NAATs) versus non-NAATs in Denmark: A retrospective cohort. PLoS Med. 2018;15:E1002483.
70. Davison R, Bartholemew S, Clutterbuck DJ. Audit of Postal Test Kits in Edinburgh for Chlamydia trachomatis as an alternative to genitourinary medicine clinic attendance. Int J STD AIDS. 2007;18:349-50.
71. De Menezes CHAB, Botto-Menezes C, Benzaken NS, De Fatima Santana Jardim L, Jardam L, Das Neves DBS, et al. Prevalence of chlamydia trachomatis infection in women aged 14-25 years: A simulation based screening program in the Brazilian Amazon region. Sex Transm Dis. 2014;41(Suppl 1):S142.
72. De Olalla PG, Gorrindo P, Gil S, Simon P, Masdeu E, Santoma MJ, et al. A need to improve partner notification in sexually transmitted infections in Barcelona. Int J STD AIDS. 2015;26:82.
73. de Vries R, van Bergen JE, de Jong-van den Berg LT, Postma MJ, PILOT-CT Study Group. Systematic screening for Chlamydia trachomatis: estimating cost-effectiveness using dynamic modeling and Dutch data. Value Health. 2006;9:1-11.
74. Dearing N, Pammi M. Management of symptomatic men in a level 2 sexual health service. Sex Transm Infect. 2012;88(Suppl 1):A54
75. Decker MR, Miller E, McCauley HL, Tancredi DJ, Levenson RR, Waldman J, et al. Intimate partner violence and partner notification of sexually transmitted infections among adolescent and young adult family planning clinic patients. Int J STD AIDS. 2011;22:345-7.
76. Denison HJ, Bromhead C, Grainger R, Dennison EM, Jutel A. What influences university students to seek sexually transmitted infection testing?: A qualitative study in New Zealand. Sex Reprod Healthc. 2018;16:56-60.
77. Desai M, Burns F, Gilson R, Nardone A, Mercey D. Facilitators and barriers to active recall for HIV and STI testing of MSM: A mixed methods study. HIV Med. 2016;17(Suppl 1):56-7.
78. Dharmaratne SD, Buddhakarale K. Concomitant sexually transmitted diseases in patients with newly diagnosed HIV in Sri Lanka. Retrovirology. 2012;9(Suppl 1):84.
79. DiClemente RJ, Wingood GM, Harrington KF, Lang DL, Davies SL, Hook EW, 3rd, et al. Efficacy of an HIV prevention intervention for African American adolescent girls: a randomized controlled trial. JAMA. 2004;292:171-9.
80. Dillow MR, Labelle S. Discussions of sexual health testing: Applying the theory of motivated information management. Pers Relatsh. 2014;21:676-91.
81. DiVasta AD, Vernacchio L, Francis ME, Focht G, Jooma F, Forman SF. A quality improvement program to increase chlamydia screening for adolescent and young adult women. J Adolesc Health. 2015;56(Suppl 1):S77.
82. Domes T, Lo K, Grober E, Mullen B, Mazzulli T, Jarvi K. The utility and cost of routine Chlamydia trachomatis and Neisseria gonorrhoeae testing in an asymptomatic male infertility population. J Urol. 2011;185:E874.
83. Donaldson A, Maehr J, Ellen J. Case manager facilitated screening for Chlamydia and gonorrhea among community-supervised Juvenile justice-involved adolescent females. J Adolesc Health. 2011;48:S61-2.
84. Downs JS, Ashcraft AM, Murray PJ. Can you get an STI from a virgin? The answer might surprise you intervention improves knowledge about sexual risk perceptions. J Adolesc Health. 2015;56(Suppl 1):S76-7.
85. Dudareva-Vizule S, Jansen K, Haar K, Sailer A, Hofmann A, Hamouda O, et al. Chlamydia trachomatis infection in women in Germany, 2008-2014. Sex Transm Infect. 2015;91:A146.
86. Dudareva-Vizule S, Sailer A, Hamouda O, Bremer V. Positivity rate of chlamydia trachomatis and status quo of opportunistic screening in Germany. Sex Transm Infect. 2013;89:A156-7.
87. Dudareva-Vizule S, Sailer A, Hamouda O, Bremer V. Results of chlamydia trachomatis laboratory sentinel in Germany. Int J Med Microbiol. 2013;303(Suppl 1):79.
88. Dukers-Muijrers NH, Theunissen KA, Wolffs PT, Kok G, Hoebe CJ. Acceptance of Home-Based Chlamydia Genital and Anorectal Testing Using Short Message Service (SMS) in Previously Tested Young People and Their Social and Sexual Networks. PLoS ONE. 2015;10:E0133575.
89. Duncan B, Hart G, Scoular A, Bigrigg A. Qualitative analysis of psychosocial impact of diagnosis of chlamydia trachomatis: Implications for screening. BMJ. 2001;322:195-9.
90. Dykstra C, Fatvich D, Flexman J, Phillips M, McCloskey J. Royal perth hospital emergency department screening project for chlamydia trachomatis. Sex Transm Infect. 2011;87:A347.
91. Eadsforth H, Southon L, Gray L, Thomas D, McQuillan O. Sexual health outreach work within prisons-treating a captive audience? Int J STD AIDS. 2013;24(Suppl 5):32-3.
92. Edmiston N, Ooi C, Merrit T. Make contact: A comparitive study of contact tracing strategies for chlamydia. Sex Health. 2009;6:354.
93. Eggman AA, Feaster DJ, Leff JA, Golden MR, Castellon PC, Gooden L, et al. The cost of implementing rapid HIV testing in sexually transmitted disease clinics in the United States. Sex Transm Dis. 2014;41:545-50.
94. Emmerton L, Buhrer Skinner M, Gardiner E, Nissen L, Debattista J. A trial of the distribution of chlamydia self-collection postal specimen kits from Australian community pharmacies. Sex Health. 2011;8:130-2.
95. Estcourt CS, Gibbs J, Sutcliffe LJ, Gkatzidou V, Tickle L, Hone K, et al. Is an automated online clinical care pathway for people with genital chlamydia (chlamydiaoccp) within an esexual health clinic feasible and acceptable? Proof of concept study. Sex Transm Infect. 2015;91:A55.
96. Estcourt CS, Gibbs J, Sutcliffe LJ, Gkatzidou V, Tickle L, Hone K, et al. The eSexual Health Clinic system for management, prevention, and control of sexually transmitted infections: exploratory studies in people testing for Chlamydia trachomatis. Lancet Public Health. 2017;2:E182-90.
97. Fenton KA, Korovessis C, Johnson AM, McCadden A, McManus S, Wellings K, et al. Sexual behaviour in Britain: Reported sexually transmitted infections and prevalent genital Chlamydia trachomatis infection. Lancet. 2001;358:1851-4.
98. Filardo T, Hussein A, Frasca K, McCollister B, Madinger N. Evidence-based care for sexually transmitted infections: Missed opportunities in an academic medical center. Sex Transm Dis. 2018;45 (Suppl 2):S92.
99. Fine D, Salomon S, Bowen V, Hughes G. Measuring program effectiveness: Screening coverage, treatment, and partner services. Sex Transm Dis. 2014;41:S34-5.
100. Fine D, Thomas KK, Nakatsukasa-Ono W, Marrazzo J. Chlamydia positivity in women screened in family planning clinics: Racial/ethnic differences and trends in the Northwest U.S., 1997-2006. Public Health Rep. 2012;127:38-51.
101. Fisher M, Wayal S, Smith H, Llewellyn C, Alexander S, Ison C, et al. Home sampling for sexually transmitted infections and HIV in men who have sex with men: a prospective observational study. PLoS ONE. 2015;10:E0120810.
102. Forbes G, Clutterbuck DJ. How many cases of chlamydial infection would we miss by not testing partners for infection? Int J STD AIDS. 2009;20:267-8.
103. Ford CA, Jaccard J, Millstein SG, Bardsley PE, Miller WC. Perceived risk of chlamydial and gonococcal infection among sexually experienced young adults in the United States. Perspect Sex Reprod Health. 2004;36:258-64.
104. Foster S, Womack V, Ainslie S, Folkard KA, Dunbar K, Saunders J. Securing excellence in chlamydia screening outcomes on a shrinking budget. Sex Transm Infect. 2015;91(Suppl 1):A7-8.
105. Franklin N, Guy R, Grulich A, Fairley CK, Chen MY, Hellard M, et al. Chlamydia testing and prevalence at australian sexual health clinics; the Australian collaboration for chlamydia enhanced sentinal surveillance (ACCESS) project. Sex Health. 2009;6:366-7.
106. Free C, Swinson R, Potter K, McCarthy O, Knight R, Baraitser P, et al. Safetxt: a randomised controlled trial of an intervention delivered by mobile phone messaging designed to reduce infection with chlamydia and gonorrhoea-recruitment methods. Sex Transm Infect. 2017;93:A88‐.
107. Friedman A, Bloodgood B, Bender J, Levine E. Can the prospect of infertility motivate young women's intentions to seek preventive healthcare? Findings from CDC concept testing focus groups. Contraception. 2010;82:209.
108. Friedman AL, Bloodgood B. "Something we'd rather not talk about": findings from CDC exploratory research on sexually transmitted disease communication with girls and women. J Womens Health (Larchmt). 2010;19:1823-31.
109. Friedman AL, Bloodgood B. Exploring the feasibility of alternative STD-testing venues and results delivery channels for a national screening campaign. Health Promot Pract. 2013;14:96-104.
110. Fuller SS, Mercer CH, Copas AJ, Saunders J, Sutcliffe LJ, Cassell JA, et al. The SPORTSMART study: a pilot randomised controlled trial of sexually transmitted infection screening interventions targeting men in football club settings. Sex Transm Infect. 2015;91:106-10.
111. Fuller SS, Mercer CH, Copas AJ, Saunders J, Sutcliffe LJ, Cassell JA, et al. Two urine-based sexually transmitted infection screening interventions targeting young men in football club settings (SPORTSMART): a pilot randomised controlled trial. Lancet. 2013;382:S38.
112. G B, Doolub R, Noden J. An audit on the rates of partner notification and verification in cases of Chlamydia. International Journal of STD and AIDS. 2013;24(Suppl 1):16.
113. Galarraga O, Sosa-Rubi SG, Infante C, Gertler PJ, Bertozzi SM. Willingness-to-accept reductions in HIV risks: Conditional economic incentives in Mexico. Eur J Health Econ. 2014;15:41-55.
114. Garcia DE. Diffusing the innovation of e-health featuring avatar videos designed to empower men who have sex with men to increase HIV testing, screening for sexually transmitted infections, and risk reduction behaviors. PhD [dissertation]. New York: Columbia University; 2014. Available from: Dissertation Abstracts International Section A: Humanities and Social Sciences.
115. Gardhouse CE, Levett PN, Horsman GB. Epidemiology, co-infection and laboratory testing of chlamydia trachomatis and neisseria gonorrhoeae in Saskatchewan. Can J Infect Dis Med Microbiol. 2012;23(Suppl B):19B.
116. Garrett N, Nori A, Lynch J, Heran H, Sarner L. Something for the weekend! Piloting a Saturday morning GUM service. HIV Med. 2010;11(Suppl 1):102.
117. Garside R, Ayres R, Owen M, Pearson VAH, Roizen J. 'They never tell you about the consequences': Young people's awareness of sexually transmitted infections. Int J STD AIDS. 2001;12:582-8.
118. Gaydos C, Hsieh YH, Barnes M, Jett-Goheen M, Quinn N, Agreda P, et al. Characteristics and predictors of women seeking rescreening for stis after using the http://www.iwantthekit.org program: Were they infected or uninfected? Sex Transm Infect. 2011;87(Suppl 1):A201-A2.
119. Gaydos C, Hsieh YH, Barnes M, Quinn N, Agreda P, Whittle P, et al. Risk for trichomonas vaginalis infections in internet-recruited females who submit self-collected vaginal swabs. J Adolesc Health. 2011;48:S13.
120. Gaydos C, Lewis M, Michele-Corinne AKO, Rothman R, Dugas A. Use of rapid diagnostics for chlamydia and gonorrhoea for women in the emergency department can improve clinical management: report of a randomised clinical trial. Sexually transmitted infections Conference: 2017 STI and HIV world congress Brazil. 2017;93(Suppl 2):A107.
121. Gaydos CA, Ako MC, Lewis M, Hsieh YH, Rothman RE, Dugas AF. Use of a Rapid Diagnostic for Chlamydia trachomatis and Neisseria gonorrhoeae for Women in the Emergency Department Can Improve Clinical Management: report of a Randomized Clinical Trial. Ann Emerg Med. 2019;74:36-44.
122. Gaydos CA, Barnes M, Aumakhan B, Quinn N, Agreda P, Whittle P, et al. Males will submit self-obtained penile swabs for the detection of chlamydia trachomatis when recruited via the internet: Acceptability and accuracy. J Adolesc Health. 2009;44:S9.
123. Gaydos CA, Hsieh YH, Galbraith JS, Barnes M, Waterfield G, Stanton B. Focus-on-Teens, sexual risk-reduction intervention for high-school adolescents: impact on knowledge, change of risk-behaviours, and prevalence of sexually transmitted diseases. Int J STD AIDS. 2008;19:704-10.
124. Gaydos CA, Rizzo-Price PA, Balakrishnan P, Mateta P, Leon SR, Verevochkin S, et al. Impact of international laboratory partnerships on the performance of HIV/sexually transmitted infection testing in five resource-constrained countries. Int J STD AIDS. 2011;22:645-52.
125. Gaydos CA, Rizzo-Price PA, Barnes M, Dwyer K, Wood BJ, Hogan MT. The use of focus groups to design an internet-based program for chlamydia screening with self-administered vaginal swabs: what women want. Sex Health. 2006;3:209-15.
126. Ghazal-Aswad S, Badrinath P, Osman N, Abdul-Khaliq S, Mc Ilvenny S, Sidky I. Prevalence of Chlamydia trachomatis infection among women in a Middle Eastern community. BMC Womens Health. 2004;4:3.
127. Gilbert M, Hottes TS, Kerr T, Taylor D, Fairley CK, Lester R, et al. Factors associated with intention to use internet-based testing for sexually transmitted infections among men who have sex with men. J Med Internet Res. 2013;15:E254.
128. Gilbert M, Thomson K, Salway T, Haag D, Grennan T, Fairley CK, et al. Differences in experiences of barriers to STI testing between clients of the internet-based diagnostic testing service GetCheckedOnline.com and an STI clinic in Vancouver, Canada. Sex Transm Infect. 2019;95:151-6.
     Gillespie P, O'Neill C, Adams E, Turner K, O'Donovan D, Brugha R, et al. The cost and cost-effectiveness of opportunistic screening for Chlamydia trachomatis in Ireland. Sex Transm Infect. 2012;88:222-8.
129. Goddard SL, Rajagopal P, Templeton DJ. Increasing yield of pharyngeal Chlamydia trachomatis among male gay and bisexual clinic attendees in Sydney: An observational study. Sex Health. 2017;14:282-5.
130. Goyal M, Fein J, Badolato G, Shea J, Trent M, Teach S, et al. Clinical decision support using computerized sexual health surveys improves STI screening rates among high-risk adolescents in a pediatric emergency department: A randomized trial. Pediatrics Conference: National Conference on Education. 2016;141.
131. Graseck A, Secura G, Allsworth J, Peipert J. Home vs. clinic-based screening for sexually transmitted infections: Patient preference and completion rates. Contraception. 2009;80:199.
     Grentzer JM, Peipert, J.F., Zhao, Q., McNicholas, C., Secura, G., Madden, T. Risk-based screening for chlamydia and gonorrhea prior to intrauterine device insertion misses few cases. J Midwifery Womens Health. 2016;61:126-.
132. Group NCHSPT. Methodological overview of a five-country community-level HIV/sexually transmitted disease prevention trial. Aids. 2007;21(Suppl 2):S3-18.
133. Group NCHSPT. Results of the NIMH collaborative HIV/sexually transmitted disease prevention trial of a community popular opinion leader intervention. J Acquir Immune Defic Syndr. 2010;54:204-14.
134. Gudka S, Misaghian J, Clifford RM. Future pharmacy-based chlamydia screening: Consumer preference. J Pharm Pract Res. 2013;43:15-8.
135. Guy RJ, Ward J, Causer LM, Natoli L, Badman SG, Tangey A, et al. Molecular point-of-care testing for chlamydia and gonorrhoea in Indigenous Australians attending remote primary health services (TTANGO): a cluster-randomised, controlled, crossover trial. The lancet Infectious diseases. 2018;18:1117‐26.
136. Habel MA, Leichliter JS, Torrone E. Exploring chlamydia positivity among females on college campuses, 2008-2010. J Am Coll Health. 2016;64:496-501.
137. Haidari G, Perry M, White JA. Are we seeing a true rise in Neisseria gonorrhoeae and Chlamydia trachomatis in men who have sex with men? Int J STD AIDS. 2013;24(Suppl 1):1.
138. Han JS, Rogers ME, Nurani S, Rubin S, Blank S. Patterns of Chlamydia/Gonorrhea positivity among voluntarily screened New York City public high school Students. J Adolesc Health. 2011;49:252-7.
139. Han Y, Coles FB, Muse A, Hipp S. Assessment of a geographically targeted field intervention on gonorrhea incidence in two New York State counties. Sex Transm Dis. 1999;26:296-302.
140. Hawkins NA, Benard VB, Greek A, Roland KB, Manninen D, Saraiya M. Patient knowledge and beliefs as barriers to extending cervical cancer screening intervals in Federally Qualified Health Centers. Prev Med. 2013;57:641-5.
141. Hazlina NHN, Zuky NA, Johari Md R, Senik N. A study on common sexually transmitted disease infection in infertile female attending to Infertility Clinic in HUSM, Kota Bharu, Kelantan. Int Med J. 2005;12:205-11.
142. Heal C, Jones B, Veitch C, Lamb S, Hodgens S, Browning S, et al. Screening for chlamydia in general practice. Aust Fam Physician. 2002;31:779-82.
143. Hengel B, Guy R, Garton L, Ward J, Rumbold A, Taylor-Thomson D, et al. Barriers and facilitators of sexually transmissible infection testing in remote Australian Aboriginal communities: results from the Sexually Transmitted Infections in Remote Communities, Improved and Enhanced Primary Health Care (STRIVE) Study. Sex Health. 2015;12:4-12.
144. Henry Ford Health System. Emergency Department, Rapid Assessment for Sexually Transmitted Infection (ED-RASTI). 2015. ClinicalTrials.gov registration number: NCT02386514.
145. Hernandez I, Adegoke K, Rosas C, Ochoa T, Reina M, Sharma V, et al. Prevalence of STI/HIV and factors associated with STI among female sex workers in Ecuador. Trop Med Int Health. 2015;20(Suppl 1):285.
146. Herzog SA, Althaus CL, Heijne JC, Oakeshott P, Kerry S, Hay P, et al. Timing of progression from Chlamydia trachomatis infection to pelvic inflammatory disease: a mathematical modelling study. BMC Infect Dis. 2012;12:187.
147. Hill-Tout R, Prime K. Are cases of gonorrhoea rising in very young patients in south west london? A retrospective case review of patients aged 18 years and younger diagnosed with gonorrhoea in a London teaching hospital GUM service. Sex Transm Infect. 2015;91(Suppl 1):A92.
148. Hocking J, Poznanski S, Vaisey A, Walker J, Wood A, Lewis D, et al. A multifaceted intervention to increase chlamydia testing in australian general practice. Sex Transm Infect. 2011;87(Suppl 1):A199.
149. Hocking JS, Spark S, Guy R, Temple-Smith M, Fairley CK, Kaldor J, et al. The Australian chlamydia control effectiveness pilot (ACCEPt): First results from a randomised trial of annual chlamydia screening in general practice. Sex Transm Infect. 2012;88(Suppl 1):A3-4.
150. Hoebe CJ, Brouwers EE, Van Bergen JE, Fennema JS, Gotz HM, Koekenbier RH, et al. Systematic selection of screening participants by risk score in chlamydia screening programme is feasible and effective. Sex Transm Infect. 2011;87(Suppl 1):A322-3.
151. Hoenderboom BM, van Oeffelen AA, van Benthem BH, van Bergen JE, Dukers-Muijrers NH, Gotz HM, et al. The Netherlands Chlamydia cohort study (NECCST) protocol to assess the risk of late complications following Chlamydia trachomatis infection in women. BMC Infect Dis. 2017;17:264.
152. Holgate HS, Longman C. Some peoples' psychological experiences of attending a sexual health clinic and having a sexually transmitted infection. J R Soc Health. 1998;118:94-6.
153. Horner P, Loaring J, Matthew H, Oliver I, Campbell R, Trotter C, et al. Could a peer driven intervention increase uptake of chlamydia screening? Proof of principle. Sex Transm Infect. 2011;87(Suppl 1):A207.
154. Hottes TS, Farrell J, Bondyra M, Haag D, Shoveller J, Gilbert M. Internet-based HIV and sexually transmitted infection testing in British Columbia, Canada: opinions and expectations of prospective clients. J Med Internet Res. 2012;14:E41.
155. Howard H, Barandas A, Creegan L, Bauer H, Chow J, Park I, et al. Developing a multi-pronged quality improvement (QI) strategy to increase chlamydia trachomatis (CT) retesting rates: Building a framework for success. Sex Transm Infect. 2011;87(Suppl 1):A320.
156. Hsieh YH, Barnes M, Jett-Goheen M, Quinn N, Agreda P, Whittle P, et al. Characteristics of men who seek rescreening for STIs after once using the http://www.iwantthekit.org screening program. Sex Transm Infect. 2011;87(Suppl 1):A203.
157. Imrie J, Stephenson JM, Cowan FM, Wanigaratne S, Billington AJ, Copas AJ, et al. A cognitive behavioural intervention to reduce sexually transmitted infections among gay men: randomised trial. BMJ. 2001;322:1451-6.
158. Isola M, Francis J. Use of expedited partner therapy for treatment of chlamydia in adolescents. J Pediatr Adolesc Gynecol. 2018;31:178.
159. ISRCTN. A randomised controlled study of mouth swab testing versus same-day blood tests for human immunodeficiency virus (HIV) infection in young people attending a young person's community drug service. <http://www.isrctn.com/ISRCTN38526137>. .
160. ISRCTN. Using the theory of planned behaviour to increase chlamydia testing in young people. <http://www.isrctn.com/ISRCTN82617454>.
161. SRCTN. “Test n Treat”: a feasibility trial of rapid STI testing and treatment. <http://www.isrctn.com/ISRCTN58038795>
162. ISRCTN. A randomised controlled trial of a safer sex intervention delivered through mobile phone messaging. <http://www.isrctn.com/ISRCTN64390461>
163. Ivanova T, Anisimova N, Guschin A, Simonova E. The pattern of STI burden in the central region of Russia: Chlamydiosis vs trichomoniasis. Which one is the leader? Sex Transm Infect. 2011;87(Suppl 1):A101.
164. Ivaz S, Brennan S, Dean S, Hay S, Hay P, Kerry S, et al. Lessons learned from recruiting young female students to a randomised controlled trial of chlamydia screening. Fam Pract. 2006;23:188-91.
165. Jackson LJ, Roberts TE, Fuller SS, Sutcliffe LJ, Saunders JM, Copas AJ, et al. Exploring the costs and outcomes of sexually transmitted infection (STI) screening interventions targeting men in football club settings: preliminary cost-consequence analysis of the SPORTSMART pilot randomised controlled trial. Sex Transm Infect. 2015;91:100-5.
166. Jemmott LS, Jemmott JB 3rd, O'Leary A. Effects on sexual risk behavior and STD rate of brief HIV/STD prevention interventions for African American women in primary care settings. Am J Public Health. 2007;97:1034-40.
167. Jenkins WD, Weis R, Campbell P, Barnes M, Barnes P, Gaydos C. Comparative effectiveness of two self-collected sample kit distribution systems for chlamydia screening on a university campus. Sex Transm Infect. 2012;88:363-7.
168. Jenkins WD, Zahnd W, Kovach R, Kissinger P. Chlamydia and gonorrhea screening in United States emergency departments. J Emerg Med. 2013;44:558-67.
169. Jeong S, Cha C, Lee J. The effects of STI education on Korean adolescents using smartphone applications. Health Educ J. 2017;76:775-86.
170. John SA, Walsh JL, Cho YI, Weinhardt LS. Perceived Risk of Intimate Partner Violence Among STI Clinic Patients: Implications for Partner Notification and Patient-Delivered Partner Therapy. Arch Sex Behav. 2018;47:481-92.
171. Johns Hopkins Bloomberg School of Public Health. Trial of an Adapted STD Screening and Risk Reduction Intervention. 2015. ClinicalTrials.gov registration number: NCT02513225.
172. Johnson A, Tucker C. Youth-led participatory evaluation of a community engagement project designed to lower rates of sexually transmitted infections among young women and transgender women of color. Sex Transm Dis. 2016;43(Suppl 2):S195.
173. Johnson K, Gilbert L, Hunt T, Wu E, Metsch L, Goddard-Eckrich D, et al. The effectiveness of a group-based computerized HIV/STI prevention intervention for black women who use drugs in the criminal justice system: study protocol for E-WORTH (Empowering African-American Women on the Road to Health), a Hybrid Type 1 randomized controlled trial. Trials. 2018;19:486.
174. Johnson-Mallard V. The effects of an education/behavioral intervention on knowledge, perceived risk and self-efficacy for sexually transmitted infections in women. PhD [dissertation]. Florida: University of South Florida; 2005. <https://scholarcommons.usf.edu/etd/2944>. Accessed 10 Sept 2020.
175. Jolly A. Evolution of sexual networks over time in Manitoba, Canada. Sex Transm Infect. 2011;87(Suppl 1):A191-2.
176. Jones HE, Holloway IW, Pressman E, Meier J, Westhoff CL. Women's preferences for testing and management of sexually transmitted infections among low-income New York City family planning clients. Int J STD AIDS. 2013;24:455-60.
177. Jones LF, Ricketts E, Town K, Rugman C, Lecky D, Folkard K, et al. Chlamydia and HIV testing, contraception advice, and free condoms offered in general practice: a qualitative interview study of young adults' perceptions of this initiative. Br J Gen Pract. 2017;67:E490-500.
178. Jordan N, Clemmons N, Gaydos J, Fishman J, Jacobsmuhlen T, Choon Lee H, et al. Chlamydia trachomatis screening initiative among female us army soldiers deployed to Korea. Sex Transm Infect. 2011;87(Suppl 1):A204.
179. Jordan NN, Clemmons NS, Gaydos JC, Lee HC, Yi SH, Klein TA. Chlamydia trachomatis screening initiative among U.S. Army soldiers assigned to Korea. MSMR. 2013;20:15-6.
180. Jumping-Eagle S, Sheeder J, Kelly LS, Stevens-Simon C. Feasibility and utility of screening adolescent mothers for Chlamydia at their children's health care visits. Matern Child Health J. 2007;11:586-94.
181. Kang M, Rochford A, Johnston V, Jackson J, Freedman E, Brown K, et al. Prevalence of Chlamydia trachomatis infection among 'high risk' young people in New South Wales. Sex Health. 2006;3:253-4.
182. Kang M, Rochford A, Skinner R, Mindel A, Webb M, Peat J, et al. Facilitating chlamydia testing among young people: a randomised controlled trial in cyberspace. Sex Transm Infect. 2012;88:568-73.
183. Kang M, Rochford A, Skinner R, Mindel A, Webb M, Usherwood T. Getting clued up in cyberspace about chlamydia: Arandomised controlled trialof an internet-based intervention. J Adolesc Health. 2009;44:S22.
184. Karamouzian M, Shoveller J, Dong H, Gilbert M, Kerr T, DeBeck K. Perceived Devaluation and STI Testing Uptake among a Cohort of Street-Involved Youth in a Canadian Setting. Arch Sex Behav. 2017;46:2165-72.
185. Katz DA, Golden MR, Hughes JP, Farquhar C, Stekler JD. HIV Self-testing increases HIV testing frequency in high-risk men who have sex with men: a randomized controlled trial. Journal of acquired immune deficiency syndromes. 2018;78:505‐12.
186. Kayaert G, Van Den Eynde SA. Are Belgian students being tested for sexually transmitted infections, and why (Not)? Sex Transm Infect. 2013;89(Suppl 1):A315.
187. Kearley-Shiers K. Partner notification resolution: Why is it overlooked in integrated community clinics? HIV Med. 2018;19(Suppl 2):S55-6.
188. Keefe A. Pubic lice and scabies: an opportunity for STI screening. Nurs Prescr. 2012;10:494-7.
189. Kerry-Barnard S, Fleming C, Reid F, Phillips R, Drennan VM, Adams EJ, et al. 'Test n Treat (TnT)'- Rapid testing and same-day, on-site treatment to reduce rates of chlamydia in sexually active further education college students: study protocol for a cluster randomised feasibility trial. Trials. 2018;19.
190. Kerry-Barnard S, Fleming C, Reid F, Phillips R, Drennan VM, Adams EJ, et al. 'Test n Treat (TnT)'- Rapid testing and same-day, on-site treatment to reduce rates of chlamydia in sexually active further education college students: study protocol for a cluster randomised feasibility trial. Trials. 2018;19:311.
191. Kersaudy-Rahib D, de Barbeyrac B, de Diego S, Le Roy C, Bebear C, Lydie N. Home screening compared with clinic-based screening for Chlamydiae trachomatis in France: a randomised controlled trial. Lancet. 2013;382:53.
192. Kirkwood K, Horn K, Glasier A, Sutherland S, Young H, Patrizio C. Non-invasive screening of teenagers for chlamydia trachomatis in a family planning setting. Br J Fam Plann. 1999;25:11-2.
193. Klomp JM, Boon ME, Dorman MZ, van Haaften M, Heintz AP. Trends in inflammatory status of the vaginal flora as established in the Dutch national screening program for cervical cancer over the last decade. Acta Cytol. 2010;54:43-9.
194. Knight R, Falasinnu T, Oliffe JL, Gilbert M, Small W, Goldenberg S, et al. Integrating gender and sex to unpack trends in sexually transmitted infection surveillance data in British Columbia, Canada: an ethno-epidemiological study. BMJ Open. 2016;6:E011209.
195. Kohler PK, Campos PE, Garcia PJ, Carcamo CP, Buendia C, Hughes JP, et al. Sexually transmitted infection screening uptake and knowledge of sexually transmitted infection symptoms among female sex workers participating in a community randomised trial in Peru. Int J STD AIDS. 2016;27:402-10.
196. Kohn R, McCright J, Ivory B, Snell A. Chlamyidia partner notification for adolescent females, San Francisco, 2010. Sex Transm Infect. 2011;87(Suppl 1):A217.
197. Kong FY, Hocking JS, Link CK, Chen MY, Hellard ME. Sex and sport: chlamydia screening in rural sporting clubs. BMC Infect Dis. 2009;9:73.
198. Kouyoumdjian FG, Main C, Calzavara LM, Kiefer L. Prevalence and predictors of urethral chlamydia and gonorrhea infection in male inmates in an Ontario correctional facility. Can J Public Health. 2011;102:220-4.
199. Kraft JM, Whiteman MK, Carter MW, Snead MC, DiClemente RJ, Murray CC, et al. Identifying psychosocial and social correlates of sexually transmitted diseases among black female teenagers. Sex Transm Dis. 2015;42:192-7.
200. La France DR, Rambin ED, Matthews-Greer J. Screening of males for chlamydia trachomatis and neisseria gonorrhoeae in an emergency room setting. Am J Clin Pathol. 2010;134:677.
201. La Montagne DS, Patrick LE, Fine DN, Marrazzo JM, Region XIPP. Re-evaluating selective screening criteria for chlamydial infection among women in the U S Pacific Northwest. Sex Transm Dis. 2004;31:283-9.
202. Labeja Acellam ET, Harris VG. A study to evaluate Chlamydia screening in colposcopy clinics. J Obstet Gynaecol. 1997;17:472-5.
203. Lachowsky NJ, Stephenson K, Cui Z, Shurgold S, Rich A, Grennan T, et al. Incident syphilis, gonorrhea, and chlamydia infection among a cohort of MSM. Top Antivir Med. 2016;24:434.
204. Lally MA, Alvarez S, Macnevin R, Cenedella C, Dispigno M, Harwell JI, et al. Acceptability of sexually transmitted infection screening among women in short-term substance abuse treatment. Sex Transm Dis. 2002;29:752-5.
205. Lau A, Spark S, Tomnay J, Smith M, Fairley C, Guy R, et al. Socio-demographic and structural barriers to being tested for chlamydia in general practice. Med J Aust. 2016;204:112-5.
206. Lee J, Seo YB, Park JJ, Jeong SK. The evaluation and risk assessment of sexually transmitted disease in Korean adolescents at risk. Int J Infect Dis. 2016;45:202.
207. Lee SJ, Cho YH, Ha US, Kim SW, Yoon MS, Bae K. Sexual behavior survey and screening for chlamydia and gonorrhea in university students in South Korea. Int J Urol. 2005;12:187-93.
208. Lee T, Ganesan A. Results of a pilot screening programme for genital and extragenital gonococcal and chlamydial infections in a military population following the repeal of 'Don't Ask, Don't Tell'. Sex Transm Infect. 2015;91:233.
209. Leichliter JS, Copen C, Dittus PJ. Confidentiality Issues and Use of Sexually Transmitted Disease Services Among Sexually Experienced Persons Aged 15-25 Years - United States, 2013-2015. MMWR Morb Mortal Wkly Rep. 2017;66:237-41.
210. León SR, Konda KA, Klausner JD, Jones FR, Cáceres CF, Coates TJ. Chlamydia trachomatis infection and associated risk factors in a low-income marginalized urban population in coastal Peru. Rev Panam Salud Publica. 2009;26:39-45.
211. Leston JD, Jessen CM, Simons BC. Alaska Native and rural youth views of sexual health: a focus group project on sexually transmitted diseases, HIV/AIDS, and unplanned pregnancy. Am Indian Alsk Native Ment Health Res. 2012;19:1-14.
212. Lewkowicz C, Anderson E, Teelin K. Adolescents chose vaginal swabs over urine sample for chlamydia screening when recommended by physician. J Adolesc Health. 2015;56:S80.
213. Li WY, Liabsuetrakul T, Stray-Pedersen B, Li YJ, Guo LJ, Qin WZ. The effects of mode of delivery and time since birth on chronic pelvic pain and health-related quality of life. Int J Gynaecol Obstet. 2014;124:139-42.
214. Li X, Wang B, Fang X, Zhao R, Stanton B, Hong Y, et al. Short-term effect of a cultural adaptation of voluntary counseling and testing among female sex workers in China: A quasi-experimental trial. AIDS Educ Prev. 2006;18:406-19.
215. Lippman SA, Jones HE, Luppi CG, Pinho AA, Veras MA, van de Wijgert JH. Home-based self-sampling and self-testing for sexually transmitted infections: acceptable and feasible alternatives to provider-based screening in low-income women in Sao Paulo, Brazil. Sex Transm Dis. 2007;34:421-8.
216. Llewellyn C, Pollard A, Miners A, Richardson D, Fisher M, Cairns J, et al. Understanding patient choices for attending sexually transmitted infection testing services: a qualitative study. Sex Transm Infect. 2012;88:504-9.
217. Llewellyn C, Pollard A, Smith H, Fisher M, Home Sampling Kit Study G. Are home sampling kits for sexually transmitted infections acceptable among men who have sex with men? J Health Serv Res Policy. 2009;14:35-43.
218. Loades N, De Visser R. A mixed-method study of how to increase STI screening among young people. Sex Transm Infect. 2012;88(Suppl 1):A74.
219. Logie CH, Lacombe-Duncan A, Weaver J, Navia D, Este D. The 'queer women conversations' groupbased HIV and STI prevention intervention for Lesbian, Bisexual, queer and other women who have sex with women in toronto and Calgary, Canada: Results from a non-randomized cohort pilot study. Can J Infect Dis Med Microbiol. 2015;26(Suppl B):120B.
220. Lorimer K, McDaid L. Young men's views toward the barriers and facilitators of Internet-based Chlamydia trachomatis screening: qualitative study. J Med Internet Res. 2013;15:E265.
221. Lorimer K, Reid ME, Hart GJ. Willingness of young men and women to be tested for Chlamydia trachomatis in three non-medical settings in Glasgow, UK. J Fam Plann Reprod Health Care. 2009;35:21-6.
222. Ludlam AH, Saxton PJ, Dickson NP, Adams J. Respondent-driven sampling among gay and bisexual men: experiences from a New Zealand pilot study. BMC Res Notes. 2015;8:549.
223. Lusk MJ, Garden FL, Rawlinson WD, Naing ZW, Cumming RG, Konecny P. Cervicitis aetiology and case definition: a study in Australian women attending sexually transmitted infection clinics. Sex Transm Infect. 2016;92:175-81.
224. Lydie N, de Barbeyrac B, Bluzat L, Le Roy C, Kersaudy-Rahib D. Chlamyweb Study I: rationale, design and acceptability of an internet-based chlamydia testing intervention. Sex Transm Infect. 2017;93:179-87.
225. Maastricht University Medical Center. Incubation Time and Test of Cure of Chlamydia Trachomatis (Incure). 2011. ClinicalTrials.gov registration number: NCT01448876.
226. Macauley S, Creighton S. Testing commercial sex workers for chlamydia and gonorrhoea on outreach. Sex Transm Infect. 2009;85:231-2.
227. MacMillan S, McKenzie H, Flett G, Templeton A. Feasibility of patient-collected vulval swabs for the diagnosis of Chlamydia trachomatis in a family planning clinic: a pilot study. Br J Fam Plann. 2000;26:202-6.
228. Madge S, Elford J, Lipman MC, Mintz J, Johnson MA. Screening for sexually transmitted diseases in an HIV testing clinic; uptake and prevalence. Genitourin Med. 1996;72:347-51.
229. Mallinson H, Hopwood J, Skidmore S, Fenton K, Phillips C, Jones I. Provision of chlamydia testing in a nationwide service offering termination of pregnancy: with data capture to monitor prevalence of infection. Sex Transm Infect. 2002;78:416-21.
230. Malta M, Bastos FI, Strathdee SA, Cunnigham SD, Pilotto JH, Kerrigan D. Knowledge, perceived stigma, and care-seeking experiences for sexually transmitted infections: a qualitative study from the perspective of public clinic attendees in Rio de Janeiro, Brazil. BMC Public Health. 2007;7:18.
231. Marcus JL, Bernstein KT, Stephens SC, Snell A, Kohn RP, Liska S, et al. Sentinel surveillance of rectal chlamydia and gonorrhea among males-San Francisco, 2005-2008. Sex Transm Dis. 2010;37:59-61.
232. Marion LN, Finnegan L, Campbell RT, Szalacha LA. The Well Woman Program: a community-based randomized trial to prevent sexually transmitted infections in low-income African American women. Res Nurs Health. 2009;32:274-85.
233. Martin SJ, Currie MJ, Deeks LS, Cooper GM, Parker RM, Del Rosario R, et al. Do cash incentives increase the uptake of chlamydia testing in pharmacies? Sex Transm Infect. 2012;88(Suppl 1):A35.
234. Matiluko A, Crystal A. Chlamydia in colposcopy clinics: To screen or not to screen? J Obstet Gynaecol. 2006;26:454-6.
235. McBride KR, Goldsworthy RC, Fortenberry JD. Patient and partner perspectives on patient-delivered partner screening: Acceptability, benefits, and barriers. AIDS Patient Care STDs. 2010;24:631-7.
236. McCarthy O, French RS, Roberts I, Free C. Simple steps to develop trial follow-up procedures. Trials. 2016;17:28.
237. McCormick K, Findlay R, Cunningham C. Chlamydia partner notification outcomes: A comparison of telephone and face-to-face consultations. Int J STD AIDS. 2013;24(Suppl 1):17-8.
238. McDaid LM, Lorimer K. A Proactive approach to online chlamydia screening: Qualitative exploration of young men's perspectives of the barriers and facilitators. Sex Transm Infect. 2013;89(Suppl 1):A348.
239. McRee AL, Esber A, Reiter PL. Acceptability of home-based chlamydia and gonorrhea testing among a national sample of sexual minority young adults. Perspect Sex Reprod Health. 2015;47:3-10.
240. McSorley J, Brook G, Shaw A. Retrospective study of the effect of enhanced systematic STI screening, facilitated by the use of electronic patient records (EPR), in an HIV cohort. HIV Med. 2013;14(Suppl 2):76.
241. Mehringer M, Hertz D, Di Paolo A. A qualitative exploration of stiscreening practices and barriers among obgyns and family practitioners in the united states. Value Health. 2014;17:A162.
242. Mehta SD, Rompalo A, Rothman RE, Londner MS, Zenilman JM. Generalizability of STD screening in urban emergency departments: comparison of results from inner city and urban sites in Baltimore, Maryland. Sex Transm Dis. 2003;30:143-8.
243. Mellor J. Implementing NICE guidelines on one-to-one interventions to reduce the risk of sexually transmitted infections. HIV Med. 2010;11:54.
244. Merritt TD, Durrheim DN, Hope K, Byron P. General practice intervention to increase opportunistic screening for chlamydia. Sex Health. 2007;4:249-51.
245. Mevissen FE, Ruiter RA, Meertens RM, Zimbile F, Schaalma HP. Justify your love: testing an online STI-risk communication intervention designed to promote condom use and STI-testing. Psychol Health. 2011;26:205-21.
246. Mimiaga MJ, Thomas B, Mayer KH, Regenauer KS, Dange A, Andres Bedoya C, et al. A randomized clinical efficacy trial of a psychosocial intervention to strengthen self-acceptance and reduce HIV risk for MSM in India: study protocol. BMC Public Health. 2018;18:890.
247. Moazenchi M, Totonchi M, Salman Yazdi R, Hratian K, Mohseni Meybodi MA, Ahmadi Panah M, et al. The impact of Chlamydia trachomatis infection on sperm parameters and male fertility: A comprehensive study. Int J STD AIDS. 2018;29:466-73.
248. Mooney-Somers J, Olsen A, Erick W, Scott R, Akee A, Kaldor J, et al. Learning from the past: young Indigenous people's accounts of blood-borne viral and sexually transmitted infections as resilience narratives. Cult Health Sex. 2011;13:173-86.
249. Morgan J, Bell A. The highs and lows of opportunistic Chlamydia testing: uptake and detection in Waikato, New Zealand. Sex Transm Infect. 2009;85:452-4.
250. Morris JL, Lippman SA, Philip S, Bernstein K, Neilands TB, Lightfoot M. Sexually transmitted infection related stigma and shame among African American male youth: implications for testing practices, partner notification, and treatment. AIDS Patient Care STDS. 2014;28:499-506.
251. Morton AN, Wakefield T, Tabrizi SN, Garland SM, Fairley CK. An outreach programme for sexually transmitted infection screening in street sex workers using self-administered samples. Int J STD AIDS. 1999;10:741-3.
252. Moss NJ, Ahrens K, Kent CK, Klausner JD, Brunham RC, Pourbohloul B, et al. The decline in clinical sequelae of genital Chlamydia trachomatis infection supports current control strategies. The unexpected impact of a Chlamydia trachomatis infection control program on susceptibility to reinfection. J Infect Dis. 2006;193:1336-9.
253. Mullan H, Oroz C, Richards J, Lee J. Staff, associate specialist and specialty (SAS) doctors' national audit on the management of gonorrhoea in the UK. Sex Transm Infect. 2016;92(Suppl 1):A40.
254. Mullinax M, Schick V, Rosenberg J, Herbenick D, Reece M. Screening for Sexually Transmitted Infections (STIs) Among a Heterogeneous Group of WSW(M). Int J Sex Health. 2016;28:9-15.
255. Mundy L, Hiller J. Rapid point-of-care for the detection of Chlamydia in individuals at risk of trachoma. Canberra (AU): Australia and New Zealand Horizon Scanning Network, Commonwealth of Australia; 2006. <http://www.horizonscanning.gov.au>. Accessed 11 Sept 2020.
256. Mustanski B, Madkins K, Greene GJ, Parsons JT, Johnson BA, Sullivan P, et al. Internet-Based HIV Prevention With At-Home Sexually Transmitted Infection Testing for Young Men Having Sex With Men: Study Protocol of a Randomized Controlled Trial of Keep It Up! 2.0. JMIR Res Protoc. 2017;6:E1.
257. National Institute of Allergy and Infectious Diseases (NIAID). Bacterial Vaginosis Home Screening to Prevent STDs. 2008. ClinicalTrials.gov registration number: NCT00667368.
258. National Institute of Allergy and Infectious Diseases (NIAID). Clinical Validation of a Molecular Test for Ciprofloxacin-Susceptibility in Neisseria Gonorrheaoe. 2016. ClinicalTrials.gov registration number: NCT02961751.
259. Ndiaye P, Fall A, Tal-Dia A, Faye A, Diongue M. Knowledge, attitudes and practices related to STD and HIV/AIDS: Men having sex with men in Senegal. Rev Epidemiol Sante Publique. 2011;59:305-11.
260. Newman M. People at high risk for STDs used a variety of primary and secondary prevention strategies. Evid Based Nurs. 2002;5:92.
261. Nielsen A, De Costa A, Bågenholm A, Danielsson KG, Marrone G, Boman J, et al. Trial protocol: a parallel group, individually randomized clinical trial to evaluate the effect of a mobile phone application to improve sexual health among youth in Stockholm County. BMC Public Health. 2018;18:216.
262. Nielsen A, De Costa A, Danielsson KG, Salazar M. Repeat testing for chlamydia trachomatis, a "safe approach" to unsafe sex? a qualitative exploration among youth in Stockholm. BMC Health Serv Res. 2017;17:730.
263. Nielsen AM, De Costa A, Gemzell-Danielsson K, Marrone G, Boman J, Salazar M, et al. The MOSEXY trial: mobile phone intervention for sexual health in youth - A pragmatic randomised controlled trial to evaluate the effect of a smartphone application on sexual health in youth in Stockholm, Sweden. Sex Transm Infect. 2019.
264. Northwestern University. Keep It Up! 2.0: A Comparison of Two Online HIV Intervention Programs for Young Men Who Have Sex With Men (KIU!). 2013. ClinicalTrials.gov registration number: NCT01836445.
265. Nsuami MJ, Sanders LS, Taylor SN. Knowledge of sexually transmitted infections among high school students. Am J Health Educ. 2010;41:206-17.
266. Nsuami MJ, Taylor SN. Most adolescents who participate in school-based screenings for sexually transmitted infections do not perceive themselves at high risk of sexually transmitted infection. Int J STD AIDS. 2012;23:822-4.
267. NTR. PREdictive value of the Self-triagecard and Chlamydia trachomatis positivity rate in vocational School students. http://wwwwhoint/trialsearch/Trial2aspx?TrialID=NTR1410. 2008.
268. Nyari T, Nyari C, Woodward M, Meszaros G, Deak J, Nagy E, et al. Screening for Chlamydia trachomatis in asymptomatic women in Hungary: an epidemiological and cost-effectiveness analysis. Acta Obstet Gynecol Scand. 2001;80:300-6.
269. Nyari T, Woodward M, Kovacs L. Should all sexually active young women in Hungary be screened for Chlamydia trachomatis. Eur J Obstet Gynecol Reprod Biol. 2003;106:55-9.
270. Nyatsanza F, Murphy S, McSorley J, Brook G. The utility of personalised short message service (SMS) texts to remind patients at higher risk of STIs and HIV to re-attend for testing. Sex Transm Infect. 2015;91(Suppl 1):A32.
271. Nyatsanza F, Trivedy A, Brook G. The effect of introducing routine self-taken extra-genital swabs in a gum clinic cohort. Sex Transm Infect. 2015;91(Suppl 1):A59-60.
272. O'Byrne P, Orser L. Overfilled urine specimens for gonorrhea and chlamydia testing: Implications for practice. Appl Nurs Res. 2018;39:121-4.
273. O'Farrell N, Weiss HA. Effect of Chlamydia diagnosis on heterosexual relationships. Int J STD AIDS. 2013;24:722-6.
274. O'Sullivan P, O'Hora A, Cullen G, Kelly T. The epidemiology of genital chlamydia infection in Ireland; 1997-2008. Impact and control considerations. Ir J Med Sci. 2011;180(Suppl 7):S228.
275. Oakeshott P, Aghaizu A, Hay P, Reid F, Kerry S, Atherton H, et al. Is Mycoplasma genitalium in women the "New Chlamydia?" A community-based prospective cohort study. Clin Infect Dis. 2010;51:1160-6.
276. Oakeshott P, Aghaizu A, Reid F, Howell-Jones R, Hay PE, Sadiq ST, et al. Frequency and risk factors for prevalent, incident, and persistent genital carcinogenic human papillomavirus infection in sexually active women: community based cohort study. BMJ. 2012;344:E4168
277. Oakeshott P, Kerry-Barnard S, Fleming C, Phillips R, Drennan VM, Adams EJ, et al. 'Test n Treat' (TnT): a cluster randomized feasibility trial of on-site rapid Chlamydia trachomatis tests and treatment in ethnically diverse, sexually active teenagers attending technical colleges. Clinical microbiology and infection. 2019;25:865‐71.
278. Oh MK, Cloud GA, Fleenor M, Sturdevant MS, Nesmith JD, Feinstein RA. Risk for gonococcal and chlamydial cervicitis in adolescent females: incidence and recurrence in a prospective cohort study. J Adolesc Health. 1996;18:270-5.
279. Okwumabua TM, Peasant C, Anderson MB, Barnes E, Craig SD. Using deep reasoning questions to improve an email-based sexually transmitted infection prevention intervention. American Journal of Sexuality Education. 2018;13:452-69.
280. Oliphant J, Azariah S. Cervicitis: limited clinical utility for the detection of Mycoplasma genitalium in a cross-sectional study of women attending a New Zealand sexual health clinic. Sex Health. 2013;10:263-7.
281. Opaneye AA, Bashford J, Ashton V. A comparison of two methods for detection of Chlamydia trachomatis in the male urethra. J R Soc Promot Health. 2002;122:58-60.
282. Oster NV, Rothenberg R, McPhillips-Tangum CA, Gazmararian J, Franks AL. Chlamydia screening in a metropolitan Atlanta primary care clinic. South Med J. 2003;96:863-7.
283. Ott MA, Campbell J, Imburgia TM, Yang Z, Tu W, Auerswald CL. Community Engagement and Venue-Based Sampling in Adolescent Male Sexually Transmitted Infection Prevention Research. J Adolesc Health. 2018;62:S58-64.
284. Ouden DD, Derouin A, Silva S, Khan A. Screening for chlamydia: are you doing it? Nurse Pract. 2014;39:41-7.
285. Ouzounova-Raykova V, Ouzounova I, Mitov I. Chlamydia trachomatis infection as a problem among male partners of infertile couples. Andrologia. 2009;41:14-9.
286. Ouzounova-Raykova V, Rangelov S, Ouzounova I, Mitov I. Detection of Chlamydia trachomatis, Ureaplasma urealyticum and Mycoplasma hominis in infertile Bulgarian men with multiplex real-time polymerase chain reaction. APMIS. 2015;123:586-8.
287. Ovens K, Smit E, Barrett S. Should we treat or rescreen patients first with equivocal chlamydia and gonorrhoea NAAT results? Sex Transm Infect. 2015;91:A55.
288. Over E, Aar F, Gotz H, Benthem B, Lugner A, Suijkerbuijk A. Economic consequences of a restricted Dutch sexually transmitted infection-testing policy. Int J Technol Assess Health Care. 2017;33(Suppl 1):165.
289. Owings AJ, Clark LL, Rohrbeck P. Incident and recurrent Chlamydia trachomatis and Neisseria gonorrhoeae infections, active component, U.S. Armed Forces, 2010-2014. MSMR. 2016;23:20-8.
290. Pack RP. Explanatory model of sexual risk behavior and sexually transmitted disease prevalence in incarcerated. PhD [dissertation]. Alabama: University of Alabama at Birmingham; 1999. Available from: Dissertation Abstracts International: Section B: The Sciences and Engineering.
291. Passanisi A, Leanza V, Leanza G. The impact of sexually transmitted diseases on quality of life: Application of three validated measures. Giorn It Ost Gin. 2013;35:722-7.
292. Patel CG, Chesson HW, Guoyu T, Tao G. Racial Differences in Receipt of Chlamydia Testing Among Medicaid-Insured Women in 2013. Sex Transm Dis. 2016;43:147-51.
293. Patel P, Bush T, Mayer K, Milam J, Richardson J, Hammer J, et al. Routine brief risk-reduction counseling with biannual STD testing reduces STD incidence among HIV-infected men who have sex with men in care. Sex Transm Dis. 2012;39:470-4.
294. Patoureau M, Ollier V, Cartoux M. Sexual behaviors and acceptability of STI (including HIV) testing outside nightclubs in Saint-Gilles, Reunion. Sante Publique. 2012;24:523-32.
295. Pattanasin S, Dunne EF, Wasinrapee P, Tongtoyai J, Chonwattana W, Sriporn A, et al. Screening for Chlamydia trachomatis and Neisseria gonorrhoeae infection among asymptomatic men who have sex with men in Bangkok, Thailand. Int J STD AIDS. 2018;29:577-87.
296. Patterson-Rose S, Hesse E, Dize L, Gaydos C, Widdice L. Feasibility of screening and follow-up for sexually transmitted infections (STIs) using rapid and standard STI testing from a mobile health van. Sex Transm Dis. 2014;41:S61-S2.
297. Pedlow CT. Randomized controlled trial of a brief information, motivation, and behavioral skills intervention to reduce HIV/STD risk in young women. PhD [dissertation]. New York: Syracuse University; 2004. Available from: Dissertation Abstracts International: Section B: The Sciences and Engineering.
298. Pedrosa AF, Azevedo F, Lisboa C. Screening for Chlamydia infection in a sexually transmitted infection clinic: a missed opportunity? Int J Dermatol. 2015;54:405-9.
299. Peeling RW, Toye B, Jessamine P, Gemmill I. Noninvasive screening for genital chlamydial infections in asymptomatic men: Strategies and costs using a urine PCR assay. Can J Infect Dis. 1998;9:281-6.
300. Peipert J, Zhao Q, Stoddard A, McNicholas C, Schreiber C, Turok DK, et al. Impact of infection and intrauterine device use on fertility. Contraception. 2014;90:344.
301. Pennise M, Inscho R, Herpin K, Owens J, Bedard BA, Weimer AC, et al. Using smartphone apps in STD interviews to find sexual partners. Public Health Rep. 2015;130:245-52.
302. Peralta L, Collinetti E, Husman C, Gorle R. Screening adolescents for Chlamydia infection at non-traditional sites. J Adolesc Health. 2011;48:S62.
303. Perez-Hernandez I, Palacios R, Gonzalez-Domenech C, Garcia V, Marquez M, Clavijo E, et al. Should screening for Chlamydia trachomatis and Neisseria gonorrhoeae in HIV-men who have sex with men be recommended? J Int AIDS Soc. 2014;17(Suppl 3):19661.
304. Persson K, Hammas B, Janson H, Bjartling C, Dillner J, Dillner L. Decline of the new Swedish variant of Chlamydia trachomatis after introduction of appropriate testing. Sex Transm Infect. 2012;88:451-5.
305. Peterman TA, Tian LH, Metcalf CA, Satterwhite CL, Malotte CK, DeAugustine N, et al. High incidence of new sexually transmitted infections in the year following a sexually transmitted infection: a case for rescreening. Ann Intern Med. 2006;145:564-72.
306. Phillips R, Oakeshott P, Kerry-Barnard S, Reid F. 'Test n Treat (TnT)': a cluster-randomised feasibility trial of frequent, rapid-testing and same-day, on-site treatment to reduce rates of chlamydia in high-risk further education college students: statistical analysis plan. Trials. 2018;19.
307. Phipps W, Stanley H, Kohn R, Stansell J, Klausner JD. Syphilis, chlamydia, and gonorrhea screening in HIV-infected patients in primary care, San Francisco, California, 2003. AIDS Patient Care STDS. 2005;19:495-8.
308. Piazzetta RC, de Carvalho NS, de Andrade RP, Piazzetta G, Piazzetta SR, Carneiro R. Prevalence of chlamydia trachomatis and neisseria gonorrhoea infections in sexual actives young women at a Southern Brazilian City. Rev Bras Ginecol Obstet. 2011;33:328-33.
309. Piercy H. "It feels good to be told that I'm all clear": patients' accounts of retesting following genital chlamydial infection. Sex Transm Infect. 2006;82:330-3.
310. Pines HA, Patterson TL, Rangel G, Martinez G, Bazzi AR, Ulibarri MD, et al. STI/HIV test result disclosure between female sex workers and their primary, non-commercial male partners in two Mexico-US border cities: a prospective study. Sex Transm Infect. 2015;91:207-13.
311. Pinto CN, Dorn LD, Chinchilli VM, Du P, Chi G. Rural counties chlamydia and gonorrhea rates in Pennsylvania among adolescents and young adults. Ann Epidemiol. 2017;27:606-10.
312. Plax K, Garbutt J, Kaushik GN. HIV and Sexually Transmitted Infection Testing Among High-Risk Youths: Supporting Positive Opportunities With Teens (SPOT) Youth Center. Am J Public Health. 2015;105:1394-8.
313. Pollard A, Llewellyn C, Miners A, Smith H. The STIPP study: Patient-centred preferences for STI testing services: A qualitative perspective. HIV Med. 2010;11(Suppl 1):92-3.
314. Pope Z, Hodge D, Donastorg Y, Khosla S, Lerebours L, Brito M. Sexual risk behaviors and prevalence of sexually transmitted diseases in a cohort of Dominican men who have sex with men. Int J Infect Dis. 2014;21(Suppl 1):424.
315. Powell R, Pattison HM, Marriott JF. Perceptions of Self-Testing for Chlamydia: Understanding and Predicting Self-Test Use. Healthcare (Basel). 2016;4:25.
316. Price MJ, Horner PJ, Ades AE. Risk of reproductive complications following chlamydia testing. Lancet Infect Dis. 2016;16:1223-4.
317. Purcell HN, Gaydos C, Widdice L. Preference for and acceptability of self-collection of pharyngeal swabs for sexually transmitted infection testing. J Adolesc Health. 2018;62(Suppl 1):S81.
318. Quinn N, Agreda P, Joffe A, Gaydos C. Prevalence of chlamydia trachomatis and Neisseria gonorrhoeae among college students who attended the Johns Hopkins university health and wellness center from 2009 TO 2010. Sex Transm Infect. 2011;87(Suppl 1):A151-2.
319. Radovic A, Burstein G, Murray P, Hall C, Sucato G. Adolescent preference for expedited partner therapy (EPT) versus standard referral for sexually transmitted diseases (STI) partner management. J Adolesc Health. 2011;48:S62-3.
320. Rahman M, Khan M, Longfellow L. Efficient gonorrhoea and chlamydia control and prevention through partner notification in high morbidity STD states: A louisiana perspective. Sex Transm Infect. 2011;87:A328.
321. Rawre J, Dhawan B, Malhotra N, Sreenivas V, Broor S, Chaudhry R. Prevalence and distribution of Chlamydia trachomatis genovars in Indian infertile patients: a pilot study. APMIS. 2016;124:1109-15.
322. Reed JL, Huppert JS, Taylor RG, Gillespie GL, Byczkowski TL, Kahn JA, et al. Improving sexually transmitted infection results notification via mobile phone technology. J Adolesc Health. 2014;55:690-7.
323. Reed JL, Simendinger L, Griffeth S, Kim HG, Huppert JS. Point-of-care testing for sexually transmitted infections increases awareness and short-term abstinence in adolescent women. J Adolesc Health. 2010;46:270-7.
324. Reed JL, Thistlethwaite JM, Huppert JS. STI research: recruiting an unbiased sample. J Adolesc Health. 2007;41:14-8.
325. Reed JL, Zaidi MA, Woods TD, Bates JR, Britto MT, Huppert JS. Impact of Post-visit Contact on Emergency Department Utilization for Adolescent Women with a Sexually Transmitted Infection. J Pediatr Adolesc Gynecol. 2015;28:144-8.
326. Reekie J, Donovan B, Guy R, Hocking JS, Kaldor JM, Mak DB, et al. Risk of Pelvic Inflammatory Disease in Relation to Chlamydia and Gonorrhea Testing, Repeat Testing, and Positivity: A Population-Based Cohort Study. Clin Infect Dis. 2018;66:437-43.
327. Reekie J, Donovan B, Guy R, Hocking JS, Kaldor JM, Mak DB, et al. Trends in chlamydia and gonorrhoea testing and positivity in Western Australian Aboriginal and non-Aboriginal women 2001-2013: a population-based cohort study. Sex Health. 2017;14:574-80.
328. Reekie J, Donovan B, Guy R, Mak D, Pearson S, Liu B. Trends in chlamydia and gonorrhoea testing and positivity in western Australian Women, 1998-2013. Sex Transm Infect. 2015;91(Suppl 2):A135.
329. Reisner SL, Hughto JM, Pardee DJ, Kuhns L, Garofalo R, Mimiaga MJ. LifeSkills for Men (LS4M): Pilot Evaluation of a Gender-Affirmative HIV and STI Prevention Intervention for Young Adult Transgender Men Who Have Sex with Men. J Urban Health. 2016;93:189-205.
330. Reno HE, Brethauer C, Spear D, Knaup R, Stoner BP. Enhanced oropharyngeal and rectal testing for Neisseria Gonorrhoeae and chlamydia trachomatis at a public STI clinic. Sex Transm Infect. 2013;89(Suppl 1):A169.
331. Reynolds R, Oakman T. Genital chlamydia in southern New South Wales: an ecological analysis of testing and notification patterns 2004-2008. Aust J Rural Health. 2010;18:159-65.
332. Ricardo Marchezini RM, Aparecida Machado de Oliveira D, Fagundes LJ, Itsuko Ciosak S. Sexually Transmitted infections in specialized service: who they are and who has them? Rev Enferm UFPE. 2018;12:137-49.
333. Rieg G, Lewis RJ, Miller LG, Witt MD, Guerrero M, Daar ES. Asymptomatic sexually transmitted infections in HIV-infected men who have sex with men: prevalence, incidence, predictors, and screening strategies. AIDS Patient Care STDS. 2008;22:947-54.
334. Rietmeijer CA, Oh MK, Bull SS, Brown PR, Wang SA, Mertz KJ. Monitoring std prevalence and reproductive health care among high-risk adolescent women. J Pediatr Adolesc Gynecol. 2000;13:90-1.
335. Rietmeijer CA, Van Bemmelen R, Judson FN, Douglas JM, Jr. Incidence and repeat infection rates of Chlamydia trachomatis among male and female patients in an STD clinic: implications for screening and rescreening. Sex Transm Dis. 2002;29:65-72.
336. Roberts W, Chauhan M, Sankar N. Is it appropriate for men who have sex with men to attend health-care assistant-led asymptomatic screening clinics? International Journal of STD and AIDS. 2013;24(Suppl 1):39.
337. Rodriguez-Hart C, Gray I, Kampert K, White M, Wolfe C, Wilson M, et al. Just text me! Texting sexually transmitted disease clients their test results in Florida, February 2012-January 2013. Sex Transm Dis. 2015;42:162-7.
338. Romer A, Shew ML, Ofner S, Gilliam ML, Martins SL, Fortenberry JD. Depot medroxyprogesterone acetate use is not associated with risk of incident sexually transmitted infections among adolescent women. J Adolesc Health. 2013;52:83-8.
339. Ronda G, van Bokhoven L, van der Weijden T. Psychosocial determinants of the intention to use a chlamydia home self-test: awareness of risk behaviour and test accuracy are important elements of educational interventions. Sex Health. 2013;10:93-4.
340. Roston A, Suleta K, Stempinski K, Keith L, Patel A. Get Yourself Tested 2011-2012: findings and prevalence of Chlamydia trachomatis and Neisseria gonorrhoeae at an urban public health system. Int J STD AIDS. 2015;26:322-8.
341. Rotblatt H, Montoya JA, Plant A, Guerry S, Kerndt PR. There's no place like home: first-year use of the "I Know" home testing program for chlamydia and gonorrhea. Am J Public Health. 2013;103:1376-80.
342. Roth A, Van Der Pol B, Dodge B, Fortenberry JD, Zimet G. Future chlamydia screening preferences of men attending a sexually transmissible infection clinic. Sex Health. 2011;8:419-26.
343. Roth AM, Rosenberger JG, Reece M, Van Der Pol B. A methodological approach to improve the sexual health of vulnerable female populations: incentivized peer-recruitment and field-based STD testing. J Health Care Poor Underserved. 2012;23:367-75.
344. Roth AM, Rosenberger JG, Reece M, Van Der Pol B. Expanding sexually transmitted infection screening among women and men engaging in transactional sex: the feasibility of field-based self-collection. Int J STD AIDS. 2013;24:323-8.
345. Rou K, Guan J, Wu Z, Li L, Rotheram MJ, Detels R, et al. Demographic and behavioral factors associated with HIV testing in China. J Acquir Immune Defic Syndr. 2009;50:432-4.
346. Roush S, Aguinaldo J, Beauvoir C, Renteria R, Puffer M, Cantu M. Student perceptions and utilization of school-based health centers in los angeles: Results of an STD prevention campaign. Sex Transm Dis. 2016;43 (Suppl 2):S152.
347. Rucker RR. HIV & STD testing among heterosexual African American men. PhD [dissertation]. Illinois: University of Illinois at Urbana-Champaign; 2010. Available from: Dissertation Abstracts International: Section B: The Sciences and Engineering.
348. Rudd S, Gemelas J, Reilley B, Leston J, Tulloch S. Integrating clinical decision support to increase HIV and chlamydia screening. Prev Med. 2013;57:908-9.
349. Rusch M, Shoveller J, Burgess S, Stancer K, Patrick D, Tyndall M. Association of sexually transmitted disease-related stigma with sexual health care among women attending a community clinic program. Sex Transm Dis. 2008;35:553-7.
350. Rutland E, Roe H, Weaver A. Health promotional messages in short message service (SMS) follow-up of GU medicine clinic defaulters; A tool to improve subsequent attendance rates? Sex Transm Infect. 2012;88(Suppl 1):A4-5.
351. Sagor RS, Golding J, Giorgio MM, Blake DR. Power of Knowledge: Effect of Two Educational Interventions on Readiness for Chlamydia Screening. Clin Pediatr (Phila). 2016;55:717-23.
352. Salerno J, Darling-Fisher C, Hawkins NM, Fraker E. Identifying relationships between high-risk sexual behaviors and screening positive for chlamydia and gonorrhea in school-wide screening events. J Sch Health. 2013;83:99-104.
353. Sales JM, Spitalnick J, Milhausen RR, Wingood GM, DiClemente RJ, Salazar LF, et al. Validation of the worry about sexual outcomes scale for use in STI/HIV prevention interventions for adolescent females. Health Educ Res. 2009;24:140-52.
354. Sanchez J, Campos PE, Courtois B, Gutierrez L, Carrillo C, Alarcon J, et al. Prevention of sexually transmitted diseases (STDs) in female sex workers: prospective evaluation of condom promotion and strengthened STD services. Sex Transm Dis. 2003;30:273-9.
355. Santer M, Wyke S, Warner P. Women's experiences of Chlamydia screening. Qualitative interviews with women in primary care. Eur J Gen Pract. 2003;9:56-61.
356. Satterwhite CL, Gray AM, Berman S, Weinstock H, Kleinbaum D, Howards PP. Chlamydia trachomatis infections among women attending prenatal clinics: United States, 2004-2009. Sex Transm Dis. 2012;39:416-20.
357. Satterwhite CL, Newman D, Collins D, Torrone E. Chlamydia Screening and Positivity in Juvenile Detention Centers, United States, 2009–2011. Women Health. 2014;54:712-25.
358. Savage E, Marsh K, Lowndes CM, Duffell S, Zaman A, Hughes G. Partner notification for gonorrhoea: Analysis of outcomes using surveillance data. Sex Transm Infect. 2012;88(Suppl 1):A2.
359. Schaalma HP, Kok G, Bosker RJ, Parcel GS, Peters L, Poelman J, et al. Planned development and evaluation of AIDS/STD education for secondary school students in The Netherlands: short-term effects. Health Educ Q. 1996;23:469-87.
360. Schick V, Van Der Pol B, Dodge B, Baldwin A, Fortenberry JD. A mixed methods approach to assess the likelihood of testing for STI using self-collected samples among behaviourally bisexual women. Sex Transm Infect. 2015;91:329-33.
361. Schmid BV, Over EA, van den Broek IV, Op de Coul EL, van Bergen JE, Fennema JS, et al. Effects of population based screening for Chlamydia infections in the Netherlands limited by declining participation rates. PLoS ONE. 2013;8:E58674.
362. Schmidt AJ, Marcus U. Self-reported history of sexually transmissible infections (STIs) and STI-related utilization of the German health care system by men who have sex with men: data from a large convenience sample. BMC Infect Dis. 2011;11:132.
363. Schmutz C, Burki D, Frei R, Mausezahl-Feuz M, Mausezahl D. Testing for Chlamydia trachomatis: Time trends in positivity rates in the canton of Basel-Stadt, Switzerland. Epidemiol Infect. 2013;141:1953-64.
364. Schneider K, FitzGerald M, Byczkowski T, Reed J. Screening for Asymptomatic Gonorrhea and Chlamydia in the Pediatric Emergency Department. Sex Transm Dis. 2016;43:209-15.
365. Scholes D, Grothaus L, McClure J, Reid R, Fishman P, Sisk C, et al. A randomized trial of strategies to increase chlamydia screening in young women. Prev Med. 2006;43:343-50.
366. Schwartz RM, Hogben M, Liddon N, Augenbraun M, McCormack WM, Rubin S, et al. Coping with a diagnosis of C trachomatis or N gonorrhoeae: psychosocial and behavioral correlates. J Health Psychol. 2008;13:921-9.
367. Schwebke JR, Lee JY, Lensing S, Philip SS, Wiesenfeld HC, Sena AC, et al. Home Screening for Bacterial Vaginosis to Prevent Sexually Transmitted Diseases. Clin Infect Dis. 2016;62:531-6.
368. Scoular A, Duncan B, Hart G. "That sort of place...where filthy men go...": A qualitative study of women's perceptions of genitourinary medicine services. Sex Transm Infect. 2001;77:340-3.
369. Scoular A, McCartney R, Kinn S, Carr S, Walker A. The 'real-world' impact of improved diagnostic techniques for Chlamydia trachomatis infection in Glasgow. Commun Dis Public Health. 2001;4:200-4.
370. Semberova J, Ulcova-Gallova Z, Manthay A, Piskata M, Balvin M, Milichovska L, et al. ELISA Detection of Antichlamydial Antibodies in Non Standard Biological Fluids and Their Relationship to Female Infertility. Clin Appl Immunol. 2003;2:250-4.
371. Senkus E, Gomez H, Dirix L, Jerusalem G, Murray E, Van Tienhoven G, et al. Attitudes of young patients with breast cancer toward fertility loss related to adjuvant systemic therapies. EORTC study 10002 BIG 3-98. Psychooncology. 2014;23:173-82.
372. Sethi S, Rajkumari N, Dhaliwal L, Roy A. Association of mycoplasma genitalium with cervicitis in North Indian women attending gynecologic clinics. Sex Transm Infect. 2013;89(Suppl 1):A240-1.
373. Sexton ME, Baker JJ, Nakagawa K, Li Y, Perkins R, Slack RS, et al. How reliable is self-testing for gonorrhea and chlamydia among men who have sex with men? J Fam Pract. 2013;62:70-8.
374. Shafer MA, Moncada J, Boyer CB, Betsinger K, Flinn SD, Schachter J. Comparing first-void urine specimens, self-collected vaginal swabs, and endocervical specimens to detect Chlamydia trachomatis and Neisseria gonorrhoeae by a nucleic acid amplification test. J Clin Microbiol. 2003;41:4395-9.
375. Shafii T, Benson S, Morrison D, Hughes J, Golden M, Holmes K. A pilot randomised controlled trial of an interactive computer-based intervention for sexual health in adolescents and young adults. Sex Transm Infect. 2015;91:A33.
376. Shafii T, Benson SK, Morrison DM, Hughes JP, Golden MR, Holmes KK. Results from e-KISS: electronic-KIOSK Intervention for Safer Sex: a pilot randomized controlled trial of an interactive computer-based intervention for sexual health in adolescents and young adults. PLoS ONE. 2019;14:E0209064.
377. Shaikh RA, Simonsen KA, O’Keefe A, Earley M, Foxall M, Islam KM, et al. Comparison of Opt-In Versus Opt-Out Testing for Sexually Transmitted Infections Among Inmates in a County Jail. J Correct Health Care. 2015;21:408-16.
378. Shamash Z, Catallozzi M, Dayan P, Chernick L. Exploring attitudes and receptivity to expedited partner therapy for adolescents in an Urban pediatric emergency department: A mixed-methods study. J Adolesc Health. 2015;56(Suppl 1):S78-9.
379. Sharp SR, Allan S. Improving clinical standards in GU medicine: A retrospective audit of Neisseria gonorrhoeae. Sex Transm Infect. 2012;88(Suppl 1):A52-3.
380. Shaw J, Ahmad S. Extragenital screening in women-is TMA value for money? HIV Med. 2014;15(Suppl 3):170.
381. Shaw JW, Ahmad S. Extragenital screening in women - Is TMA value for money? Sex Transm Infect. 2013;89(Suppl 1):A110.
382. Shaw JW, Ahmad S. Seek and you shall find - Value of extragenital chlamydia and gonorrhoea TMA testing in a cohort of MSM. Sex Transm Infect. 2013;89(Suppl 1):A110.
383. Shawe J, White A, Ball A, Stretch R, Cannon E, Rees L, et al. Improving the sexual health of homeless people: Does providing nurse-led care within hostels improve contraceptive use and uptake of sexual health screening? Eur J Contracept Reprod Health Care. 2014;91(Suppl 1):S140.
384. Sheeder J, Stevens-Simon C, Lezotte D, Glazner J, Scott S. Cervicitis: to treat or not to treat? The role of patient preferences and decision analysis. J Adolesc Health. 2006;39:887-92.
385. Shepherd L, Harwood H. The role of STI-related attitudes on screening attendance in young adults. Psychol Health Med. 2017;22:753-8.
386. Shepherd L, Smith MA. The role of fear in predicting sexually transmitted infection screening. Psychol Health. 2017;32:876-94.
387. Shew ML, Ermel AC, Tong Y, Tu W, Qadadri B, Brown DR. Episodic detection of human papillomavirus within a longitudinal cohort of young women. J Med Virol. 2015;87:2122-9.
388. Shi L, Xie Y, Liu J, Kissinger P, Khan M. Is out-of-pocket cost a barrier to receiving repeat tests for chlamydia and gonorrhoea? Int J STD AIDS. 2013;24:301-6.
389. Short VL, Totten PA, Ness RB, Astete SG, Kelsey SF, Haggerty CL. Clinical presentation of Mycoplasma genitalium Infection versus Neisseria gonorrhoeae infection among women with pelvic inflammatory disease. Clin Infect Dis. 2009;48:41-7.
390. Shrier L, Ancheta R, Goodman E, Chiou V, Lyden M, Emans S. Randomized controlled trial of a safer sex intervention for high-risk adolescent girls. Arch Pediatr Adolesc Med. 2001;155:73-9.
391. Sieck CJ, Dembe AE. Results of a pilot study of pre-release STD testing and inmates' risk behaviors in an Ohio prison. J Urban Health. 2011;88:690-9.
392. Silva-Santisteban A, Konda KA, Leon S, Salazar X, Sandoval C, Clark J, et al. Effectiveness of communidades positivas: A randomised community-level combination HIV prevention intervention for men who have sex with men in Peru. Sex Transm Infect. 2013;89(Suppl 1):A325-6.
393. Silver BJ, Guy RJ, Wand H, Ward J, Rumbold AR, Fairley CK, et al. Incidence of curable sexually transmissible infections among adolescents and young adults in remote Australian Aboriginal communities: analysis of longitudinal clinical service data. Sex Transm Infect. 2015;91:135-41.
394. Simms I, Catchpole MA, Robinson AJ, Laas C. Provision of diagnostic services for genital chlamydial infection in genitourinary medicine clinics: England and Wales 1996. Genitourin Med. 1997;73:147-8.
395. Simms I, Hopwood J, Mallinson H, Rogers P, Webb A. Changing screening strategies for genital chlamydia in family planning clinics: a good public health strategy? Eur J Contracept Reprod Health Care. 2000;5:91-5.
396. Simms I, Talebi A, Rhia J, Horner P, French RS, Sarah R, et al. The English National Chlamydia Screening Programme: variations in positivity in 2007/2008. Sex Transm Dis. 2009;36:522-7.
397. Skidmore S, Horner P, Herring A, Sell J, Paul I, Thomas J, et al. Vulvovaginal-swab or first-catch urine specimen to detect Chlamydia trachomatis in women in a community setting? J Clin Microbiol. 2006;44:4389-94.
398. Skjeldestad FE, Marsico MA, Sings HL, Nordbo SA, Storvold G. Incidence and risk factors for genital Chlamydia trachomatis infection: a 4-year prospective cohort study. Sex Transm Dis. 2009;36:273-9.
399. Smith NA, Carlin EM, Boag FC. Screening for sexually transmitted diseases in an HIV testing clinic: uptake and prevalence. Genitourin Med. 1997;73:229-30.
400. Snead MC, Wiener J, Ewumi S, Phillips C, Flowers L, Hylton-Kong T, et al. Prevalence and risk factors associated with STIs among women initiating contraceptive implants in Kingston, Jamaica. Sex Transm Infect. 2017;93:503-7.
401. Soetens LC, van Benthem BH, Op de Coul EL. Chlamydia test results were associated with sexual risk behavior change among participants of the Chlamydia screening implementation in The Netherlands. Sex Transm Dis. 2015;42:109-14.
402. Soni S, Alexander S, Verlander N, Saunders P, Richardson D, Fisher M, et al. The prevalence of urethral and rectal Mycoplasma genitalium and its associations in men who have sex with men attending a genitourinary medicine clinic. Sex Transm Infect. 2010;86:21-4.
403. Soni S, White JA. Self-screening for Neisseria gonorrhoeae and Chlamydia trachomatis in the human immunodeficiency virus clinic--high yields and high acceptability. Sex Transm Dis. 2011;38:1107-9.
404. Sonnenberg P, Clifton S, Beddows S, Field N, Soldan K, Tanton C, et al. Prevalence, risk factors, and uptake of interventions for sexually transmitted infections in Britain: findings from the National Surveys of Sexual Attitudes and Lifestyles (Natsal). Lancet. 2013;382:1795-806.
405. Sood T, Sally D, Spencer N, Banerjee A, Hinchley G. Feasibility of screening for Chlamydia trachomatis in young men attending an emergency department. Emerg Med J. 2008;25:428-30.
406. Souleymanov R, Lachowsky NJ, Brennan DJ. Regional differences in HIV and STI testing, treatment, and notification among Ontario Gay, Bisexual, and other men who use the internet to seek sex with other men (MISM). Can J Infect Dis Med Microbiol. 2015;26(Suppl B):97B.
407. Sow CK. The decision to name sex partners: Determinants of provider referral compliance in chlamydia-infected adolescents (named sex partners). PhD [dissertation]. Louisiana: Tulane University; 1999. Available from: Dissertation Abstracts International: Section B: The Sciences and Engineering.
408. Spielberg F, Branson BM, Goldbaum GM, Lockhart D, Kurth A, Celum CL, et al. Overcoming barriers to HIV testing: preferences for new strategies among clients of a needle exchange, a sexually transmitted disease clinic, and sex venues for men who have sex with men. J Acquir Immune Defic Syndr. 2003;32:318-27.
409. Spiteri G, Amato-Gauci AJ. Surveillance of sexually transmitted infections among men who have sex with men in the European Union/European Economic Area. Int J STD AIDS. 2015;26:6.
410. Sripada S, Logan S, McGillivray S, McKenzie H, Templeton A, Hamilton M, et al. Opportunistic screening for Chlamydia trachomatis in men attending three different secondary healthcare settings. Sex Transm Infect. 2007;83:282-5.
411. Stary A, Heller-Vitouch C, Binder M, Geusau A, Stary G, Rappersberger K, et al. Gonococcal infections in Austria: a long-term observation of prevalence and resistance profiles from 1999 to 2014. J Dtsch Dermatol Ges. 2015;13:1136-45.
412. Steenbeek A, Tyndall M, Sheps S, Rothenberg R. An epidemiological survey of chlamydial and gonococcal infections in a Canadian arctic community. Sex Transm Dis. 2009;36:79-83.
413. Steiner KC, Davila V, Kent CK, Chaw JK, Fischer L, Klausner JD. Field-delivered therapy increases treatment for chlamydia and gonorrhea. Am J Public Health. 2003;93:882-4.
414. Sutton TL, Martinko T, Hale S, Fairchok MP. Prevalence and high rate of asymptomatic infection of Chlamydia trachomatis in male college Reserve Officer Training Corps cadets. Sex Transm Dis. 2003;30:901-4.
415. Swartzendruber A, DiClemente R, Sales J, Brown JL, Rose E. Predictors of repeat chlamydia trachomatis and/or neisseria gonorrhoeae infections among African-American adolescent females. Sex Transm Infect. 2011;87:A30.
416. Swartzendruber A, DiClemente RJ, Sales JM, Brown JL, Rose ES. Correlates of incident Trichomonas Vaginalis infections among African-American adolescent females. Sex Transm Infect. 2013;89(Suppl 1):A33.
417. Syred J, Naidoo C, Woodhall SC, Baraitser P. Would you tell everyone this? Facebook conversations as health promotion interventions. J Med Internet Res. 2014;16:E108.
418. Sznitman SR, Carey MP, Vanable PA, DiClemente RJ, Brown LK, Valois RF, et al. The impact of community-based sexually transmitted infection screening results on sexual risk behaviors of African American adolescents. J Adolesc Health. 2010;47:12-9.
419. Tabet SR, Krone MR, Paradise MA, Corey L, Stamm WE, Celum CL. Incidence of HIV and sexually transmitted diseases (STD) in a cohort of HIV-negative men who have sex with men (MSM). AIDS. 1998;12:2041-8.
420. Tan WS, Chio TW. Which partner notification method do patients prefer? Results of a patient preference survey at the national STI clinic in Singapore. Sex Transm Infect. 2015;91:A96-7.
421. Tao G, Walsh CM, Anderson LA, Irwin KL. Understanding sexual activity defined in the HEDIS measure of screening young women for Chlamydia trachomatis. Jt Comm J Qual Improv. 2002;28:435-40.
422. Tebb K, Shafer MA. A clinical practice intervention to increase chlamydial screening: Sustaining the gain and translating into practice 4 years later. Sex Transm Infect. 2011;87:A321-2.
423. Tebb KP, Paukku MH, Pai-Dhungat MR, Gyamfi AA, Shafer MA. Home STI testing: the adolescent female's opinion. J Adolesc Health. 2004;35:462-7.
424. Tebb KP, Shafer MA, Wibbelsman CJ, Pecson S, Tipton AC, Neuhaus JM, et al. To screen or not to screen: Prevalence of C. trachomatis among sexually active asymptomatic male adolescents attending health maintenance pediatric visits. J Adolesc Health. 2004;34:166-8.
425. Thomas M. Chlamydia testing within family planning services: An audit of compliance with policies. Br J Fam Plann. 1997;23:92-5.
426. Thorsteinsson K, Ladelund S, Storgaard M, Ronsholt FF, Johansen IS, Pedersen G, et al. Sexually transmitted infections and use of contraceptives in women living with HIV in Denmark - the SHADE cohort. BMC Infect Dis. 2016;16:81.
427. Tibbits M, Maloney S, Ndashe TP, Grimm B, Johansson P, Siahpush M. Impact of the Community-Wide Adolescent Health Project on Sexually Transmitted Infection Testing in Omaha, Nebraska. Am J Public Health. 2018;108:782-4.
428. Tingey L, Strom R, Hastings R, Parker A, Barlow A, Rompalo A, et al. Self-administered sample collection for screening of sexually transmitted infection among reservation-based American Indian youth. Int J STD AIDS. 2015;26:661-6.
429. Tobin C, Aggarwal R, Clarke J, Chown R, King D. Chlamydia trachomatis: Opportunistic screening in primary care. Br J Gen Pract. 2001;51:565-6.
430. Tobin JM, Harindra V, Mani R. Which treatment for genital tract Chlamydia trachomatis infection? Int J STD AIDS. 2004;15:737-9.
431. Tobin K, Edwards C, Flath N, Lee A, Tormohlen K, Gaydos CA. Acceptability and feasibility of a Peer Mentor program to train young Black men who have sex with men to promote HIV and STI home-testing to their social network members. AIDS Care. 2018;30:896-902.
432. Toby M, White J, De Ruiter A, Chilton D. A retrospective analysis of HIV-positive patients diagnosed with sexually transmitted infection (STI) on asymptomatic screening: the risk to public health. HIV Med. 2011;12(Suppl 1):6.
433. Todd CS, Haase C, Stoner BP. Emergency department screening for asymptomatic sexually transmitted infections. Am J Public Health. 2001;91:461-4.
434. Tomnay JE, Coelli L, Hocking JS. High rates of chlamydia found among 12- to 16-year-olds attending a rural sexual health clinic: implications for practice. Sex Health. 2016;13:193-5.
435. Torrone E, Papp J, Weinstock H. Prevalence of Chlamydia trachomatis genital infection among persons aged 14-39 years--United States, 2007-2012. MMWR Morb Mortal Wkly Rep. 2014;63:834-8.
436. Touboul Lundgren P, Detanne S, Dunais B, Bruno P, Bentz L, Khouri P, et al. Promoting primary care screening for Chlamydia trachomatis infection. Sante Publique. 2016;28:299-308.
437. Town K, McNulty CA, Ricketts EJ, Hartney T, Nardone A, Folkard KA, et al. Service evaluation of an educational intervention to improve sexual health services in primary care implemented using a step-wedge design: analysis of chlamydia testing and diagnosis rate changes. BMC Public Health. 2016;16:686.
438. Town K, Ricketts EJ, Hartney T, Dunbar JK, Nardone A, Folkard KA, et al. Supporting general practices to provide sexual and reproductive health services: protocol for the 3Cs & HIV programme. Public Health. 2015;129:1244-50.
439. Tozzini R, Cipulli G, Abad IR, Copari C, Sutich E, Sera G, et al. Detection of Chlamydia trachomatis (C.t.) and postreatment control in women with CDC screening criteria. 10th World Congress of Cervical Pathology and Colposcopy; 1999 Nov 7-11; Buenos Aires, AR. Bologna: Monduzzi editore, International Proceedings Division; 1999.
440. Truong HH, Kellogg T, Klausner JD, Katz MH, Dilley J, Knapper K, et al. Increases in sexually transmitted infections and sexual risk behaviour without a concurrent increase in HIV incidence among men who have sex with men in San Francisco: A suggestion of HIV serosorting? Sex Transm Infect. 2006;82:461-6.
441. Turley M, McNicholas A, Nesdale A, Bennett S, Garrett N. Sexually transmitted infections at New Zealand sexual health clinics, 1999. N Z Public Health Rep. 2000;7:49-52.
442. Turner CF, Rogers SM, Miller HG, Miller WC, Gribble JN, Chromy JR, et al. Untreated gonococcal and chlamydial infection in a probability sample of adults. JAMA. 2002;287:726-33.
443. Turok DK, Eisenberg DL, Teal SB, Keder LM, Creinin MD. A prospective assessment of pelvic infection risk following same-day sexually transmitted infection testing and levonorgestrel intrauterine system placement. Am J Obstet Gynecol. 2016;215:599.E1-E6.
444. Uddin Z. Impact of an internet based sexually transmitted infections testing program in Ottawa, Canada. Results from the "Get Tested. Why not?" Campaign, a first of its kind model to increase access to testing. Can J Infect Dis Med Microbiol. 2014;25:23A.
445. Uhrig JD, Friedman A, Poehlman J, Scales M, Forsythe A. Knowledge, beliefs and behaviours related to STD risk, prevention, and screening among a sample of African American men and women. Health Educ J. 2014;73:332-40.
446. University Hospital Inselspital Berne. Prevalence of Sexually Transmitted Infections (STIs) in HIV-infected Patients (CTNG). 2009. ClinicalTrials.gov registration number: NCT00973466.
447. University of California San Diego. Texting Intervention to Sustain HIV Prevention in Women in High-drug-use Contexts. 2015. ClinicalTrials.gov registration number: NCT02447484.
448. University of California San Francisco. Leveraging Technology as a Clinician Extender to Screen Culturally Diverse Young Women for Chlamydia. 2010. ClinicalTrials.gov registration number: NCT01140022.
449. University of Washington. Washington State Community Expedited Partner Treatment (EPT) Trial. 2012. ClinicalTrials.gov registration number: NCT01665690.
450. Unknown. Concerns About Privacy May Prevent Some Youth From Getting STI Tests. Contracept Technol Update. 2017;38:12-3.
451. Valway S, Jenison S, Keller N, Vega-Hernandez J, Hubbard McCree D. Risk assessment and screening for sexually transmitted infections, HIV, and hepatitis virus among long-distance truck drivers in New Mexico, 2004-2006. Am J Public Health. 2009;99:2063-8.
452. van Bergen JE, Postma MJ, Peerbooms PG, Spangenberg AC, Tjen ATJ, Bindels PJ. Effectiveness and cost-effectiveness of a pharmacy-based screening programme for Chlamydia trachomatis in a high-risk health centre population in Amsterdam using mailed home-collected urine samples. Int J STD AIDS. 2004;15:797-802.
453. van Bergen JE, Spaargaren J, Gotz HM, Veldhuijzen IK, Bindels PJ, Coenen TJ, et al. Population prevalence of Chlamydia trachomatis and Neisseria gonorrhoeae in the Netherlands. Should asymptomatic persons be tested during population-based Chlamydia screening also for gonorrhoea or only if chlamydial infection is found? BMC Infect Dis. 2006;6:42.
454. van Liere G, Dukers-Muijrers N, Levels L, Hoebe C. High Proportion of Anorectal Chlamydia trachomatis and Neisseria gonorrhoeae After Routine Universal Urogenital and Anorectal Screening in Women Visiting the Sexually Transmitted Infection Clinic. Clin Infect Dis. 2017;64:1705-10.
455. van Liere GA, Dukers-Muijrers NH, van Bergen JE, Gotz HM, Stals F, Hoebe CJ. The added value of chlamydia screening between 2008-2010 in reaching young people in addition to chlamydia testing in regular care; an observational study. BMC Infect Dis. 2014;14:612.
456. Van Liere GA, Dukers-Muijrers NH, Wolffs PF, Hoebe CJ. Substantial natural clearance of genital and extragenital chlamydia trachomatis and Neisseria Gonorrhoeae in STD clinic attendees. Sex Transm Infect. 2013;89(Suppl 1):A206.
457. Van Liere GA, Hoebe CJ, Dukers-Muijrers NH. Anatomic site distribution of sexually transmitted diseases in men who have sex with men and high risk females by routine testing, including anorectal and oropharyngeal testing. Sex Transm Infect. 2013;89(Suppl 1):A195-6.
458. Van Rooijen MS, Schim Van Der Loeff MF, Van Dam AP, Speksnijder AG, De Vries HJ. High persistence of pharyngeal chlamydia in high risk visitors at the STI clinic, Amsterdam. Ned Tijdschr Dermatol Venereol. 2013;23:49.
459. Van Rooijen MS, Vriens P, Gotz H, Heijman T, Voeten H, Koekenbier R. Acceptance of an online partner notification tool for STI, Called Suggest-A-Test. Sex Transm Infect. 2013;89(Suppl 1):A335.
460. Vanrolleghem A, Van Soest EM, Van Den Broek I, Sturkenboom MCJM. Incidence of chlamydia diagnoses, tests, and the use of antibiotics. Pharmacoepidemiol Drug Saf. 2009;18(Suppl 1):S55.
461. Vanwesenbeeck I, Bakker F, Gesell S. Sexual health in the Netherlands: Main results of a population survey among Dutch adults. Int J Sex Health. 2010;22:55-71.
462. Ventimiglia E, Pederzoli F, Capogrosso P, Cazzaniga W, Boeri L, Alfano M, et al. When to perform semen culture in asymptomatic infertile men? Hints from a cross sectional study. Euro Urol. 2018;17:E1097.
463. Ventimiglia E, Pederzoli F, Capogrosso P, Cazzaniga W, Frego N, Chierigo F, et al. When to perform semen culture in asymptomatic infertile men? hints from a cross sectional study. J Urol. 2018;199(Suppl 1):E794-5.
464. Venzon DK, Molinari C, Hendryx M, Ahern M. Will high-risk STD clinic clients use home HIV test kits? Am J Health Behav. 1998;22:283-91.
465. Wade AJ, Hocking JS, Hellard ME. Chlamydia trachomatis prevalence in heterosexual men in Melbourne: a community-based study. Sex Health. 2007;4:137-8.
466. Walker J, Walker S, Fairley CK, Gunn J, Pirotta M, Gurrin L, et al. Computer reminders for chlamydia screening in general practice: A randomised controlled trial. Sex Health. 2009;6:363.
467. Walter J, Wiggins H, Meade R, Hill S. If it's good for the goose it's good for the gander: Are we missing rectal Chlamydia trachomatis (CT) infection in women by performing selective screening? HIV Med. 2014;15(Suppl 3):104.
468. Ward J, Guy RJ, Rumbold AR, McGregor S, Wand H, McManus H, et al. Strategies to improve control of sexually transmissible infections in remote Australian Aboriginal communities: a stepped-wedge, cluster-randomised trial. Lancet Glob Health. 2019;7:E1553-63.
469. Warner L, Klausner JD, Rietmeijer CA, Malotte CK, O'Donnell L, Margolis AD, et al. Effect of a brief video intervention on incident infection among patients attending sexually transmitted disease clinics. PLoS Med. 2008;5:E135.
470. Washington R, Whitaker A. Intrauterine device use in an inner city university clinic: A retrospective analysis. Contraception. 2011;84:318.
471. Watson J, Carlile J, Dunn A, Evans M, Fratto E, Hartsell J, et al. Increased Gonorrhea Cases - Utah, 2009-2014. MMWR Morb Mortal Wkly Rep. 2016;65:889-93.
472. Watson MC, Flett G. Chlamydia testing: A prospective study of delivery in general practice, community pharmacy and non-health community sites. Int J Pharm Pract. 2009;17:B10-1.
473. Watson PG. Using a national guideline: an audit of the management of gonorrhoea in Newcastle upon Tyne. Int J STD AIDS. 2000;11:677-81.
474. Watson V, Ryan M, Watson E. Valuing experience factors in the provision of Chlamydia screening: an application to women attending the family planning clinic. Value Health. 2009;12:621-3.
475. Weber RA. Factors associated with sexually transmitted infections including the human immunodeficiency virus among sex workers in Moscow, Russian Federation and Israel. PhD [dissertation]. Maryland: Johns Hopkins University; 2009. Available from: Dissertation Abstracts International: Section B: The Sciences and Engineering.
476. Weston E, Kreisel K, Torrone E. The curious case of rates of gonorrhea and chlamydia among adolescents (15-19 years) in the United States, 2011-2015. Sex Transm Dis. 2016;43(Suppl 2):S163.
477. Whitlock G, Duke O, Nwokolo N, McOwan A. Active recall of high-risk MSM by text message. Sex Transm Infect. 2015;91:A72.
478. Widdice LE, Hsieh YH, Silver B, Barnes M, Barnes P, Gaydos CA. Performance of the Atlas rapid test for Chlamydia trachomatis and women's attitudes toward point-of-care testing. Sex Transm Dis. 2018;45:723-7.
479. Widdice LE, Owens D, Silver B, Barnes M, Barnes P, Dize L, et al. Performance of a Rapid, Point-of-Care Test for Chlamydia Trachomatis and Women’s Attitudes Towards Rapid Testing. J Adolesc Health. 2017;60(Suppl 1):S117.
480. Wiest DR, Spear SJ, Bartfield JM. Empiric treatment of gonorrhea and chlamydia in the ED. Am J Emerg Med. 2001;19:274-5.
481. Wilcox MH, Reynolds MT, Hoy CM, Brayson J. Combined cervical swab and urine specimens for PCR diagnosis of genital Chlamydia trachomatis infection. Sex Transm Infect. 2000;76:177-8.
482. Wilkins A, Mak DB. . . . Sending out an SMS: an impact and outcome evaluation of the Western Australian Department of Health's 2005 chlamydia campaign. Health Promot J Austr. 2007;18:113-20.
483. Wilkowska-Trojniel M, Zdrodowska-Stefanow B, Ostaszewska-Puchalska I, Zbucka M, Wolczynski S, Grygoruk C, et al. Chlamydia trachomatis urogenital infection in women with infertility. Adv Med Sci. 2009;54:82-5.
484. Willers DM, Peipert JF, Allsworth JE, Stein MD, Rose JS, Clarke JG. Prevalence and predictors of sexually transmitted infection among newly incarcerated females. Sex Transm Dis. 2008;35:68-72.
485. Williams SP, Myles RL, Sperling CC, Carey D. An intervention for reducing the sexual risk of men released from jails. J Correct Health Care. 2018;24:71-83.
486. Willis LA, Kachur R, Castellanos TJ, Nichols K, Mendoza MCB, Gaul ZJ, et al. Developing a Motion Comic for HIV/STD Prevention for Young People Ages 15-24, Part 2: Evaluation of a Pilot Intervention. Health Commun. 2018;33:229-37.
487. Wingood GM, Diclemente RJ, Robinson-Simpson L, Lang DL, Caliendo A, Hardin JW. Efficacy of an HIV intervention in reducing high-risk human papillomavirus, nonviral sexually transmitted infections, and concurrency among African American women: a randomized-controlled trial. J Acquir Immune Defic Syndr. 2013;63(Suppl 1):S36-43.
488. Winzor G, Habib A. An audit of treatment and follow-up of patients diagnosed with genital gonorrhoea in south warwickshire gum departmentcategory: Lesson in microbiology & infection control. J Infect. 2011;63:E45-6.
489. Wolfers M, de Zwart O, Kok G. Adolescents in The Netherlands Underestimate Risk for Sexually Transmitted Infections and Deny the Need for Sexually Transmitted Infection Testing. AIDS Patient Care STDS. 2011;25:311-9.
490. Wolfers M, Kok G, Looman C, de Zwart O, Mackenbach J. Promoting STI testing among senior vocational students in Rotterdam, the Netherlands: effects of a cluster randomized study. BMC Public Health. 2011;11:937.
491. Wolfers ME, Kok G, Mackenbach JP, de Zwart O. Correlates of STI testing among vocational school students in the Netherlands. BMC Public Health. 2010;10:725.
492. Wolitski R, Parsons J, Gómez C, Purcell D, Hoff C, Halkitis P. Prevention with gay and bisexual men living with HIV: rationale and methods of the Seropositive Urban Men's Intervention Trial (SUMIT). AIDS. 2005;19(Suppl 1):S1-11.
493. Wombacher K, Dai M, Matig JJ, Harrington NG. Using the integrative model of behavioral prediction to understand college students' STI testing beliefs, intentions, and behaviors. J Am Coll Health. 2018:1-8.
494. Wong ML, Chan RK, Koh D, Wee S. Increase in oral sex and pharyngeal gonorrhoea: An unintended effect of a successful condom promotion programme for vaginal sex. AIDS. 1999;13:1981-2.
495. Woodhall SC, Buitendam E, Town K, Baraitser P, McNeil F, Clarke J, et al. To re-test or not to re-test? Findings from the english national chlamydia screening programme consultation on routine re-testing following a chlamydia diagnosis. Sex Transm Dis. 2014;41:S59-60.
496. Woodhall SC, Wills G, Horner P, Craig R, Mindell JS, Murphy G, et al. Insights into chlamydia trachomatis cumulative incidence in the context of widespread opportunistic chlamydia screening in england: Seroprevalence study using sera from a nationally-representative household survey. Sex Transm Infect. 2015;91(Suppl 2):A136.
497. Wouters K, Van Damme P, Vercauteren A, Verheyen J, Castermans S, Meheus A. Sexually transmitted infections (STI) among prostitutes in Antwerp, Belgium. Importance and feasibility of a hepatitis B vaccination programme. Arch Public Health. 2002;60:27-38.
498. Wu J. HIV/STIs related risk among middle aged and old MSM in Shenzhen, China. PhD [dissertation]. Los Angelas: University of California; 2017. Available from: Dissertation Abstracts International: Section B: The Sciences and Engineering.
499. Yarber WL, Torabi MR. Impact of a theory-based, school HIV/STD curriculum on eighth graders' attitudes and knowledge. J Health Educ. 1997;28:74-84.
500. Yeow TC, Wong WF, Sabet NS, Sulaiman S, Shahhosseini F, Tan GM, et al. Prevalence of plasmid-bearing and plasmid-free Chlamydia trachomatis infection among women who visited obstetrics and gynecology clinics in Malaysia. BMC Microbiol. 2016;16:45.
501. Young MK, McCall BJ, Jardine D. Two years of enhanced surveillance of sexually-transmitted chlamydia in South East Queensland. Commun Dis Intell Q Rep. 2006;30:456-61.
502. Yuguero O, Casanova JM, Manonelles A, Godoy P. Detection of Chlamydia trachomatis infection in patients seen at a sexually transmitted infection clinic. Actas Dermosifiliogr. 2015;106:235-8.
503. Zenner D, Molinar D, Nichols T, Riha J, Macintosh M, Nardone A. Should young people be paid for getting tested? A national comparative study to evaluate patient financial incentives for chlamydia screening. BMC Public Health. 2012;12:261.
504. Zhang Q, Huhn KJ, Tan A, Douglas RE, Li HG, Murti M, et al. "Testing is Healthy" TimePlay campaign: Evaluation of sexual health promotion gamification intervention targeting young adults. Can J Public Health. 2017;108:E85-90.
505. Zou H, Fairley CK, Guy R, Bilardi J, Bradshaw CS, Garland SM, et al. Automated, computer generated reminders and increased detection of gonorrhoea, chlamydia and syphilis in men who have sex with men. PLoS ONE. 2013;8:E61972.
506. Zou H, Meng X, Grulich A, Huang S, Jia T, Zhang X, et al. A randomised controlled trial to evaluate the impact of sexual health clinic based automated text message reminders on testing of HIV and other sexually transmitted infections in men who have sex with men in China: protocol for the T2T Study. BMJ Open. 2017;7:E015787.

**NO COMPARATOR**

1. Aghaizu A, Reid F, Kerry S, Hay PE, Mallinson H, Jensen JS, et al. Frequency and risk factors for incident and redetected Chlamydia trachomatis infection in sexually active, young, multi-ethnic women: a community based cohort study. Sex Transm Infect. 2014;90:524-8.
2. Ako MC, Lewis M, Peterson S, Gaydos CA, Rothman R, Dugas A. The clinical impact of rapid diagnostics on improving appropriate treatment of STIs in women in the emergency department. Sex Transm Dis. 2016;43 (Suppl 2):S136.
3. Anschuetz GL, Asbel L, Spain CV, Salmon M, Lewis F, Newbern EC, et al. Association between enhanced screening for Chlamydia trachomatis and Neisseria gonorrhoeae and reductions in sequelae among women. J Adolesc Health. 2012;51:80-5.
4. Apostolou A, McCollum J, Person M. Using electronic health records to examine rates of chlamydia among American indians and Alaska natives. Sex Transm Dis. 2016;43(Suppl 2):S163-4.
5. Atkinson LM, Vijeratnam D, Mani R, Patel R. 'The waiting game': are current chlamydia and gonorrhoea near-patient/point-of-care tests acceptable to service users and will they impact on treatment? Int J STD AIDS. 2016;27:650-5.
6. Auerswald CL, Sugano E, Ellen JM, Klausner JD. Street-based STD testing and treatment of homeless youth are feasible, acceptable and effective. J Adolesc Health. 2006;38:208-12.
7. Badolato G, Goyal MK. STI Screening In An Urban Emergency Department Based On Chief Complaint. J Adolesc Health. 2019;64 (Suppl 2):S133-4.
8. Bailey JV, Pavlou M, Copas A, McCarthy O, Carswell K, Rait G, et al. The Sexunzipped trial: optimizing the design of online randomized controlled trials. J Med Internet Res. 2013;15:E278.
9. Bakken IJ, Skjeldestad FE, Halvorsen TF. Norwegian men diagnosed with genital Chlamydia trachomatis infection notified two-thirds of their sexual partners. Scand J Infect Dis. 2008;40:275-8.
10. Balendra A, Cousins E, Lamplough H, Oakeshott P, Majewska W, Kerry SR. Pilot study for the 'Test n Treat' trial of on-site rapid chlamydia/gonorrhoea tests and same day treatment. Sex Transm Infect. 2017;93:283.
11. Barry PM, Kent CK, Scott KC, Snell A, Goldenson J, Klausner JD. Optimising sexually transmitted infection screening in correctional facilities: San Francisco, 2003-2005. Sex Transm Infect. 2007;83:416-8.
12. Beanland F, Schoeman S, Davis P, McCusker P, Doyle T. A year of 'sex, steam and stis'. Sex Transm Infect. 2015;91:A91.
13. Bernstein KT, Stephens S, Torrone E, Chow J, Philip S. Can chlamydia prevalence monitoring data be used to evaluate impact of screening? The US CDC infertility prevention project experience. Sex Transm Infect. 2013;89(Suppl 1):A247.
14. Berry SA, Ghanem KG, Page KR, Gange SJ, Thio CL, Moore RD, et al. Increased gonorrhoea and chlamydia testing did not increase case detection in an HIV clinical cohort 1999-2007. Sex Transm Infect. 2011;87:469-75.
15. Breslin KA, Tuchman L, Hayes KL, Goyal M. Notification and treatment of positive sexually transmitted infection test results in a pediatric emergency department. J Investig Med. 2014;62:775.
16. Breslin KA, Tuchman L, Hayes KL, Goyal M. Sensitivity and specificity of empiric treatment for sexually transmitted infections in the pediatric emergency department. J Investig Med. 2014;62:760-1.
17. Brown CK, Earley M, Shaikh R, Fickenscher J, Ott J, Person A, et al. Voluntary STD testing and treatment program at a metropolitan correctional facility: evaluation of test acceptability and associated risk factors. J Correct Health Care. 2014;20:70-80.
18. Buhrer-Skinner M, Muller R, Menon A, Gordon R. Novel approach to an effective community-based chlamydia screening program within the routine operation of a primary healthcare service. Sex Health. 2009;6:51-6.
19. Bull SS, Jones CA, Granberry-Owens D, Stoner BP, Rietmeijer CA. Acceptability and feasibility of urine screening for Chlamydia and gonorrhea in community organizations: perspectives from Denver and St Louis. Am J Public Health. 2000;90:285-6.
20. Burstein GR, Waterfield G, Joffe A, Zenilman JM, Quinn TC, Gaydos CA. Screening for gonorrhea and chlamydia by DNA amplification in adolescents attending middle school health centers. Opportunity for early intervention. Sex Transm Dis. 1998;25:395-402.
21. Campos PE, Buffardi AL, Carcamo CP, Garcia PJ, Buendia C, Chiappe M, et al. Reaching the unreachable: providing STI control services to female sex workers via mobile team outreach. PLoS ONE. 2013;8:E81041.
22. Cassell JA, Brook MG, Mercer CH, Murphy S, Johnson AM. Maintaining patient access to GUM clinics: Is it compatible with appointments? Sex Transm Infect. 2003;79:11-5.
23. Centers for Disease Control and Prevention. Chlamydia trachomatis genital infections--United States, 1995. MMWR Morb Mortal Wkly Rep. 1997;46:193-8.
24. Centers for Disease Control and Prevention. Chlamydia trachomatis genital infections--United States, 1995. Can Commun Dis Rep Wkly. 1998;24:5-8.
25. Chacko MR, Wiemann CM, Kozinetz CA, Diclemente RJ, Smith PB, Velasquez MM, et al. New sexual partners and readiness to seek screening for chlamydia and gonorrhoea: predictors among minority young women. Sex Transm Infect. 2006;82:75-9.
26. Cheaveau J, Manavi K. Does PEPSE reduce high-risk sexual activity? A comparison of the rates of sexually transmitted infections at the time of PEPSE and at subsequent screening. HIV Med. 2016;17(Suppl 1):70-1.
27. Chernesky MA, Hook EW 3rd, Martin DH, Lane J, Johnson R, Jordan JA, et al. Women find it easy and prefer to collect their own vaginal swabs to diagnose Chlamydia trachomatis or Neisseria gonorrhoeae infections. Sex Transm Dis. 2005;32:729-33.
28. Chernesky MA, Jang D, Portillo E, Smieja M, Gilchrist J, Ewert R, et al. Self-collected swabs of the urinary meatus diagnose more Chlamydia trachomatis and Neisseria gonorrhoeae infections than first catch urine from men. Sex Transm Infect. 2013;89:102-4.
29. Cohen DA, Nsuami M, Etame RB, Tropez-Sims S, Abdalian S, Farley TA, et al. A school-based Chlamydia control program using DNA amplification technology. Pediatrics. 1998;101:E1.
30. Cohen SE, Vittinghoff E, Philip SS, Elion R, Kolber MA, Liu AY. Repeat rectal gonorrhea and chlamydia infections in a cohort of participants on prep. Sex Transm Dis. 2016;43(Suppl 2):S177.
31. Cole J, Hotton A, Zawitz C, Kessler H. Opt-out screening for Chlamydia trachomatis and Neisseria gonorrhoeae in female detainees at Cook County jail in Chicago, IL. Sex Transm Dis. 2014;41:161-5.
32. Copeland ER, Henry-Reid LM, Hotton AL, Anaene M, Martinez J. Incident and prevalent sexually transmitted infections after diagnosis and engagement in care in HIV positive youth in an urban care setting. J Adolesc Health. 2014;54:S59.
33. Cowan E, Zahn JA, Nagel F, Calderon Y, Herman H, Wood E, et al. Implementation of a chlamydia and gonorrhea screening program in an urban emergency department setting. Acad Emerg Med. 2017;24(Suppl 1):S273.
34. Cucinella A, Priestley CJF. Audit of the use of microscopy in heterosexual male patients diagnosed with non-specific urethritis and chlamydial urethritis. Sex Transm Infect. 2012;88(Suppl 1):A58.
35. Davison T. Chlamydia screening in a primary care setting. Prim Health Care. 2013;23:16-21.
36. De Vrieze NH, Mooij SH, De Vries HJ, Van Rooijen M, Heijman T, Van Eeden A, et al. Sexually transmitted infections in HIV-infected men who have sex with men (MSM), can we predict a new episode of STI? Ned Tijdschr Dermatol Venereol. 2014;24:51-2.
37. den Heijer CD, Hoebe CJ, van Liere GA, van Bergen JE, Cals JW, Stals FS, et al. A comprehensive overview of urogenital, anorectal and oropharyngeal Neisseria gonorrhoeae testing and diagnoses among different STI care providers: a cross-sectional study. BMC Infect Dis. 2017;17:1-10.
38. Dooley S, Delamere S, Caroline H, O'Dea S, Mulcahy F. Self-taking sexually transmitted infection screening in asymptomatic men who have sex with men in an HIV clinic in Dublin - Outcomes and acceptability. Int J STD AIDS. 2015;26:55.
39. Dudareva-Vizule S, Alt K, Hofmann A, Jansen K, Sailer A, Haar K, et al. Chlamydia trachomatis infection in men in Germany, 2008-2014. Int J STD AIDS. 2015;26:72.
40. Dukers-Muijrers NH, van Liere GA, Hoebe CJ. Re-screening Chlamydia trachomatis positive subjects: a comparison of practices between an STI clinic, general practitioners and gynaecologists. Sex Transm Infect. 2013;89:25-7.
41. Dunville R, Peterson A, Liddon N, Roach M, Coleman K, Dittus P. Sustained Reduction in Chlamydia Infections Following a School-Based Screening: Detroit, 2010–2015. Am J Public Health. 2018;108:231-3.
42. Fagan P, Cannon F, Crouch A. The Young Person Check: screening for sexually transmitted infections and chronic disease risk in remote Aboriginal and Torres Strait Islander youth. Aust N Z J Public Health. 2013;37:316-21.
43. Fernando K, Flew S, Phattey J, Harding J, Fowler T, Caley M, et al. Systematic recall vs standard care, addressing the increased risk of re-infection in patients presenting with gonorrhoea. Sex Transm Infect. 2012;88(Suppl 1):A25-6.
44. Fortenberry JD, Brizendine EJ, Katz BP, Wools KK, Blythe MJ, Orr DP. Subsequent sexually transmitted infections among adolescent women with genital infection due to Chlamydia trachomatis, Neisseria gonorrhoeae, or Trichomonas vaginalis. Sex Transm Dis. 1999;26:26-32.
45. Foster R, Ali H, Crowley M, Dyer R, Grant K, Lenton J, et al. Does Living Outside of a Major City Impact on the Timeliness of Chlamydia Treatment? A Multicenter Cross-Sectional Analysis. Sex Transm Dis. 2016;43:506-12.
46. Foster S, Wolf S, Cox C, Tunacao J. The point in point of care (POC) testing for sexually transmitted infections (STIS). Int J Gynecol Obstet. 2015;131(Suppl 5):E211.
47. Garton L, Dyda A, Guy R, Silver B, McGregor S, Hengel B, et al. High chlamydia and gonorrhoea repeat positivity in remote Aboriginal communities 2009-2011: longitudinal analysis of testing for re-infection at 3 months suggests the need for more frequent screening. Sex Health. 2016;13:568-74.
48. Ghanem M, Cousins D, Migliorini D, Riddell L. Testing for rectal chlamydia and gonorrhea in women reported receptive anal intercourse. Int J STD AIDS. 2015;26:49-50.
49. Goings S, Chau D. Uptake and acceptability of STI screenings for transgender and gender non-binary individuals in a sexual health clinical setting. Sex Transm Dis. 2018;45 (Suppl 2):S27.
50. Goller JL, Fairley CK, Bradshaw CS, De Livera AM, Chen MY, Guy RJ, et al. Risk of pelvic inflammatory disease from chlamydia and gonorrhoea among Australian sexual health clinic attendees. Sex Transm Infect. 2015;91(Suppl 2):A139.
51. Gomes G, Palma F. Sexually transmitted infections-the experience of an adolescents' unit Marta Britoa, Alexandra Ruivo Coelhoa. Eur J Contracept Reprod Health Care. 2018;23(Suppl 1):42-3.
52. Gotz H, Lindback J, Ripa T, Arneborn M, Ramstedt K, Ekdahl K. Is the increase in notifications in chlamydia trachomatis infections in Sweden the result of changes in prevalence, sampling frequency or diagnostic methods? Scand J Infect Dis. 2002;34:28-34.
53. Gotz HM, Hoebe CJ, Van Bergen JE, Brouwers EE, Op De Coul EL, Fennema JS, et al. High yield in reinfections during a chlamydia screening programme when automatically sending testkits after 6 months to previously infected. Sex Transm Infect. 2011;87(Suppl 1):A21-2.
54. Gunn RA, Podschun GD, Fitzgerald S, Hovell MF, Farshy CE, Black CM, et al. Screening high-risk adolescent males for Chlamydia trachomatis infection: Obtaining urine specimens in the field. Sex Transm Dis. 1998;25:49-52.
55. Gutfreund D, Thakker U, Kopecky A, Sivitz A. Asymptomatic gonorrhea and chlamydia screening within the pediatric emergency department of a disease-prevalent population. Acad Emerg Med. 2013;20(Suppl 1):S309.
56. Haidari G, Perry ME, White JA. Are we seeing a true rise in Neisseria Gonorrhoeae and chlamydia trachomatis in England in Men Who Have Sex with Men? Sex Transm Infect. 2013;89(Suppl 1):A185.
57. Haidari G, Stockwell S, Elgalib A, Surah S, Tong C, Alexander S, et al. Clinical features and treatment responses in 300 pharyngeal Chlamydia trachomatis infections in men who have sex with men. Int J STD AIDS. 2013;41:32.
58. Hassan SJ, Dunphy E, Navin E, Marron L, Fitzsimmons C, Loy A, et al. Screening for Chlamydia is acceptable and feasible during Cervical Screening in General Practice. Ir Med J. 2016;109:326-7.
59. Hay PE, Kerry SR, Normansell R, Horner PJ, Reid F, Kerry SM, et al. Which sexually active young female students are most at risk of pelvic inflammatory disease? A prospective study. Sex Transm Infect. 2016;92:63-6.
60. Hiltunen-Back E, Kautiainen H. Increasing sexually transmitted infection rates among men who have sex with men in Finland 2004-2014. Int J STD AIDS. 2015;26:75.
61. Huang R, Ward J, Tangey A, Causer L, Guy R. New molecular point-of-care test improves timeliness of treatment for chlamydia trachomatis (CT) and neisseria gonorrhoea (Ng) in a remote aboriginal health clinic. Sex Transm Infect. 2015;91:A121-A2.
62. Hunjan T, Kerry SR, Normansell R, Hay PE, Sadiq ST, Planche T, et al. Chlamydia testing: where are we now? Recruiting high-risk women to a pilot STI screening trial. Sex Transm Infect. 2013;89:556.
63. James NJ, Wilson S, Hughes S. A pilot study to incorporate chlamydial testing in the management of women anticipating IUD insertion in community clinics. Br J Fam Plann. 1997;23:16-9.
64. Javanbakht M, Boudov M, Anderson LJ, Malek M, Smith LV, Chien M, et al. Sexually Transmitted Infections Among Incarcerated Women: Findings From a Decade of Screening in a Los Angeles County Jail, 2002–2012. Am J Public Health. 2014;104:E103-9.
65. Jennings AL, Theriot JA, Franco SM. Gonorrhea and chlamydia screening: Who should we screen? J Investig Med. 2013;61:453.
66. Jessen H, Lenz J, Jessen A, Stein L, Zedlack C, Hechler D, et al. Asymptomatic gonococcal and chlamydia infections in HIV-negative and HIV-positive men having sex with men. HIV Med. 2009;10(Suppl 2):18-9.
67. Jin F, Prestage GP, Imrie J, Kippax SC, Donovan B, Templeton DJ, et al. Anal sexually transmitted infections and risk of HIV infection in homosexual men. J Acquir Immune Defic Syndr. 2010;53:144-9.
68. Jin F, Prestage GP, Mao L, Kippax SC, Pell CM, Donovan B, et al. Incidence and risk factors for urethral and anal gonorrhoea and chlamydia in a cohort of HIV-negative homosexual men: the Health in Men Study. Sex Transm Infect. 2007;83:113-9.
69. Joesoef MR, Mosure DJ. Prevalence of chlamydia in young men in the United States from newly implemented universal screening in a national job training program. Sex Transm Dis. 2006;33:636-9.
70. Kaithampillai CJ, Varma R. Do Saturday sexual health clinics in North East Essex attract greater rates of chlamydia in young people? HIV Med. 2010;11(Suppl 1):98.
71. Kawsar M, Richards R. Impact of National Chlamydia Screening Programme on sexual health of children under the age of 16 years. Int J STD AIDS. 2008;19:51-2.
72. Kelley CF, Vaughan AS, Luisi N, Sanchez T, Frew P, Del Rio C, et al. The effect of sexually transmitted infections on HIV incidence among MSM in Atlanta, GA. Top Antivir Med. 2014;22:544.
73. Kent CK, Branzuela A, Fischer L, Bascom T, Klausner JD. Chlamydia and gonorrhea screening in San Francisco high schools. Sex Transm Dis. 2002;29:373-5.
74. Kerrigan D, Moreno L, Rosario S, Gomez B, Jerez H, Barrington C, et al. Environmental-structural interventions to reduce HIV/STI risk among female sex workers in the Dominican Republic. Am J Public Health. 2006;96:120-5.
75. Kerry SR, Nightingale CM, Hay P, Oakeshott P. Which sexually active female students get themselves tested for Chlamydia trachomatis? A cohort study. Int J STD AIDS. 2016;27:586-90.
76. Khan A, Dalton M. Chlamydia screening in gynaecology. Int J Gynecol Obstet. 2009;107(Suppl 2):S566-7.
77. Khan A, Fortenberry JD, Juliar BE, Tu W, Orr DP, Batteiger BE, et al. The prevalence of chlamydia, gonorrhea, and trichomonas in sexual partnerships: implications for partner notification and treatment. Sex Transm Dis. 2005;32:260-4.
78. Koutraki M, Valassidou E, Paparizos V, Grillias A, Frangouli E, Katsambas A, et al. Asymptomatic Chlamydia trachomatis infection in men who have sex with men (MSM) with HIV infection. Proceedings of the 15th Congress of the European Academy of Dermatology and Venereology; 2006 Oct 4-8; Rhodes, GR. Bologna: Medimond International Proceedings; 2006.
79. Kwan KSH, Jachimowicz EA, Bastian L, Marshall L, Mak DB. Online chlamydia testing: An innovative approach that appeals to young people. Med J Aust. 2012;197:287-90.
80. Lee VF, Tobin JM, Harindra V. Re-infection of Chlamydia trachomatis in patients presenting to the genitourinary medicine clinic in Portsmouth: the chlamydia screening pilot study - three years on. Int J STD AIDS. 2004;15:744-6.
81. Levitt MA, Johnson S, Engelstad L, Montana R, Stewart S. Clinical management of chlamydia and gonorrhea infection in a county teaching emergency department--concerns in overtreatment, undertreatment, and follow-up treatment success. J Emerg Med. 2003;25:7-11.
82. Lo B, Schott C, Best H, Visintainer C. Diagnosing and treating cervicitis in the emergency department: How good (or bad) are we? Acad Emerg Med. 2011;18(Suppl 1):S18.
83. Lolar SA, Sherwin RL, Robinson DM, Courage C, Welch RD. Effectiveness of an urban emergency department call-back system in the successful linkage to treatment of sexually transmitted infections. South Med J. 2015;108:268-73.
84. Ma S, Dukers NH, van den Hoek A, Yuliang F, Zhiheng C, Jiangting F, et al. Decreasing STD incidence and increasing condom use among Chinese sex workers following a short term intervention: a prospective cohort study. Sex Transm Infect. 2002;78:110-4.
85. Maraynes ME, Chao JH, Agoritsas K, Zehbtachi S. Screening for asymptomatic chlamydia and gonorrhea in young males in an urban emergency department. Acad Emerg Med. 2015;22(Suppl 1):S53.
86. Markham MR, Maggio L, Shah UR, Sangi-Haghpeykar H, Raine SP. Effects of routine screening for gonorrhea and chlamydia before intrauterine device insertion. Obstet Gynecol. 2014;123:11S.
87. Mayer K, Conron K, Crane H, Haubrich R, Geng E, Grasso C, et al. Low STD screening rates and high STD prevalence among HIV-infected patients in primary care in 5 US centers. Sex Transm Dis. 2014;41:S61.
88. McCarthy AE, Macdonald NE, Feder S, Doherty JA, McAvoy L, Toye B. Urine testing for Chlamydia trachomatis and hassle-free follow-up is acceptable to street youth. Paediatr Child Health. 1999;4:395-9.
89. McMillan S, Whitlock G, Day S, Allen K, Gilmour C, Jenkins J, et al. Targeted outreach: Does it work? HIV Med. 2014;15(Suppl 3):18.
90. Nett RJ, Choi P, Murolo C, Murphy JS. Notes from the field: increase in gonorrhea cases in counties associated with American Indian Reservations -- Montana, January 2012-August 2014. MMWR: Morb Mortal Wkly Rep. 2014;63:937.
91. Noone A, Spiers A, Allardice G, Carr S, Flett G, Brown A, et al. Opportunistic screening for genital Chlamydia trachomatis infection and partner follow-up in family planning clinics in three Scottish cities. J Fam Plann Reprod Health Care. 2004;30:84-5.
92. Nsuami MJ, Taylor SN, Smith BS, Martin DH. Increases in gonorrhea among high school students following hurricane Katrina. Sex Transm Infect. 2009;85:194-8.
93. O'Byrne P, MacPherson P, Ember A, Grayson M-O, Bourgault A. Overview of a gay men's STI/HIV testing clinic in Ottawa: Clinical operations and outcomes. Can J Public Health. 2014;105:E389-94.
94. Oakeshott P, Kerry S, Hay S, Hay P. Opportunistic screening for chlamydial infection at time of cervical smear testing in general practice: Prevalence study. BMJ. 1998;316:351-2.
95. Olson K, Galbraith J, Cain G, Geisler W. Missed opportunities to screen and treat chlamydia and gonorrhea in the emergency department setting. Sex Transm Dis. 2016;43(Suppl 2):S224-5.
96. Paparello J, Chilton D. Sexually transmitted infection (STI) screening in an inner London HIV outpatient unit: Positivity, partner notification (PN), and public health. HIV Med. 2014;15(Suppl 3):102.
97. Peterman TA, Tian LH, Metcalf CA, Satterwhite CL, Malotte CK, DeAugustine N, et al. High incidence of new sexually transmitted infections in the year following a sexually transmitted infection: A case for rescreening. Ann Intern Med. 2006;145:564-72.
98. Peterson A, Roach M, McMillan D. Participatory approach to increasing chlamydia screening rates in title X family planning clinics: A double digit improvement. Sex Transm Dis. 2018;45 (Supple 2):S47.
99. Pham TV, Mezzadra H, Holley C, Willis G, Witting M. Empiric treatment of gonorrhea and chlamydial infection in the ED: Are we overtreating? Acad Emerg Med. 2014;21(Suppl 1):S59-60.
100. Pimenta JM, Catchpole M, Rogers PA, Hopwood J, Randall S, Mallinson H, et al. Opportunistic screening for genital chlamydial infection. II: prevalence among healthcare attenders, outcome, and evaluation of positive cases. Sex Transm Infect. 2003;79:22-7.
101. Pittrof R, McLellan J. Test Not Talk screening for asymptomatic men. Int J STD AIDS. 2007;18:274-5.
102. Pomeroy L, Quinlan M, Enkelmann J, Clarke S. Is it NAAT time to change management of gonorrhoea contacts? HIV Med. 2014;15(Suppl 3):126-7.
103. Reichenbach S, Sanchez LD, Volz KA. Should empiric treatment of gonorrhea and chlamydia be used in the emergency department? Acad Emerg Med. 2012;19(Suppl 1):S126.
104. Rietmeijer CA, Yamaguchi KJ, Ortiz CG, Montstream SA, LeRoux T, Ehret JM, et al. Feasibility and yield of screening urine for Chlamydia trachomatis by polymerase chain reaction among high-risk male youth in field-based and other nonclinic settings. A new strategy for sexually transmitted disease control. Sex Transm Dis. 1997;24:429-35.
105. Risser JM, Risser WL, Gefter LR, Brandstetter DM, Cromwell PF. Implementation of a screening program for chlamydial infection in incarcerated adolescents. Sex Transm Dis. 2001;28:43-6.
106. Risser W, Risser J. Pelvic inflammatory disease (PID) in adolescents after treatment for cervicitis. Sex Transm Infect. 2011;87:A158.
107. Risser W, Risser J. Pelvic inflammatory disease occurring between the time of testing and treatment for gonorrhoea and chlamydia. Sex Transm Infect. 2011;87:A31.
108. Risser WL, Risser JM, Benjamins LJ. Pelvic inflammatory disease in adolescents between the time of testing and treatment and after treatment for gonorrhoeal and chlamydial infection. Int J STD AIDS. 2012;23:457-8.
109. Ritchie S, Henley R, Hilton J, Handy R, Ingram J, Mundt S, et al. Uptake, yield and resource requirements of screening for asymptomatic sexually transmissible infections among HIV-positive people attending a hospital outpatient clinic. Sex Health. 2014;11:67-72.
110. Rivard KR, Dumkow LE, Draper HM, Brandt KL, Whalen DW, Egwuatu NE. Impact of rapid diagnostic testing for chlamydia and gonorrhea on appropriate antimicrobial utilization in the emergency department. Diagn Microbiol Infect Dis. 2017;87:175-9.
111. Roberts C, Watson L, Turner R, Caverley-Frost L, Scott P, Allen K. Reaching the unreachable-nurse-led STI screening at erotica 2013. HIV Med. 2014;15(Suppl 3):27.
112. Rogers SM, Miller WC, Turner CF, Ellen J, Zenilman J, Rothman R, et al. Concordance of chlamydia trachomatis infections within sexual partnerships. Sex Transm Infect. 2008;84:23-8.
113. Rogers SM, Turner CF, Miller WC, Erbelding E, Eggleston E, Tan S, et al. Gender-based screening for chlamydial infection and divergent infection trends in men and women. PLoS ONE. 2014;9:E99374.
114. Ronda J, Gaydos C, Perin J, Tabacco L, Coleman J, Trent M. Does mycoplasma genitalium infection predict future sexually transmitted infections in female urban adolescents and young adults? J Adolesc Health. 2018;62(Suppl 1):S82.
115. Rose SB, Garrett SM, Stanley J, Pullon SR. Retesting and repeat positivity following diagnosis of Chlamydia trachomatis and Neisseria gonorrhoea in New Zealand: a retrospective cohort study. BMC Infect Dis. 2017;17:526.
116. Rose SB, Lawton BA, Bromhead C, Macdonald EJ, Lund KA. Self-obtained vaginal swabs for PCR chlamydia testing: a practical alternative. Aust N Z J Obstet Gynaecol. 2007;47:415-8.
117. Rosenberger JG, Dodge B, Van Der Pol B, Reece M, Herbenick D, Fortenberry JD. Reactions to self-sampling for ano-rectal sexually transmitted infections among men who have sex with men: a qualitative study. Arch Sex Behav. 2011;40:281-8.
118. Rotheram-Borus MJ, Wu Z, Liang LJ, Li L, Detels R, Guan J, et al. Reductions in sexually transmitted infections associated with popular opinion leaders in China in a randomised controlled trial. Sex Transm Infect. 2011;87:337-43.
119. Rukh S. Chlamydia and gonorrhea diagnosis, treatment, personnel cost savings, and service delivery improvements following the implementation of express STD testing in Maricopa County, Arizona. Sex Transm Dis. 2014;41(Suppl 1):S60-1.
120. Rukh S. Screening and treatment outcomes for female inmates in the maricopa county correctional facilities. Sex Transm Dis. 2016;43(Suppl 2):S195.
121. Rukh S, Khurana R, Mickey T, Anderson L, Velasquez C, Taylor M. Chlamydia and gonorrhea diagnosis, treatment, personnel cost savings, and service delivery improvements after the implementation of express sexually transmitted disease testing in Maricopa County, Arizona. Sex Transm Dis. 2014;41:74-8.
122. Santo I, Azevedo J, Nunes B, Gomes JP, Borrego MJ. Partner notification for chlamydia trachomatis urogenital infections: eight years of patient referral experience in the major Portuguese sexually transmitted infections clinic, 2000-07. Int J STD AIDS. 2011;22:548-51.
123. Satterwhite CL. Chlamydia surveillance in the United States: New analytic approaches and alternate considerations for monitoring trends in disease burden. PhD [dissertation]. Atlana: Emory University; 2011. Available from: Dissertation Abstracts International: Section B: The Sciences and Engineering.
124. Satterwhite CL, Grier L, Patzer R, Weinstock H, Howards PP, Kleinbaum D. Chlamydia positivity trends among women attending family planning clinics: United States, 2004-2008. Sex Transm Dis. 2011;38:989-94.
125. Schwebke JR, Sadler R, Sutton JM, Hook EW 3rd. Positive screening tests for gonorrhea and chlamydial infection fail to lead consistently to treatment of patients attending a sexually transmitted disease clinic. Sex Transm Dis. 1997;24:181-4.
126. Scott Lamontagne D, Baster K, Emmett L, Nichols T, Randall S, McLean L, et al. Incidence and reinfection rates of genital chlamydial infection among women aged 16-24 years attending general practice, family planning and genitourinary medicine clinics in England: a prospective cohort study by the Chlamydia Recall Study Advisory Group. Sex Transm Infect. 2007;83:292-303.
127. Seneviratne K, Pammi M. Audit of BASHH management standards-key performance indicators in diagnostics. HIV Med. 2014;15(Suppl 3):36.
128. Shafer MA, Boyer CB, Pollack LM, Moncada J, Chang YJ, Schachter J. Acquisition of Chlamydia trachomatis by young women during their first year of military service. Sex Transm Dis. 2008;35:255-9.
129. Shamos SJ, Mettenbrink CJ, Subiadur JA, Mitchell BL, Rietmeijer CA. Evaluation of a testing-only "express" visit option to enhance efficiency in a busy STI clinic. Sex Transm Dis. 2008;35:336-40.
130. Shannon CL, Koussa M, Lee SJ, Fournier J, Abdalian SE, Rotheram MJ, et al. Community-Based, Point-of-Care Sexually Transmitted Infection Screening Among High-Risk Adolescents in Los Angeles and New Orleans: Protocol for a Mixed-Methods Study. JMIR Res Protoc. 2019;8:E10795.
131. Sherley M, Kennedy KJ, Martin SJ. Screening with nucleic acid amplification tests for gonorrhoea in men who have sex with men. Med J Aust. 2012;197:332.
132. Siassakos D, Manley K, Wardle P, Halawa S. Chlamydia screening or prophylaxis before laparoscopy and dye hydrotubation: no readmissions, no worry, or is that so? Int J STD AIDS. 2007;18:861-2.
133. Silva A, Glick NR, Lyss SB, Hutchinson AB, Gift TL, Pealer LN, et al. Implementing an HIV and sexually transmitted disease screening program in an emergency department. Ann Emerg Med. 2007;49:564-72.
134. Snow A, Fortune R, Chen M, Fairley C, Lee D. An audit of time to treatment for bacterial STIs, and time to provision of HIV diagnosis, in a large urban sexual health clinic. Sex Transm Infect. 2016;92(Suppl 1):A22.
135. Somayaji R, Naugler C, Guo M, Church D. Examining Chlamydia trachomatis and Neisseria gonorrhoeae rates between 2010 and 2015: a population-based observational study. Int J STD AIDS. 2017;28:822-8.
136. Sparrow M, Lewis H, Brown P, Bromhead C, Fernando D, Maitra A. Chlamydia screening in Wellington Family Planning Association (FPA) clinics: a demonstration project. N Z Med J. 2007;120:U2490.
137. Spaulding AC, Clarke JG, Jongco AM, Flanigan TP. Small reservoirs: jail screening for gonorrhea and Chlamydia in low prevalence areas. J Correct Health Care. 2009;15:28-34; quiz 80-1.
138. Spielberg F, Levy V, Lensing S, Chattopadhyay I, Venkatasubramanian L, Acevedo N, et al. Fully integrated e-services for prevention, diagnosis, and treatment of sexually transmitted infections: results of a 4-county study in California. Am J Public Health. 2014;104:2313-20.
139. Squance S, Courtney G, Crowley B, McRae S, Loy A. If you don't take a temperature you don't find a fever! Rectal chlamydia in an urban clinic for MSM. HIV Med. 2010;11(Suppl 1):89.
140. Sri T, Southgate E, Kerry SR, Nightingale C, Oakeshott P. Health-related quality of life and Chlamydia trachomatis infection in sexually experienced female inner-city students: a community-based cross-sectional study. Int J STD AIDS. 2017;28:367-71.
141. Stanley L, Ellks R. Can Chlamydia screening in the Emergency Department reach a different section of the target population? Sex Transm Infect. 2012;88:551.
142. Stein MD, Caviness CM, Anderson BJ. Incidence of sexually transmitted infections among hazardously drinking women after incarceration. Womens Health Issues. 2012;22:E1-7.
143. Stoner B, Reno H, Brethauer C, Spear D, Knaup R. "Fast-track" STD services in an urban STD clinic: Increased clinical capacity, but reduced opportunities for same-day treatment. Sex Transm Infect. 2012;88(Suppl 1):A59.
144. Su JY, Skov S. An assessment of the effectiveness of the Tiwi Sexual Health Program 2002-2005. Aust N Z J Public Health. 2008;32:554-8.
145. Swartzendruber A, Sales JM, Brown JL, Davis TL, DiClemente RJ, Rose E. Predictors of repeat Chlamydia trachomatis and/or Neisseria gonorrhoeae infections among African-American adolescent women. Sex Transm Infect. 2013;89:76-82.
146. Tait IA, Hart CA. Chlamydia trachomatis in non-gonococcal urethritis patients and their heterosexual partners: routine testing by polymerase chain reaction. Sex Transm Infect. 2002;78:286-8.
147. Tanaka M, Nakayama H, Sakumoto M, Matsumoto T, Akazawa K, Kumazawa J. Trends in sexually transmitted diseases and condom use patterns among commercial sex workers in Fukuoka City, Japan 1990-93. Genitourin Med. 1996;72:358-61.
148. Tanaka M, Nakayama H, Sakumoto M, Takahashi K, Nagafuji T, Akazawa K, et al. Reduced chlamydial infection and gonorrhea among commercial sex workers in Fukuoka City, Japan. Int J Urol. 1998;5:471-5.
149. Tao G, Hoover KW, Nye MB, Peters P, Gift TL, Peruvemba R, et al. Rectal Infection With Neisseria gonorrhoeae and Chlamydia trachomatis in Men in the United States. Clin Infect Dis. 2016;63:1325-31.
150. Tayal SC, Ochogwu SA, Crindon S. Audit of partner notification for chlamydia infection in the genitourinary medicine clinic at the University Hospital of Hartlepool: 2004-2008. Int J STD AIDS. 2010;21:516-8.
151. Taylor MM, Reilley B, Yellowman M, Anderson L, de Ravello L, Tulloch S. Use of expedited partner therapy among chlamydia cases diagnosed at an urban Indian health centre, Arizona. Int J STD AIDS. 2013;24:371-4.
152. Tebb K, Wibbelsman C, Ko T, Neuhaus JM, Shafer MA. Translating and sustaining a chlamydial screening intervention 4 years later. Arch Intern Med. 2011;171:1767-8.
153. Tebb KP, Shafer M, Wibbelsman CJ, Pecson S, Tipton AC, Neuhaus JM, et al. To screen or not to screen: prevalence of C. trachomatis among sexually asymptomatic male adolescents attending health maintenance pediatric visits. J Adolesc Health. 2004;34:166-8.
154. Templeton DJ, Tyson BA, Meharg JP, Habgood KE, Bullen PM, Malek S, et al. Aboriginal health worker screening for sexually transmissible infections and blood-borne viruses in a rural Australian juvenile correctional facility. Sex Health. 2010;7:44-8.
155. Teplow-Phipps R, Catallozzi M, Stockwel M, Shearer LS, Chuang JH, Pfeffer BS, et al. Management and follow-up of positive chlamydia tests in an urban adolescent population: A quality improvement (QI) evaluation. J Adolesc Health. 2013;52(Suppl 1):S23-4.
156. Tipple C, Rayment M, Mandalia S, Walton L, O'Neill S, Murray J, et al. An evaluation study of the Becton-Dickinson ProbeTec Qx (BDQx) Trichomonas vaginalis trichomoniasis molecular diagnostic test in two large, urban STD services. Sex Transm Infect. 2017;20:20.
157. Torrone EA, Geisler WM, Gift TL, Weinstock HS. Chlamydia trachomatis Infection Among Women 26 to 39 Years of Age in the United States, 1999 to 2010. Sex Transm Dis. 2013;40:335-7.
158. Turner K, Clarke W, Priestley C, Chapman C, Callaghan S, Scofield S. 'By Royal appointment': Community sexually transmitted infection screening at the 'Bournemouth Pride event'. Int J STD AIDS. 2013;24(Suppl 1):31.
159. Turner KM, Horner PJ, Trela-Larsen L, Sharp M, May M. Chlamydia screening, retesting and repeat diagnoses in Cornwall, UK 2003-2009. Sex Transm Infect. 2013;89:70-5.
160. Turok DK, Eisenberg DL, Teal SB, Westhoff CL, Keder LM, Creinin MD. Evaluation of pelvic infection in women using LilettaTM, a new 52 mg levonorgestrel-releasing intrauterine system, for up to 2 years. Int J Gynecol Obstet. 2015;131(Suppl 5):E136.
161. Underhill G, Hewitt G, McLean L, Randall S, Tobin J, Harindra V. Who has chlamydia? The prevalence of genital tract Chlamydia trachomatis within Portsmouth and South East Hampshire, UK. J Fam Plann Reprod Health Care. 2003;29:17-20.
162. Vacca SH, Gold MA, Salsgiver EL, Rodriquez J, Neu N. Patient delivered expedited partner therapy for chlamydia among female adolescents using school based health centers: Is it feasible? J Pediatr Adolesc Gynecol. 2017;30:319.
163. van Bergen JE, Fennema JS, van den Broek IV, Brouwers EE, de Feijter EM, Hoebe CJ, et al. Rationale, design, and results of the first screening round of a comprehensive, register-based, Chlamydia screening implementation programme in the Netherlands. BMC Infect Dis. 2010;10:293.
164. Van Den Broek IV, Van Bergen JA, Fennema HS, Gotz HM, Hoebe CJ, Over E, et al. Main results and impact analysis of annual chlamydia screening in a large register-based programme in the netherlands. Sex Transm Infect. 2011;87:A45.
165. Van Der Bij AK, Geskus RB, Fennema HS, Adams K, Coutinho RA, Dukers NH. No evidence for a sustained increase in sexually transmitted diseases among heterosexuals in Amsterdam, The Netherlands: a 12-year trend analysis at the sexually transmitted disease outpatient clinic Amsterdam. Sex Transm Dis. 2007;34:461-7.
166. Van Der Helm JJ, Koekenbier RH, Van Rooijen MS, De Vries HJ. What is the optimal time to rescreen STI clinic visitors with a urogenital chlamydia infection? Sex Transm Infect. 2013;89(Suppl 1):A339.
167. Van Rijckevorsel GG, Sonder GJ, Bovee LP, Thiesbrummel HF, Geskus RB, Van Den Hoek A. Trends in hepatitis A, B, and shigellosis compared with gonorrhea and syphilis in men who have sex with men in Amsterdam, 1992-2006. Sex Transm Dis. 2008;35:930-4.
168. Van Rooijen MS, Schim Van Der Loeff MF, Van Dam AP, Speksnijder AG, De Vries HJ. Persistence of pharyngeal chlamydia trachomatis for 1-2 weeks is common among clients at the amsterdam STI clinic. Sex Transm Infect. 2013;89(Suppl 1):A62.
169. Varma R. Women requiring emergency contraception are a high risk group for sexually transmitted infections in future. Sex Transm Infect. 2012;88(Suppl 1):A42.
170. Veldhuijzen IK, Van Bergen JE, Gotz HM, Hoebe CJ, Morre SA, Richardus JH, et al. Reinfections, persistent infections, and new infections after general population screening for Chlamydia trachomatis infection in the Netherlands. Sex Transm Dis. 2005;32:599-604.
171. Verhoeven V, Avonts D, Van Royen P, Denekens J. Implementation of a pilot programme for screening for chlamydial infection in general practice. Eur J Gen Pract. 2004;10:157-61.
172. Vermeiren AP, Hoebe CJ, Van Liere GA, Kauhl B, Ziemann A, Krafft T, et al. Geographical clustering of repeat positive tests with chlamydia trachomatis among young people (16-29 Years); Identification of a hidden key chlamydia population. Sex Transm Infect. 2013;89(Suppl 1):A155.
173. Vodstrcil LA, Fairley CK, Fehler G, Leslie D, Walker J, Bradshaw CS, et al. Trends in chlamydia and gonorrhea positivity among heterosexual men and men who have sex with men attending a large urban sexual health service in Australia, 2002-2009. BMC Infect Dis. 2011;11:158.
174. Walker J, Tabrizi SN, Fairley CK, Chen MY, Bradshaw CS, Twin J, et al. Chlamydia trachomatis incidence and re-infection among young women--behavioural and microbiological characteristics. PLoS ONE. 2012;7:E37778.
175. Weaver ER, Bowring AL, Guy R, van Gemert C, Hocking JS, Boyle DI, et al. Reattendance and chlamydia retesting rates at 12 months among young people attending Australian general practice clinics 2007-10: a longitudinal study. Sex Health. 2014;11:366-9.
176. Welsh A, Currie A. Managing gonorrhoea - Are we meeting the BASHH standards? Int J STD AIDS. 2015;26:55-6.
177. Wetten S, Mohammed H, Yung M, Mercer CH, Cassell JA, Hughes G. Diagnosis and treatment of chlamydia and gonorrhoea in general practice in England 2000-2011: a population-based study using data from the UK Clinical Practice Research Datalink. BMJ Open. 2015;5:E007776.
178. Whitehead SJ, Leelawiwat W, Jeeyapant S, Chaikummao S, Papp J, Kilmarx PH, et al. Increase in sexual risk behavior and prevalence of Chlamydia trachomatis among adolescents in Northern Thailand. Sex Transm Dis. 2008;35:883-8.
179. Wijers J, van Liere G, Hoebe C, Cals JW, Wolffs PF, Dukers-Muijrers N. Test of cure, retesting and extragenital testing practices for Chlamydia trachomatis and Neisseria gonorrhoeae among general practitioners in different socioeconomic status areas: A retrospective cohort study, 2011-2016. PLoS ONE. 2018;13:E0194351.
180. Williams E, Leverett K, Apea V. Rising STI rate in female sex workers attending an inner city dedicated clinic. Sex Transm Infect. 2015;91:A88.
181. Williams JA, Weaver B, Van Der Pol B, Mi D, Fortenberry JD. Mycoplasma genitalium DNA detected from adolescent males in a longitudinal cohort. Sex Transm Infect. 2013;89(Suppl 1):A167.
182. Williamson L, Proserpio M, Dosekun O. Audit of re-testing and reinfection in London men who have sex with men with acute STIs in a large gum outpatient clinic. Sex Transm Infect. 2015;91:A25.
183. Wilson SP, Iordanova R, Knych M, Mahan M, Vohra T. Are we acting appropriately by presumptively treating men more aggressively than women for gonorrhea and chlamydia in the urban emergency department? Ann Emerg Med. 2014;64(Suppl 1):S91.
184. Wilson SP, Knych M, Iordanova R, Mahan M, Vohra T. Prevalence of gonorrhea and chlamydia in the emergency department and effectiveness of presumptive treatment. Ann Emerg Med. 2014;64(Suppl 1):S91.
185. Wilson SP, Vohra T, Knych M, Goldberg J, Price C, Calo S, et al. Gonorrhea and chlamydia in the emergency department: Continued need for more focused treatment for men, women and pregnant women. Am J Emerg Med. 2017;35:701-3.
186. Wingrove I, McOwan A, Whitlock G. Using GeneXpert within the clinic to test for gonorrhoea and chlamydia reduces the time to treatment. HIV Med. 2014;15(Suppl 3):92.
187. Yeung A, Temple-Smith M, Bingham A, Fairley C, Law M, Guy R, et al. Is concurrency, number of partners or duration of partnership the most important factor associated with chlamydia in Young Australian Adults? Sex Transm Infect. 2013;89(Suppl 1):A153-4.
188. Zwank MD, Burnett AM, Anderson CP. Laboratory confirmed gonorrhea and/or chlamydia rates in clinically diagnosed PID and cervicitis. Acad Emerg Med. 2011;18(Suppl 1):S127.

**OUTCOME**

1. ACTRN12605000411640. Chlamydia screening in general practice - a randomised controlled trial.

http://wwwwhoint/trialsearch/Trial2aspx?TrialID=ACTRN12605000411640. 2005.

1. Aghaizu A, Adams EJ, Turner K, Kerry S, Hay P, Simms I, et al. What is the cost of pelvic inflammatory disease and how much could be prevented by screening for chlamydia trachomatis? Cost analysis of the Prevention of Pelvic Infection (POPI) trial. Sex Transm Infect. 2011;87:312-7.
2. Ahmad FA, Jeffe DB, Plax K, Schechtman KB, Doerhoff DE, Garbutt JM, et al. Characteristics of youth agreeing to electronic sexually transmitted infection risk assessment in the emergency department. Emerg Med J. 2018;35:46-51.
3. Ahmed N, Jayasinghe Y, Moore EE, Fenner Y, Fletcher A, Tabrizi SN, et al. Knowledge, barriers and facilitating factors for chlamydia screening in victorian women aged 16-25 using social networking sites. J Pediatr Adolesc Gynecol. 2011;24:E63.
4. Al-Kattan T, White S. University students' perspectives on community pharmacy public health services: A qualitative study. Int J Pharm Pract. 2011;19(Suppl 2):80-1.
5. Allen JS, Rhee JY, Calderon Y. The interest an urban hospital 15-24 year old population has in receiving testing for sexually transmitted infections (chlamydia, gonorrhea, and human papillomavirus). Acad Emerg Med. 2013;20(Suppl 1):S302.
6. Andersen B, Eidner PO, Hagensen D, Lomborg S, Hoff G. Opportunities screening of young men for urogenital Chlamydia trachomatis infection in general practice. Scand J Infect Dis. 2005;37:35-9.
7. Andersen B, Olesen F, Moller JK, Ostergaard L. Population-based strategies for outreach screening of urogenital Chlamydia trachomatis infections: a randomized, controlled trial. J Infect Dis. 2002;185:252-8.

9. Andersen B, Ostergaard L, Moller JK, Olesen F. Home sampling versus conventional contact tracing for detecting Chlamydia trachomatis infection in male partners of infected women: randomised study. BMJ. 1998;316:350-1.

1. Andrinopoulos KM. Examining HIV/AIDS within the context of incarceration in Jamaica. PhD [dissertation]. Maryland: Johns Hopkins University; 2008. Available from: Dissertation Abstracts International: Section B: The Sciences and Engineering.
2. Araujo MA, Montagner MA, da Silva RM, Lopes FL, de Freitas MM. Symbolic violence experienced by men who have sex with men in the primary health service in Fortaleza, Ceara, Brazil: Negotiating identity under stigma. AIDS Patient Care STDs. 2009;23:663-8.

12. Arnet I, Gudka S, Salter S, Hersberger KE, Clifford R. Readiness of pharmacists and consumers for pharmacy-based chlamydia screening in Australia and Switzerland. Sexual & Reproductive HealthCare. 2018;16:138-53.

1. Balfe M, Brugha R, O'Connell E, McGee H, O'Donovan D, Vaughan D. Why don't young women go for Chlamydia testing? A qualitative study employing Goffman's stigma framework. Health Risk Soc. 2010;12:131-48.
2. Baraitser P, Pearce V, Holmes J, Horne N, Boynton PM. Chlamydia testing in community pharmacies: evaluation of a feasibility pilot in south east London. Qual Saf Health Care. 2007;16:303-7.
3. Barbee LA, Tat S, Dhanireddy S, Marrazzo JM. Implementation and Operational Research: Effectiveness and Patient Acceptability of a Sexually Transmitted Infection Self-Testing Program in an HIV Care Setting. J Acquir Immune Defic Syndr. 2016;72:E26-31.
4. Blake DR, Kearney MH, Oakes JM, Druker SK, Bibace R. Improving participation in Chlamydia screening programs: perspectives of high-risk youth. Arch Pediatr Adolesc Med. 2003;157:523-9.
5. Blake DR, Lemay CA, Indurkhya A. Correlates of readiness to receive Chlamydia screening among 2 populations of youths. Arch Pediatr Adolesc Med. 2007;161:1088-94.
6. Bloomfield PJ, Kent C, Campbell D, Hanbrook L, Klausner JD. Community-based chlamydia and gonorrhea screening through the United States mail, San Francisco. Sex Transm Dis. 2002;29:294-7.
7. Bloomfield PJ, Steiner KC, Kent CK, Klausner JD. Repeat chlamydia screening by mail, San Francisco. Sex Transm Infect. 2003;79:28-30.
8. Booth AR, Norman P, Harris PR, Goyder E. Using the theory of planned behaviour and self-identity to explain chlamydia testing intentions in young people living in deprived areas. Br J Health Psychol. 2014;19:101-12.
9. Brook G, Burton J, McSorley J, Murphy S. The effectiveness of SMS texts for reminding patients at high risk of sexually transmitted infections and HIV to return for testing. Int J STD AIDS. 2013;24(Suppl 1):34.
10. Burstein GR, Snyder MH, Conley D, Newman DR, Walsh CM, Tao G, et al. Chlamydia screening in a Health Plan before and after a national performance measure introduction. Obstet Gynecol. 2005;106:327-34.
11. Champion JD, Wilford K, Shain RN, Piper JM. Risk and protective behaviours of bisexual minority women: A qualitative analysis. Int Nurs Rev. 2005;52:115-22.
12. Chow EP, Fehler G, Chen MY, Bradshaw CS, Denham I, Law MG, et al. Testing commercial sex workers for sexually transmitted infections in Victoria, Australia: an evaluation of the impact of reducing the frequency of testing. PLoS ONE. 2014;9:E103081.
13. Chow EP, Fehler G, Chen M, Bradshaw C, Fairley C. Evaluation of the change in screening regulation of sex workers for sexually transmitted infections in Victoria, Australia. Sex Transm Dis. 2014;41(Suppl 1):S14.
14. Collins M, Holehouse R, Kaczorowski J. Chlamydia screening in an international resort community: A pilot outreach program to expand access. J Adolesc Health. 2011;48(Suppl 1):S63.
15. De Baetselier I, Smet H, Abdellati S, De Deken B, Cuylaerts V, Reyniers T, et al. Evaluation of the 'Colli-Pee', a first-void urine collection device for self-sampling at home for the detection of sexually transmitted infections, versus a routine clinic-based urine collection in a one-to-one comparison study design: efficacy and acceptability among MSM in Belgium. BMJ Open. 2019;9:E028145.
16. De Barbeyrac B, Rahib D, De Diego S, Le Roy C, Bebear C, Lydie N. Internet testing for chlamydia trachomatis in France in 2012. Sex Transm Infect. 2013;89(Suppl 1):A155-6.
17. Denison HJ, Bromhead C, Grainger R, Dennison EM, Jutel A. Barriers to sexually transmitted infection testing in New Zealand: a qualitative study. Aust N Z J Public Health. 2017;41:432-7.
18. Douge J, Trent M, Liebow E, Wagaman K, Blount S, Gay J. Community Perspectives On Developing An Educational Campaign To Improve Sexually Transmitted Infection (STI) Prevention Knowledge And Access To STI Testing Among Suburban Youth. J Adolesc Health. 2019;64 (Suppl 2):S131-2.
19. East L, Jackson D, O'Brien L, Peters K. Healthcare experiences of women who have been diagnosed with a sexually transmitted infection. J Clin Nurs. 2011;20:2259-65.
20. Eaton S, Biggerstaff D, Pink J, Petrou S, Osipenko L, Gibbs J, et al. Factors aff ecting young people's preferences for emerging technologies for chlamydia testing and treatment: A discrete choice experiment in England. Lancet. 2016;388(Suppl 2):S44.

33. Fenton KA, Copas A, Mitchell K, Elam G, Carder C, Ridgway G, et al. The acceptability of urinary LCR testing for Chlamydia trachomatis among participants in a probability sample survey of sexual attitudes and lifestyles. Sex Transm Infect. 2001;77:194-8.

1. Ford CA, Jaccard J, Millstein SG, Viadro CI, Eaton JL, Miller WC. Young adults' attitudes, beliefs, and feelings about testing for curable STDs outside of clinic settings. J Adolesc Health. 2004;34:266-9.
2. Fuller SS, Aicken C, Sutcliffe LJ, Estcourt CS, Gkatzidou V, Hone K, et al. What are young people's perceptions of using electronic self-tests for STIs linked to mobile technology for diagnosis and care (eSTI2)? Sex Transm Infect. 2013;89(Suppl 1):A69-70.
3. Garcia-Perez H, Harlow SD, Denman C. The effect of pelvic pain and urinary incontinence on women's self-rated health in northern Mexico. Int Urogynecol J Pelvic Floor Dysfunct. 2018;29:243-50.
4. Graseck AS, Secura GM, Allsworth JE, Madden T, Peipert JF. Home compared with clinic-based screening for sexually transmitted infections: a randomized controlled trial. Obstet Gynecol. 2010;116:1311-8.
5. Green R, Kerry SR, Reid F, Hay PE, Kerry SM, Aghaizu A, et al. Where do sexually active female London students go to access healthcare? Evidence from the POPI (Prevention of Pelvic Infection) chlamydia screening trial. Sex Transm Infect. 2012;88:382-5.

39. Griner SB, Vamos CA, Puccio JA, Perrin KM, Beckstead JW, Daley EM. "I'll Just Pick It Up...": Women's Acceptability of Self-Sampling Methods for Sexually Transmitted Infection Screening. Sex Transm Dis. 2019;46:762-7.

1. Habel MA, Brookmeyer KA, Oliver-Veronesi R, Haffner MM. Creating innovative sexually transmitted infection testing options for university students: The impact of an STI self-testing program. Sex Transm Dis. 2018;45:272-7.
2. Hart-Cooper G, Owusu-Edusei K, Chesson H, Hoover K. Opt-out rectal screening for chlamydia and gonorrhea in young men who have sex with men (YMSM). Sex Transm Infect. 2013;89(Suppl 1):A284.
3. Hartney T, Baraitser P, Nardone A. The behavioural impact of chlamydia testing and attitudes towards testing among young adults in england. Sex Transm Infect. 2013;89(Suppl 1):A56-7.
4. Hocking J. Screening for chlamydia: Does it work, results from accept. Sex Transm Infect. 2015;91:A3.
5. Hocking J, Temple-Smith M, Guy R, Kong F, Low N, Donovan B, et al. The australian chlamydia control effectiveness pilot (ACCEPt): Early results: From a randomised trial of annual chlamydia screening in general practise. Sex Transm Infect. 2013;89(Suppl 1):A339-40.
6. Hocking J, Temple-Smith M, Poznanski S, Guy R, Low N, Donovan B, et al. Australian chlamydia control effectiveness pilot: Preliminary results from a trial of chlamydia testing in general practice. Sex Transm Infect. 2011;87:A202.
7. Hocking JS, Temple-Smith M, Low N, Donovan B, Gunn J, Law M, et al. Accept (Australian chlamydia control effectiveness pilot): Design of the pilot evaluation. Sex Health. 2009;6:367-8.
8. Hubach RD, Dodge B, Davis A, Smith AD, Zimet GD, Van Der Pol B. Preferred methods of sexually transmitted infection service delivery among an urban sample of underserved midwestern men. Sex Transm Dis. 2014;41:129-32.
9. Jackson L, Thorley N, Munetsi L, Ross J. Evaluating online and clinic-based STI screening services: A case study of umbrella sexual health services, UK. Sex Transm Infect. 2019;95 (Suppl 1):A112-3.
10. Jackson RM, Gill M, Foley E, Patel R. Do patients have confidence in trained non medical health care staff screening for sexually transmitted infections? Sex Transm Infect. 2013;89(Suppl 1):A132.
11. Jones L, Ricketts E, Town K, Lecky D, Rugman C, Folkard K, et al. Perceptions of chlamydia screening, contraception and HIV testing among 16-24 year old patients visiting a GP surgery. Sex Transm Infect. 2016;92(Suppl 1):A57-8.
12. Jones S, Barker S, Athan E, Graves S. The tip of the iceberg: opportunistic screening for Chlamydia trachomatis in asymptomatic patients attending a young people's health clinic reveals a high prevalence--a pilot study. Sex Health. 2004;1:115-9.
13. Kang M, Hillier L. Medieval wenches and other icky connotations: How young people's constructions of sexually transmitted infections deepen our understanding of their sexual health. J Adolesc Health. 2014;54(Suppl 1):S65.
14. Kersaudy-Rahib D, Lydie N, Leroy C, March L, Bebear C, Arwidson P, et al. Chlamyweb Study II: a randomised controlled trial (RCT) of an online offer of home-based Chlamydia trachomatis sampling in France. Sex Transm Infect. 2017;93:188-95.
15. Kimble TD. A new paradigm for sexually transmitted infection (STI) screening in the hampton roads community of southeastern Virginia. Fertil Steril. 2014;102(Suppl 1):E246.
16. Koekenbier LK, Zuure F, Davidovich U. Why individuals do not return their requested chlamydia trachomatis (CT) home collection kit: Results from a qualitative study. Sex Transm Infect. 2011;87:A224.
17. Kohl KS, Sternberg MR, Markowitz LE, Blythe MJ, Kissinger P, Lafferty WE, et al. Screening of males for Chlamydia trachomatis and Neisseria gonorrhoeae infections at STD clinics in three US cities - Indianapolis, New Orleans, Seattle. Int J STD AIDS. 2004;15:822-8.
18. Lim RBT, Cheung ONY, Tai BC, Chen MI, Chan RKW, Wong ML. Efficacy of multicomponent culturally tailored HIV/ STI prevention interventions targeting foreign female entertainment workers: a quasi-experimental trial. Sex Transm Infect. 2018;94:449-56.
19. Low N, McCarthy A, Macleod J, Salisbury C, Campbell R, Roberts TE, et al. Epidemiological, social, diagnostic and economic evaluation of population screening for genital chlamydial infection. Health Technol Assess. 2007;11:iii-iv, ix-xii, 1-165
20. Masinter L, Parchem S, Perez E, Redman S, Nelson S. Findings from a rigorous evaluation of a chicago school based STD program. Sex Transm Dis. 2018;45 (Suppl 2):S45.
21. Mays RM, Zimet GD, Winston Y, Kee R, Dickes J, Su L. Human papillomavirus, genital warts, pap smears, and cervical cancer: Knowledge and beliefs of adolescent and adult women. Health Care Women Int. 2000;21:361-74.
22. McCadden A, Fenton KA, McManus S, Mercer CH, Erens B, Carder C, et al. Chlamydia trachomatis testing in the second British national survey of sexual attitudes and lifestyles: respondent uptake and treatment outcomes. Sex Transm Dis. 2005;32:387-94.
23. McDonagh L, Hunt E, Naidoo C, Saunders J, Dunbar K, Amlot R, et al. Psychosocial factors associated with chlamydia retesting among young people in the UK. Sex Transm Infect. 2019;95 (Suppl 1):A179.
24. McMunn VA, Caan W. Chlamydia infection, alcohol and sexual behaviour in women. Br J Midwifery. 2007;15:221-4.
25. Mooney-Somers J, Olsen A, Erick W, Scott R, Akee A, Maher L. Young Indigenous Australians' Sexually Transmitted Infection Prevention Practices: A Community-based Participatory Research Project. J Community Appl Soc Psychol. 2012;22:519-32.
26. Morelli A, Baraitser P, Syred J. Addressing clinical sexual health questions of people living in south-east London through an online sexual health provider: A qualitative study. HIV Med. 2018;19(Suppl 2):S96.
27. Mulholland E, Van Wersch A. Stigma, sexually transmitted infections and attendance at the GUM Clinic: an exploratory study with implications for the theory of planned behaviour. J Health Psychol. 2007;12:17-31.
28. Nadarzynski T, Burton J, Henderson K, Zimmerman D, Hill O, Graham C. Targeted advertisement of chlamydia screening on social media: A mixed-methods analysis. Digital Health. 2019;5:2055207619827193.
29. Newton DC, Bayly C, Fairley CK, Chen M, Williams H, Keogh L, et al. The impact of pelvic inflammatory disease on sexual, reproductive and psychological health. J Sex Med. 2011;8(Suppl 3):233.
30. Norman JE, Wu O, Twaddle S, Macmillan S, McMillan L, Templeton A, et al. An evaluation of economics and acceptability of screening for Chlamydia trachomatis infection, in women attending antenatal, abortion, colposcopy and family planning clinics in Scotland, UK. BJOG. 2004;111:1261-8.
31. Normansell R, Drennan VM, Oakeshott P. Exploring access and attitudes to regular sexually transmitted infection screening: the views of young, multi-ethnic, inner-city, female students. Health Expect. 2016;19:322-30.
32. Pastolero P, Suss A, Hammerschlag M. Acceptability of point of care testing for chlamydia trachomatis in adolescents: If we make it, will they take it? Sex Transm Infect. 2019;95 (Suppl 1):A193.
33. Patterson-Rose S, Hesse E, Gaydos C, Widdice L. Acceptability and perceived accuracy of rapid and standard sexually transmitted infection (STI) screening and self-collection of samples on a mobile health van. Sex Transm Dis. 2014;41(Suppl 1):S79.
34. Patton ME, Kirkcaldy RD, Chang DC, Markman S, Yellowman M, Petrosky E, et al. Increased Gonorrhea Screening and Case Finding After Implementation of Expanded Screening Criteria-Urban Indian Health Service Facility in Phoenix, Arizona, 2011-2013. Sex Transm Dis. 2016;43:396-401.
35. Pavlin NL, Parker R, Fairley CK, Gunn JM, Hocking J. Take the sex out of STI screening! Views of young women on implementing chlamydia screening in General Practice. BMC Infect Dis. 2008;8:62.
36. Pickett ML, Melzer-Lange MD, Miller MK, Menon S, Visotcky AM, Drendel AL. Perceived Patient Preference and Clinical Testing for Chlamydia and Gonorrhea in Females: How Closely Are These Aligned? Clin Pediatr (Phila). 2018;57:106-8.
37. Pimenta JM, Catchpole M, Rogers PA, Perkins E, Jackson N, Carlisle C, et al. Opportunistic screening for genital chlamydial infection. I: acceptability of urine testing in primary and secondary healthcare settings. Sex Transm Infect. 2003;79:16-21.
38. Pittaway H, Barnard S, Wilson E, Baraitser P. SH:24-user perspectives on an online sexual health service. Sex Transm Infect. 2016;92(Suppl 1):A19-20.
39. Poedel RJ. A narrative exploration of emerging adult females and their experiences with recurrent chlamydia infection. PhD [dissertation]. Milwaukee: University of Wisconsin; 2010. Available from: UMI Dissertation Publishing.
40. Potocki BL. Exploring STI screening intentions and behaviors in relationships: Integrating individual and relational determinants. PhD [dissertation]. Ohio: Ohio State Unviersity; 2017. Available from: Dissertation Abstracts International Section A: Humanities and Social Sciences.
41. Richardson D, Maple K, Perry N, Ambler E, Jurd C, Fisher M. A pilot qualitative analysis of the psychosocial factors which drive young people to decline chlamydia testing in the UK: implications for health promotion and screening. Int J STD AIDS. 2010;21:187-90.
42. Rose SB, Smith MC, Lawton BA. "If everyone does it, it's not a big deal." Young people talk about chlamydia testing. N Z Med J. 2008;121:33-42.
43. Rostad B, Schmidt L, Sundby J, Schei B. Infertility experience and health differentials - a population-based comparative study on infertile and non-infertile women (the HUNT Study). Acta Obstet Gynecol Scand. 2014;93:757-64.
44. Ryan M, Watson V. Comparing welfare estimates from payment card contingent valuation and discrete choice experiments. Health Econ. 2009;18:389-401.
45. Sagor RS, Golding J, Blake DR. Knowledge is power: Effect of educational interventions on readiness for chlamydia screening. J Adolesc Health. 2013;52:S23.
46. Sanders LS, Nsuami M, Cropley LD, Talyer SN. Reasons given by high school students for refusing sexually transmitted disease screening. Health Educ J. 2007;66:44-57.
47. Scholes D, Heidrich FE, Yarbro P, Lindenbaum JE, Marrazzo JM. Population-based outreach for Chlamydia screening in men: results from a randomized trial. Sex Transm Dis. 2007;34:837-9.
48. Serlin M, Shafer MA, Tebb K, Gyamfi AA, Moncada J, Schachter J, et al. What sexually transmitted disease screening method does the adolescent prefer? Adolescents' attitudes toward first-void urine, self-collected vaginal swab, and pelvic examination. Arch Pediatr Adolesc Med. 2002;156:588-91.
49. Shipitsyna E, Krasnoselskikh T, Zolotoverkhaya E, Savicheva A, Krotin P, Domeika M, et al. Sexual behaviours, knowledge and attitudes regarding safe sex, and prevalence of non-viral sexually transmitted infections among attendees of youth clinics in St. Petersburg, Russia. J Eur Acad Dermatol Venereol. 2013;27:E75-84.
50. Shoveller J, Johnson J, Rosenberg M, Greaves L, Patrick DM, Oliffe JL, et al. Youth's experiences with STI testing in four communities in British Columbia, Canada. Sex Transm Infect. 2009;85:397-401.
51. Shoveller JA, Knight R, Johnson J, Oliffe JL, Goldenberg S. 'Not the swab!' Young men's experiences with STI testing. Sociol Health Illn. 2010;32:57-73.
52. Silver B, Kaldor JM, Rumbold A, Ward J, Smith K, Dyda A, et al. Community and clinic-based screening for curable sexually transmissible infections in a high prevalence setting in Australia: a retrospective longitudinal analysis of clinical service data from 2006 to 2009. Sex Health. 2016;13:140-7.
53. Smardon T, Vaisey A, Chow E, Fairley C, Hocking J. Sexual practices and healthcare use of men who have sex with men only and men who have sex with men and women. Sex Transm Infect. 2019;95 (Suppl 1):A236-7.
54. Smartlowit-Briggs L, Pearson C, Whitefoot P, Altamirano BN, Womack M, Bastin M, et al. Community-Based Assessment to Inform a Chlamydia Screening Program for Women in a Rural American Indian Community. Sex Transm Dis. 2016;43:390-5.
55. Smith LV, Larro ML, Malotte CK, St. Lawrence JS, McFarlane M, Stoner BP, et al. Urine tests for gonorrhea and chlamydia: great technology but will the community accept it? Int Q Community Health Educ. 1999;19:133-43.
56. So J, Shen L. Personalization of risk through convergence of self- and character-risk: Narrative effects on social distance and self-character risk perception gap. Commun Res. 2016;43:1094-115.
57. Songer T, Lave J, Kamlet M, Frederick, S, Ness R. Preferences for fertility in women with pelvic inflammatory disease. Fetil Steril. 2004;81:1344-50.
58. Spielberg F, Kurth A, Gorbach PM, Goldbaum G. Moving from apprehension to action: HIV counseling and testing preferences in three at-risk populations. AIDS Educ Prev. 2001;13:524-40.
59. Su JY, Belton S, Ryder N. Why are men less tested for sexually transmitted infections in remote Australian Indigenous communities? A mixed-methods study. Cult Health Sex. 2016;18:1150-64.
60. Sullivan S, Sullivan P, Stephenson R. Acceptability and Feasibility of a Telehealth Intervention for Sexually Transmitted Infection Testing Among Male Couples: Protocol for a Pilot Study. JMIR Res Protoc. 2019;8:E14481.
61. Swain GR, McDonald RA, Pfister JR, Gradus MS, Sedmak GV, Singh A. Decision analysis: point-of-care Chlamydia testing vs. laboratory-based methods. Clin Med Res. 2004;2:29-35.
62. Teitelman AM, Calhoun J, Duncan R, Washio Y, McDougal R. Young women's views on testing for sexually transmitted infections and HIV as a risk reduction strategy in mutual and choice-restricted relationships. Appl Nurs Res. 2015;28:215-21.
63. Temple-Smith M. How can chlamydia screening work in australian general practice? Sex Transm Infect. 2015;91(Suppl 2):A16.
64. ten Hoor GA, Ruiter RA, van Bergen JE, Hoebe CJ, Houben K, Kok G. Non-participation in chlamydia screening in The Netherlands: determinants associated with young people's intention to participate in chlamydia screening. BMC Public Health. 2013;13:1091.
65. ten Hoor GA, Ruiter RAC, van Bergen J, Hoebe C, Dukers-Muijrers N, Kok G. Predictors of Chlamydia Trachomatis testing: perceived norms, susceptibility, changes in partner status, and underestimation of own risk. BMC Public Health. 2016;16:55.
66. Tilson EC, Sanchez V, Ford CL, Smurzynski M, Leone PA, Fox KK, et al. Barriers to asymptomatic screening and other STD services for adolescents and young adults: focus group discussions. BMC Public Health. 2004;4:21.
67. Trent ME, Ness RB, Bass D, Haggerty CL. Health-related quality of life and biological test results as predictors of adverse adolescent pelvic inflammatory disease outcomes. Sex Transm Infect. 2013;89(Suppl 1):A137.
68. Uuskula A, Kangur K, McNutt LA. Barriers to effective STI screening in a post-Soviet society: results from a qualitative study. Sex Transm Infect. 2006;82:323-6.
69. Vaisey A. The prevalence of chlamydia among 16 to 29 year olds in Australia. Sex Transm Infect. 2015;91(Suppl 2):A16.
70. Walker J, Walker S, Fairley CK, Gunn J, Pirotta M, Gurrin L, et al. Computer reminders for chlamydia screening in general practice: a randomised controlled trial. Sex Health. 2009;6:363.
71. Ward J, Guy R, Garton L, Silver B, Taylor-Thomson D, Hengel B, et al. Addressing endemic rates of STI in remote aboriginal communities in australia using quality improvement as a key strategy: The STRIVE study. Sex Transm Infect. 2013;89(Suppl 1):A371.
72. Wayal S, Llewellyn C, Smith H, Fisher M. Home sampling kits for sexually transmitted infections: preferences and concerns of men who have sex with men. Cult Health Sex. 2011;13:343-53.
73. Wayal S, Llewellyn C, Smith H, Hankins M, Phillips A, Richardson D, et al. Self-sampling for oropharyngeal and rectal specimens to screen for sexually transmitted infections: acceptability among men who have sex with men. Sex Transm Infect. 2009;85:60-4.
74. Wilson E, Free C, Morris TP, Syred J, Menon-Johansson AS, Palmer MJ, et al. Effect of an internet-based sexually transmitted infection testing and results service on diagnoses and testing uptake: A single-blind, randomised controlled trial. The Lancet. 2017;390 (SPEC.ISS 1):S95.
75. Wilson E, Leyrat C, Baraitser P, Free C. Does internet-accessed STI (e-STI) testing increase testing uptake for chlamydia and other STIs among a young population who have never tested? Secondary analyses of data from a randomised controlled trial. Sex Transm Infect. 2019.
76. Wong JPH, Chan KBK, Boi-Doku R, McWatt S. Risk discourse and sexual stigma: Barriers to STI testing, treatment and care among young heterosexual women in disadvantaged neighbourhoods in Toronto. Can J Hum Sex. 2012;21:75-89.
77. Woodhall SC, Sile B, Talebi A, Nardone A, Baraitser P. Internet testing for Chlamydia trachomatis in England, 2006 to 2010. BMC Public Health. 2012;12:1095.
78. Yakubu BD, Simkhada P, Van Teijlingen E, Eboh W. Sexual health information and uptake of sexual health services by African women in Scotland: A pilot study. Int J Health Promot Educ. 2010;48:79-84.
79. Young SD. Potential moral stigma: Defining, testing, and changing people's willingness to test for sexually transmitted infections. PhD [dissertation]. California: Stanford University; 2008. Avaialble from: Dissertation Abstracts International: Section B: The Sciences and Engineering.
80. Young SD, Nussbaum AD, Monin B. Potential moral stigma and reactions to sexually transmitted diseases: evidence for a disjunction fallacy. Pers Soc Psychol Bull. 2007;33:789-99.
81. Zakher B, Kang M. Attitudes to chlamydia screening in general practice among Australian university students: a pilot study. Sex Health. 2008;5:359-63.

**SETTING**

1. ChiCTR1900025462. Pay-it-forward gonorrhea and chlamydia testing among students in China: a randomized controlled trial.

http://wwwwhoint/trialsearch/Trial2aspx?TrialID=ChiCTR1900025462. 2019.

1. Hiransuthikul A, Sungsing T, Jantarapakde J, Trachunthong D, Mills S, Vannakit R, et al. Correlations of chlamydia and gonorrhoea among pharyngeal, rectal and urethral sites among Thai men who have sex with men: multicentre community-led test and treat cohort in Thailand. BMJ Open. 2019;9:E028162.
2. Kakaire O, Byamugisha JK, Tumwesigye NM, Gemzell-Danielsson K. Clinical versus laboratory screening for sexually transmitted infections prior to insertion of intrauterine contraception among women living with HIV/AIDS: a randomized controlled trial. Hum Reprod. 2015;30:1573-9.
3. Li K, Yang F, Huang W, Zhao Y, Wu D, Tang W, et al. A mixed-methods analysis of a pay-it-forward gonorrhea/chlamydia testing program among men who have sex with men in China. Sex Transm Infect. 2019;95 (Suppl 1):A91.
4. Li KT, Tang W, Wu D, Huang W, Wu F, Lee A, et al. Pay-it-forward strategy to enhance uptake of dual gonorrhea and chlamydia testing among men who have sex with men in China: a pragmatic, quasi-experimental study. Lancet infectious diseases. 2019;19:76‐82.
5. Lockhart A, Psioda M, Ting J, Campbell S, Mugo N, Kwatampora J, et al. Prospective Evaluation of Cervicovaginal Self- and Cervical Physician Collection for the Detection of Chlamydia trachomatis, Neisseria gonorrhoeae, Trichomonas vaginalis, and Mycoplasma genitalium Infections. Sex Transm Dis. 2018;45:488-93.
6. NCT03741725. Pay-it-forward RCT for Gonorrhea and Chlamydia Testing. <https://clinicaltrials.gov/ct2/show/NCT03741725>
7. Ndiaye AG, Faye CM, Ndiaye I, Fall K, Gaye AG, Diop IL, et al. Screening for HIV, syphilis. Chlamydia trachomatis and Neisseria gonorrhoreae during a combined survey conducted in Malicouna, a Senegalese rural area. Bull Soc Pathol Exot. 2009;102:150-4.
8. Young T, de Kock A, Jones H, Altini L, Ferguson T, van de Wijgert J. A comparison of two methods of partner notification for sexually transmitted infections in South Africa: patient-delivered partner medication and patient-based partner referral. Int J STD AIDS. 2007;18:338-40.

10. Zhang T, Yang F, Tang W, Huang W, Wang Y, Alexander M, et al. Pay-it-forward gonorrhea and chlamydia testing among Chinese men who have sex with men: a cluster randomized controlled trial. Sex Transm Infect. 2019;95:A89‐.

11. Zhang TP, Yang F, Tang W, Alexander M, Forastiere L, Kumar N, et al. Pay-it-forward gonorrhea and chlamydia testing among men who have sex with men in China: a study protocol for a three-arm cluster randomized controlled trial. Infect. 2019;8:1-11.

**NOT RESEARCH**

1. Abter EI, Mahmud MA. Screening for chlamydia to prevent pelvic inflammatory disease. N Engl J Med. 1996;335:1531-3.
2. ACTRN12610000297022. Australian Chlamydia Control Effectiveness PIlot: a trial to determine whether annual chlamydia testing in general practice can lead to a reduction in chlamydia prevalence.

http://wwwwhoint/trialsearch/Trial2aspx?TrialID=ACTRN12610000297022. 2010.

1. ACTRN12610000358044. Sexually Transmitted Infections (STI) in Remote communities: improVed & Enhanced primary health care.

http://wwwwhoint/trialsearch/Trial2aspx?TrialID=ACTRN12610000358044. 2010.

1. Amor L, Cresswell F, Peters J, Dunne A, Dean G, Paul J. Gonorrhoea in Brighton: A description of epidemiology, antimicrobial resistance and implications for public health interventions. Int J STD AIDS. 2015;26(Suppl 1):110-1.
2. ANAES. Assessment of screening for Chlamydia trachomatis infection of the lower genitourinary tract in France Health Technology Assessment Database [Internet]. 2003; (4). Available from: <http://cochranelibrary-wiley.com/o/cochrane/clhta/articles/HTA-32004000016/frame.html>.
3. ANAES. Place of molecular biology methods in detecting Chlamydia trachomatis infection of the lower genitourinary tract Health Technology Assessment Database [Internet]. 2003; (4). Available from: <http://cochranelibrary-wiley.com/o/cochrane/clhta/articles/HTA-32004000017/frame.html>.
4. Andersen B, Olesen F. Screening for Chlamydia trachomatis. BMJ. 2012;345:E4231.
5. Anonymous. Abstracts of the 4th Joint BASHH-ASTDA Meeting. Sexually Transmitted Infections Conference: 4th Joint BASHH ASTDA Meeting Brighton United Kingdom Conference Publication:. 2012;88(Suppl 1).
6. Anonymous. 2016 STD Prevention Conference. Sexually Transmitted Diseases Conference. 2016;43(Suppl 2)
7. Anonymous. Correction: A randomised controlled trial to evaluate the impact of sexual health clinic based automated text message reminders on testing of HIV and other sexually transmitted infections in men who have sex with men in China: protocol for the T2T Study. BMJ Open. 2017;7:E015787corr1.
8. Assistance Publique - Hôpitaux de Paris. Prevention of Diseases Induced by Chlamydia Trachomatis (i-PREDICT). 2016. ClinicalTrials.gov registration number: NCT02904811.
9. Atherton H, Banks D, Harbit R, Oakeshott P. Recruiting young women to a trial of chlamydia screening. Int J STD AIDS. 2006;17:712.
10. Bower H. Britain launches pilot screening programme for chlamydia. BMJ. 1998;316:1479.
11. Brady M, Ardines E, McBain J, Parry G, Holdsworth G. Integrating online services with face-to-face clinics: How we successfully managed channel shift. HIV Med. 2018;19(Suppl 2):S19.
12. Centers for Disease Control and Prevention. Use of Self-collected Vaginal Swabs as an Innovative Approach to Facilitate Testing for Repeat Chlamydia Infection. 2005. ClinicalTrials.gov registration number: NCT00132457.
13. Chi Wai Wong W, Tsz Hei Lau S, Pui Hang Choi E, Tucker JD, Fairley CK, Saunders JM. A Systematic Literature Review of Reviews on the Effectiveness of Chlamydia Screening. Epidemiol Rev. 2019;30:30.
14. Chow JM. Equipoise redux: do we need more randomized controlled trials of Chlamydia screening? Sex Transm Dis. 2013;40:103-4.
15. Contraceptive Technology Update. Rapid chlamydia test for men examined. Contracept Technol Update. 2009;30:125-6.
16. Danish Centre for Evaluation and Health Tecdhnology Assessment. Chlamydia screening with home testing - a Health Technology Assessment – Primary research, Expert panel, Systematic review, Clinical guidelines. Copenhagen: Danish Centre for Evaluation and Health Technology Assessment; 2002. <https://www.crd.york.ac.uk/CRDWeb/ResultsPage.asp>. Accessed 13 Sept 2020.
17. Eunice Kennedy Shriver National Institute of Child Health and Human Development (NICHD). Randomized Trial of Home Versus Clinic-Based STD Testing. 2012. ClinicalTrials.gov registration number: NCT01654991.
18. Frelih T. Chlamydia screening project starts in Nova Gorica, Slovenia. Euro Surveill. 2005;10:E050609.3.
19. George Washington University. Evaluation of Immediate v. Delayed CT/NG Test for Treatment in the ED. 2013. ClinicalTrials.gov registration number: NCT01989130.
20. Guy R, Ward J, Rumbold A, Silver B, Skov S, Boffa J, et al. The evidence for primary health STI screening programs in remote aboriginal communities. Sex Health. 2009;6:378.
21. Guy RJ, Natoli L, Ward J, Causer L, Hengel B, Whiley D, et al. A randomised trial of point-of-care tests for chlamydia and gonorrhoea infections in remote Aboriginal communities: Test, Treat ANd GO- the "TTANGO" trial protocol. BMC Infect Dis. 2013;13:485.
22. Hadgu A, Brunham RC, Pourbohloul B, Mak S, White R, Rekart ML. Issues in Chlamydia trachomatis testing by nucleic acid amplification test. J Infect Dis. 2006;193:1335-9.
23. Handy P. Sexually transmitted infection screening in asymptomatic women. Int J STD AIDS. 2007;18:648-9.
24. Hay S, Hay P, Oakeshott P. Feasibility of recruiting in a student bar for a trial of chlamydia screening in young women. Fam Pract. 2004;21:223-4.
25. Hicks N R, Dawes M, Fleminger M, Goldman D, Hamling J, Hicks LJ. Evidence based case report. Chlamydia infection in general practice. BMJ. 1999;318:790-2.
26. Hocking JS, Temple-Smith M, Low N, Donovan B, Gunn J, Law M, et al. Accept (Australian chlamydia control effectiveness pilot): design of the pilot evaluation. Sex Health. 2009;6:367‐8.
27. Jenkins WD. Chlamydia and gonorrhea screening in the emergency department setting: increasing evidence of utility and need for further research. Am J Emerg Med. 2019;37:1196.

30. Kachur R, Dunville RL, Le Backes K. New directions in addressing adolescent STD risk. Sex Transm Dis. 2016;43 (Supple 2):S130.

31. Krahn J, Louette A, Caine V, Ha S, Wong T, Lau TTY, et al. Non-standard treatment for uncomplicated Chlamydia trachomatis urogenital infections: a systematic review. BMJ Open. 2018;8:E023808.

32. Loosier PS, Malcarney M-B, Slive L, Cramer RC, Burgess B, Hoover KW, et al. Chlamydia screening for sexually active young women under the affordable care act: new opportunities and lingering barriers. Sex Transm Dis. 2014;41:538-44.

1. Lorimer K. Pilot qualitative analysis of the psychosocial factors which drive young people to decline chlamydia testing in the UK: implications for health promotion and screening. Int J STD AIDS. 2010;21:379.
2. Low N, Harbord RM, Egger M, Sterne JAC, Herrmann B, Macleod J, et al. Screening for chlamydia [2] (multiple letters). Lancet. 2005;365:1539-40.
3. Low N, Hocking J. The POPI trial: What does it mean for chlamydia control now? Sex Transm Infect. 2010;86:158-9.
4. Low N, Redmond S, Uuskula A, van Bergen J, Ward H, Andersen B, et al. Screening for genital chlamydia infection. Cochrane Database Syst Rev. 2013:CD010866.
5. MaHTAS. Point-Of-Care test for Chlamydia Health Technology Assessment Database [Internet]. 2012; (4). Available from:

<http://cochranelibrary-wiley.com/o/cochrane/clhta/articles/HTA-32016000507/frame.html>.

1. Markos AR. Testing asymptomatic heterosexual men for gonorrhoea. Int J STD AIDS. 2010;21:302.
2. McDonagh LK, Saunders JM, Cassell J, Curtis T, Bastaki H, Hartney T, et al. Application of the COM-B model to barriers and facilitators to chlamydia testing in general practice for young people and primary care practitioners: a systematic review. Implement Sci. 2018;13:130
3. Mitka M. CDC: Improve targeted screening for chlamydia. JAMA. 2012;307:1472.
4. Mundy L, Hiller J. Opportunistic screening of asymptomatic individuals for Chlamydia. Adelaide: Adelaide Health Technology Assessment; 2007. https://www.crd.york.ac.uk/CRDWeb/ResultsPage.asp.
5. North Bronx Healthcare Network. Project AWARE: Using the Emergency Department (ED) to Prevent Sexually Transmitted Infections (STIs) in Youth (AWARE). 2010. ClinicalTrials.gov registration number: NCT01195220.
6. Norwegian Institute of Public Health. Home Sampling Versus Conventional Sampling for Screening of Urogenital Chlamydia Trachomatis in Young Men and Women. 2006. ClinicalTrials.gov registration number: NCT00283127.44. O'Byrne P. Recommended screenings for chlamydia and gonorrhea: A Canadian guidelines review. Nurse Pract. 2019;44(2):35-41.
7. O’Connell E, Hogan A, Ricketts E, Jacomelli J, McNulty C. Advantages of chlamydia screening in general practice settings. Prim Health Care. 2013;23:26-9.
8. Oakeshott P, Kerry S, Atherton H, Aghaizu A, Hay S, Taylor-Robinson D, et al. Community-based trial of screening for Chlamydia trachomatis to prevent pelvic inflammatory disease: the POPI (prevention of pelvic infection) trial. Trials. 2008;9:73.
9. Pillay J, Moore A, Rahman P, Lewin G, Reynolds D, Riva J, et al. Screening for chlamydia and/or gonorrhea in primary health care: protocol for systematic review. Syst. 2018;7:248.
10. Pittrof R. Screening for chlamydia to prevent pelvic inflammatory disease. N Engl J Med. 1996;335:1532-3.
11. Powers ME, Adekeye T, Volny R, Braun R, Provost J. Chlamydia screening: what about the men? Am J Public Health. 2011;101:583-5.
12. Primary Health Care. Chlamydia screening trial boosts rate of testing by up to double. Prim Health Care. 2013;23:6
13. RCM Midwives. Men targeted in chlamydia testing drive. RCM Midwives. 2006;9:293.
14. Santer M, Wyke S. Chlamydia trachomatis: Opportunistic screening in primary care. Br J Gen Pract. 2001;51:931.
15. Sheringham J. Screening for Chlamydia. BMJ. 2010;340:C1698.
16. Shih SL, Graseck AS, Secura GM, Peipert JF. Screening for sexually transmitted infections at home or in the clinic? Curr Opin Infect Dis. 2011;24:78-84.
17. Soldan K, Berman SM. Danish health register study: a randomised trial with findings about the implementation of chlamydia screening, but not about its benefits. Sex Transm Infect. 2011;87:86-7.
18. St George's University of London. Community-Based Trial of Screening for Chlamydia Trachomatis to Prevent Pelvic Inflammatory Disease (POPI). 2005. ClinicalTrials.gov registration number: NCT00115388.
19. Stephenson J, Hopwood J, Babiker A, Copas A, Vickers M. Recent pilot studies of chlamydia screening. Sex Transm Infect. 2003;79:352.
20. Tamarelle J, Thiebaut ACM, Sabin B, Bebear C, Judlin P, Fauconnier A, et al. Early screening for Chlamydia trachomatis in young women for primary prevention of pelvic inflammatory disease (i-Predict): study protocol for a randomised controlled trial. Trials. 2017;18:534.
21. Tsoumanis A, Hens N, Kenyon CR. Is Screening for Chlamydia and Gonorrhea in Men Who Have Sex With Men Associated With Reduction of the Prevalence of these Infections? A Systematic Review of Observational Studies. Sex Transm Dis. 2018;45:615-22.
22. Tulane University. Check it: A New Approach to Controlling Chlamydia Transmission in Young People. 2017. ClinicalTrials.gov registration number: NCT03098329.
23. University of Aarhus. Randomized Population-Based Study on Chlamydia Trachomatis Screening. 2009. ClinicalTrials.gov registration number: NCT00827970.
24. University of California Los Angeles. Expedited Partner Therapy for MSM in Peru. 2012. ClinicalTrials.gov registration number: NCT01720654.
25. University of California Los Angeles. STI Screening as a Combined HIV Prevention Platform for MSM in Peru. 2017. ClinicalTrials.gov registration number: NCT03010020.
26. University of Pittsburgh. Home Screening for Chlamydia Surveillance. 2005. ClinicalTrials.gov registration number: NCT00177437.
27. Washington University School of Medicine. Randomized Trial of Home Versus Clinic-based Screening for Sexually Transmitted Infections. 2010. ClinicalTrials.gov registration number: NCT01184157
28. Watkins K, Lee JEC. Narrative Review of Barriers to the Secondary Prevention of Sexually Transmitted Infections: Implications for the Military Context and Current Research Gaps. J Mil Veterans Health. 2014;22:44-52.
29. Wilson E, Free C, Morris TP, Kenward MG, Syred J, Baraitser P. Can Internet-Based Sexual Health Services Increase Diagnoses of Sexually Transmitted Infections (STI)? Protocol for a Randomized Evaluation of an Internet-Based STI Testing and Results Service. JMIR Res Protoc. 2016;5:E9.
30. Winceslaus SJ. Response to tipple et al: Is screening for pharyngeal Chlamydia trachomatis warranted in high-risk groups? Int J STD AIDS. 2011;22:419.
31. Woodhall SC, Turner KME, Hughes G. Maximising the effectiveness of the national chlamydia screening programme in England: Should we routinely retest positives? Sex Transm Infect. 2013;89:2-3.

**OTHER STUDY DESIGN**

1. Balfe M, Brugha R, D OD, E OC, Vaughan D. Triggers of self-conscious emotions in the sexually transmitted infection testing process. BMC Res Notes. 2010;3:229.
2. Chaudhary R, Heffernan CM, Illsley AL, Jarvie LK, Lattimer C, Nwuba AE, et al. Opportunistic screening for Chlamydia: a pilot study into male perspectives on provision of Chlamydia screening in a UK university. J Public Health (Oxf). 2008;30:466-71.
3. Darroch J, Myers L, Cassell J. Sex differences in the experience of testing positive for genital chlamydia infection: a qualitative study with implications for public health and for a national screening programme. Sex Transm Infect. 2003;79:372-3.
4. Fortenberry JD, McFarlane M, Bleakley A, Bull S, Fishbein M, Grimley DM, et al. Relationships of stigma and shame to gonorrhea and HIV screening. Am J Public Health. 2002;92:378-81.
5. Habel MA, Brookmeyer KA, Oliver-Veronesi R, Haffner MM. Creating Innovative Sexually Transmitted Infection Testing Options for University Students: The Impact of an STI Self-testing Program. Sex Transm Dis. 2018;45:272-7.
6. Hogan AH, Howell-Jones RS, Pottinger E, Wallace LM, McNulty CA. "...they should be offering it": a qualitative study to investigate young peoples' attitudes towards chlamydia screening in GP surgeries. BMC Public Health. 2010;10:616.
7. Kounali DZ, Welton NJ, Soldan K, Woodhall SC, Dunbar JK, Migchelsen SJ, et al. Has Chlamydia trachomatis prevalence in young women in England, Scotland and Wales changed? Evidence from national probability surveys. Epidemiol Infect. 2019;147:E107.
8. Learner ER, Torrone EA, Fine JP, Pence BW, Powers KA, Miller WC. Chlamydia Prevalence Trends Among Women and Men Entering the National Job Training Program From 1990 Through 2012. Sex Transm Dis. 2018;45:554-9.
9. Newton DC, Fairley CK, Chen M, Williams H, Keogh L, Temple-Smith M, et al. The impact of chlamydia on sexual, reproductive and psychological health. J Sex Med. 2011;8(Suppl 3):233.
10. Owusu-Edusei K Jr., Bohm MK, Chesson HW, Kent CK. Chlamydia screening and pelvic inflammatory disease: Insights from exploratory time-series analyses. Am J Prev Med. 2010;38:652-7.
11. Temple-Smith M, Hopkins C, Fairley C, Tomnay J, Pavlin N, Parker R, et al. The right thing to do: patients' views and experiences of telling partners about chlamydia. Fam Pract. 2010;27:418-23.
12. Walker-Baban C, Anschuetz G, Lopez G, Gaymon L, Asbel L. Keeping up with the times: The philadelphia high school screening program (HSSP). Sex Transm Dis. 2018;45 (Suppl 2):S37.

13. Yussman SM, Urbach K. Introduction of universal chlamydia and gonorrhea screening in an urban school-based health center. J Adolesc Health. 2018;62 (Suppl1):S80-1.

14. Yussman SM, Urbach K. 243. Universal Chlamydia and Gonorrhea Screening in an Urban School-Based Health Center 2016-2019. J Adolesc Health. 2020;66 (Suppl 2):S123.

**LANGUAGE**

1. Health Council of the Netherlands Gezondheidsraad. Screening for chlamydia. The Hague: Health Council of the Netherlands; 2004.

<https://www.crd.york.ac.uk/CRDWeb/ResultsPage.asp>. Accessed 13 Sept 2020.

1. Ostergaard L, Andersen B, Moller J, Olesen F. Screening for chlamydia with hometest. Copenhagen: Danish Centre for Evaluation and Health Technology Assessment; 2002. <https://www.crd.york.ac.uk/CRDWeb/ResultsPage.asp>. Accessed 13 Sept 2020.
2. Ostergaard L, Andersen B, Olesen F, Møller J. Detection of Chlamydia trachomatis infection among young people. The effect of home-sampling and mailing the samples. 1999;161:4514-8.
3. Postma MJ, Welte R, Hoek JA, Doornum GJ, Coutinho RA, Jager JC. Opportunistic screening for genital infections with Chlamydia trachomatis in sexually active population of Amsterdam. II: Cost-effectiveness analysis of screening women Ned Tijdschr Geneeskd. 1999;143:677-81.

**DUPLICATE**

1. Bartelsman M, Straetemans M, Vaughan K, Alba S, Van Rooijen MS, De Vries HJC. Cost effectiveness of Gram stained smears in the management of urogenital gonorrhea among high risk patients of a sexually transmitted infections outpatient clinic in Amsterdam. Ned Tijdschr Dermatol Venereol. 2014;24:49.
2. Hunjan T, Kerry SR, Normansell R, Hay PE, Sadiq TS, Planche T, et al. Chlamydia testing: Where are we now? Recruiting high-risk women to a pilot STI screening trial. Sex Transm Infect. 2013;89:556.
3. Kersaudy-Rahib D, Lydié N, Leroy C, March L, Bébéar C, Arwidson P, et al. Chlamyweb Study II: a randomised controlled trial (RCT) of an online offer of home-based. Sex Transm Infect. 2017; 93:188-95.
4. Low N, McCarthy A, Macleod J, Salisbury C, Campbell, Roberts T, et al. A study to evaluate the most cost-effective way to screen for Chlamydia trachomatis genital tract infection and reduce its prevalence and associated burden of disease - primary research. Health Technol Assess Database; 2007.
5. O'Farrell N, Weiss H. Effect of chlamydia diagnosis on heterosexual relationships. Sex Transm Infect. 2013;89(Suppl 1):A328.
6. Pimenta JM, Hewitt G, Underhill G, Gleave T, McLean L, Ghosh A, et al. Opportunistic screening for genital chlamydial infection. II: Prevalence among healthcare attenders, outcome, and evaluation of positive cases. Sex Transm Infect. 2003;79:22-7.
7. Sellors J, Paavonen J. Screening for chlamydia to prevent pelvic inflammatory disease. N Engl J Med. 1996;335:1531-3.
8. Zou H, Meng X, Grulich A, Huang S, Jia T, Zhang X, et al. A randomised controlled trial to evaluate the impact of sexual health clinic based automated text message reminders on testing of HIV and other sexually transmitted infections in men who have sex with men in China: Protocol for the T2T Study. BMJ Open. 2017;7:E015787.

**ONGOING STUDY**

1. NCT03715335. Adolescent Sexually Transmitted Infection Screening in the Emergency Department. https://clinicaltrialsgov/show/NCT03715335. 2018.

2. Tamarelle J, Thiébaut ACM, Sabin B, Bébéar C, Judlin P, Fauconnier A, et al. Early screening for Chlamydia trachomatis in young women for primary prevention of pelvic inflammatory disease (i-Predict): study protocol for a randomised controlled trial. Trials. 2017;18:534.
