# Supplementary material for: Screening for chlamydia and/or gonorrhea in primary health care: systematic reviews on effectiveness and patient preferences
Source: Syst Rev. 2021 Apr 19;10:118. doi: 10.1186/s13643-021-01658-w (PMC8056106; doi:10.1186/s13643-021-01658-w)
Supplement: Supplementary file 4 — Additional file 4. Study characteristics tables. [file 13643_2021_1658_MOESM4_ESM.docx]

**Additional file 4: Study characteristics tables**

**Key Question 1: Benefit outcomes**

| **Author, year**  **Country** | **Design; Intensity; Sample size; Risk of Bias** | **Screening rates in intervention group (IG); Rates of testing outside of study (IG; control group [CG])** | **Sex and Age** | **Baseline CT positivity of tested; All sexually active (Y/N)** | **Recruitment** | **Screening approach; CT vs CT & NG; Location, test and person testing; Re-testing; Co-interventions** | **Outcome Assessment**  **Incidence of PID in CG**  **Follow-up Durations** |
| --- | --- | --- | --- | --- | --- | --- | --- |
| **Randomized controlled trials** | | | | | | | |
| Andersen 2011  Denmark | RCT  1 screen offered  15,459  ROB: unclear (detection bias) | 29%  IG 9.0% vs CG 9.4% during 3 mo study period | F  21-24 yrs | 7.1%; N | Population-based via mailed kits | Universal; CT; home-collected vaginal pipette, with NAAT; 70% re-tested; none | **PID**: Hospital discharge ICD codes or doxycycline prescriptions (only used for PID in Denmark; 33% by GPs). Incidence in CG 0.65%. Follow-up duration: 1 yr  **EP and infertility**: hospital discharge ICD codes. Follow-up duration: 9 yrs |
| Garcia 2012  Brazil | RCT Cluster  Screening offered every 8 wks over 3 yrs  20 cities with >50,000 inhabitants; follow-up survey using sampling at random sites at baseline (3,732) and after 4 yrs (4,156)  ROB: unclear (performance bias) | NR (interviewed  FSWs 48,207 times during 20 8-wk cycles)  NR | F  >14 yrs  Mean 24.5 yrs | 15.5% CT, 2.4% NG; Y | Outreach via mobile teams | Universal; CT & NG (and other STIs); mobile sites self-collected vaginal swabs with NAAT; re-testing NR but frequent visits; multi-faceted syndromic management in general population and clients of FSWs; condom promotion with motivational interviewing and free condoms; peer education | **Estimated population prevalence** in FSWs using surveys (>99% of eligible enrolled) at random FSW sites. Follow-up duration: 3.5 yrs |
| Hocking 2018  Rural Australia | RCT Cluster  3 annual screens offered  52 clusters (130 clinics of >500 16-29 yr olds)  ROB: unclear (performance and attrition biases) | 24% ≥1 times over 3 yrs (8.2% pre-trial yr to 20% at 25-36 mos)  IG NR; CG rates increased from 8.2% pre-trial to 12.9% (stable over trial) | F & M  16-29 yrs | 10% in those testing; 4.8% (4.5% females & 5.5% males) in prevalence surveys; Y | Primary care (clinic attenders) | Opportunistic; CT; in-clinic patient-collected vaginal or urine with NAAT; approx. 25% re-testing each year; multi-faceted with provider reminders, incentives, education, payments & feedback, and patient recall systems | **Clinic PID**: cumulative incidence in clinics for women 16-33 with at least one clinic visit during intervention period. Criteria provided to all providers but not blinded. Incidence in CG 0.4%.  **Hospital PID**: ICD codes for all 15-34 yr olds living in each cluster. Incidence in CG 0.4%. Not used for main analysis because of low ascertainment and difference in trial and hospitalized populations  **Estimated population prevalence (in clinic attenders)**: Using surveys of consecutive clinic attenders (70% response) before randomization and at end of trial (difference in change from baseline)  Follow-up duration for all outcomes: 3 yrs |
| Hodgins 2002  Nunavik region in Northern Quebec | RCT Cluster  1 screen offered  12 communities in Nunavut (2,320 15-39 yrs)  ROB: high (incomplete outcome data with reported rates based on low uptake; multiple unclear domains) | 31%  NR | F & M  All; focus on 15-39 yrs | 7%; Y | Outreach via community | Universal CT; home urine sampling with PCR; re-testing NR; intensive community health education program | **Estimated population prevalence** via reported rates over past yr in communities.  Follow-up duration: 1 yr |
| Klovstad 2013  Norway | RCT  1 screen offered  41,519 (10,000 IG)  ROB: low | IG 14% (85% of 16.5% testing via study or healthcare system)    IG 2.5%, CG 3.4% | F & M  18-25 yrs | IG 6.3% vs 11.6%; N | Population-based register via mailed invitations with screening kits (no reminders) | Universal; CT; home urine sampling via mailed kit via NAAT; N; N | **Treatment for CT:** national prescription database (filled at least one prescription for (azithromycin, doxycycline, erythromycin, lymecyklin, amoxicillin) within 30 days following a positive test result. Follow-up duration: 3 mos |
| Oakeshott 2010  London, UK | RCT  1 screen  2,529  ROB: low | 100%  22% both groups (43% of those CT+ in CG) | F  16-27 (mean 21) yrs | 5.4%; Y | Outreach at common rooms, lecture theatres, and student bars at universities and further education colleges in London | Universal; CT; outreach site self-collected vaginal swabs with NAAT; re-testing NR; informed of risks of CT infection | **PID**: Any report by participants or their providers about signs and symptoms or dx, looked to medical records in general practitioners, hospitals, family planning clinics, and genitourinary medicine clinics. Used criteria for all cases, but medical records sometimes incomplete. Incidence in CG 1.8%.  Follow-up duration: 1 yr |
| Ostergaard 2000  Denmark | RCT Cluster  1 screen  17 schools (IG 928 vs CG 833)  ROB: high (attrition [>50%] and lack of cluster analysis), unclear for other domains except allocation concealment | IG 93% vs CG 7.5%  IG 29% &. CG 36% (p=0.04) | F  ≥15 yrs in high school (9% ≥19 yrs) | IG 5% vs CG 7.9%; Y | Outreach in schools with provision of home kits or invitation/reminder to go to general practitioner or STI clinics | Universal; CT; home sampling using vaginal pipette and NAAT vs. in-clinic swab with EIA; re-testing NR; information about consequences | **PID**: Self-reported at follow up questionnaire, with confirmation in registration for prescriptions. Incidence in CG 4.1%. Follow-up duration: 1 yr |
| Scholes 1996  Washington, US | RCT  1 screen offered to selected females  2,607  ROB: unclear (selection, performance and detection biases) | 64%  NR | F  18-34 yrs;  81% ≤24 yrs | 7%; Y | Primary care using telephone recruitment with questionnaire for high-risk considering race, douching, and ≥2 sexual partners in the preceding 12 months; married women excluded | Universal; CT; in-clinic; clinician-collected cervical swabs (EIA or culture); re-testing NR; none | **PID**: Self-report signs, symptoms, dx; medical records; hospital discharge and pharmacy records. Dx had to be recorded and considered “clinical” (37 of 142 reported PID confirmed) but no specific criteria provided. Incidence in CG 2%. Follow-up duration: 1 yr |
| Senok 2005  Location | RCT postal & opportunistic vs usual care over 4-month period  476  ROB: high for attrition bias; unclear for selection and detection | Opportunistic 21%; Postal 48%; UC 0%; NR | F  16-30 yrs; mean 24 yrs | Opportunistic 14%  Postal 5%  Usual care NR; N | Letters from general practice lists | Universal opportunistic & postal; CT; NR; no; incentives to providers at practices | **Treatment for CT:** clinic records. Follow-up duration: 4 mos |
| van den Broek 2012  Netherlands | RCT stepped-wedged cluster  3 annual offered  190 clusters with 317,304 people in three regions  ROB: unclear for performance and detection biases and incomplete outcome data (prevalence); high for incomplete outcome data (positivity) | 16% (1^st^ round), 10% (3^rd^ round) (vs 13% in controls)  NR | F & M  16-29 yrs | 4.3% (7.1% in <20 yr olds); NR | Population-based with postal invite to request sampling kit via internet | Universal; CT; Home with urine for males & vaginal swab or urine for females; kits for re-testing sent 6 mos after CT+ (uptake NR); none | **Estimated population prevalence** using data from positivity with extrapolation to sexually active population of same ages in communities.  Follow-up duration: 2 yrs |
| **Controlled clinical trials** | | | | | | | |
| Clark 2001  US (Army recruits to South Carolina) | CCT  1 screen  28,074  ROB: high (selection and detection bias) | 100%  NR | F  17-39 yrs;  88% ≤25yrs | 9.1%; N (93% of IG but higher and unknown for CG) | Community outreach via non-health Army training examination centre | Universal; CT; on-site self-collected urine with NAAT; re-testing NR; education on STDs | **PID, EP, infertility**: Hospital discharge records. Incidence in CG 5.1/1000 PY (0.8%), 1.9/1000 PY and <0.01/1000 PY. Follow-up duration: mean 1.5 yrs |
| Cohen 1999  Louisiana, US | CCT  Bi-annual screening offered over 2.5 years (CG invited in 3^rd^ year with 1 test period)  5,907 from 3 IG and 5 CG schools  ROB: high for selection, performance (11-53% testing outside of trial), attrition and other (no cluster adjustment) biases | 83% at least once; annually, 52% to 65%  IG & CG: 11% grade 9 and 53% grade 12, males ~20% | F & M  Grade 9-12 | CT: 11.5% females (8.7% in grade 9s vs 14% in grade 12s), 6.2% males  NG: 2.5% females, 1.2% males (similar across ages)  N | Community health in high school health centres | Universal; CT & NG; on-site urine with NAAT; re-testing NR but bi-annual testing; information about risks and consequences | **CT positivity in screening eligible students**, who participated in screening, in IG after year 2 (5 tests offered) and CG (offered screening after year 2 in IG); Follow-up duration: 2.5 yrs  **NG positivity in screening eligible students**, who participated in screening, in IG after year 1 (3 tests offered) and CG (offered screening after 1 year of NG testing added in 2^nd^ year of study). Follow-up duration 1.5 yrs |
| **Observational studies** | | | | | | | |
| Sufrin 2012  California, US | Retrospective cohort  1 screen  57,728  ROB: moderate (selection bias with some adjustment) | 100%  NA | F  14-49 yrs; mean 32 yrs (non-screened 7yrs older) | NR; Y | Primary care | Unknown but assume some form of risk assessment for screening same day or up to 1 yr before IUD insertion; CT; in-clinic but unknown test and methods; re-testing NR; none | **PID after IUD insertion**: Health Maintenance Organization database ICD plus antibiotic pharmacy dispensed, with record review in 10% random sample if discordant; closed system. No specific criteria. Incidence in CG 0.36%. Follow-up duration: 3-15 mos (3 mos after IUD insertion but up to 15 mos from screen) |
| Low 2006  Sweden | Retrospective cohort  48% screened once, 22% twice, 30% ≥3 tests over 10 yr  43,715  ROB: moderate (selection bias with some adjustment) | 100%  NA | F  15-24 yrs | NR (11.5% at some time during follow-up); N | Population-based | Opportunistic used in county; CT; in-clinic sampling NR with culture; none | **PID, EP, infertility**: Hospital discharge in-(all yrs) and out-(last 6 yrs) patient. No criteria. Incidence in CG 2.9%. 1.9%, 3.1% over 10 yrs. Follow-up duration: 10 yrs |

Abbreviations: CCT=controlled clinical trial; CG=control group; CT=Chlamydia trachomatis; Dx=diagnosis; F=females; FSW=female sex workers; ICD=International Classification of Diseases; IG=intervention group; IUD=intrauterine device; M=males; mos=months; NAAT=nucleic acid amplification test; NG=Neisseria gonorrhoeae; NR=not reported; PID=pelvic inflammatory disease; RCT=randomized controlled trial; ROB=risk of bias; wks=weeks; yrs=years

**Key Question 1: Harm outcomes**

| **Author, Year** | **Sex (%F);**  **Age (yr)** | **Sample size; Response rate; Risk status** | **Setting; Screening Methods; Sampling** | **Outcome Assessment** | **Timing of Outcome Assessment; Knowledge of CT status** | **Outcomes** |
| --- | --- | --- | --- | --- | --- | --- |
| Andersson, 2017 | 58  Age: mean 24 (F) and 25 (M); 18-45; ≤23 44% | 128 (CTPos)  77% of eligible; 69% for HADS  **High-risk**  60% of men and 72% of women in this study were risk consumers of alcohol via Alcohol Use Disorder Identification Test | **Primary care**: STI clinic at a university hospital, Sweden  NAAT: urine in men, self-swab vaginal by women  Consecutive sample of pts treated and attending counselling for contact tracing | **Symptoms Questionnaire:** Since I found I had CT I feel – 26 questions (6 only for those with partner), 5pt Likert (5=completely agree; 3=neutral)  Analyzed based on sex, age and relationship status, unadjusted  **Hospital Anxiety and Depression Scale** - 14 statements and 4 answer alternatives (7 about anxiety; 0-21 for each subscale)  Analyzed by sex, unadjusted | After treatment at STI clinic (most of patients) and attending counselling for partner tracing  Known CT status | **CT Diagnosis**   - Anxiety (generic and about infertility) - Shame/Stigma - Embarrassment - Guilt - Relationship distress (break-up and related) |
| Campbell, 2006 | 60  16-39 (87% <26) | All CTNeg  Before invitation (BI)  n=218 (60% response)  At invitation (I)  n=397 (92%)  Receiving Negative Result (NegR)  n=227 (71%)  **General-risk**  Under-deprived areas over-represented in non-responders, but not for ethnicity | **Primary Care**: 27 general practices in the 2 large cities in UK  Home screening w/  urine or vulvo-vaginal  Random sample of each time point, stratified for practice and sex | **Hospital Anxiety and Depression Scale** – 7 item subscales, 0 – 21 score  Analysis across time accounted for age, sex, practice & clustering effects  **Rosenburg Self Esteem Scale** – 10 items, 10 – 40 score (used to measure possible stigma)  Analysis across time accounted for age, sex, practice & clustering effects | Measurements taken at 3 time points – Before invitation (BI) for screening, at invitation (I) and when a negative result (NegR) received.  Known CT status for NegR group only | **CT Screening Invitation/Process & Receiving Results: CT Neg**   - Anxiety (generic) - Shame/Stigma (self-esteem) |
| Fielder, 2013 | 100  18-21; mean 18.1 | 290 (99% CTNeg)  94% of those taking STI test (64% of those in longitudinal health study)  **General-risk**  1% had STI | **Outreach recruitment at university with testing at health centre**: private university, USA  Self-collected vaginal swabs tested for CT, NG & trichomoniasis  All first year students (mass recruitment) | **Participants Subjective Experience with STI screening test; statements on Likert scale from 1 (strongly disagree) to 4 (strongly agree)**  Unadjusted descriptive statistics | Approximately 1 month after test (but asking about testing experience)  Known CT&NG status | **CT & NG Screening Invitation/Process: CT&NG Neg**   - Anxiety about CT - Embarrassment |
| France, 2001 | 100  < 25yrs | 4 (all CTPos)  100% response  **General-risk**  6% CTPos | **Primary Care**: family planning clinic, UK  Selective screening with urine testing at clinic | Questionnaire – completed by 4 CTPos with negative re-test results at 6 months  .  . | 6 months after screening | **CD Diagnosis**   - Anxiety about infertility - Embarrassment |
| Gottlieb, 2011 | 100  ≥16; mean 23yrs;  30+ 16% | CTNeg n=1593 at baseline & 280 at follow-up  (51% response of those eligible)    CTPos n=149 at baseline & 71 at FU  (50% of eligible)  78% baseline participation rate  **High-risk**  4% exposed to STI  7% symptomatic  34% history of CT  8.8% positive for CT  65% Black or African American | **Primary care:**  Midwestern family planning clinics, USA  CT testing as part of annual routine clinic appointment (annual screening in sexually active women who are <25 years of age, have new or multiple sex partners, or a symptomatic partner). Women who are CTPos receive counselling messages about (1) the nature of  chlamydial infection, its complications, and its tendency to  cause asymptomatic infection; (2) the potential for reinfection  and the recommendation to be tested again in 3 to 4 months;  and (3) the importance of partner treatment.  All CTPos not involved in case-control study; CTNeg 4:1 random sample | **Rosenberg Self-Esteem Scale** – 10 items, 4-point scale (range 0-30, higher score higher self-esteem)  **Brief Symptom Inventory** (5 subscales) with anxiety (6 items; 5-points, 0=not at all, 2=a little bit, 3=moderately, 4=quite a bit, 5=extremely)  **Multidimensional Sexual Self-Concept Questionnaire** (3 subscales)– anxiety (5 items; 5 points, 1=strongly disagree, 3=neutral)  **Relationship Stress (Break up) (Yes/No)**  **General thoughts and concerns about CT** (Think about CT a lot, I am concerned about CT) (5 points, 0=strongly disagree, 3=neutral)  **General thoughts and concerns about chlamydia (concerns, think a lot about CT)**  Analyses for above just adjusted for CT status  **Chlamydia-specific concerns** (CTpos at 1 month FU; 26 items; **1=**strongly disagree, disagree, feel neutral, agree, and strongly agree**)** | Baseline questionnaire (during visit for testing) and 4-6 weeks after test results  CT Screening Invitation/Process: no knowledge of CT status | **CT Screening (CTPos & Neg)**   - Anxiety (generic, sexual aspects) - Shame/Stigma - Relationship distress (break-up)   **CT Diagnosis**   - Anxiety (generic, sexual aspects, about CT, about infertility) - Shame/Stigma - Embarrassed - Guilt - Relationship distress (break-up and related) |
| Gotz, 2005 | CTNeg 62  CTPos 82  15-29 yrs | 351  CTPos n= 76  CTNeg n= 275  50% response rate  **General-Risk**  2% CT prevalence, symptoms of other risk factors in CTPos NR  Between group data NR | **Population-based**: municipal health services, Netherlands  Population-based screening program with home based urine CT screening  15-29ys participating in program; NR how selected CTPos; CTNeg random samples | **Experiences with Test Results, questionnaire with open questions, multiple choice or a 5pt scale (1=negative; 5=positive; 4-5 Very much agree or agree)**  Comparisons based on CT status unadjusted | 6-to-12 weeks after results (but about receiving test results)  CT Screening knowledge of CT status | **CT Screening (CTNeg)**   - Anxiety about CT and infertility - Shame/Stigma   **CT Diagnosis**   - Anxiety about CT and infertility - Shame/Stigma - Relationship Distress |
| Kangas, 2006 | 64  18-43; mean 25 | 277  CTPos  n=82  61% response  CTNeg  n=195  53% response  **High-risk**  Only 22% (71/277) were offered test by GP; others sought test for risk-factors (20% for symptoms); 60% of CTPos reported symptoms upon questioning | **Primary care**: general practice, Denmark  Opportunistic screening, part of partner notification, prior to transcervical procedure, symptomatic (20%)  >18yrs and acceptance of GP performing testing; recruited over 3 months all CTPos eligible and 4:1 CTNeg matched for sex | **Questionnaire (19 items)**– stigmatization, partner’s reaction and future reproductive health (fully agreed, somehow agreed [both = agreed”], somehow disagreed, or fully disagreed with the statement). Piloted in 20 people w/out changes  Adjusted for age (18-25 vs 26-43) | Recruited 1 week after test  All knowledge of CT status | **CT Screening (CTNeg)**   - Anxiety about infertility - Shame/Stigma - Embarrassment - Relationship distress   **CT Diagnosis**   - Anxiety about infertility - Shame/Stigma - Embarrassment - Relationship distress |
| Low, 2003 | 64  ≥16 yrs  83% 16-20 | 109 (Both CTPos and Neg)  87% of those participating in health sessions and sexually active (response rates to sessions NR)  **High-risk**  9.2% CTPos; 2% also NGPos; high prevalence community; 86% non-Caucasian; 50% condom use | **Outreach**: further education colleges, inner London  Screening within health promotion intervention with education; urine samples  Screening provided to all regardless of sexual activity (to avoid embarrassment) but results for those sexually active | **Brief questionnaires during health promotion intervention where screening was offered *with education about STIs* via interactive game by sexual health advisor**  Unadjusted for CT status or other variables | During testing session  No knowledge of CT status | **CT Screening Invitation/Process (CTPos & Neg)**   - Embarrassment |
| O’Farrell, 2013 | NR  ≥16 yrs  16 – 61yrs (~70% 16-29) | 298  CTPos n=149  Gender-matched non-STI control n=149  >99% response rate  **High-risk**  CTPos vs nonSTI: same age and education, but more frequently had 2+ partners in past 3 mos 49% vs 28% | **Primary care**: STI clinic,  London  Screened at clinic  Exposed: CTPos and regular partner at diagnosis  Controls: regular STI clinic but no STI at last visit & regular partner | **Questionnaire** – either face to face or over the phone about relationships and physical violence  Comparison between CTPos and nonSTI groups unadjusted | 3-12 months after diagnosis  All aware of CT status | **CT Diagnosis**   - Relationship distress (break-up and physical violence) |
| Walker, 2013 | 100  16 –25yrs | 872 (67 CTPos, 805 CTNeg)  78% participating in CT incidence study with recruitment response 66%  **General-risk**  4.9% prevalence at baseline; more educated and sexually active than general population | **Primary Care**; general practice, sexual health and family planning clinics,  Australia  Repeated screening (q3-6 mos over 12 mos) offers as part of incidence study; urine or self-collected swab (mailed after first baseline)  CTPos telephone consultation about results, treatment, and support if required  16-25 yrs recruited at 29 general practice and sexual health or family planning clinics | **Questionnaires**  **CT DX:** about their experiences (or expectations for CTNeg) of being Dx with CT  **CT Screening**: about their thoughts on future testing  (five-point Likert scale, graded from ‘strongly agree’ to ‘strongly disagree’ or ‘never’ to ‘always’ [4 & 5 combined])  Only comparisons between CTPos and Neg no other adjustments | At end of 12-month study of repeated screening  All aware of CT status | **CT Screening (CTPos & Neg)**   - Embarrassment - Relationship distress   **CT Diagnosis**   - Anxious - Shame/Stigma - Embarrassment - Relationship distress |

Abbreviations: CT=Chlamydia trachomatis; Dx=diagnosis; HADS=Hospital Anxiety and Depression Scale; NAAT=nucleic acid amplification test;

**Key Question 2: Comparative effectiveness of different screening strategies (all home vs clinic sampling)**

| **Author, Year** | **Design; Intensity; Sample size; ROB** | **Uptake of screening in IG & CG; Rates of testing outside of study (IG;CG)** | **Sex** | **Age** | **Baseline CT+ prevalence; all sexually active (Y/N)** | **Recruitment/Patient selection** | **Screening approach; CT vs CT & NG; Location, test and person testing; Re-testing; Co-interventions** | **Outcome assessment** |
| --- | --- | --- | --- | --- | --- | --- | --- | --- |
| Cook 2007 | RCT  Biannual testing for 18 months  190 (half of study population with others all previous recent STI)  ROB: unclear for selection and performance biases | NR but good uptake and higher in home screening; NR | F | 15-24 yrs | 17%*; Y | Outreach with community advertisement with provision of home testing kits (via mail or pick-up) or invite for clinic testing | Universal for high-risk; CT & NG; Home sampling using vaginal swab and NAAT vs. in-clinic swab after postcard invites; NR; No | **Incidence of CT and NG:** positivity at 6, 12, or 18 month follow-up after treatment of CTPos cases at baseline  Follow-up duration: >80% for >24 mos |
| Wilson 2017  United Kingdom | RCT home vs clinic  1 screen  2,072  ROB: unclear for performance bias | IG 50% vs CG 26.6%; both numbers include all testing (IG 43% via mail) | Both; 59% females | 16-30 yrs (evenly distributed) | 2.8%; Y | Outreach via wide community promotion and recruitment online or face-to-face. | Universal via text message with either internet link to postal test and results service or websites to local sexual health clinics (all participants had to attend clinic for treatment); STIs (CT, NG, gonorrhoea, HIV, and syphilis); Home sampling using vaginal swab or urine testing (males; MSM also sent pharyngeal and rectal kits) with NAAT vs clinic testing details NR; None; 10£ incentive | **Treatment of STIs:** self-reported prescriptions confirmed by records (data for CT and NG treatment obtained from author)  Follow-up duration: min 10 mos |
| Reagan 2012  USA | RCT home vs clinic  1 screen  200  ROB: unclear for performance, and detection biases | IG 72% vs CG 48% | M | 18-45 yrs; mean 30 | CT 3.3%; NG 2.5% | Outreach via health clinic and community promotion; those interested called recruiters | Universal home or clinic testing with (treatment at clinic); CT & NG urine testing using NAAT; None; $10 incentive | **Treatment:** “treated at clinic”  Follow-up duration: 10-12 weeks |
| Senok 2005  Scotland | RCT postal vs opportunistic vs usual care over 4-month period  476 (260 in IGs)  ROB: unclear for sequence generation, performance and detection biases | Opportunistic 19%; Postal 38%; UC 0%; NR | F | 16-30 yrs; mean 24 | Opportunistic 14%  Postal 5%; N | Letters from general practice lists | Universal opportunistic vs postal with reminders; CT; NR; No; incentives to providers at practices | **Treatment:** clinic records  Follow-up duration: 4 mos |

Abbreviations: CG=control group; CT=Chlamydia trachomatis; IG=intervention group; NG=Neisseria gonorrhoeae; NR=not reported; RCT=randomized controlled trial; ROB=risk of bias; UC=usual care

* Women needed to meet at least three of the following five criteria associated with an increased risk of acquiring a STD: young age (≤20 years), black race, monthly douching, >1 sexual partner in the past year or living in a neighbourhood that has within the top 33% of chlamydial rates in the county

**Key Question 3: Patient preferences: Health-state utility studies**

| **Author, Year**  **Location** | **Study aims** | **Participants** | **Number of participants** | **Methods for preferences** | **Relevant heath states valued** | **Further information on scenario/health state descriptors** | **Utility values** | **Risk of Bias*** |
| --- | --- | --- | --- | --- | --- | --- | --- | --- |
| Kuppermann 2007  USA | Assess the impact of abnormal uterine bleeding and pelvic pain and pressure on HRQOL and sexual functioning | Women seeking care for noncancerous pelvic problem  Age: mean 42.5; range 31-54 yr  Other:   - 42% Caucasian - 51% symptoms have been somewhat or mostly resolved with treatment - SF12 Physical and Mental functioning scale each 45 on scale 0-100 | 1,493; Pain only=272  By disease history:  PID:56  No PID: 150 | TTO  Patients were asked how many years of their remaining lives they would be willing to give up to live without the symptoms they were experiencing.  Utility for current health = reduced life expectancy with no symptoms / full life  expectancy with symptoms at the point of indifference. | Pelvic pain | None. All patients with condition. No explicit consideration of lessening symptoms over time or other possible complications, but many considering hysterectomy thus impact on fertility this way. | All participants:  range 0.78-0.88  Pelvic pain only group (used for analysis): 0.83 ±0.01 | Fair |
| Smith 2008  USA | Measure quality-of-life utilities for health states associated with PID | Women with and without a history of PID from PID treatment trial & STI clinic  Age  PID: 31.4 ±8.2 yr  No PID: 27.2 ± 9.3 yr  Other:   - Caucasian 25% with PID, 47% without PID - No children 27% with PID, 58% without PID - Self-reported infertility 14% with PID, 1% without PID - 60% wanted children or more children | 206    PID: 56  No PID: 150 | TTO; VAS  TOO: Participants were asked to trade-off between living 10 y in the health state and varying amounts of time in full health until point of indifference  VAS: scale anchored by dead at the bottom (valued at 0%) and perfect health at the top (valued at 100%) | PID OP, PID IP, ectopic pregnancy, infertility, chronic pelvic pain | Participants read scenarios with  information about a 25-year old woman with symptoms associated with the condition, treatment options, complication risks (e.g. small chance of infertility from PID), and functional limitations that might occur as a result of the condition.  Duration: PID (7 days; IP and OP same duration but more severe in IP), ectopic pregnancy short-term state (few weeks); infertility and  chronic pelvic pain long-term; PID and ectopic pregnancy scenarios stated possibility of long-term consequences | **TTO** (PID vs no PID; mean and SD)  PID OP: 0.90 (0.22) and 0.87 (0.26)  PID IP: 0.82 (0.29) and 0.84 (0.27)  Ectopic pregnancy: 0.79 (0.34) and 0.87 (0.26)  Pelvic pain: 0.69 (0.37) and 0.79 (0.29)  Infertility: 0.76 (0.34) and 0.84 (0.29)  **VAS** (PID vs no PID; mean and SD)  PID OP: 0.70 (0.20) and 0.69 (0.17)  PID IP: 0.61 (0.22) and 0.60 (0.20)  Ectopic pregnancy: 0.55 (0.21) and 0.63 (0.22) (p=0.12)  Pelvic pain: 0.45 (0.22) and 0.53 (0.20) (p=0.02)  Infertility: 0.53 (0.29) and 0.66 (0.24) (p=0.003) | Good (Fair for PID and ectopic pregnancy)  Response of eligible participants NR |
| Institute of Medicine 1999  USA | Develop a quantitative model to prioritize the development of vaccines for infectious diseases | Experts and members of committee | NR | Indirect method:  Quality-adjusted weights (using HUI2 having 7 component attributes) multiplied by weights from general health status of population | Cervicitis/bartholinitis, PID OP, PID IPNS, IPS, OPAIP, ectopic pregnancy (IP/OP), infertility, chronic pelvic pain | A committee to study priorities for vaccine development developed scenarios using HUI components (i.e., filled in the tool) for health conditions with input from experts.    Not accounting for duration. | Utility and duration accounted for in economic evaluation:   - Cervicitis 0.9 (4 weeks) - PID OP 0.63 (10 days) - PID IPNS 0.57 (2 days) - PID IPS 0.46 (2 days) & PID AIP 0.83 (10 days) - Ectopic pregnancy OP 0.58 (4 weeks)* - Ectopic pregnancy IP 0.23 (3 days) with 4 weeks recuperation (0.60)* - Chronic pelvic pain 0.60 (remaining lifetime)* - Infertility 0.82 (remaining lifetime)*   *Except for PID and cervicitis, account for getting PID too | Fair  Response of eligible participants NR |
| Trent 2011  USA | Investigate and compare adolescent and parent PID-related health utilities | Adolescents (aged 12-19 y) and their parents (aged 18+ y) recruited from medical and school health clinics in areas of high CT prevalence  Age:  Adolescents 16.2 ± 1.7  Parents 42.6 ± 10  Other:   - 20% Caucasian - 22% history of STI - 4.3% history of PID - About 10% had previous ectopic pregnancy and infertility | 255  Adolescents: 134  Parents: 121 | TTO; VAS  Participants completed a Web-based survey in which they were asked to trade-off between a longer life with a health condition and a shorter life in perfect health. 50 yr timespan | PID OP, PID IP, ectopic pregnancy, infertility, chronic pelvic pain | Scenarios slightly modified from that by Smith et al. for use with adolescents. Except for infertility health states described in 15-yr old.  Parents were responding for their children. | Utilities (Adolescents vs. parents, mean and SD):  **TOO**:  PID OP: 0.82 (0.33) vs 0.902 (0.27)  PID IP: 0.78 (0.36) vs 0.88 (0.30)  Ectopic pregnancy: 0.82 (0.35) vs 0.91 (0.26)  Infertility: 0.84 (0.32) vs. 0.91 (0.25)  Chronic pelvic pain: 0.76 (0.38) vs. 0.85 (0.31)  Except for infertility all lower (p<0.05) for adolescents  **VAS**:  PID OP: 61.95 (24.06) vs 76.24 (23.33)  PID IP: 57.11 (25.13) vs 73.80 (23.09)  Ectopic pregnancy: 55.10 (25.38) vs. 73.47 (23.77)  Infertility: 58.74 (28.62) vs. 68.41 (27.05)  Chronic pelvic pain: 47.85 (25.36) vs. 60.87 (23.78)  All lower (p<0.001) for adolescents | Good (Fair for PID and ectopic pregnancy)  Response of eligible participants NR |

Abbreviations: IP=in-patient; IPNS= in-patient nonsurgical; IPS= in-patient surgical; OP=out-patient; OPAIP=out-patient after in-patient; PID=pelvic inflammatory disease; SD=standard deviation; TTO=time trade off; VAS=visual analogue scale

**Key Question 3: Patient preferences: Non-utility studies**

| **Author Year, Country**  **Study Description**  **Sample Size, Age, Female (%), Ethnicity**  **Screening History**  **Risk Indicators**  **Response Rate**  **Overall Study Quality** | **Outcome Data Provided to Participants; Details of Data Collection Methods; Analysis** | **Weighing of Outcomes** | **Interpretation of Findings & Limitations** |
| --- | --- | --- | --- |
| Balfe 2010, Ireland  Qualitative interviews (telephone or face-to-face) with patients from GP practices and clinics  N=35; 18-29, 100%, NR  Previous screening: NR  Risk status: general-risk  Response rate: NR  Overall quality: good | No outcome data provided  Open-ended questionnaire about offer-of-screening for CT opportunistically during healthcare visits for non-sexual health issues  Thematic analysis to generate explanatory framework | **Reasons for (not) screening**    **Future reproductive health (not specified) vs. stigma (“identity threat”):**  Women reported concerns about their future reproductive health as reasons for accepting screening. However, the stigma of having an STI could outweigh the benefits of testing.   - “It would be just how people would see you...they would get a bad negative picture of you” - ”Men doctors would just look at you like you were a little slut or something”   **Subgroups:**  **Risk status:** Those with higher perceived risk of CT may not screen due to threat of negative influence of their “good girl” identity.  **Younger and those from lower SES & rural locations** had more concerns about stigmatization (i.e., seen as threat to their ‘good girl’ identity, STIs associated with promiscuity, dirtiness and low-class status), especially if peers in their social networks discovered they accepted screening. Women from middle-class and urban settings less concerned about screening and trusted healthcare professionals to maintain confidentiality. | Potential benefits for ‘future reproductive health’ may not outweigh anticipated harms from stigmatization for some women, particularly those younger and from rural areas.  Future health benefits = anticipated harms from stigma (for younger, rural)  Knowledge about specific benefits is unknown; harms were anticipated |
| Barth 2002, US  Qualitative interviews (in-person) with undergraduate students enrolled in first or second summer session at an urban university  N=41, 18-23, 61%, 63% Caucasian  Previous screening: 27% routinely, 61% had never tested for STIs  7% tested when symptomatic; 5% tested due to exposure  Risk status: general-risk (29% with 0 and 25% with >5 previous sexual partners)  Response rate: >50% but unknown because of changing eligibility  Overall quality: fair | No outcome data provided  Face-to-face interviews about students’ knowledge and opinions about STIs, using hypothetical scenarios to elicit factors that are relevant to decision-making  Qualitative content analysis incorporating health belief model, theory or reasoned action & theory of health services utilization | **Factors relevant to decision-making**  **Transmission vs. future health & health benefits (not specified) vs. harms (% respondents that identified each factor):**  10% concern for partner; “You wouldn’t want to infect someone that you cared about. I mean, that would be my nightmare about it”  12%: One’s future might be affected  39% there are health benefits to screening  56% anticipated stigma (“perceived as being ‘loose’, ‘dirty’); 88% about what others would think; 61% embarrassment; 56% anxiety (e.g., “fear of negative results”)  **Subgroups**: Not reported | Potential benefits for future health and transmission were outweighed by anticipated harms from stigmatization and anxiety for many young (<21) women (e.g., 61% had not screened in past but # offered NR).  Harms from stigma & anxiety > health benefits> transmission  Knowledge about specific benefits is unknown; harms were mostly anticipated; no actual behaviours or intentions examined |
| Booth 2013, UK  Paper questionnaire with open-ended questions to students from academic and vocational colleges  N=128; 16-24 (median 17), 49%, diverse ethnic representation  Previous screening: 39% ever tested for CT; 52% had never tested for CT  Risk status: high-risk (71% resided in deprived areas)  Response rate: 85%  Overall quality: fair | No outcome data provided  Self-administered open-ended questionnaire about beliefs regarding CT screening (Getting tested for CT every 12 months…”)  Content analysis using theory of planned behavior (behavioural, control and normative beliefs) | **Beliefs elicited about screening**  **Benefits:**   - ‘Testing can prevent the spread of infection’ (F 17%, M 15%) - I can notify past partners (F 3%, M 0%) - ‘Testing can prevent future health problems’ (F 5%, M 0%)   **Harms:**   - ‘Nothing bad about being tested’ (F 27%, M 23%) - **Stigma/Shame**: ‘People might think badly of me if I get tested’ (F 2%, M 2%); ‘I would feel dirty getting tested’ (F 2%); ‘I would feel embarrassed about getting tested’ (F 8%, M 6%) - **Relationship distress**: Testing could affect their partner/relationship (F 8%, M 3%); ‘My partners would think my testing is a good idea’ (F 29%, M 28%) or bad idea (F 24%, M 11%). - **Anxiety about CT:** Women (8%) and men (9%) worried about getting tested; ‘The worry of having chlamydia’ was a barrier (F 3%, M 3%); ‘Testing could make you worried/paranoid’ (F 2%, M 2%); “I would feel scared about getting tested” (F 5%, M 6%)   **Subgroups**: Females vs males similar responses except for future health and females more worried about relationship distress; all young (93% 16-18 yrs) | Benefits and harms fairly balanced: Transmission>harms>health benefits  Knowledge about specific benefits is unknown; harms were mostly anticipated; no actual behaviours or intentions examined |
| Booth 2015, UK  Self-administered paper questionnaire to students from a vocational college  N=278; 16-24 (mean 17), 47%, 81% Caucasian  Previous screening: 50%  Risk status: high-risk (75% from deprived areas)  Response rate: >95% attending tutorials  Overall quality: good | No outcome data provided  Self-administered questionnaire items on 7-point response scales to measure beliefs and intentions about regular CT testing  Questionnaire data analyzed and correlated to intentions | 7-pt scale: 1=definitely do not/unlikely; 7=definitely do/likely, mean (SD)  **Intentions to screen regularly:** F 4.73 (1.66); M 3.81 (1.69) (p<0.001)  **Benefits:**  **Transmission:** It would help stop the spread of infection: F 6.20 (1.34); M 5.68 (1.70) (p<0.01) Pearson correlation with intentions r=0.32 (strongest behavioral belief)  **Harms:**  **Anxiety about CT:** Being worried or scared about having chlamydia: F 5.89 (1.68); M 5.24 (1.63) (p<0.01) r=0.24  **Related to stigma/shame:** People might find out about me testing: F 3.42 (1.82); M 3.70 (1.71) r=0.26  **Relationship distress**: My partner thinks I should get tested: F 4.89 (2.12); M 4.87 (1.80) r=0.17  (All positive correlations indicating increase in intentions)  **Subgroups**: sex; females intended to screen more and valued transmission | Transmission strongest driver of intentions (and more for females)  Harms from mostly anxiety had *positive* influence on testing  Benefits > harms for women; uncertain for men  Neutral intentions not described well from data  Knowledge about specific benefits is unknown & no question about benefits other than transmission; harms were often anticipated |
| Chacko 2008, US  Cross-sectional study within RCT of motivational interviewing to increase screening for CT & NG; participants were attending the community reproductive health clinic for any reason  N=192, 16-21 (mean 19), 100%, 68% African-American  Previous screening: NR  Risk status: high-risk (52% ever had STI, 18% symptomatic)  Response rate: ~100% of those in RCT but 48% of total eligible in RCT  Overall quality: good | Unclear (all received risk-reduction counselling)  Decisional balance exercise (worksheet of participant-generated pros and cons), one-on-one, facilitated by a counselor  Open & axial coding with thematic analysis of responses obtained based on grounded theory; overall frequency (of responses and people endorsing) for each category was calculated | **Benefits:**  **Prevention/Protection 31% endorsed**: prevention of future STIs, long-term effects, and future pregnancy (“To stop long term effects so I can have babies”)  **Effect on others 33.7% endorsed:** concern for others (including unborn/future baby) and worry about transmission **(**“To protect my partner”; “So I won’t harm anyone else”; “Knowing about better prevention”; “Help prevent STIs in the future”; “Not to harm an unborn child”  **Harms**  **Anxiety: Fear & Aversion 47.3% endorsed (**“Being scared of my results”; “Being nervous waiting for my results”, aversion to the testing procedures)  **Relationship distress: Partner Trust 6.5% endorsed as cons (12% as pro, e.g., helping to build trust if negative test) (**“Partner thinking I was with other people”; “False accusations with partner”; “If I have an STD I would feel lied to”)  **Stigma** not coded but alluded to somewhat in confidentiality category endorsed as con by 9.8% (“People knowing”) | Benefits for transmission and future health (including infertility) > harms from anxiety and relationship distress  Knowledge about specific benefits is uncertain; responses in relation to number accepting screening test NR |
| Reed 2017, US  Qualitative interviews (in-person) with adolescents and separately, parent(s)/guardian(s), who presented to urban EDs for any reason  N=40 (each adolescents and parents), 14-21, 53%, 63% Caucasian/14% Black/2% Other  Previous screening: 35%  Risk status: high-risk (many have poor access to healthcare, 30% tested CTPos in past from ad hoc testing approach)  Response rate: 77% adolescents and 93% parents  Overall quality: good | No outcome data provided  Semi-structured interviews with adolescents vs. parents/guardians about benefits and barriers of offering CT and NG screening (including modality of testing and acceptability of data collection method) to all adolescents in the pediatric ED  Questions developed & framework analysis using content analysis based on behavioral model of health services use and health belief model | **Overall support *for offering* CT & NG screening in EDs: 93% adolescents, 98% parents**  **Benefits:**  **Transmission (benefit reported frequently by both study groups):**   - “Catch it in time before it gets worse or before they can infect someone else” - “Just making sure that stuff doesn’t go untreated and cause other problems or they’re spreading it around” - “I think that could prevent a lot of spread of STDs...” - “It benefits everybody...running around spreading it” - “I think if it can stop the spread of STDs, I think that’s a good idea”   **Improved adolescent long-term health (not specified) (benefit reported by parents only):**   - “Just to prevent long-term effects if a child does have an STD...if not it can really impact their health”   **Harms:**  **Majority of adolescents expressed no concerns.**  **Stigma & embarrassment (by adolescents):** “It’s a lot about embarrassment; they don’t want to make themselves look bad just because they have an STD” | Benefits from reducing transmission and improving health >> harms from embarrassment and stigma  Knowledge about specific benefits is unknown, harms were mostly anticipated; overall acceptance of offers may not represent individual decisions |
| Theunissen 2015, Netherlands  Qualitative interviews (in-person) with participants attending an STI clinic (tested) or young people at a secondary school or university (never tested)  N=23, 16-24 (mean 20), 57%, 96% Dutch  Previous screening: 57% never CT-tested; 43% ever CT-tested (range 1-5 tests prior to study)  Risk status: both general- and high-risk (70% of tested were CT-positive)  Response rate: NR  Overall quality: good | No outcome data provided  Semi-structured interviews about anticipations and experiences of CT testing, disclosure, and encouragement of peers to test  Qualitative content analysis using Framework approach | **Transmission & health:**  Most of the never tested participants indicated they would encourage sex partners to test to prevent transmission or would be concerned about their own or their partner’s health.   - “...if I have it then she can also have it and imagine that she has it and then we do it again, and I’ll have it again”   **Shame/stigma of testing and diagnosis:**  Some tested participants who had a diagnosis expressed feelings of shame.  Never tested and tested participants would not disclose to a broader network (beyond parents, friends, sex partners) due to anticipated shame and stigmatizing reactions, including gossiping and insulting language.   - “I would feel a bit ashamed...didn’t pay attention...haven’t been safe” - “I was really ashamed...that I had caught something like that and hadn’t taken better care” - “...not everyone needs to know that I have it...called a little slut, or have people think that I jump into bed with everyone...”   They would however disclose to those close in their network  Anticipation of stigma by those never testing differed from experiences of those tested who had good experiences.  **Anxiety about CT**: Some of the never tested were afraid of the testing procedure and possibility of positive result.  **Relationship distress**: Some tested participants who had a diagnosis expressed feelings of anger towards their sex partner.  Despite anticipated harms, most never tested said they would test because they valued their own health and that of their peers.  **Subgroups**: never vs ever tested as above | Most participants would test for CT; those with a positive diagnosis would encourage others to test. This was balanced against perceptions of public stigma and associated anticipated shame and stigmatizing reactions (e.g., gossiping and insulting language).  Benefits for health and transmission > stigma > anxiety (more so for those with screening experience who had experienced less harm than anticipated)  Participants perceptions of the health consequences are uncertain |
| Duncan 2001, UK  Qualitative (open-ended) interviews with women **with a current or recent diagnosis of CT** attending a GUM or FP clinic  N=17 (10 GUM; 7 FP), 18-29, 100%, NR  Previous screening: 100% at least once (100% diagnosis)  Risk status: high-risk due to setting & referrals (all had CT infection; 24% attributed symptoms to an infection; many not with long-term partner)  Response rate: 62%  Overall quality: fair | All women reported receiving verbal and/or written information about CT infection, treatment and possible side effects. Most recalled information about infertility (but NR if any absolute risks for infertility from T or reduction from screening presented).  Iterative process with interpretive phenomenological analysis | **Experienced Benefits**  **Female infertility vs. anxiety after diagnosis about infertility:**  Relief that the infection had been diagnosed, but also anxiety about future reproductive morbidity which was exacerbated by the clinical uncertainty about the natural course of CT and prognostic issues related to reproductive effects (e.g. told that may not get PID before infertility). Also anxiety about duration of their infection (unknown) which could impact chances of infertility being caused.   - “I just said I had been told I had this and like how long I have had it and it was possible that it would cause infertility” - “What if I’ve passed this on to somebody else and some other poor woman can’t have children...”   **Experienced Harms from Diagnosis**  **Stigma/Shame: mild self-disgust to distress by 100% & most had some anxiety about disclosing result to family and friends**   - **“**When you go (to the clinic) I felt really dirty because it’s an STD... people like me don’t get these kind of things”; “I always imagined that [the clinic was] ...seedy, seedy people”; “I had dreadful guilt...what if I’ve passed this on to somebody else...”   **Relationship Distress: guilt and regret**   - “I felt just guilt more than anything else, regret....and the fact I’ve harmed [current partner] as well just made it worse”; “He shouted and bawled saying are you trying to say that I gave it to you...so it was very tough to tell him but he was fine” - Did not seriously threaten current relationship of participants; may have been less severe/discounted because many had previous partner to attribute infection to | Women are concerned about perceived stigma of STIs, their future reproductive health (mainly potential infertility) and anxiety regarding notifying partners. A CT diagnosis, regardless of treatment, was worrisome to women because of the clinical uncertainty of infertility and prognostic reproductive effects.  Infertility = harms related to anxiety about infertility > harms from stigma/shame = transmission > relationship distress  Benefits may not outweigh all harms because infertility was most important benefit and uncertainty about the risks for this outcome caused anxiety  All women had diagnosis; some information about outcomes provided but unlikely absolute numbers (e.g., risk for infertility from CT infection); no relation to intention to re-screen |
| Mills 2006, UK  Qualitative interviews (in-person) with participants of a screening program in their homes or on university premises  N=45, 16-39, 58%, 93% Caucasian  Previous screening: 100% at least once  Risk status: general-risk population although 50% participants chosen because CT diagnosis  Response rate: 33% of those contacted with invitation  Overall quality: fair  Balfe | Not reported but answers indicted some knowledge of infertility risk from CT  Interviews about participants’ expectations, experiences, and understanding of the postal CT screening process, including their own general and sexual health, knowledge of sexual health issues.  Analyzed inductively using constant comparison method of grounded theory | No one regretted their decision to be screened.  **Infertility vs anxiety about infertility after diagnosis:**  Women expressed sense of relief at having identified their infection sooner rather than later because they thought it minimized the risk of infertility.  A third of women who tested positive were significantly concerned about the possibility of being infertile, and distressed by their unanswered questions.   - “I know it can affect your chances of having children and no one said anything about how I could find out or...you have to wait and see...hanging over my head...may never be able to have children now” - “It can make you infertile...I’m absolutely petrified I can’t have kids...my main concern at the moment”   **Harms:**  **Anxiety about screening and CT diagnosis:**  Anxiety about all stages of screening programme, but fewer and transient after invitation (some) and while waiting for results (several) vs more and severe after receiving diagnosis (majority):   - (after invitation; for some) “A bit apprehensive...makes you think have I got something or haven’t I...makes you sort of wonder a bit” - (after diagnosis) “I was absolutely devastated...just so upset”   **Stigma about screening and CT diagnosis:**  Stigma only “hinted” at around the invitation, but clearly evident after diagnosis and more upsetting for women than men. Men viewed telling others as a form of entertainment. For many women, having CT altered how they felt about themselves and thought others would see them and that people who contracted STIs were labelled ‘slappers, ‘tarts’ and thought of as ‘dirty’:   - “it made me feel dirty... only dirty people get things like that, that sleep about... you’re like a tart” - Most women described ‘felt stigma’ – feeling of shame and fear of discrimination, rather than actual passing of judgment, ‘enacted stigma’; in many cases the opposite happened with partners and family largely supportive   **Embarrassment about CT testing:**   - “It [arrival of a study pack] was a bit embarrassing...that everyone else saw it...”   **Relationship distress from CT diagnosis:**  One of the most fear-inducing consequences of a diagnosis was need to inform current partner. More men than women expressed concern about need to inform current and recent sexual partners, primarily because they thought their partners would be upset:   - “I was absolutely petrified he was gonna turn round and say what have you got? Who have you slept with? I really didn’t know how he was gonna react” - In most cases, their partners’ reactions were not as bad as expected. | For females: Benefits from infertility > harms of diagnosis >> harms from screening  For men: unclear  Uncertain about knowledge of absolute risks for infertility; all participants had screened at least once |
| Nielsen 2017, Sweden  Qualitative interviews (in-person) with participants **repeatedly testing** at a Youth Health Clinic (STI clinic)  N=15, 18-22, 53%, NR  Previous screening: 100% at least twice in 6 months  Risk status: high-risk (all screened 2+ times over 6 months); participants 56% CT-positive; 44% CT-negative (not purposively chosen)  Response rate: NR  Overall quality: poor | No outcome data provided  Semi-structured open-ended interviews about motives for repeat CT testing and how testing affects their sexual risk-taking  Informed by Model of Health Service Utilization framework and data analysis using constructivist grounded theory with iterative process | **Social consequences of CT much more serious than medical consequences**  **Health consequences (unspecified) were considered severe if CT left over long-term but could be avoided or minimized if caught early**  **Transmission important but because of the psychosocial consequences:**   - Reducing anxiety related to the risk of spreading CT - Avoiding social stigma: “To infect someone is...most embarrassing thing you can do. People will see you as revolting…as dirty…as a whore” - All interviewees spoke of shame from infecting someone else (much less I they did not know them).   Participants were motivated to undergo repeat testing for CT mainly by fears/anxiety related to social stigma of transmission. | Harms of diagnosis > benefits but encouraged repeat testing to alleviate  Participants perceptions of the health consequences are uncertain, all participants are repeat testers |

Abbreviations: CT=Chlamydia trachomatis; GUM=genitourinary medicine; IG=intervention group; NG=Neisseria gonorrhoeae; NR=not reported; RCT=randomized controlled trial; STI=sexually transmitted infection
