# Supplementary material for: Screening for chlamydia and/or gonorrhea in primary health care: systematic reviews on effectiveness and patient preferences
Source: Syst Rev. 2021 Apr 19;10:118. doi: 10.1186/s13643-021-01658-w (PMC8056106; doi:10.1186/s13643-021-01658-w)
Supplement: Supplementary file 5 — Additional file 5. Risk of bias assessments. [file 13643_2021_1658_MOESM5_ESM.docx]

**Additional file 5: Risk of bias assessments**

**Key Question 1: Screening versus no screening**

**Benefits.** For PID, one RCT was considered at low risk of bias (only trial with blinding) (28). Three were considered as having unclear risk of bias (see main manuscript reference list: 8, 59, 108), in the domains of detection bias in all three (from provider awareness of the CT results when making decisions about PID diagnosis), performance bias in two (8, 108) (with control arms aware of their allocation and screening more often than expected), and selection bias in one (no allocation of concealment) (108). The CCT by Clark was at high risk due to lack of sequence generation and selective participation in the screening arm, as well as unclear detection bias (awareness of CT status in intervention arm) (88). The RCT by Ostergaard was at high risk due to attrition (>50%) and lack of adjustment for clustering, as well as unclear risk for selection (randomization before enrollment and many fewer participating) and performance biases (105). For ectopic pregnancy and infertility where Anderson and Clark were the relevant trials, the risk of bias changed in Anderson from being unclear due to concerns about detection bias to low because of less concern for this domain with these outcomes (ascertainment of these outcomes would not be as influenced as PID by knowledge of CT status). Neither of the two cohort studies by Low (101) and Sufrin (111) reporting on clinical outcomes demonstrated comparability between groups for important confounders including sexual behaviors and other risk factors. One cohort study was suspected to have selectively reported their analysis (101).

For transmission, three RCTs (8, 9, 60) were at unclear risk of bias in the domains of performance bias (all three), attrition bias (in two (8, 9)), and detection bias (in one (9)). Two trials had high risk of bias. The CCT by Cohen was assessed as unclear rather than high risk for selection bias, because the cluster design and retrospective inclusion of demographically matched control sites was thought to guard to some degree against bias, but was at high risk for performance bias and failure to adjust for clustering (89). The RCT by Hodgins was at high risk for attrition and other (lack of cluster adjustment) biases, and unclear for all other domains (12).

**Coding: Green = low, yellow = unclear, red = high risk of bias**

**Pelvic inflammatory disease (PID), trials**

|  | **Selection Bias** | | **Performance Bias** | **Detection Bias** | **Attrition Bias** | **Reporting Bias** | **Other Bias** |
| --- | --- | --- | --- | --- | --- | --- | --- |
|  | Random sequence generation | Allocation concealment | Evidence of contamination? | Awareness if PID status? | >10-20% |  | Adjustment for clustering? |
| Andersen 2011  (RCT) |  |  |  |  |  |  |  |
| Clark 2001  (CCT) |  |  |  |  |  |  |  |
| Hocking 2019  (cluster RCT) |  |  |  |  |  |  |  |
| Oakeshott 2010  (RCT) |  |  |  |  |  |  |  |
| Ostergaard 2002  (RCT) |  |  |  |  |  |  |  |
| Scholes 1996  (RCT) |  |  |  |  |  |  |  |

**Ectopic pregnancy (EP) & female infertility, trials**

|  | **Selection Bias** | | **Performance Bias** | | **Detection Bias** | | **Attrition Bias** | | **Reporting Bias** | **Other Bias** |
| --- | --- | --- | --- | --- | --- | --- | --- | --- | --- | --- |
|  | Random sequence generation | Allocation concealment | Ectopic pregnancy | Female infertility | Ectopic pregnancy | Female infertility | Ectopic pregnancy | Female infertility |  | Adjustment for clustering? |
| Andersen 2011  (RCT) |  |  |  |  |  |  |  |  |  |  |
| Clark 2001  (CCT) |  |  |  |  |  |  |  |  |  |  |

**PID, EP & infertility (females), observational studies**

|  | **Selection Bias**  **(max. 4 stars)** | | | | **Comparability**  **(max. 2 stars)** | **Outcome**  **(max. 3 stars)** | | | **OVERALL**  **(max. 8 stars)** | **Selective Outcome Reporting†** |
| --- | --- | --- | --- | --- | --- | --- | --- | --- | --- | --- |
|  | 1. Representativeness | 1. Selection of non-exposed cohort | 1. Ascertainment of exposure | 1. Demonstration that outcome not present at start of study | 1. Comparability based on design or analysis | 1. Assessment of outcome | 1. Follow-up long enough for outcomes to occur | 1. Adequacy of follow-up cohorts |  |  |
| Low 2006 | NA | * | * | * | * | * | * | * | **LOW**  **(7 out of 8)** | **Suspected** |
| Sufrin 2012 | NA | * | * | * | * | * | * | - | **UNCLEAR**  **(6 out of 8)** | **Not suspected** |

† Assessed due to concerns regarding reporting bias in the study (Low), but this additional domain is not included in the overall score

**Transmission using prevalence of CT and/or NG, trials**

All studies estimated prevalence using representative samples or analytc methods, with the exception of Cohen.

|  | **Selection Bias** | | **Performance Bias** | | **Detection Bias** | **Attrition Bias** | **Reporting Bias** | **Other Bias (no adjustment for clustering)** |
| --- | --- | --- | --- | --- | --- | --- | --- | --- |
|  | Random sequence generation | Allocation concealment |  | |  |  |  |  |
| Cohen 1999  (cluster CCT) |  |  | M* | F* |  |  |  |  |
| Garcia 2012  (cluster RCT) |  |  |  | |  |  |  |  |
| Hocking 2019  (cluster RCT) |  |  |  | |  |  |  |  |
| Hodgins 2002  (cluster RCT) |  |  |  | |  |  |  |  |
| van den Broek  2012  (cluster RCT) |  |  |  | |  |  |  |  |

***for females this is considered high because of more likelihood of contamination via high rates (11-53% depending on age) of testing outside of the trial (NR by group)**

**` Harms.** For the harms of screening, four studies were rated as low risk of bias (adjusting/accounting for some but not all important confounders) (94, 95, 97, 114) and three as unclear risk (additional concerns about follow-up duration (86), ascertainment of exposure (92), and no accounting for confounders (100)). For the harms of a CT diagnosis that were attributed by the authors to the diagnosis (e.g., relationship distress after the diagnosis), six were at low risk of bias (81, 93, 95, 97, 104, 114) and one was at unclear risk (no adjustment for confounders (94)). Of the two studies reporting on non-specific harms from the diagnosis (e.g., general anxiety or self-esteem), one was at low risk from proving comparisons from before the diagnosis and with CT negative people (94), while the other was at high risk for providing no comparison, having short-term follow-up for this outcome, and for lack of adequate data (81).

**CT Screening for non-specific outcomes (e.g., generalized anxiety, self-esteem)**

| **Study** | **Selection of the non exposed cohort (i.e. before screening)** | **Ascertainment of exposure** | **Analysis or adjustment for confounders**  **(2 stars)** | **Assessment of outcome** | **Was follow-up long enough for outcomes to occur** | **Adequacy of data** | **Total** |
| --- | --- | --- | --- | --- | --- | --- | --- |
| Campbell 2006 | * | * | * | * | 0 | * | 5/7 |
| Gottlieb 2011 | * | * | * | * | * | * | 6/7 |

**CT Screening for outcomes attributed to screening process (e.g., anxiety about infertility, partner break-up)**

| **Study** | **Ascertainment of exposure** | **Analysis or adjustment for confounders**  **(2 stars)** | **Assessment of outcome** | **Was follow-up long enough for outcomes to occur** | **Adequacy of data** | **Total** |
| --- | --- | --- | --- | --- | --- | --- |
| Fielder 2012 | 0 | * | * | * | * | 4/6 |
| Gotz 2005 | * | * | * | * | * | 5/6 |
| Kangas 2006 | * | ** | * | *^1^ | * | 6/6^1^ |
| Low 2003 | * | 0 | * | * | * | 4/6 |
| Walker 2013 | * | ** | * | * | * | 6/6 |

^1^ Adequacy of follow-up was not sufficient for relationship break-up (questionnaire 1 week after testing)

**CT Diagnosis for non-specific outcomes (e.g., general anxiety, self-esteem)**

| **Study** | **Ascertainment of exposure** | **Comparison provided from before Dx or with CTNeg participants** | **Analysis or adjustment for confounders (2 stars)** | **Assessment of outcome** | **Was follow-up long enough for outcomes to occur** | **Adequacy of data** | **Total (max 7)** |
| --- | --- | --- | --- | --- | --- | --- | --- |
| Andersson 2017 | * | 0 | * | * | 0 | 0 | 3/7 |
| Gottlieb 2011 | * | * | * | * | * | * | 6/7 |

**CT Diagnosis for outcomes attributed to diagnosis**

| **Study** | **Ascertainment of exposure** | **Analysis or adjustment for confounders**  **(2 stars)** | **Assessment of outcome** | **Was follow-up long enough for outcomes to occur** | **Adequacy of data** | **Total (max 6)** |
| --- | --- | --- | --- | --- | --- | --- |
| Andersson 2017 | * | ** | * | *^1^ | 0 | 5/6^1^ |
| France 2001 | * | * | * | * | * | 5/6 |
| Gottlieb 2011 | * | 0 | * | * | * | 4/6 |
| Gotz 2005 | * | * | * | * | * | 5/6 |
| Kangas 2006 | * | ** | * | *^1^ | * | 6/6 |
| O’Farrell 2013 | * | * | * | * | * | 5/6 |
| Walker 2013 | * | ** | * | * | * | 6/6 |

^1^ Follow-up was not adequate for outcomes of relationship break-up (Andersson and Kangas) or thinking about the way their friends see them (Andersson) (questionnaire during partner notification counselling [Andersson] or 1 week after test [Kangas])

**Key Question 2: Comparative effectiveness of different screening strategies**

**Transmission via incidence, trials**

|  | **Selection Bias** | | **Performance Bias** | **Detection Bias** | **Attrition Bias** | **Reporting Bias** | **Other Bias** |
| --- | --- | --- | --- | --- | --- | --- | --- |
|  | Random sequence generation | Allocation concealment | Prevalence - positivity | Prevalence - positivity | Prevalence - positivity |  |  |
| Cook 2007  (RCT) |  |  |  |  |  |  |  |

**Transmission via treatment, trials**

|  | **Selection Bias** | | **Performance Bias** | **Detection Bias** | **Attrition Bias** | **Reporting Bias** | **Other Bias** |
| --- | --- | --- | --- | --- | --- | --- | --- |
|  | **Random sequence generation** | **Allocation concealment** |  |  |  |  |  |
| Reagan 2012  (RCT) |  |  |  |  |  |  |  |
| Senok 2005  (RCT) |  |  |  |  |  |  |  |
| Wilson 2017  (RCT) |  |  |  |  |  |  |  |

**Key Questions 3: Relative importance of outcomes**

Of the studies reporting utilities, Smith (110) and Trent (113) were at low risk of bias for chronic pelvic pain and infertility. For ectopic pregnancy, there was concern that the scenario used to inform the TTO exercise underestimated the potential impact of this condition. For PID, there were concerns about using TTO methods for temporary heath states (i.e., the method assumes death follows the health state which is unrealistic for temporary states (119)). Kupperman (99) was considered to have unclear risk of bias due to lack of comparison of chronic pelvic pain with other outcomes of interest, and because of the participants experience with a chronic condition (with associated adaptations) as well as, for several, recent symptom resolution, both of which can lead to an underestimation of the health loss associated with the condition (132). The study by the IOM had high risk of bias due to suboptimal selection of participants (experts representing patient perspective) and lack of reporting any measurement of variance of the findings (96).

The survey by Booth in 2015 was at low risk of bias (85). The nine qualitative studies were of low risk in most domains, except for the relevance of the methods to our research question. Although the data addressed the authors’ purposes of eliciting views, for this KQ the lack of informed views (e.g., answers based on knowledge on potential benefits of screening and their risks) makes interpretations very difficult. Moreover, several studies lacked descriptions of how much the perceived outcomes were thought to influence screening intentions or behaviors (a main indirect indication of the relative importance of benefits and harms in these studies). Three studies were rated as high risk for this domain (83, 84, 103); studies with unclear risk either provided some information to participants (e.g., on risk for infertility but unlikely absolute magnitude of risk from CT) (87, 91, 102) or accounted for intentions or overall acceptability to screen to some extent (82, 107, 112).

**Utility studies**

| **Study** | **Selection of (representative) participants** | **Appropriate administration and choice of instrument** | **Analysis and presentation of methods and results (completeness of data)** | **Instrument-described health state presentation (of all relevant outcomes and valid with respect to health state)** | **Patient understanding** | **Subgroup analysis to explore heterogeneity** |
| --- | --- | --- | --- | --- | --- | --- |
| Kupperman 2007 | Unclear (46% of eligible enrolled; older than average age of screening participants; 51% had had at least partial symptom resolution which may overestimate utility) | Low (TTO described and administered appropriately) | Low (complete results) | Unclear (not all outcomes considered) | Low (patients had experienced outcome) | Unclear (severity/presence of current symptoms may have been relevant) |
| Smith 2008 | Unclear (response of eligible NR) | Low except for PID where some concern about using TTO for short-term heath states (terminal status of the temporary health state, caused by subsequent death) | Low (complete results) | Low except for ectopic pregnancy where (although reviewed by PID experts) health state presentation considered to misrepresent serious and severity in some cases | Low (in-depth scenarios using understandable language) | Low (previous experience explored) |
| Trent 2011 | Unclear (response of eligible NR) | Low except for PID where some concern about using TTO for short-term heath states (terminal status of the temporary health state, caused by subsequent death) | Low (complete results) | Low except for ectopic pregnancy where (although reviewed by PID experts) health state presentation considered to misrepresent serious and severity in some cases | Low (in-depth scenarios using understandable language) | Unclear (may have been relevant to history of STI in 22%) |
| Institute of Medicine 1999 | High (use of experts and members of committee) | Low (indirect nature of methods for patient utilities accounted for in indirectness GRADE domain) | Unclear (variance of estimates NR) | Low except for ectopic pregnancy where (although reviewed by PID experts) health state presentation considered to misrepresent serious and severity in some cases | Low | Unclear (characteristics NR) |

**Qualitative studies**

| **Study** | **Was there a clear statement of the aims of the research?** | **Is a qualitative methodology appropriate?** | **Was the research design appropriate to address the aims of the research?** | **Was the recruitment strategy appropriate to the aims of the research?*** | **Was the data collected in a way that addressed the research issue?**** | **Has the relationship between researcher and participants been adequately considered?** | **Have ethical issues been taken into consideration?** | **Was the data analysis sufficiently rigorous?** | **Is there a clear statement of findings?** |
| --- | --- | --- | --- | --- | --- | --- | --- | --- | --- |
| **Populations considering screening** | | | | | | | |  |  |
| Balfe, 2010 | Y | Y | Y | Y | U | Y | Y | Y | Y |
| Barth 2002 | Y | Y | Y | Y | N | U | Y | Y | Y |
| Booth 2012 | Y | Y | Y | Y | N | U | Y | Y | Y |
| Chako 2008 | Y | Y | Y | Y | U | U | Y | Y | Y |
| Reed 2017 | Y | Y | Y | Y | U | U | Y | Y | Y |
| Theunissen 2015 | Y | Y | Y | Y | U | U | Y | Y | Y |
| **Screened populations** | | | | | | | | | |
| Duncan 2001 | Y | Y | Y | U | U | U | Y | Y | Y |
| Mills 2006 | Y | Y | Y | U | U | U | Y | Y | Y |
| Nielsen 2017 | Y | Y | Y | U | N | U | Y | Y | Y |

Notes: The data addressed the author’s purpose of eliciting views but for this review the lack of informed views (e.g. having knowledge on potential benefits of screening) and an explicit incorporation of stated or revealed intentions/behaviors makes interpretation very difficult. Studies with unclear risk either provided some information to participants (e.g., on risk for infertility but unlikely absolute risk from CT)(Chako, Duncan, Mills) or accounted for intentions to screen to some extent (Balfe, Reed, Theunissen)

**Cross-sectional study**

| **Study** | **Did the study address a clearly focused question/ issue?** | **Is the research method appropriate for answering the research question?** | **Is the method of selection of the subjects clearly described?** | **Was the sample of subjects representative with regard to the population to which the findings will be referred?** | **Was the sample size based on pre-study considerations of statistical power?** | **Was a satisfactory response rate achieved?** | **Are the measurements likely to be valid and reliable?** | **Was the statistical significance assessed?** | **Are confidence intervals given for the main results?** | **Have confounding factors been accounted for?** | **Can the results be applied to your organization?** |
| --- | --- | --- | --- | --- | --- | --- | --- | --- | --- | --- | --- |
| **Booth 2015** | **Y** | **Y** | **Y** | **Y** | **Y** | **Y** | **Y** | **Y** | **Y** | **Y** | **Y** |
