# Supplementary material for: Screening for chlamydia and/or gonorrhea in primary health care: systematic reviews on effectiveness and patient preferences
Source: Syst Rev. 2021 Apr 19;10:118. doi: 10.1186/s13643-021-01658-w (PMC8056106; doi:10.1186/s13643-021-01658-w)
Supplement: Supplementary file 6 — Additional file 6. Evidence tables. [file 13643_2021_1658_MOESM6_ESM.docx]

**Additonal file 6: Evidence Tables**

**Contents**

| **Evidence Sets** | **Page** |
| --- | --- |
| Evidence Set 1: Screening vs. no screening; Pelvic Inflammatory Disease (PID; All-cause) (Offer of screening- All eligible participants and selected individuals)(16-29 year-olds) | 2 |
| Evidence Set 2: Screening vs. no screening; Pelvic Inflammatory Disease (PID; All-cause) (Acceptors of screening)(15-29 year-olds) | 8 |
| Evidence Set 3: Screening vs. no screening; Ectopic Pregnancy (All-cause)(Offer to screen and acceptors of screening) (17-29 yrs) | 13 |
| Evidence Set 4: Screening vs. no screening; Infertility (All-cause) (Offer to screen and acceptors of screening) (17-29 yrs) | 16 |
| Evidence Set 5: Screening vs. no screening; Transmission of CT: Population prevalence | 19 |
| Evidence Set 6: Screening vs. no screening; Transmission of NG: Population prevalence | 26 |
| Evidence Set 7: Screening vs. no screening; Transmission of CT & NG: Treatment as surrogate (indirect) outcome (# treated/# randomized) | 29 |
| Evidence Set 8: Harms of CT Screening | 32 |
| Evidence Set 9: Harms of CT Diagnosis | 39 |
| Evidence Set 10 Different screening approaches; Transmission of CT & NG: Incidence of CT & NG | 46 |
| Evidence Set 11 Different screening approaches; Transmission of CT & NG: Treatment as surrogate (indirect) outcome (# treated/# randomized) | 47 |
| Evidence Set 12 Patient preferences (relative importance for outcomes): health-state utility values | 52 |
| Evidence Set 13 Patient preferences (relative importance on benefits versus harms): non-utility studies | 57 |

**Evidence Set 1: Screening vs. no screening; Pelvic Inflammatory Disease (PID; All-cause) (Offer to screen - All eligible & selected individuals in a screening program)(16-29 year-olds)**

Included studies: Trials: All eligible: Hocking 2018 & Andersen 2011; Selected individuals: Scholes 1996

Threshold for important effect: 2.5 per 1000 fewer [benefit] or more [harm]

**1A GRADE Evidence Profile**

| **Population** | **Certainty assessment** | | | | | | | **№ of patients** | | **Effect** | | **Certainty** |
| --- | --- | --- | --- | --- | --- | --- | --- | --- | --- | --- | --- | --- |
|  | **№ of studies** | **Study design** | **Risk of bias** | **Inconsistency** | **Indirectness** | **Imprecision** | **Other considerations** | **Screening** | **Usual care** | **Relative (95% CI)** | **Absolute (95% CI)** |  |
| **Offer to screen - All eligible participants in a screening program – All-cause PID** | | | | | | | | | | | | |
| **Median control event rate (0.5%)** | 2 | RCT | No concerns | No concerns | -1.5^a^ | No concerns | No concerns | 69519 | 71843 | 1.01 (0.72 to 1.40) | 0.1 more in 1000 (2.1 fewer to 1.5 more) | ⊕⊕⊖⊖-⊕⊕⊕⊖  LOW-TO-MODERATE |
| **General-risk population (2.7%)** | 2 | RCT | No concerns | No concerns | -1.5^a^ | -1.5^b^ | No concerns | 69519 | 71843 | 1.01 (0.72 to 1.40) | 0.3 more in 1000 (7.6 fewer to 11 more) | ⊕⊖⊖⊖  VERY LOW |
| **High-risk population (4.7%)** | 2 | RCT | No concerns | No concerns | -2.0^a,c^ | -1.5^b^ | No concerns | 69519 | 71843 | 1.01 (0.72 to 1.40) | 0.5 more in 1000 (13.1 fewer to 18.7 more) | ⊕⊖⊖⊖  VERY LOW |
| **Offer to screen - All eligible participants in a screening program – hospitalization for PID** | | | | | | | | | | | | |
| **Control event rate (0.38%)** | 1 | RCT | No concerns | No concerns | -2.0^d^ | No concerns^e^ | No concerns | 23,527 | 23,219 | 0.6 (0.4 to 1.0) | 1.52 fewer per 1000 (2.3 to 0 fewer) | ⊕⊕⊖⊖  LOW |
| **General-risk population (2.7%)*** | 1 | RCT | No concerns | -1.0^f^ | -1.5^g^ | -0.5^h^ | No concerns | 23,527 | 23,219 | 0.6 (0.4 to 1.0) | 10.8 fewer per 1000 (16.2 to 0 fewer) | ⊕⊖⊖⊖  VERY LOW |
| **High-risk population (4.7%)^†^** | 1 | RCT | No concerns | -1.0^f^ | -2.0^c, g^ | -0.5^h^ | No concerns | 23,527 | 23,219 | 0.6 (0.4 to 1.0) | 18.8 fewer per 1000 (28.2 to 0 fewer) | ⊕⊖⊖⊖  VERY LOW |
| **Scholes et al. – Offer to screen - selected individuals – All-cause PID** | | | | | | | | | | | | |
| **Control event rate (2.1%)** | 1 | RCT | -0.5^i^ | No concerns^j^ | No concerns^k^ | -1.0^l^ | No concerns | 1009 | 1598 | 0.43 (0.21 to 0.89) | 11.8 fewer per 1000 (2.3 to 16.3 fewer) | ⊕⊕⊖⊖-⊕⊕⊕⊖  LOW-TO-MODERATE |
| **General-risk population (2.7%)*** | 1 | RCT | -0.5^i^ | No concerns^j^ | No concerns^k^ | -1.0^l^ | No concerns | 1009 | 1598 | 0.43 (0.21 to 0.89) | 15.4 fewer per 1000 (3 to 21.3 fewer) | ⊕⊕⊖⊖-⊕⊕⊕⊖  LOW-TO-MODERATE |
| **High-risk population (4.7%)^†^** | 1 | RCT | -0.5^i^ | No concerns^j^ | -0.5^c,k^ | -1.0^l^ | No concerns | 1009 | 1598 | 0.43 (0.21 to 0.89) | 26.8 fewer per 1000 (5.2 to 37.1 fewer) | ⊕⊕⊖⊖  LOW |

Abbreviations: CI=confidence interval; CT= chlamydia trachomatis; MID=minimally important difference; PID= pelvic inflammatory disease; RCT= randomized controlled trial; ROB= risk of bias

*The absolute effects in all-cause PID for the general-risk population assumed that approximately 6% of the female population would have CT (prevalence), and that about 13% of these females would develop PID (0.78%), and that approximately 25-30% of all-cause PID is attributed to CT (all cause PID = 3.5 times PID from CT); 0.78% x 3.5 = 2.7%.

†The absolute effects in all-cause PID for the high-risk population assumed a higher (12%) CT prevalence and slightly higher (33%) attribution to CT (1.56% x 3 =4.7%).

**Explanations:**

^a^ Indirectness: Serious concerns about lack of complete PID ascertainment (Andersen used hospital diagnoses and doxycycline prescriptions; Hocking only used clinic charts; PID assessed in some ineligible [i.e. not sexually active] individuals), use of population registers in Andersen whch may not reflect practice in primary care, and use of usual care (rather than no screening) comparisons which may have underestimated the effects.

^b^ Imprecision: Sample size adequate but 95% CIs cross both benefit (2.5 fewer) and harm (2.5 more) thresholds.

^c^ Indirectness of high-risk estimate: Added concerns about using RR and natural history data from studies in general-risk population to estimate the effects of screening in high-risk populations.

^d^ Indirectness: Very serious concerns about lack of PID ascertainment (hospital data in different source population from trial and only capturing 21% of PID cases if assuming each case of PID is in a unique individual), and use of usual care (rather than no screening) comparison which may have underestimated the effects.

**^e^** Imprecision: No concerns; sample size adequate and entire 95% CI does not meet threshold for important effect.

^f^ Inconsistency: Serious concerns because inconsistent with other analysis (clinic PID) showing effects below threshold.

^g^ Indirectness: Serious concerns about lack of PID ascertainment. Use of usual care control group (rather than no screening) that underwent some screening, but we did not rate down because believe that this would have dampened the effects such the true effect with a no screening control would still surpass the MID threshold.

^h^ Imprecision: Sample size adequate but entire 95% CI does not surpass the threshold.

^i^ Risk of bias: Some concerns about unclear ROB for selection, performance, and detection biases.

^j^ Inconsistency: Only study in analysis but findings are similar to those for acceptors of screening (see Evidence Set 2) and this study had fairly high rates of acceptance to the screening.

^k^ Indirectness: Use of usual care control group (rather than no screening) that would have undergone some degree of testing in asymptomatic cases, but did not rate down because believe that this would have dampened the effects such the true effect with a no screening control would still surpass the MID threshold; outcome ascertainment and applicability of setting is good in this study.

^l^ Imprecision: 95% CI indicates benefit but sample size is small for rare outcome.

**1B GRADE Summary of Findings**

| **Outcome**  **No. participants (studies)** | **Relative effect (95% CI)** | **Anticipated absolute effects (95% CI)** | | | **Certainty of the evidence (GRADE)** | **What happens?** |
| --- | --- | --- | --- | --- | --- | --- |
|  |  | **Without screening*†** | **With a single CT screen** | **Difference** |  |  |
| **Offer to screen - All eligible participants in a screening program – All-cause PID** | | | | | | |
| All-cause PID (Eligible participants)  Follow-up: 12-36 mos  141,362 16-29 yrs (2 RCTs) | 1.01 (0.72 to 1.40) | Median control event rate (5 per 1000) | | | ⊕⊕⊖⊖-⊕⊕⊕⊖  LOW-TO-MODERATE  (Median control event rate with low PID prevalence)^a^ due to indirectness  ⊕⊖⊖⊖  VERY LOW  (General- and high-risk population estimates)^a-c^ due to indirectness and imprecision | Offering screening for CT via opportunistic or population-based approaches to all females 16-29 years old in general- or high-risk populations may make little to no difference in risk of all-cause PID (general risk: 0.3 more in 1000 [7.6 fewer to 11 more]; high-risk: 0.5 more in 1000 [13.1 fewer to 18.7 more]), but the evidence is very uncertain. |
|  |  | 5 per 1000 | 5.1 per 1000 (2.9 to 6.5) | 0.1 more in 1000 (2.1 fewer to 1.5 more) |  |  |
|  |  | General-risk population^†^ | | |  |  |
|  |  | 27 per 1000 | 27.3 per 1000 (19.4 to 38) | 0.3 more in 1000 (7.6 fewer to 11 more) |  |  |
|  |  | High-risk population^‡^ | | |  |  |
|  |  | 47 per 1000 | 47.5 per 1000 (33.9 to 65.7) | 0.5 more in 1000 (13.1 fewer to 18.7 more) |  |  |
| **Offer to screen - All eligible participants in a screening program – All-cause PID using hospitalization data** | | | | | | |
| All-cause PID (Eligible participants) using data on hospitalizations attributed to PID  Follow-up: 36 mos  46,746 16-29 yrs (1 RCT) | 0.6 (0.4 to 1.0) | Median control event rate (5 per 1000) | | | ⊕⊕⊖⊖  LOW  (Control event rate with low PID prevalence)^d,e^ due to very serious indirectness  ⊕⊖⊖⊖  VERY LOW  (General- and high-risk population estimates)^c, f-h^ due to serious indirectness and inconsistency, and some imprecision | Offering screening for CT via opportunistic approaches to all females 16-29 years old at general- or high-risk for CT may reduce all-cause PID (general risk: 10.8 fewer per 1000 [16.2 to 0 fewer]; high risk: 18.8 fewer per 1000 [28.2 to 0 fewer]), but the evidence is very uncertain. |
|  |  | 3.8 per 1000 | 2.3 per 1000 (1.5 to 3.8) | 1.52 fewer per 1000 (2.3 to 0 fewer) |  |  |
|  |  | General-risk population^†^ | | |  |  |
|  |  | 27 per 1000 | 16.2 per 1000 (10.8 to 27) | 10.8 fewer per 1000 (16.2 to 0 fewer) |  |  |
|  |  | High-risk population^‡^ | | |  |  |
|  |  | 47 per 1000 | 28.2 per 1000 (18,8 to 47) | 18.8 fewer per 1000 (28.2 to 0 fewer) |  |  |
| **Scholes et al. – Offer to screen - selected individuals in screening program – All-cause PID** | | | | | | |
| All-cause PID (Eligible selected participants)  Follow-up: 12 mos  2,607 18-34 yrs (1 RCT) | 0.43 (0.21 to 0.89) | Control event rate (21 per 1000) | | | ⊕⊕⊖⊖-⊕⊕⊕⊖  LOW-TO-MODERATE  (General risk population) ^i-l^ due to some risk of bias and serious imprecision  ⊕⊕⊖⊖  LOW (High-risk populations) ^c, i-l^ due to some risk of bias and indirectness, and serious imprecision | Offering a single CT screen to select groups of females 18-34 years old in general-risk populations may reduce all-cause PID (15.4 fewer per 1000 [3 to 21.3 fewer]; NNS 65 [47 to 333]).  The reduction in PID may be larger for those in populations at high-risk for CT, but the magnitude of the difference is uncertain. |
|  |  | 21 per 1000 | 9.2 per 1000 (4.7 to 18.7) | 11.8 fewer per 1000 (2.3 to 16.3 fewer) |  |  |
|  |  | General-risk population^†^ | | |  |  |
|  |  | 27 per 1000 | 11.6 per 1000 (5.7 to 24) | 15.4 fewer per 1000 (3 to 21.3 fewer) |  |  |
|  |  | High-risk population^‡^ | | |  |  |
|  |  | 47 per 1000 | 20.2 per 1000 (9.9 to 41.8) | 26.8 fewer per 1000 (5.2 to 37.1 fewer) |  |  |

Abbreviations: CI=confidence interval; CT= chlamydia trachomatis; MID=minimally important difference; PID= pelvic inflammatory disease; RCT= randomized controlled trial; ROB= risk of bias; RR=relative risk

*The absolute effect (and its 95% CI) without screening (i.e. baseline rate) is based on the estimated risk in the comparison group; the effect with a single screen is based on applying the relative effect of the intervention (and its 95% CI) to the effect without screening.

†The effects without screening for the general-risk population assumed that approximately 6% of the female population would have CT (prevalence), and that about 13% of these females would develop PID (0.78%), and that approximately 25-30% of all-cause PID is attributed to CT (all cause PID = 3.5 times PID from CT); 0.78% x 3.5 = 2.7%.

‡The absolute effects for the high-risk population assumed a higher (12%) CT prevalence and slightly higher (33%) attribution to CT (1.56% x 3 =4.7%).

**Explanations:**

^a^ Indirectness: Serious concerns about lack of complete PID ascertainment (Andersen used hospital diagnoses and doxycycline prescriptions; Hocking only used clinic charts; PID assessed in some ineligible [i.e. not sexually active] individuals), use of population registers in Andersen whch may not reflect practice in primary care, and use of usual care (rather than no screening) comparisons which may have underestimated the effects.

^b^ Imprecision: Sample size adequate but 95% CIs cross both benefit (2.5 fewer) and harm (2.5 more) thresholds.

^c^ Indirectness of high-risk estimate: Added concerns about using RR and natural history data from studies in general-risk population to estimate the effects of screening in high-risk populations where other factors (e.g., immunocompromised, re-infection rates, higher STI co-infection) may be important.

^d^ Indirectness: Very serious concerns about lack of PID ascertainment (hospital data in different source population from trial and only capturing 21% of PID cases if assuming each case of PID is in a unique individual), and use of usual care (rather than no screening) comparison which may have underestimated the effects.

**^e^** Imprecision: No concerns; sample size adequate and entire 95% CI does not meet threshold for important effect.

^f^ Inconsistency: Serious concerns because inconsistent with other analysis (clinic PID) showing effects below threshold.

^g^ Indirectness: Serious concerns about lack of PID ascertainment. Use of usual care control group (rather than no screening) that underwent some screening, but we did not rate down because believe that this would have dampened the effects such the true effect with a no screening control would still surpass the MID threshold (i.e., certainty in an effect greater than the MID is not reduced).

^h^ Imprecision: Sample size adequate but entire 95% CI does not surpass the threshold.

^i^ Risk of bias: Some concerns about unclear ROB for selection, performance, and detection biases.

^j^ Inconsistency: Only study in analysis but findings are similar to those for acceptors of screening (see Evidence Set 2) and this study had fairly high rates of acceptance to the screening.

^k^ Indirectness: Use of usual care control group (rather than no screening) that would have undergone some degree of testing in asymptomatic cases, but did not rate down because believe that this would have dampened the effects such the true effect with a no screening control would still surpass the MID threshold; outcome ascertainment and applicability of setting is good in this study.

^l^ Imprecision: 95% CI indicates benefit but sample size is small for rare outcome.

**1C Forest Plot**

**
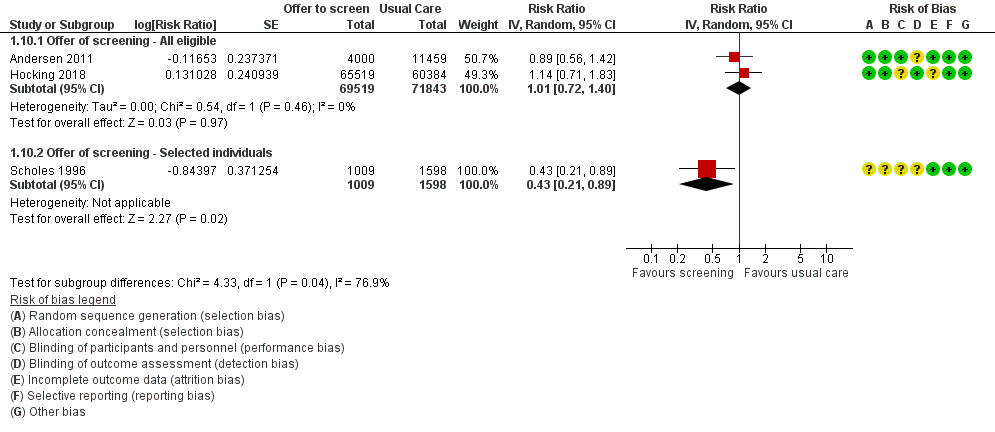
**

**Evidence Set 2: Screening vs. no screening; Pelvic Inflammatory Disease (PID; All-cause) (Acceptors of screening)(15-29 year-olds)**

Included studies: Ostergaard 2000 (RCT), Oakeshott 2010 (RCT), Clark 2001 (CCT); Sufrin 2012 and Low 2006 (cohort studies)

Threshold for important effect: 2.5 per 1000 fewer [benefit] or more [harm]

**2A GRADE Evidence Profile - Trials**

| **Population** | **Certainty assessment** | | | | | | | **№ of patients** | | **Effect** | | **Certainty** |
| --- | --- | --- | --- | --- | --- | --- | --- | --- | --- | --- | --- | --- |
|  | **№ of studies** | **Study design** | **Risk of bias** | **Inconsistency** | **Indirectness** | **Imprecision** | **Other considerations** | **Screening** | **Usual care** | **Relative (95% CI)** | **Absolute (95% CI)** |  |
| **Acceptors of screening – All-cause PID** | | | | | | | | | | | | |
| **Median control event rate (1.8%)** | 3 | 2 RCTs  1 CCT | No serious concerns^a^ | No serious concerns | -1.0 or -0.5^b^ | -1.0^c^ | No concerns | 8,283 | 22,369 | 0.79 (0.60 to 1.04) | 3.7 fewer per 1000 (7.1 fewer to 0.7 more) | ⊕⊕⊖⊖  LOW  (LOW-TO-MODERATE for outreach settings) |
| **General-risk population (2.7%)*** | 3 | 2 RCTs  1 CCT | No serious concerns^a^ | No serious concerns | -1.0 or -0.5^b^ | -1.0^c^ | No concerns | 8,283 | 22,369 | 0.79 (0.60 to 1.04) | 5.7 fewer per 1000 (10.8 fewer to 1.1 more) | ⊕⊕⊖⊖  LOW  (LOW-TO-MODERATE for outreach settings) |
| **High-risk population (4.7%)^†^** | 3 | 2 RCTs  1 CCT | No serious concerns^a^ | No serious concerns | -1.5 or -1.0^b^ | -1.0^c^ | No concerns | 8,283 | 22,369 | 0.79 (0.60 to 1.04) | 9.9 fewer per 1000 (18.8 fewer to 1.9 more) | ⊕⊖⊖⊖-⊕⊕⊖⊖  VERY LOW-TO-LOW  (LOW for outreach settings) |

Abbreviations: CI=confidence interval; CCT=controlled clinical trial; CT= chlamydia trachomatis; MID=minimally important difference; PID= pelvic inflammatory disease; RCT=randomized controlled trial; ROB=risk of bias

*The absolute effects in all-cause PID for the general-risk population assumed that approximately 6% of the female population would have CT (prevalence), and that about 13% of these females would develop PID (0.78%), and that approximately 25-30% of all-cause PID is attributed to CT (all cause PID = 3.5 times PID from CT); 0.78% x 3.5 = 2.7%.

†The absolute effects in all-cause PID for the high-risk population assumed a higher (12%) CT prevalence and slightly higher (33%) attribution to CT (1.56% x 3 =4.7%).

**Explanations:**

^a^ Risk of bias: Some concerns in Clark (selection bias) and Ostergaard (incomplete outcome data; use of complete case analysis with 47% follow-up) but point estimate quite consistent with those from low ROB trial Oakeshott, and Ostergaard contributes very little weight (5%) in analysis so did not rate down.

^b^ Indirectness: Serious concerns from inapplicability of settings (outreach) to primary care in all three trials and possibly low ascertainment of PID (Clark used hospital diagnoses only; Ostergaard used self-report). The use of usual care rather than no screening comparators may have dampened the effects, but the true effect with a no screening control would still surpass the MID threshold so did not rate down. We also assessed the certainty specific to an outreach setting which led to less uncertainty (-0.5 vs -1.0). For the high-risk population estimate of small-to-moderate effects, we have additional uncertainty because of reliance on the RR and baseline estimates of PID that were generated from data in general-risk populations.

^c^ Adequate sample size but large portion of 95% CI does not pass MID threshold.

**2B GRADE Summary of Findings - Trials**

| **Outcome**  **No. participants (studies)** | **Relative effect (95% CI)** | **Anticipated absolute effects (95% CI)*** | | | **Certainty of the evidence (GRADE)** | **What happens?** |
| --- | --- | --- | --- | --- | --- | --- |
|  |  | **Without screening** | **With a single CT screen** | **Difference** |  |  |
| **Acceptors of screening – All-cause PID** | | | | | | |
| All-cause PID (Trials)  Follow-up: 12-18 mos  30,652 (2 RCTs, 1 CCT) 15-29 years | 0.79 (0.60 to 1.04) | Median control event rate (18 per 1000) | | | ⊕⊕⊖⊖  LOW (General-risk populations) due to indirectness and imprecision^a-c^  ⊕⊖⊖⊖-⊕⊕⊖⊖  VERY LOW-TO-LOW (Hiigh-risk populations) due to (more) indirectness, and imprecision^a-c^ | Females 15-29 years of age in general-risk populations who undergo one CT screen may have a reduced risk for PID (5.7 fewer per 1000 [10.8 fewer to 1.1 more]; NNS 75 [CI not esteemable]).  The benefits may be greater for those in populations at high-risk for CT, but the magnitude of the difference is uncertain. |
|  |  | 18 per 1000 | 14.3 per 1000 (10.9 to 18.7) | 3.7 fewer per 1000 (7.1 fewer to 0.7 more) |  |  |
|  |  | General-risk population (27 per 1000)† | | |  |  |
|  |  | 27 per 1000 | 21.3 per 1000 (16.2 to 28.1) | 5.7 fewer per 1000 (10.8 fewer to 1.1 more) |  |  |
|  |  | High-risk population (47 per 1000)‡ | | |  |  |
|  |  | 47 per 1000 | 37.1 per 1000 (28.2 to 48.9) | 9.9 fewer per 1000 (18.8 fewer to 1.9 more) |  |  |

Abbreviations: CI=confidence interval; CCT=controlled clinical trial; CT= chlamydia trachomatis; NNS:number needed to screen; PID= pelvic inflammatory disease; RCT=randomized controlled trial; ROB=risk of bias

*The absolute effect (and its 95% CI) without screening (i.e. baseline rate) is based on the estimated risk in the comparison group; the effect with a single screen is based on applying the relative effect of the intervention (and its 95% CI) to the effect without screening.

†The effects without screening for the general-risk population assumed that approximately 6% of the female population would have CT (prevalence), and that about 13% of these females would develop PID (0.78%), and that approximately 25-30% of all-cause PID is attributed to CT (all cause PID = 3.5 times PID from CT); 0.78% x 3.5 = 2.7%.

‡The absolute effects for the high-risk population assumed a higher (12%) CT prevalence and slightly higher (33%) attribution to CT (1.56% x 3 =4.7%).

**Explanations:**

^a^ Some concerns in Clark (selection bias) and Ostergaard (incomplete outcome data; use of complete case analysis with 47% follow-up) but point estimate quite consistent with those from low ROB trial Oakeshott, and Ostergaard contributes very little weight (5%) in analysis so did not rate down.

^b^ Serious concerns about applicability of settings (outreach) to primary care in all three trials and possibly low ascertainment of PID (Clark used hospital diagnoses only; Ostergaard used self-report). The use of usual care rather than no screening comparators may have dampened the effects, but the true effect with a no screening control would still surpass the MID threshold (i.e., certainty in an effect greater than the MID is not reduced) so did not rate down. We also assessed the certainty specific to an outreach setting which led to less uncertainty (-1.0 versus -1.5). For the high-risk population estimate of a small-to-moderate effect, we have additional uncertainty because of reliance on the RR and baseline estimates of PID that were generated from data in general-risk populations.

^c^ Adequate sample size but large portion of 95% CI does not pass MID threshold.

**2C Forest Plot - Trials**


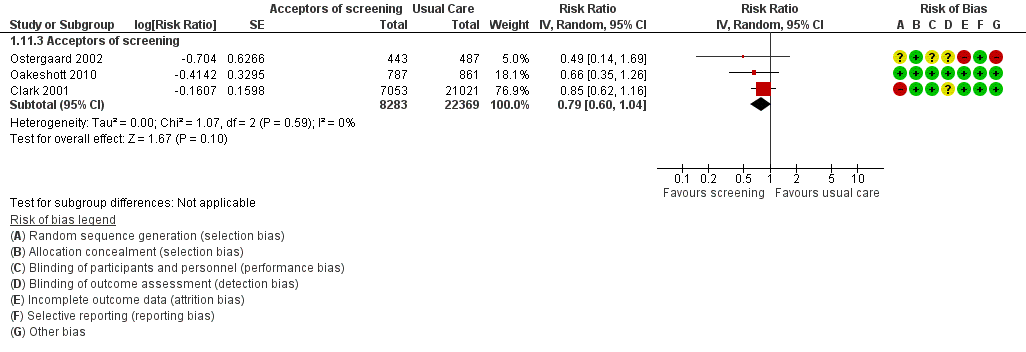


**2D GRADE Evidence Profile – Observational studies**

| **Population** | **Certainty assessment** | | | | | | | **Findings** | **Certainty** |
| --- | --- | --- | --- | --- | --- | --- | --- | --- | --- |
|  | **№ of studies** | **Study design** | **Risk of bias** | **Inconsistency** | **Indirectness** | **Imprecision** | **Other considerations** |  |  |
| **OBSERVATIONAL STUDIES** | | | | | | | | | |
| **General risk** | 2 | Cohort | -0.5^a^ | -2.0^b^ | -0.5^c^ | No concerns^d^ | No concerns | One study (Sufrin; n=57,728) found little to no difference in PID rates (ORa 1.05, 95% CI 0.78 to 1.43). In the other (Low; n=709,000 women years), PID occurred at 5.6% (4.7% to 6.7%) in women who ever tested positive for CT, 4.0% (3.7% to 4.4%) in those with all negative tests, and 2.9% (2.7% to 3.2%) in those who were never screened opportunistically between the ages of 15 and 24 years. | ⊕⊖⊖⊖  VERY LOW |

Abbreviations: CT=chlamydia trachomatis; ORa= odds ratio adjusted; PID=pelvic inflammatory disease

**Explanations:**

^a^ Concerns about comparability between groups in both studies (and no adjustment for sexual behaviors) and possible selective analysis reporting in Low; may contribute to differences in findings between studies.

^b^ Serious concerns about heterogeneity; one study found that screening did not make an impact whereas the other found screening may have a large harm (e.g. between 11 and 27 more in 1000, with threshold of important harm at 2.5 in 1000 more).

^c^  Concerns about low outcome ascertainment; Low study used hospital data only.

^d^ Sample sizes adequate but wide confidence interval assumed if combining studies; attributed to inconsistency.

**2E GRADE Summary of Findings Table – Observational studies**

| **Outcome**  **No. participants (studies)** | **Findings** | **Certainty of the evidence (GRADE)** | **What happens?** |
| --- | --- | --- | --- |
| **OBSERVATIONAL STUDIES** | | | |
| All-cause PID (Observational)  100% adherence designs  Follow-up: 3 mos and 10 yrs  101,443 (2 cohorts) | One study (Sufrin; n=57,728) found little to no difference in PID rates (ORa 1.05, 95% CI 0.78 to 1.43). In the other (Low; n=43, 715 with 709,000 women years), PID occurred at 5.6% (4.7% to 6.7%) in women who ever tested positive for CT, 4.0% (3.7% to 4.4%) in those with all negative tests, and 2.9% (2.7% to 3.2%) in those who were never screened opportunistically between the ages of 15-24. | ⊕⊖⊖⊖  VERY LOW due to ROB, inconsistency, and indirectness ^a-d^ | The evidence from observational studies is very uncertain about the effects from screening for CT on PID. |

Abbreviatiomns: CT=chlamydia trachomatis; ORa=odds ratio adjusted; PID=pelvic inflammatory disease

**Explanations:**

^a^ Concerns about comparability between groups in both studies (and no adjustment for sexual behaviors) and possible selective analysis reporting in Low; may contribute to differences in findings between studies.

^b^ Serious concerns about heterogeneity; one study found that screening did not make an impact whereas the other found screening may have a large harm (e.g. between 11 and 27 more in 1000, with threshold of important harm at 2.5 in 1000 more).

^c^  Concerns about low outcome ascertainment; Low study used hospital data only.

^d^ Sample sizes adequate but wide confidence interval assumed if combining studies; attributed to inconsistency.

**Evidence Set 3: Screening vs. no screening; Ectopic Pregnancy (All-cause)(Offer to screen and acceptors of screening)(17-29 yrs)**

Included studies: Offer to screen: Andersen 2011 (RCT); acceptors of screening: Clark 2001 (CCT), Low 2006 (cohort)

Threshold for important effect: 1 per 1000 fewer [benefit] or more [harm]

**3A GRADE Evidence Profile Table**

| **Certainty assessment** | | | | | | | **№ of patients** | | **Effect** | | **Certainty** |
| --- | --- | --- | --- | --- | --- | --- | --- | --- | --- | --- | --- |
| **№ of studies** | **Study design** | **Risk of bias** | **Inconsistency** | **Indirectness** | **Imprecision** | **Other considerations** | **Screening** | **Usual care** | **Relative (95% CI)** | **Absolute (95% CI)** |  |
| **Offer to screen - All eligible participants** | | | | | | | | | | | |
| 1 | RCT | No concerns | -0.5^a^ | -1.0^b^ | -1.5^c^ | No concerns | 4000 | 11459 | 1.03 (0.67 to 1.60) | 0.20 more per 1000 (2.2 fewer to 3.9 more) | ⊕⊖⊖⊖  VERY LOW |
| **Acceptors of screening** | | | | | | | | | | | |
| 1 | CCT | -0.5^d^ | No concerns^e^ | -2.0^f^ | -1.0^g^ | No concerns | 7053 | 21021 | RR 1.19 (0.77 to 1.85) | 0.63 more per 1000 (0.76 fewer to 2.8 more) | ⊕⊖⊖⊖  VERY LOW |
| 1 | Cohort | -0.5^h^ | No concerns^e^ | -0.5^i^ | -0.5^j^ | No concerns | Ectopic pregnancy occurred in 2.7% (2.1% to 3.5%) of women who ever tested positive for CT, 2.0% (1.8% to 2.3%) in those with all negative tests, and 1.9% (1.7% to 2.1%) in those who were never screened. | | | | ⊕⊖⊖⊖  VERY LOW |

CI: confidence interval; CT: chlamydia trachomatis; CCT: controlled clinical trial; RCT: randomized controlled trial; RR=relative risk

**Explanations:**

^a^ Concern about lack of evidence of consistency.

^b^ Concern about indirectness from poor outcome ascertainment (only hospital diagnoses) and use of usual care comparison group where some screening occurred.

^c^ Serious concern about imprecision because 95% CI of absolute effects passes MID thresholds for benefits and harms (1 fewer and 1 more per 1000, respectively)..

^d^ Concern about ROB from high selection bias.

^e^ Some concerns about lack of evidence of consistency but results are similar between CTT and observational study.

^f^ Very serious concern about indirectness from poor outcome ascertainment (only hospital diagnoses), use of usual care comparison group, setting being inapplicable to primary care, and having limited follow-up duration (3 yrs).

^g^ Concern about imprecision because 95% CI of absolute passes threshold for harm.

^h^ Concerns about comparability between groups and possible selective analysis reporting because of no comparison based on screening status.

^i^ Concerns because only used hospital diagnoses.

^j^ Sample size adequate. No adjusted effects for screened versus not screened populations, but 95% CIs around absolute effects (knowing majority of females are negative for CT) indicates the effects could likely be a small increase (>1 in 1000 threshold) or little to no difference.

**3B GRADE Summary of Findings**

| **Outcome**  **No. participants (studies)** | **Relative effect (95% CI)** | **Anticipated absolute effects (95% CI)*** | | | **Certainty of the evidence (GRADE)** | **What happens?** |
| --- | --- | --- | --- | --- | --- | --- |
|  |  | **Without screening** | **With a single CT screen** | **Difference** |  |  |
| **Offer to screen - All eligible participants in a screening programme (general risk)** | | | | | | |
| Ectopic pregnancy (general risk)  Follow-up: 9 yrs  15,459 (1 RCT) | RR 1.03 (0.67 to 1.60) | 6.5 per 1000 | 6.35 per 1000 (4.4 to 10.5) | 0.20 more per 1000 (2.2 fewer to 3.9 more) | ⊕⊖⊖⊖  VERY LOW for concerns about lack of consistency and indirectness and serious concerns about imprecision^a-c^ | Offering a single CT screen to general-risk females may make little to no difference in rates of ectopic pregnancy (0.20 more in 1000 [2.2 fewer to 3.9 more]), but the evidence is very uncertain. |
| **Acceptors of screening programme** | | | | | | |
| Ectopic pregnancy (high risk)  Follow-up: 1.5 yrs  28,074 (1 CCT) | RR 1.19 (0.77 to 1.85) | 3.3 per 1000 | 4.0 per 1000 (2.6 to 6.2) | 0.63 more per 1000 (0.76 fewer to 2.8 more) | ⊕⊖⊖⊖  VERY LOW for concerns about risk of bias and imprecision and very serious concerns about indirectness^d-g^ | For females who attend a single screen for CT, there may be little to no difference in rates of ectopic pregnancy (0.63 more per 1000 [0.76 fewer to 2.8 more]), but the evidence is very uncertain. |
| Ectopic pregnancy  Follow-up: 10 yrs  43,714 (1 cohort) | Ectopic pregnancy occurred in 2.7% (2.1% to 3.5%) of women who ever tested positive for CT, 2.0% (1.8% to 2.3%) in those with all negative tests, and 1.9% (1.7% to 2.1%) in those who were never screened (Low). | | | | ⊕⊖⊖⊖  VERY LOW^h-j^ due to risk of bias, indirectness, and imprecision | The evidence from observational studies is very uncertain about the effects from screening for CT on ectopic pregnancy. |

CI: confidence interval; CT: chlamydia trachomatis; CCT: controlled clinical trial; RCT: randomized controlled trial; RR=relarive risk

*The effect with screening (and its 95% confidence interval) is based on the effects without screening and the relative effect of the intervention (and its 95% CI).

**Explanations:**

^a^ Concern about lack of evidence of consistency.

^b^ Concern about indirectness from poor outcome ascertainment (only hospital diagnoses) and use of usual care comparison group.

^c^ Serious concern about imprecision because 95% CI of absolute effects passes thresholds for benefits and harms.

^d^ Concern about ROB from selection bias.

^e^ Some concerns about lack of evidence of consistency but results are similar between CTT and observational study.

^f^ Very serious concern about indirectness from poor outcome ascertainment (only hospital diagnoses), use of usual care comparison group, setting being inapplicable to primary care, and having limited follow-up duration (3 yrs).

^g^ Concern about imprecision because 95% CI of absolute effects passes threshold for harm.

^h^ Concerns about comparability between groups and possible selective analysis reporting because of no comparison based on screening status.

^i^ Concerns because only used hospital diagnoses.

^j^ Sample size adequate. No adjusted effects for screened versus not screened populations, but 95% CIs around absolute effects (knowing majority of females are negative for CT) indicates the effects could likely be a small increase (>1 in 1000 threshold) or little to no difference.

**3C Forest Plot for Trials**


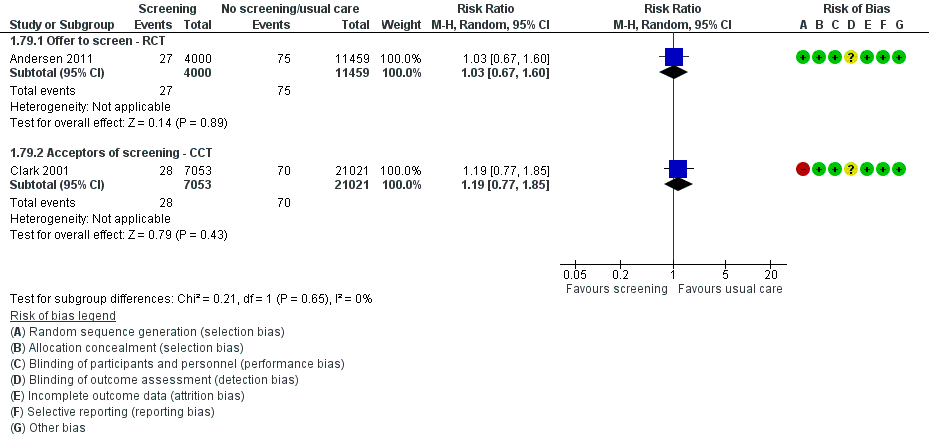


**Evidence Set 4: Screening vs. no screening; Infertility (All-cause)(Offer to screen and acceptors of screening)(17-29 yrs)**

Included studies: Offer to screen: Andersen 2011 (RCT); acceptors of screening: Clark 2001 (CCT), Low 2006 (cohort)

Threshold for important effect: 1 per 1000 fewer [benefit] or more [harm]

**4A GRADE Evidence Profile**

| **Certainty assessment** | | | | | | | **№ of patients** | | **Effect** | | **Certainty** |
| --- | --- | --- | --- | --- | --- | --- | --- | --- | --- | --- | --- |
| **№ of studies** | **Study design** | **Risk of bias** | **Inconsistency** | **Indirectness** | **Imprecision** | **Other considerations** | **Screening** | **Usual care** | **Relative (95% CI)** | **Absolute (95% CI)** |  |
| **Offer to sreen – all eligible participants** | | | | | | | | | | | |
| 1 | RCT | No concerns | -0.5^a^ | -1.0^b^ | -1.5^c^ | No concerns | 4000 | 11459 | 1.15 (0.94 to 1.40) | 4.2 more per 1000 (1.7 fewer to 11.2 more) | ⊕⊖⊖⊖  VERY LOW |
| **Acceptors of screening** | | | | | | | | | | | |
| 1 | CCT | -0.5^d^ | -0.5^e^ | -2.0^f^ | No concerns | No concerns | 7053 | 21021 | 0.66 (0.14 to 3.06) | 0.15 fewer per 1000 (0.37 fewer to 0.88 more) | ⊕⊖⊖⊖  VERY LOW |
| 1 | Cohort | -0.5^g^ | -0.5^e^ | -0.5^h^ | No concerns^i^ | No concerns | Infertility occurred in 6.7% (5.7% to 7.9%) of women who ever tested positive for CT, 4.7% (4.4% to 5.1%) in those with all negative tests, and 3.1% (2.8% to 3.3%) in those who were never screened. | | | | ⊕⊖⊖⊖  VERY LOW |

Abbreviations: CI=confidence interval; CT=chlamydia trachomatis; CCT=controlled clinical trial; RCT=randomized controlled trial; ROB=risk of bias

**Explanations:**

^a^ Concerns about lack of evidence of consistency.

^b^ Serious concerns about outcome ascertainment (hospital diagnoses) and use of usual care comparison group.

^c^ Sample size may be adequate but 95% CI wide with limits passing thresholds for benefit and harm (1 fewer and 1 more per 1000, respectively)

^d^ Concerns about ROB from selection bias.

^e^ Some concerns that studies in these analyses had different findings.

^f^ Very serious concern about indirectness from poor outcome ascertainment (only hospital diagnoses), use of usual care comparison group, setting being inapplicable to primary care, and having limited follow-up duration (1.5 yrs).

^g^ Concerns about comparability between groups and possible selective analysis reporting.

^h^ Concerns because only used hospital diagnoses.

^i^ Sample size adequate. No adjusted effects for screened versus not screened populations, but 95% CIs around absolute effects (knowing majority of females do not experience CT) indicates the effects would likely show an increase (>1 in 1000 threshold) which is precise.

**4B GRADE Summary of Findings**

| **Outcome**  **No. participants (studies)** | **Relative effect (95% CI)** | **Anticipated absolute effects (95% CI)*** | | | **Certainty of the evidence (GRADE)** | **What happens?** |
| --- | --- | --- | --- | --- | --- | --- |
|  |  | **Without screening** | **With a single CT screen** | **Difference** |  |  |
| **Offer to screen – all eligible participants** | | | | | | |
| Infertility (general-risk females)  Follow-up: 9 years  15,459 (1 RCT) | RR 1.15 (0.94 to 1.40) | 28.1 per 1000 | 32.3 per 1000 (26.4 to 39.3) | 4.2 more per 1000 (1.7 fewer to 11.2 more) | ⊕⊖⊖⊖  VERY LOW due to lack of consistency, indirectness and imprecision^a-c^ | The evidence is very uncertain about the effects on infertility from offering a single CT screen to general-risk females. |
| **Acceptors of screening** | | | | | | |
| Infertility (high-risk females)  Follow-up: 1.5 years  28,074 (1 CCT) | RR 0.66 (0.14 to 3.06) | 0.43 per 1000 | 0.28 per 1000 (0.06 to 1.31) | 0.15 fewer per 1000 (0.37 fewer to 0.88 more) | ⊕⊖⊖⊖  VERY LOW due to ROB, inconsistency and indirectness^d-f^ | The evidence is very uncertain about the effects on infertility for general-risk females who undertake a single CT screen. |
| Infertility (females)- Observational  Follow-up: 10 years  43,715 (cohort) | Infertility occurred in 6.7% (5.7% to 7.9%) of women who ever tested positive for CT, 4.7% (4.4% to 5.1%) in those with all negative tests, and 3.1% (2.8% to 3.3%) in those who were never screened. | | | | ⊕⊖⊖⊖  VERY LOW^e-h^ due to risk of bias, inconsistency and indirectness | The evidence from observational studies is very uncertain about the effects from screening for CT on infertility. |

CI: confidence interval; CT: chlamydia trachomatis; CCT: controlled clinical trial; RCT: randomized controlled trial; RR=relative risk

*The effect with a single CT screen (and its 95% confidence interval) is based on the effect without screening and the relative effect of the intervention (and its 95% CI).

**Explanations:**

^a^ Concerns about lack of evidence of consistency.

^b^ Serious concerns about outcome ascertainment (hospital diagnoses) and use of usual care comparison group.

^c^ Sample size may be adequate but 95% CI wide with limits passing thresholds for benefit and harm (1 fewer and 1 more per 1000, respectively)

^d^ Concerns about ROB from selection bias

^e^.Some concerns that studies in these analyses had different findings

^f^ Very serious concern about indirectness from poor outcome ascertainment (only hospital diagnoses), use of usual care comparison group, setting being inapplicable to primary care, and having limited follow-up duration (1.5 yrs).

^g^ Concern about inadequate sample size with very low rate of events.

^h^ Concerns about comparability between groups and possible selective analysis reporting.

^i^ Concerns because only used hospital diagnoses.

^j^ Sample size adequate. No adjusted effects for screened versus not screened populations, but 95% CIs around absolute effects (knowing majority of females do not experience CT) indicates the effects would likely show an increase (>1 in 1000 threshold) which is precise.

**4C Forest Plot for Trials**

**
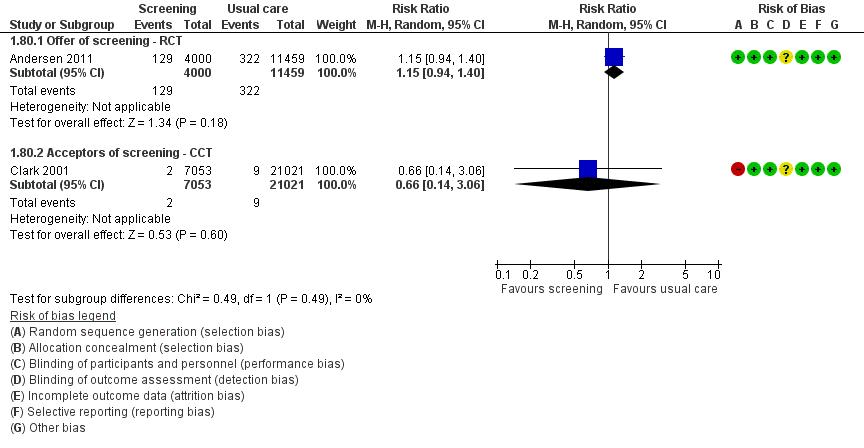
**

**Evidence Set 5: Screening offer vs. no screening; Transmission of CT: Population prevalence**

Included studies: RCTs: van den Broek, Hocking, Hodgins, Garcia, CCT: Cohen

Thresholds for important effect (MID): 5 or 10 per 1000 fewer [benefit] or more [harm]

**5A GRADE Evidence Profile Table**

| **Certainty assessment** | | | | | | | **№ of patients** | | **Effect** | | **Certainty** |
| --- | --- | --- | --- | --- | --- | --- | --- | --- | --- | --- | --- |
| **№ of studies** | **Study design** | **Risk of bias** | **Inconsistency** | **Indirectness** | **Imprecision** | **Other considerations** | **Screening** | **Usual care** | **Relative (95% CI)** | **Absolute (95% CI)** |  |
| **Both sexes in general population; low-intensity CT screening for females and males** | | | | | | | | | | | |
| 3 | RCTs | -0.5 | No concerns | -0.5 | -1.0 for 0.5% MID  -0.5 for 1% MID | No concerns | 32,405 | 9,304 | OR: 0.88 (95% CI 0.64 to 1.20); calculated RR: 0.91 (0.65-1.21) using control event rate 3.3% | 3 fewer per 1000 (11.5 fewer to 6.9 more) | ⊕⊕⊖⊖  LOW  (0.5% MID)  ⊕⊕⊕⊖-⊕⊕⊖⊖  LOW-TO-MODERATE  (1% MID) |
| **Females in general population; low-intensity CT screening for females and males** | | | | | | | | | | | |
| 2 | RCTs | -0.5 | No concerns | -0.5 | -1.5 for 0.5% MID but -1.0 for 1.0% MID | No concerns | 21,862 | 5,438 | OR: 0.89 (95% CI 0.63 to 1.27); calculated RR: 0.89 (95% CI 0.64 to 1.26) using 3.75% control event rate | 4.1 fewer (13.5 fewer to 9.75 more) | ⊕⊖⊖⊖-⊕⊕⊖⊖  VERY LOW to LOW (0.5% MID)  ⊕⊕⊖⊖  LOW (1% MID) |
| **Males in general population; low-intensity CT screening for females and males** | | | | | | | | | | | |
| 2 | RCTs | -0.5 | -0.5 | -0.5 | -1.5  OR,  1% MID  -1.0 | No concerns | 9,237 | 2,512 | OR 0.83 (95% CI 0.57 to 1.20); calculated RR 0.84 (95% CI 0.58 to 1.19) using control evet rate 4.5% | 7.2 fewer (18.9 fewer to 8.6 more) | ⊕⊕⊖⊖  VERY LOW (0.5% MID)  ⊕⊕⊖⊖ to ⊕⊖⊖⊖  LOW-TO-VERY LOW (1.0% MID) |
| **Both sexes in high-risk population; moderate-intensity CT and NG screening for females and males** | | | | | | | | | | | |
| 1 | CCT | -1.0 | -0.5 | -1.0 | -1.5  Both MIDs | No concerns | 1,150 | 2,653 | RR 0.72 (955 CI 0.30 to 1.73) after adjustment for clustering | 26 fewer cases in 1000 (65 fewer to 68 more) | ⊕⊖⊖⊖  VERY LOW |
| **Females in high-risk population; high-intensity screening for CT & NG** | | | | | | | | | | | |
| 2 | 1 RCT  1 CCT | -0.5 | No concerns | -1.0  OR (Outreach settings)  -0.5 | No concerns | No concerns | 2,654 | 3,473 | RR: 0.74 (95% CI 0.56 to 0.97) | 34.3 fewer per 1000 (4 to 58 fewer) | ⊕⊕⊕⊖-⊕⊕⊖⊖  LOW-TO-MODERATE (0.5% and 10% MIDs) |
| **Males in high-risk population; moderate-intensity screening for CT & NG** | | | | | | | | | | | |
| 1 | CCT | -1.0 | -0.5 | -0.5 | -1.5 | No concerns | 588 | 1242 | RR 0.51 (95% CI 0.14 to 1.82) adjusted for clustering | 31 fewer per 1000 (55 fewer to 52 more) | ⊕⊖⊖⊖  VERY LOW (0.5% & 1.0 MIDs) |

Abbreviations: CI=confidence interval; CT=chlamydia trachomatis; CCT=controlled clinical trial; MID=minimally important difference; NG=neisseria gonorrhea; OR=odds ratio; RCT=randomized controlled trial; ROB=risk of bias; RR=relative risk

**Explanations:**

Both sexes in general population; low-intensity CT screening for females and males: for all three trials there was some concern about **ROB** from performance bias and attrition bias, Hodgins and van den Broek were also unclear for detection bias**;** concerns about **indirectness** because of the use of usual care (rather than no screening) for the control groups which may have underestimated the effects from screening**; imprecision** around the finding of little to no difference is serious, with the range of effects indicating possible benefit. There is more certainty that the true effect will not meet an importance threshold of 10 fewer in 1000.

Females in general population; low-intensity CT screening for females and males: **ROB** was rated down by 0.5 levels because of concerns about risk for performance and detection biases, and incomplete outcome data; some concern about **indirectness** mostly due to the use of a usual care control group; very serious concerns (-1.5) about **imprecision** when *applying the 0.5% importance threshold* because the range of possible effects includes moderate benefit and small harm. Less uncertainty (-1.0) for the higher threshold of 1% reduction.

Males in general-risk population; low-intensity CT screening: **ROB** was rated down by 0.5 levels because of concerns about risk for performance and detection biases, and incomplete outcome data; some concern about **inconsistency** in magnitude of effects (14 fewer in Hocking and 2 fewer in van den Brooek); some concern about **indirectness** from the use of usual care comparators; and very serious (0.5% MID) **imprecision** about the effect of small reduction when the possible range of effects (95% CI) crosses both no effect and harm. Less uncertainty about the lack of a moderate effect (<1% MID).

Both sexes in high-risk population; moderate-intensity CT and NG screening for females and males: serious concerns about **ROB** from unclear selection (not randomized but groups balanced) and performance biases, and high risk for attrition (>30%) and other biases from failure to adjust for clustering; concerns about **lack of any consistency** shown because only study in analysis; serious concerns about **indirectness** due to use of positivity of those testing rather than prevalence from a representative sample for outcome ascertainment, and from use of usual care control group; serious concerns about **imprecision** for both the 0.5% and 1.0% important thresholds due to inadequate sample size (for cluster RCT) and the range where the effects may be (95% CI) including effects of no benefit and important harm.

Females in high-risk population; high-intensity screening for CT & NG: some concerns that **ROB** due to performance bias (both trials) and selection, attrition and other biases (lack of control for clustering) in Cohen contributed to heterogeneity in magnitude of effects (did not also downgrade for inconsistency); serious concerns about **indirectness** related to setting (not healthcare settings), and co-interventions in the Garcia trial (less concern about indirectness for screening in outreach settings); no serious concerns for imprecision as 95% CI crosses importance thresholds of 5 and 10 per 1000 but large majority of range of effects pass threshold.

Males in high-risk population; moderate-intensity screening for CT & NG: serious concerns for **ROB** from selection, performance, attrition and other biases; some concerns about **inconsistency** (only study) and **indirectness** (usual care comparator); serious concerns about **imprecision** due to small sample for cluster study leading to wide 95% CI including both no effect and important harm.

**5B GRADE Summary of Findings Tables**

| **Outcome**  **No. participants (studies)** | **Relative effect (95% CI)** | **Anticipated absolute effects (95% CI)*** | | | **Certainty of the evidence (GRADE)** | **What happens?** |
| --- | --- | --- | --- | --- | --- | --- |
|  |  | **Without screening** | **With screening** | **Difference** |  |  |
| **Both sexes in general population; low-intensity CT screening for females and males** | | | | | | |
| **Transmission: estimated population prevalence of CT (Both sexes in general-risk population)**  Follow-up: 12-36 mos  41,709 (3 cluster RCTs) | RR: 0.91 (0.65 to1.21) | 33 per 1000 | 30 per 1000 (21.5 to 39.93) | 3 fewer per 1000 (11.5 fewer to 6.9 more) | ⊕⊕⊖⊖  LOW  (0.5% MID)  ⊕⊕⊕⊖-⊕⊕⊖⊖  LOW-TO-MODERATE  (1% MID) | Offering screening to both sexes, 15-29 years old at general-risk, for CT annually may make little to no difference in the prevalence of CT. |
| **Females in general population; low-intensity CT screening for females and males** | | | | | | |
| **Transmission: estimated population prevalence of CT (Females in general-risk population)**  Follow-up: 24-36 mos  27,300 (2 cluster RCTs) | RR: 0.89 (95% CI 0.64 to 1.26) | 37.5 per 1000 | 33.4 (24 to 47.25) | 4.1 fewer per 1000 (13.5 fewer to 9.75 more) | ⊕⊖⊖⊖-⊕⊕⊖⊖  VERY LOW to LOW (0.5% MID)  ⊕⊕⊖⊖  LOW (1% MID) | Offering screening to both sexes, 15-29 years old at general-risk, for CT annually may make little to no difference in the prevalence of CT in females of this population. |
| **Males in general population; low-intensity CT screening for females and males** | | | | | | |
| **Transmission: estimated population prevalence of CT (Males in general-risk population)**  Follow-up: 24-36 mos  11,749 (2 cluster RCTs) | RR 0.84 (95% CI 0.58 to 1.19) | 45 per 1000 | 37.8 (26.1 to 53.55) | 7.2 fewer per 1000 (18.9 fewer to 8.6 more) | ⊕⊕⊖⊖  VERY LOW (0.5% MID)  ⊕⊕⊖⊖ to ⊕⊖⊖⊖  LOW-TO-VERY LOW (1.0% MID) | The evidence in very uncertain about the effects on general-risk males from offering screening to both sexes 15-29 years old for CT annually. |
| **Both sexes in high-risk population; moderate-intensity CT and NG screening for females and males** | | | | | | |
| **Transmission: estimated population prevalence of CT (Both sexes in high-risk population)**  Follow-up: 30 mos  3,803 (1 cluster CCT) | RR 0.72 (955 CI 0.30 to 1.73) | 93 per 1000 | 67 per 1000 (28 to 161) | 26 fewer per 1000 (65 fewer to 68 more) | ⊕⊖⊖⊖  VERY LOW | The evidence is very uncertain for offering screening to both sexes, 15-19 years old at high-risk, for CT biannually to reduce the prevalence of CT. |
| **Females in high-risk population; high-intensity screening for CT & NG** | | | | | | |
| **Transmission: estimated population prevalence of CT (Females in high-risk population)**  Follow-up: 30-48 mos  6,127 (1 RCT, 1 CCT, both cluster) | RR: 0.74 (95% CI 0.56 to 0.97) | 132 per 1000 | 98 per 1000 (74 to 128) | 34.3 fewer per 1000 (4 to 58 fewer) | ⊕⊕⊕⊖-⊕⊕⊖⊖  MODERATE-TO-LOW (0.5% and 10% MIDs) | Frequent offers of screening for CT and NG to high-risk females, 15-29 years, may reduce CT prevalence in these females to a moderate extent.  The use of outreach settings for this form of screening may provide more certainty in the effects. |
| **Males in high-risk population; moderate-intensity screening for CT & NG** | | | | | | |
| **Transmission: estimated population prevalence of CT (Males in high-risk population; moderate-intensity screening for CT & NG)**  Follow-up: 30 mos  1,826 (1 CCT) | RR 0.51 (95% CI 0.14 to 1.82) | 64 per 1000 | 33 per 1000 (9 to 116) | 31 fewer per 1000 (55 fewer to 52 more) | ⊕⊖⊖⊖  VERY LOW (0.5% & 1.0 MIDs) | The evidence is very uncertain for offering screening to high-risk males, 15-19 years old, biannually to reduce the prevalence of CT. |

Abbreviations: CI=confidence interval; CT=chlamydia trachomatis; CCT=controlled clinical trial; MID=minimally important difference; NG=neisseria gonorrhea; OR=odds ratio; RCT=randomized controlled trial; ROB=risk of bias; RR=relative risk

*The effect with screening (and its 95% confidence interval) is based on the effect without screening and the relative effect of the intervention (and its 95% CI).

**Explanations:**

Both sexes in general population; low-intensity CT screening for females and males: for all three trials there was some concern about **ROB** from performance and attrition biases, Hodgins and van den Broek were also unclear for detection bias**;** concerns about **indirectness** because of the use of usual care (rather than no screening) for the control groups which may have underestimated the effects from screening**; imprecision** around the finding of little to no difference is serious, with the range of effects indicating possible benefit. There is more certainty that the true effect will not meet an importance threshold of 10 fewer in 1000.

Females in general population; low-intensity CT screening for females and males: **ROB** was rated down by 0.5 levels because of concerns about risk for performance and detection biases, and incomplete outcome data; some concern about **indirectness** mostly due to the use of a usual care control group; very serious concerns (-1.5) about **imprecision** when *applying the 0.5% importance threshold* because the range of possible effects includes moderate benefit and small harm. Less uncertainty (-1.0) for the higher threshold of 1% reduction.

Males in general-risk population; low-intensity CT screening: **ROB** was rated down by 0.5 levels because of concerns about risk for performance and detection biases, and incomplete outcome data; some concern about **inconsistency** in magnitude of effects (14 fewer in Hocking and 2 fewer in van den Brooek); some concern about **indirectness** from the use of usual care comparators; and very serious (0.5% MID) **imprecision** about the effect of small reduction when the possible range of effects (95% CI) crosses both no effect and harm. Less uncertainty about the lack of a moderate effect (<1% MID).

Both sexes in high-risk population; moderate-intensity CT and NG screening for females and males: serious concerns about **ROB** from unclear selection (not randomized but groups balanced) and performance biases, and high risk for attrition (>30%) and other biases from failure to adjust for clustering; concerns about **lack of any consistency** shown because only study in analysis; serious concerns about **indirectness** due to use of positivity of those testing rather than prevalence from a representative sample for outcome ascertainment, and from use of usual care control group; serious concerns about **imprecision** for both the 0.5% and 1.0% important thresholds due to inadequate sample size (for cluster RCT) and the range where the effects may be (95% CI) including effects of no benefit and important harm.

Females in high-risk population; high-intensity screening for CT & NG: some concerns that **ROB** due to performance bias (both trials) and selection, attrition and other biases (lack of control for clustering) in Cohen contributed to heterogeneity in magnitude of effects (did not also downgrade for inconsistency); serious concerns about **indirectness** related to setting (not healthcare settings), and co-interventions in the Garcia trial (less concern about indirectness for screening in outreach settings); no serious concerns for imprecision as 95% CI crosses importance thresholds of 5 and 10 per 1000 but large majority of range of effects pass threshold.

Males in high-risk population; moderate-intensity screening for CT & NG: serious concerns for **ROB** from selection, performance, attrition and other biases; some concerns about **inconsistency** (only study) and **indirectness** (usual care comparator); serious concerns about **imprecision** due to small sample for cluster study leading to wide 95% CI including both no effect and important harm.

**5C Forest Plots**

**CT prevalence in both sexes**

**
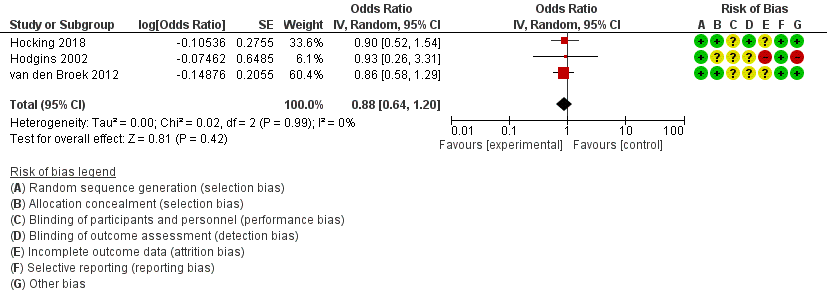
**

**CT prevalence in females**

**
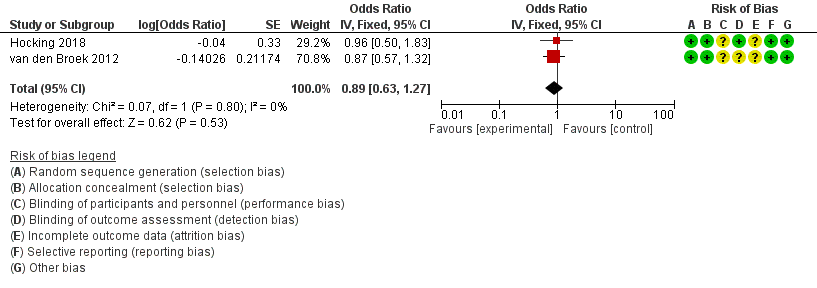
**

**CT prevlance in males**

**
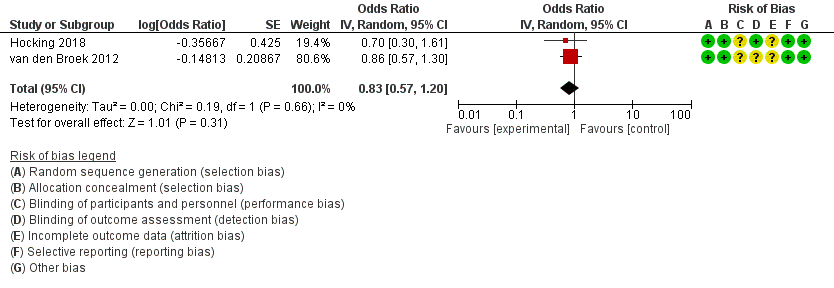
**

**CT prevalence in high-risk females**


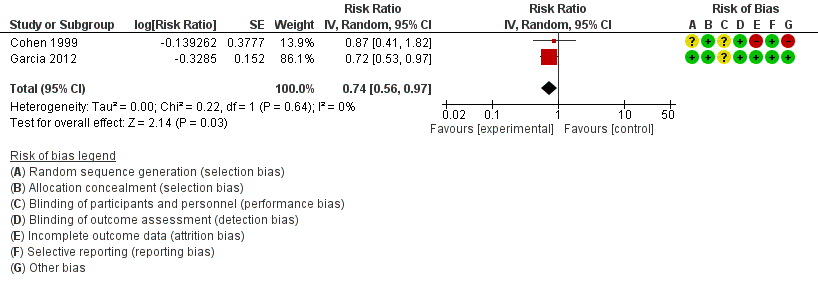


**Evidence Set 6: Screening offer vs. no screening; Transmission of NG: Population prevalence**

Included studies: Garcia (RCT), Cohen (CCT)

**6A GRADE Evidence Profile Table**

| **Certainty assessment (see Explanations for each row below table)** | | | | | | | **Findings** | **Certainty** |
| --- | --- | --- | --- | --- | --- | --- | --- | --- |
| **№ of studies** | **Study design** | **Risk of bias** | **Inconsistency** | **Indirectness** | **Imprecision** | **Other considerations** |  |  |
| **Both sexes in high-risk population; moderate-intensity CT & NG screening for females and males** | | | | | | | | |
| 1 | CCT | -1.0 | -0.5 | -1.0 | -1.5 | No concerns | The CCT found imprecise results (RR 0.81, 95% CI; 0.11 to 6.10 [adjusted for clustering); 3 fewer in 1000 [15 fewer to 87 more]). | ⊕⊖⊖⊖  VERY LOW |
| **Females in high-risk population; high-intensity CT and NG screening** | | | | | | | | |
| 2 | RCT  CCT | -0.5 | No concerns | -1.0  Or,  Outreach  -0.5 | -1.0 | No concerns | Both trials showed reductions in NG prevalence (Cohen: RR 0.77, 95% CI 0.12 to 4.84 [adjusted for clustering], 5 fewer in 1000 [20 fewer to 88 more]; Garcia RCT: RRa: 1.44, 95% CI 0.02 to 122 [uncertainty in the model applied], 7 fewer in 1000 [p=0.026]). An estimate of combined findings is of a beneficial effect. | ⊕⊕⊖⊖ to ⊕⊖⊖⊖  LOW-TO-VERY LOW (0.5% MID)  ⊕⊕⊖⊖  LOW  (1.0% MID) |
| **Males in high-risk population; moderate-intensity CT & NG screening for females and males** | | | | | | | | |
| 1 | CCT | -1.0 | -0.5 | -0.5 | -1.5 | No concerns | The CTT found no benefit for NG prevalence in males (RR 0.96, 95% CI 0.08 to 12.07 [adjusted for clustering], 0.4 fewer in 1000 [10 fewer to 117 more]). | ⊕⊖⊖⊖  VERY LOW |

Abbreviations: CI: confidence interval; CT: chlamydia trachomatis; CCT: controlled clinical trial; NG: neisseria gonorrhea; RCT: randomized controlled trial; ROB: risk of bias; RR: relative risk

**Explanations:**

Both sexes in high-risk population; moderate-intensity CT and NG screening for females and males: serious concerns about **ROB** from unclear selection (not randomized but groups balanced) and performance biases, and high risk for incomplete outcome data (<55%) and other biases from failure to adjust for clustering; concerns about **lack of any consistency** shown because only study in analysis; serious concerns about **indirectness** due to use of positivity in those testing rather than prevalence in a representative sample for this outcome ascertainment, and from use of usual care control group; serious concerns about **imprecision** for both the 0.5% and 1.0% important thresholds due to inadequate sample size (for cluster RCT) and the range where the effects may be (95% CI) including effects of no benefit and important harm.

Females in high-risk population; high-intensity CT and NG screening: some concerns that **ROB** from performance and attrition biases may impact findings; there were serious concerns about **indirectness** from the larger study (Garcia) using an outreach approach and offering co-interventions (e.g. mass promotional campaign, motivational interviewing), from the Cohen study using NG positivity for the prevalence estimate, and from both studies having usual care as the control; the sample size may be adequate but we would expect there to be **imprecision** around the reduction such that the effects may be of little to no difference.

Males in high-risk population; moderate-intensity CT & NG screening for females and males: serious concerns about **ROB** from unclear selection (not randomized but groups balanced) and performance biases, and high risk for incomplete outcome data (<55%) and other biases from failure to adjust for clustering; concerns about **lack of any** **consistency** shown because only study in analysis; serious concerns about **indirectness** due to use of positivity rather than representative sample for this outcome ascertainment, and from use of usual care control group; serious concerns about imprecision due to small sample size and wide CI.

**6B GRADE Summary of Findings Table**

| **Outcome**  **No. Participants (studies)** | **Findings** | **Certainty of the evidence (GRADE) (see Explanations below table)** | **What happens?** |
| --- | --- | --- | --- |
| **Transmission: estimated population prevalence of NG (Both sexes in high-risk population)**  Follow-up: 30 mos  3,765 (1 CCT) | The CCT found imprecise results (RR 0.81, 95% CI; 0.11 to 6.10 [adjusted for clustering); 3 fewer in 1000 [15 fewer to 87 more]). | ⊕⊖⊖⊖  VERY LOW due to serious concerns for risk of bias, imprecision and indirectness, some concern about inconsistency | The evidence is very uncertain about the effects on NG prevalence from offering screening for CT and NG biannually to sexes, 15-19 years old at high-risk. |
| **Transmission: estimated population prevalence of NG (Females in high-risk population)**  Follow-up: 30-48 mos  6,000 (1 RCT, 1 CCT) | Both trials showed reductions in NG prevalence (Cohen: RR 0.77, 95% CI 0.12 to 4.84 [adjusted for clustering], 5 fewer in 1000 [20 fewer to 88 more]; Garcia RCT: RRa: 1.44, 95% CI 0.02 to 122 [uncertainty in the model applied], 7 fewer in 1000 [p=0.026]). An estimate of combined findings is of a beneficial effect. | ⊕⊕⊖⊖ to ⊕⊖⊖⊖  LOW-TO-VERY LOW due to some concern about risk of bias and serious concerns about indirectness and imprecision  ⊕⊕⊖⊖  LOW in outreach settings | Frequent offers of screening for CT and NG to high-risk females may reduce NG to a moderate extent in these females. |
| **Transmission: estimated population prevalence of NG (Males in high-risk population)**  Follow-up: 30 mos  1,826 (1 CCT) | The CCT found no benefit for NG prevalence in males (RR 0.96, 95% CI 0.08 to 12.07 [adjusted for clustering], 0.4 fewer in 1000 [10 fewer to 117 more]). | ⊕⊖⊖⊖  VERY LOW due to serious concerns for risk of bias, imprecision and indirectness, some concern about inconsistency | The evidence is very uncertain about the effects on NG prevalence in males from screening both sexes, 15-19 years old at high-risk, for CT and NG biannually. |

Abbreviations: CI=confidence interval; CT=chlamydia trachomatis; CCT= controlled clinical trial; NG=neisseria gonorrhea; RCT=randomized controlled trial; ROB=risk of bias; RR=relative risk

**Explanations:**

Both sexes in high-risk population; moderate-intensity CT and NG screening for females and males: serious concerns about **ROB** from unclear selection (not randomized but groups balanced) and performance biases, and high risk for attrition (>30%) and other biases from failure to adjust for clustering; concerns about **lack of any consistency** shown because only study in analysis; serious concerns about **indirectness** due to use of positivity of those testing rather than prevalence from a representative sample for outcome ascertainment for this outcome ascertainment, and from use of usual care control group; serious concerns about **imprecision** for both the 0.5% and 1.0% important thresholds due to inadequate sample size (for cluster RCT) and the range where the effects may be (95% CI) including effects of no benefit and important harm.

Females in high-risk population; high-intensity CT and NG screening: some concerns that **ROB** from performance and attrition biases may impact findings; there were serious concerns about **indirectness** from the larger study (Garcia) using an outreach approach and offering co-interventions (e.g. mass promotional campaign, motivational interviewing), from the Cohen study using NG positivity for the prevalence estimate, and from both studies having usual care as the control; the sample size may be adequate but we would expect there to be **imprecision** around the reduction such that the effects may be of little to no difference.

Males in high-risk population; moderate-intensity CT & NG screening for females and males: serious concerns about **ROB** from unclear selection (not randomized but groups balanced) and performance biases, and high risk for incomplete outcome data (<55%) and other biases from failure to adjust for clustering; concerns about **lack of any** **consistency** shown because only study in analysis; serious concerns about **indirectness** due to use of positivity rather than representative sample for this outcome ascertainment, and from use of usual care control group; serious concerns about **imprecision** due to small sample size and wide CI.

**Evidence Set 7: Screening vs. no screening; Transmission of CT & NG: Treatment as surrogate (indirect) outcome (# treated/# randomized)**

Included studies: Klovstad 2013 (RCT), Senok 2005 (RCT)

Threshold for important effect: >20 in 1000 more [benefit] or less [harm]

**7A GRADE Evidence Profile Table**

| **Certainty assessment** | | | | | | | **№ of patients assessed for outcome** | | **Effect** | | **Certainty** |
| --- | --- | --- | --- | --- | --- | --- | --- | --- | --- | --- | --- |
| **№ of studies** | **Study design** | **Risk of bias** | **Inconsistency** | **Indirectness** | **Imprecision** | **Other considerations** | **Screening** | **Usual care** | **Relative (95% CI)** | **Absolute (95% CI)** |  |
| **Treatment of CT for both sexes;** **offer of 1 screen to general-risk population** | | | | | | | | | | | |
| 1 | RCT | No concerns | -0.5 | -1.5 | No concerns | No oncerns | 10000 | 31519 | RR 2.53 (1.91 to 3.34) | 5.4 more per 1000 (3.2 to 8.2 more) | ⊕⊕⊖⊖  LOW |
| **Treatment of CT for females; offer of 1 screen to general-risk population** | | | | | | | | | | | |
| 2 | RCT | No concerns | No concerns | -2.0 | No concerns | No concerns | 5183 | 15662 | RR 2.6 (1.86 to 6.64) | 7.2 more per 1000 (3.9 to 11.9) | ⊕⊕⊖⊖  LOW |
| **Treatment of CT for males; offer of 1 screen to general-risk population** | | | | | | | | | | | |
| 1 | RCT | No concerns | No concerns | -2.0 | No concerns | No concerns | 5077 | 16002 | RR 2.47 (1.52 to 4.01) | 3.4 more per 1000 (1.2 to 6.9) | ⊕⊕⊖⊖  LOW |

Abreviatimns: CI=confidence interval; CT= chlamydia trachomatis; CCT=controlled clinical trial; NG=neisseria gonorrhea; RCT=randomized controlled trial; ROB=risk of bias; RR=relative risk

**Explanations:**

**Treatment of CT for both sexes; offer of 1 screen to general-risk population**:No concerns with ROB. Some concern about lack of consistency. Very serious **indirectness** from use of treatment outcome to serve as surrogate for transmission; **no serious imprecision** because the range where the actual effects may be (95% CI) does not include an important increase or decrease to treatment (>20 more or less in 1000) that may translate into an important reduction in transmission.

**Treatment of CT for females; offer of 1 screen to general-risk population:** Some concerns with ROB from Senok but this study contributes very little weight in analysis; very serious i**ndirectness** from use of treatment outcome to serve as proxy for transmission and primary study (Klovstad) not being conducted in primary care; **no serious imprecision** because the range where the actual effects may be (95% CI) does not include an important increase or decrease to treatment (>20 more or less in 1000) that may translate into an important reduction in transmission.

**Treatment of CT for males; offer of 1 screen to general-risk population:** Very serious **indirectness** from use of treatment outcome to serve as proxy for transmission and study not conducted in primary care setting; no serious **imprecision** because the range where the actual effects may be (95% CI) does not include an important increase or decrease to treatment (>20 more or less in 1000) that may translate into an important reduction in transmission.

**7B GRADE Summary of Findings Table**

| **Outcome**  **No. participants (studies)** | **Relative effect (95% CI)** | **Anticipated absolute effects (95% CI)*** | | | **Certainty of the evidence (GRADE)** | **What happens?** |
| --- | --- | --- | --- | --- | --- | --- |
|  |  | **Without screening** | **With a single CT screen** | **Difference** |  |  |
| Transmission: Treatment of CT in both sexes  Follow-up: 3 mos  41,519 (1 RCT) | RR 2.53 (1.91 to 3.34) | 3.5 per 1000 | 8.9 per 1000 (6.7 to 11.7) | 5.4 more per 1000 (3.2 to 8.2 more) | ⊕⊕⊖⊖  LOW due to inconsistency and, indirectness^a^ | Offering a single CT screen to both sexes aged 18-25 may make little to no difference in transmission of CT (5.4 more per 1000 [3.2 to 8.2 more]). |
| Transmission: Treatment of CT in females  Follow-up: 3-4 mos  20,845 (2 RCTs) | RR 2.60 (1.86 to 3.64) | 4.5 per 1000 | 11.7 per 1000 (8.4 to 16.4) | 7.2 more per 1000 (3.9 to 11.9) | ⊕⊕⊖⊖  LOW due to indirectness^b^ | Offering a single CT screen to general-risk females aged 16-30 may make little or no difference in transmission of CT (7.2 more per 1000 [3.9 to 11.9 more]). |
| Transmission: Treatment of CT in males  Follow-up: 3 mos  21,079 (1 RCT) | RR 2.47 (1.52 to 4.01) | 2.3 per 1000 | 5.7 per 1000 (3.5 to 9.2) | 3.4 more per 1000 (1.2 to 6.9) | ⊕⊕⊖⊖  LOW due to indirectness^c^ | Offering a single CT screen to general-risk males aged 18-25 may make little to no difference in the transmission of CT (3.4 more per 1000 [1.2 to 6.9 more]). |

Abbreviations: CI=confidence interval; CT= chlamydia trachomatis; CCT=controlled clinical trial; NG=neisseria gonorrhea; RCT=randomized controlled trial; ROB=risk of bias; RR=relative risk

*The effect with a single CT screen (and its 95% confidence interval) is based on the effect without screening and the relative effect of the intervention (and its 95% CI).

**Explanations:**

**Treatment of CT for both sexes; offer of 1 screen to general-risk population**: No concerns with ROB. Some concern about lack of consistency. Very serious **indirectness** from use of treatment outcome to serve as surrogate for transmission; **no serious imprecision** because the range where the actual effects may be (95% CI) does not include an important increase or decrease to treatment (>20 more or less in 1000) that may translate into an important reduction in transmission.

**Treatment of CT for females; offer of 1 screen to general-risk population:** Some concerns with ROB from Senok but this study contributes very little weight in analysis; very serious i**ndirectness** from use of treatment outcome to serve as proxy for transmission and primary study (Klovstad) not being conducted in primary care; **no serious imprecision** because the range where the actual effects may be (95% CI) does not include an important increase or decrease to treatment (>20 more or less in 1000) that may translate into an important reduction in transmission.

**Treatment of CT for males; offer of 1 screen to general-risk population:** Very serious **indirectness** from use of treatment outcome to serve as proxy for transmission and study not conducted in primary care setting; no serious **imprecision** because the range where the actual effects may be (95% CI) does not include an important increase or decrease to treatment (>20 more or less in 1000) that may translate into an important reduction in transmission.

**7C Forest Plots**

**Treatment of CT for females; offer of 1 screen to general-risk population**

**
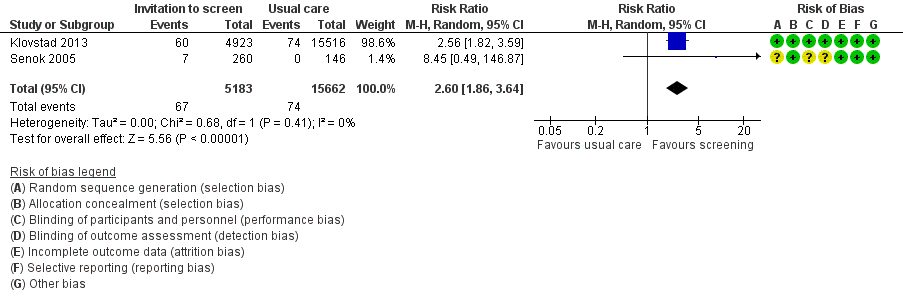
**

**Evidence Set 8: Harms of CT Screening**

Included studies: Hocking 2018 (RCT; serious AEs), Campbell 2006, Gottlieb 2011, Kangas 2006, Gotz 2005, Fielder 2013, Low 2003, Waller 2013 (observational studies)

**8A GRADE Evidence Profile Table**

| **Outcome** | **Certainty assessment** | | | | | | | **Findings** | **Certainty** |
| --- | --- | --- | --- | --- | --- | --- | --- | --- | --- |
|  | **№ of studies & Participants** | **Study design** | **Risk of bias** | **Inconsistency** | **Indirectness** | **Imprecision** | **Other considerations** |  |  |
| Serious Adverse Events from Treatment | 1  N=37,543 tested with 4,574 having CT | RCT | -1.0 | - | - | -2.0 | - | One RCT (Hocking) reported methods of passive surveillance of adverse events from screening and treatment and reported no events. | ⊕⊖⊖⊖  VERY LOW |
| Anxiety-Generalized | 2  N=2,139 | Observational | -0.5 | - | - | - | - | Two studies of CT screening (Campbell and Gottlieb) measured general anxiety related to screening (testing and receiving results) using validated scales (Hospital Anxiety and Depression Scale [HADS; range 0-21] and Brief Symptom Inventory [BSI]; range 0-5 using mean of items for anxiety]). Campbell et al. found that anxiety levels in general-risk participants were not positive for CT were lower during testing (n=397) than before (n=218) receiving the invitation (change in HADS score -0.66 [95% CI -1.23 to -0.09]). Anxiety levels reduced over time (p=0.005). High-risk female patients (n=1,742) had low levels of anxiety during their testing visit, although there was slightly more anxiety for those who were later found to be CT positive than negative (BSI scores 1.52 vs. 1.42, respectively, p=0.08) (Gottlieb). For those with negative CT results and followed up after 4-6 weeks (n=280), receiving results appeared to raise their anxiety symptoms slightly (mean BSI score increased by 0.074; p value for difference from baseline NR) but not beyond a level considered “a little bit” (BSI score of 2). | ⊕⊕⊖⊖  LOW |
| Anxiety –Sexual Aspects of Life | 2  N=1,937 | Observational | - | - | -0.5 (High-risk)  -1.0 (General-risk) | - | - | One study of high-risk females measured anxiety about their sexual aspects of life using the anxiety subscale of the Multidimensional Sexual Self-Concept Questionnaire (MSQ; 5 items with mean scores 1-5 and 1= strongly disagree) (Gottlieb). During the testing process, those who were later found to be CT negative (n=1,593) or positive (n=149) had mean scores that indicated either low or no anxiety (CT negative 1.32 vs CT positive 1.47, p=0.04), although scores for those with a CT infection were slightly higher. Scores for the CT negative females who responded to follow-up 4-6 weeks later (n=280) indicated that anxiety increased a small amount but was still very low (0.09 unit increase, p<0.05) after receiving test results. Of 195 CT-negative respondents who were mostly (78%) seeking screening due to risk factors, few agreed (strongly or somewhat) that they did not feel sexually attractive (7% females and 10% males) right after receiving their results (Kangas). | ⊕⊕⊖⊖  LOW (High-risk)  ⊕⊖⊖⊖  VERY LOW (General-risk) |
| Anxiety about Infertility | 2  N=450 | Observational | -0.5 | -1.0 | -1.0 | - | - | Two studies asked CT-negative people whether they had anxiety about becoming infertile upon receiving their results (Gotz, Kangas). In one study, only 5.4% of 275 respondents agreed to some extent that the results gave them this anxiety (Gotz). In a high-risk sample, between 20-40% of 175 CT-negative respondents felt nervous about their or their partner’s chances of getting pregnant, thought it may be a problem for them or their partner to become pregnant, and/or considered their chances of becoming pregnant different from before (Kangas). Results were similar between sexes. The study reporting more symptoms reported a high (92%) level of knowledge about CT causing infertility, it is unlikely they knew the absolute risk of infertility from a CT infection. | ⊕⊖⊖⊖  VERY LOW |
| Anxiety – other single item questions | 3  N=2,307 | Observational | -1.0 | - | -1.0 | - | - | From asking females, when undergoing their screening test, single questions related to anxiety about testing or CT, two studies indicate that some participants without a CT infection will have concerns about having CT (33% of 1,593)(Gottieb) or feel some anxiety about the testing (n=290; mean 2.0 ± 0.9; 2=somewhat disagree, 3=somewhat agree)(Fielder). Without knowledge of their test results, more infected than non-infected women reported having concerns about CT (46% vs. 33%, p=0.03; n=1,742) or that they thought about CT a lot (20% vs. 10%, p=0.02) (Gottlieb). Coming from a high-risk sample, these findings may better reflect anxiety related to one’s high-risk status rather than from the testing process itself.  Two studies reported on this outcome from follow-up 1-2 months after people received a negative test result (Gottlieb, Gotz). One study with high-risk patients found that concerns about CT for females (n=280) remained and may have increased (40% vs 33%, p=0.07) after the test process (Gottlieb). The other study of general-risk participants found that 12% (n=275) had some worry about their results although the results were not separated by sex (Gotz). | ⊕⊖⊖⊖  VERY LOW |
| Shame/Stigma Symptoms | 4  N=2,542 | Observational | - | - | -0.5 | - | - | (Self-esteem) Using the Rosenburg Self Esteem Scale to indicate possible stigmatization, Campbell et al. found no difference in self-esteem between those responding before (n=218) versus after receiving (n=397) an invitation for screening in a general-risk population. Using the same scale in a high-risk sample, Gottlieb et al. did not find low levels of self-esteem (24 on 0-30 point scale; <15 considered low) at the testing visit (n=1593), and these results did not change at 1-month follow-up after receiving negative results (n=280).  (Symptoms) Two other studies relied on single questions about specific symptoms experienced during the CT screening offer or after receiving negative results. In one study (n=71), 13% and 6% of females and males, respectively, agreed that they felt stigmatized by the offer by their general practitioner of a CT test (Kangas). Of those whom were seeking testing in the same practice due to high-risk behaviours (n=206), 18% of males and 14% of females without a CT infection agreed that they felt stigmatized telling their friends about the test. The other study found that 30% of participants (n=275) did not agree that their social environment would approve of their testing for CT (Gotz). Both studies reported that, upon receiving results, few individuals had feelings of shame (0.8%)(Gotz) or being dirty (1.5% (Gotz); 0% males and 7% females of n=195 (Kangas)). | ⊕⊕⊖⊖  LOW |
| Embarrassment | 4  N=1,353 | Observational | -0.5 | - | -0.5 | - | - | Two studies (n=399) indicate that undergoing a screening test in an outreach setting using either self-collected swabs (1.7 ±0.8 on 4-point Likert, Fielder) or urine samples (7% Low) may not be embarrassing for most 18-21 year olds. The screening in Low et al. was accompanied by an interactive educational intervention about STIs and screening developed as a “non-stigmatizing” intervention. Findings in primary care, also using urine or self-collected swabs, generally agree with this: 17% and 12% of CT positive (n=805) and negative (n=67) patients, respectively, stated that they would feel embarrassed undergoing future screening (Walker). In another study of screening high-risk populations in primary care (n=82), upon receiving their test results few of those without a CT infection felt embarrassed about the results (3%)(Kangas). | ⊕⊖⊖⊖  VERY LOW |
| Guilt | 0 | - | - | - | - | - | - | None of the included studies reported on this outcome as relates to the CT screening process. |  |
| Relationship Violence | 0 | - | - | - | - | - | - | We did not locate any studies that reported on violence related to the screening process. |  |
| Relationship Break-up | 2  N=445 | Observational | - | - | -0.5 (High-risk)  -1.0  (Genera-risk) | - | - | In one study with high-risk participants, one female (n=122) and no male (n=73) reported a break-up with a partner as a result of their negative CT test result (Kangas). Another study asking a high-risk sample of females without CT about break-up with a main partner within the past 30 days, both at baseline and at 4-6 week followup, found a similar number (11% of 250) of break-ups in both time periods (Gottlieb). | ⊕⊕⊖⊖  LOW (High-risk)  ⊕⊖⊖⊖  VERY LOW (General-risk) |
| Relationship Distress | 2  N=1,000 | Observational | - | -1.0 | -0.5 | - | - | Very few (≤5% of 195) high-risk individuals of both sexes in one study reported that their partner was upset or disappointed about their screening (Kangas). In the other study, several (25% of 805) general-risk females without CT felt concerned about their partner’s reaction to having another test in the future (Walker). | ⊕⊖⊖⊖  VERY LOW |

Abbreviations: AE: adverse event; CT: chlamydia Chlamydia trachomatis; N: sample size; NR: not reported; RCT: randomized controlled trial; ROB: risk of bias

**Explanations:**

**Serious Adverse Events from Treatment**: Serious concerns about **ROB** from lack of active harms surveillance. Very serious concerns about **imprecision** due to small sample for this very rare event.

**Anxiety-Generalized**: Some concern about **ROB** from inadequate length of follow-up for this outcome.

**Anxiety –Sexual Aspects of Life:** Some concerns about **indirectness** due to Kangas study being largely symptomatic even though only 20% seeking testing due to this, and for indirectness to general-risk population.

**Anxiety about Infertility**: Some concern about **ROB** due to selection bias in Gotz. Serious concerns about **unexplained inconsistency** between magnitudes of effects. Serious concerns about **indirectness** due to symptomatic population in Kangas and lack of informed responses.

**Anxiety – other single item questions**: Serious concerns about **ROB** from selection bias and poor attribution to screening. Serious **indirectness** for men and general-risk populations.

**Shame/Stigma Symptoms**: Some concern about **ROB** from Gotz but no impact on overall conclusions. Some concern about **indirectness** from use of self-esteem for stigma and about population (20% seeking care for symptoms) in Kangas.

**Embarrassment**: Some concern about **ROB** from selection bias. Some concern about **indirectness** from outreach setting for two studies and population in Kangas.

**Relationship Break-up**: Some concerns about **indirectness** to general-risk population in Kangas and othe high-risk population.

**Relationship Distress**: Serious concerns about **inconsistency** in magnitude of effects. Some concerns about **indirectness** from use of anticipated reactions in Walker.

**8B GRADE Summary of Findings Table**

| **Outcome**  **No. Participants (studies)** | **Findings** | **Certainty of the evidence (GRADE) (see Explanations below table)** | **What happens?** |
| --- | --- | --- | --- |
| Serious Adverse Events from Treatment  4,574 receiving a positive diagnosis (1 RCT) | One RCT (Hocking) reported methods of passive surveillance of adverse events from screening and treatment and reported no events. | ⊕⊖⊖⊖  VERY LOW due to ROB and imprecision | The evidence about serious adverse effects from CT screening is uncertain (no events). |
| Anxiety-Generalized  2,139 (2 studies) | Two studies of CT screening (Campbell and Gottlieb) measured general anxiety related to screening (testing and receiving results) using validated scales (Hospital Anxiety and Depression Scale [HADS; range 0-21] and Brief Symptom Inventory [BSI]; range 0-5 using mean of items for anxiety]). Campbell et al. found that anxiety levels in general-risk participants were not positive for CT were lower during testing (n=397) than before (n=218) receiving the invitation (change in HADS score -0.66 [95% CI -1.23 to -0.09]). Anxiety levels reduced over time (p=0.005). High-risk female patients (n=1,742) had low levels of anxiety during their testing visit, although there was slightly more anxiety for those who were later found to be CT positive than negative (BSI scores 1.52 vs. 1.42, respectively, p=0.08) (Gottlieb). For those with negative CT results and followed up after 4-6 weeks (n=280), receiving results appeared to raise their anxiety symptoms slightly (mean BSI score increased by 0.074; p value for difference from baseline NR) but not beyond a level considered “a little bit” (BSI score of 2). | ⊕⊕⊖⊖  LOW with some concern about ROB | Screening for CT in primary care may make little to no difference for generalized anxiety in high-risk or general-risk individuals over the short-term. |
| Anxiety –Sexual Aspects of Life  1,937 (2 studies) | One study of high-risk females measured anxiety about their sexual aspects of life using the anxiety subscale of the Multidimensional Sexual Self-Concept Questionnaire (MSQ; 5 items with mean scores 1-5 and 1= strongly disagree) (Gottlieb). During the testing process, those who were later found to be CT negative (n=1,593) or positive (n=149) had mean scores that indicated either low or no anxiety (CT negative 1.32 vs CT positive 1.47, p=0.04), although scores for those with a CT infection were slightly higher. Scores for the CT negative females who responded to follow-up 4-6 weeks later (n=280) indicated that anxiety increased a small amount but was still very low (0.09 unit increase, p<0.05) after receiving test results. Of 195 CT-negative respondents who were mostly (78%) seeking screening due to risk factors, few agreed (strongly or somewhat) that they did not feel sexually attractive (7% females and 10% males) right after receiving their results (Kangas). | ⊕⊕⊖⊖  LOW (High-risk) with some indirectness  ⊕⊖⊖⊖  VERY LOW (General-risk) | Screening for CT may make little to no difference in anxiety about one’s sexual aspects of life over the short-term, for people who are in high-risk groups. The effects for people at general-risk for CT are very uncertain. |
| Anxiety about Infertility  450 (2 studies) | Two studies asked CT-negative people whether they had anxiety about becoming infertile upon receiving their results (Gotz, Kangas). In one study, only 5.4% of 275 respondents agreed to some extent that the results gave them this anxiety (Gotz). In a high-risk sample, between 20-40% of 175 CT-negative respondents felt nervous about their or their partner’s chances of getting pregnant, thought it may be a problem for them or their partner to become pregnant, and/or considered their chances of becoming pregnant different from before (Kangas). Results were similar between sexes. The study reporting more symptoms reported a high (92%) level of knowledge about CT causing infertility, it is unlikely they knew the absolute risk of infertility from a CT infection. | ⊕⊖⊖⊖  VERY LOW from ROB, inconsistency and indirectness | A small-to-moderate proportion (5-40%) of people screening for CT may experience some anxiety about the impact of CT on their or their partner’s future fertility. However, the evidence is uncertain particularly about the duration of effects.  It is unknown if this effect represents the experience of those well-informed about the absolute risk for infertility from CT. |
| Anxiety – other single item questions  2,307 (3 studies) | From asking females, when undergoing their screening test, single questions related to anxiety about testing or CT, two studies indicate that some participants without a CT infection will have concerns about having CT (33% of 1,593)(Gottieb) or feel some anxiety about the testing (n=290; mean 2.0 ± 0.9; 2=somewhat disagree, 3=somewhat agree)(Fielder). Without knowledge of their test results, more infected than non-infected women reported having concerns about CT (46% vs. 33%, p=0.03; n=1,742) or that they thought about CT a lot (20% vs. 10%, p=0.02) (Gottlieb). Coming from a high-risk sample, these findings may better reflect anxiety related to one’s high-risk status rather than from the testing process itself.  Two studies reported on this outcome from follow-up 1-2 months after people received a negative test result (Gottlieb, Gotz). One study with high-risk patients found that concerns about CT for females (n=280) remained and may have increased (40% vs 33%, p=0.07) after the test process (Gottlieb). The other study of general-risk participants found that 12% (n=275) had some worry about their results although the results were not separated by sex (Gotz). | ⊕⊖⊖⊖  VERY LOW from ROB and indirectness | Screening may make a small-to-moderate (10-46%) number of people feel concern or anxiety about CT; the evidence is uncertain, particularly for men and those without risk factors for CT. Feelings of concern and worry about CT may persist after receiving a negative result. |
| Shame/Stigma Symptoms  Generalized Stigma: 1,990 (2 studies)  Symptoms: 552 (2 studies) | (Self-esteem) Using the Rosenburg Self Esteem Scale to indicate possible stigmatization, Campbell et al. found no difference in self-esteem between those responding before (n=218) versus after receiving (n=397) an invitation for screening in a general-risk population. Using the same scale in a high-risk sample, Gottlieb et al. did not find low levels of self-esteem (24 on 0-30 point scale; <15 considered low) at the testing visit (n=1593), and these results did not change at 1-month follow-up after receiving negative results (n=280).  (Symptoms) Two other studies relied on single questions about specific symptoms experienced during the CT screening offer or after receiving negative results. In one study (n=71), 13% and 6% of females and males, respectively, agreed that they felt stigmatized by the offer by their general practitioner of a CT test (Kangas). Of those whom were seeking testing in the same practice due to high-risk behaviours (n=206), 18% of males and 14% of females without a CT infection agreed that they felt stigmatized telling their friends about the test. The other study found that 30% of participants (n=275) did not agree that their social environment would approve of their testing for CT (Gotz). Both studies reported that, upon receiving results, few individuals had feelings of shame (0.8%)(Gotz) or being dirty (1.5% (Gotz); 0% males and 7% females of n=195 (Kangas)). | ⊕⊕⊖⊖  LOW with some indirectness | Over the short-term, stigma as manifested in low levels of overall self-esteem may not be caused by CT screening.  Symptoms related to stigmatization, particularly within one’s social circle, may be experienced by a small-to-moderate number (6-30%) of individuals who undertake screening, although the effects may be transient for most. |
| Embarrassment  1,353 (4 studies) | Two studies (n=399) indicate that undergoing a screening test in an outreach setting using either self-collected swabs (1.7 ±0.8 on 4-point Likert, Fielder) or urine samples (7% Low) may not be embarrassing for most 18-21 year olds. The screening in Low et al. was accompanied by an interactive educational intervention about STIs and screening developed as a “non-stigmatizing” intervention. Findings in primary care, also using urine or self-collected swabs, generally agree with this: 17% and 12% of CT positive (n=805) and negative (n=67) patients, respectively, stated that they would feel embarrassed undergoing future screening (Walker). In another study of screening high-risk populations in primary care (n=82), upon receiving their test results few of those without a CT infection felt embarrassed about the results (3%)(Kangas). | ⊕⊖⊖⊖  VERY LOW from ROB and indirectness | CT screening using self-collected samples may cause embarrassment for a small number of people, but the evidence is uncertain. |
| Guilt | None of the included studies reported on this outcome as relates to the CT screening process. |  |  |
| Relationship Violence | We did not locate any studies that reported on violence related to the screening process. |  |  |
| Relationship Break-up  445 (2 studies) | In one study with high-risk participants, one female (n=122) and no male (n=73) reported a break-up with a partner as a result of their negative CT test result (Kangas). Another study asking a high-risk sample of females without CT about break-up with a main partner within the past 30 days, both at baseline and at 4-6 week followup, found a similar number (11% of 250) of break-ups in both time periods (Gottlieb). | ⊕⊕⊖⊖  LOW (High-risk) from indirectness  ⊕⊖⊖⊖  VERY LOW (General-risk) | In high-risk individuals, there may be little to no effects on relationship break-up as a direct consequence of undergoing screening (2 studies, n=445; low certainty). The effects for a general-risk population are uncertain. |
| Relationship Distress  1,000 (2 studies) | Very few (≤5% of 195) high-risk individuals of both sexes in one study reported that their partner was upset or disappointed about their screening (Kangas). In the other study, several (25% of 805) general-risk females without CT felt concerned about their partner’s reaction to having another test in the future (Walker). | ⊕⊖⊖⊖  VERY LOW from inconsistency and indirectness | Findings on the effects from CT screening on general relationship distress are uncertain, but suggest that responses from partners about screening may not be very negative and may be better than anticipated (2 studies, n=1,000; very low certainty). |

Abbreviations: AE: adverse event; CT: chlamydia Chlamydia trachomatis; N: sample size; NR: not reported; RCT: randomized controlled trial; ROB: risk of bias

**Explanations:**

**Serious Adverse Events from Treatment**: Serious concerns about **ROB** from lack of active harms surveillance. Very serious concerns about **imprecision** due to small sample for this very rare event.

**Anxiety-Generalized**: Some concern about **ROB** from inadequate length of follow-up for this outcome.

**Anxiety –Sexual Aspects of Life:** Some concerns about **indirectness** due to Kangas study being largely symptomatic even though only 20% seeking testing due to this, and for indirectness to general-risk population.

**Anxiety about Infertility**: Some concern about **ROB** due to selection bias in Gotz. Serious concerns about **unexplained inconsistency** between magnitudes of effects. Serious concerns about **indirectness** due to symptomatic population in Kangas and lack of informed responses.

**Anxiety – other single item questions**: Serious concerns about **ROB** from selection bias and poor attribution to screening. Serious **indirectness** for men and general-risk populations.

**Shame/Stigma Symptoms**: Some concern about **ROB** from Gotz but no impact on overall conclusions. Some concern about **indirectness** from use of self-esteem for stigma and about population (20% seeking care for symptoms) in Kangas.

**Embarrassment**: Some concern about **ROB** from selection bias. Some concern about **indirectness** from outreach setting for two studies and population in Kangas.

**Relationship Break-up**: Some concerns about **indirectness** to general-risk population in Kangas and othe high-risk population.

**Relationship Distress**: Serious concerns about **inconsistency** in magnitude of effects. Some concerns about **indirectness** from use of anticipated reactions in Walker.

**Evidence Set 9: Harms of a CT Diagnosis**

Included studies: Andersson 2018, Gottlieb 2011, Kangas 2006, Gotz 2005, Walker 2013, France 2001, O’Farrell 2013

**9A GRADE Evidence Profile Table**

| **Outcome** | **Certainty assessment** | | | | | | | **Findings** | **Certainty** |
| --- | --- | --- | --- | --- | --- | --- | --- | --- | --- |
|  | **№ of studies & Participants** | **Study design** | **Risk of bias** | **Inconsistency** | **Indirectness** | **Imprecision** | **Other considerations** |  |  |
| Anxiety -Generalized | 2  N=277 |  | -0.5 | -1.0 | -0.5 | -0.5 | - | Andersson et al. (n=128) found anxiety symptoms or clinically significant anxiety (≥7 or ≥10 out of 20 on Hospital Anxiety and Depression Scale [HADS]) in several people but particularly in females (symptoms: 19% males and 48% females [p<0.001]; clinical: 8.5% males and 31% females [p=0.002]) during their counselling for partner tracing after receiving treatment for CT. Without measurement of their anxiety levels before the diagnosis, the contribution to these levels from the CT diagnosis is unknown for this sample. Moreover, findings from a study using the HADS across timepoints from before a screening invitation to after receiving results indicated that several CT negative females would also have anxiety symptoms or clinical anxiety (HADS score 8.14 ± 3.89) (Campbell). Females with CT in the study by Gottlieb et al. reported low anxiety levels (Brief Symptom Inventory [BSI]; range 0-5 using mean of items for anxiety; mean BSI 1.52 and 1.51) when testing (but unaware of their CT status; n=149) and after receiving their results (n=71), respectively. | ⊕⊖⊖⊖  VERY LOW |
| Anxiety – Sexual Aspects of Life | 3  N=359 | Observational | - | -0.5 | -1.0 (General-risk and males) | -0.5 | - | For high-risk females with a CT infection, anxiety about their sexual aspects of life increased between the testing visit (n=149) and one-month follow-up (n=71) (Multidimensional Sexual Self-Concept Questionnaire; 0.34 units higher, p<0.001) (Gottlieb). The increase was greater than for those receiving negative results (0.086 units, p=0.02) but still reflected low overall anxiety for most respondents. A moderate proportion of 82 CT-positive patients reported that they did not feel sexually attractive after first gaining knowledge of their infection (37% and 18% in females and males, respectively); the difference compared with CT negative females was higher (30%, p<0.001) than for CT negative males (8%, p=0.45) (Kangas).  Two studies asked high-risk CT-positive patients, soon after their diagnosis, about whether it would be hard to trust future partners (5-point Likert with 1=strongly disagree; 3=neutral; 5=strongly agree) (Andersson, Gottlieb). One study found that females and males (n=128) were generally neutral or did not think it would be difficult to trust (mean scores 2.6 and 2.3, p=0.24) (Andersson), but findings indicate at least a small proportion would agree. Gottlieb et al. found that 87% of females (n=71) reported some difficulty. | ⊕⊕⊖⊖  VERY LOW |
| Anxiety about Infertility | 6  N=428 | Observational | - | - | - | - | - | Findings across studies of general- and high-risk populations suggest that a moderate number of females (about 40-60%) diagnosed with CT will experience some degree of worry, anxiety, and/or nervousness about their future infertility (Andersson, France, Gottieb, Gotz, Kangas, Walker). Men may have less, but still some, anxiety about their, or their partner’s, ability to have children in the future (Andersson). Because of findings from two studies indicating some of this anxiety in people without CT based on the screening (Gotz, Kangas), full attribution of the findings to the diagnosis is unlikely. Most of the people in these studies would have already received treatment for their CT infection. No study reported providing information about the proportion of females or males that will become infertile from one or more CT infections. | ⊕⊕⊖⊖  LOW |
| Anxiety – other single item questions | 3  N=292 | Observational | -1.0 | - | - | -0.5 | - | In a high-risk sample of females (n=71), 80%, 37%, and 75% agreed that they were concerned about the result, worried that the infection would not go away, or worried that they would get the infection again, respectively (Gottlieb). More CT positive females (n=149) were concerned about CT after receiving their diagnosis than during the testing (80% vs 46%, p value not reported). In general-risk populations, 75% of 76 individuals with CT (vs. 12% of 275 without CT) had some worry about the result (Gotz), and 79%, 56%, and 71% of 67 CT-positive females agreed somewhat or strongly that they felt anxious about the result, were worried people would treat them differently, or were afraid of the result, respectively (Walker). | ⊕⊖⊖⊖  VERY LOW |
| Shame/Stigma | 5  N=502 | Observational | - | -0.5 | -0.5 (Generalized Stigma) | -1.0  (Generalized Stigma) | - | (Self-esteem) Similar to their sample without a CT infection, overall self-esteem using the Rosensburg Self-Esteem Scale did not change from the testing visit (n=149; 23 on 0-30 point scale; <15 considered low) to 1-month follow-up (n=71)(Gottlieb).  (Symptoms) Findings from three studies generally indicate that a moderate number (about 25-50%) of those infected with CT may feel dirty, shame, or that they did not take care of themselves (Gottlieb, Gotz, Kangas) Another study’s findings indicated that on average people did not feel dirty (2.5 on 5-point Likert with 1=completely agree; no measure of variance)(Andersson). Findings were similar for stigma anticipated from one’s social circle. Two studies reported that a number of participants reported stigma related to telling their friends about the test (38%, n=82)(Kangas) or about worry that people would treat them differently (56%, n=67) (Walker), but another only reported that on average people did not feel that the result changed the way their friends saw them (4.1 on 5-point Likert scale, no measure of variance n=128)(Andersson). | ⊕⊖⊖⊖  VERY LOW (Generalized stigma)  ⊕⊕⊖⊖  LOW (Symptoms) |
| Embarrassment | 5  N=348 | Observational | - | - | - | -0.5 | - | Results across studies indicate that a moderate proportion (40-65%) of female patients will feel some embarrassment about their positive CT result (Andersson, Gottlieb, Kangas, Walker). In one study, 55% of women agreed that they felt “really embarrassed” (Gottlieb). Two studies found that males will also experience this feeling, but with somewhat less frequency than females (Andersson, Kangas). A very small study (n=4) reported that 75% of the females reported feeling embarrassed about the need to trace contacts (France). | ⊕⊕⊖⊖  LOW |
| Guilt | 2  N=278 | Observational | - | - | - | -0.5 | - | Two studies with participants being at high-risk (n=278) reported results about feelings of guilt (about passing their infection to someone else) shortly after receiving a CT diagnosis (3.2 on 5-point Likert by Andersson, 54% by Gottlieb)**.** At least this number of people may feel not proud of their actions (3.3 on 5-point Likert by Andersson, 74% by Gottlieb). | ⊕⊕⊖⊖  LOW |
| Relationship Violence | 1  N=298 | Observational | - | -0.5 | -0.5 | -1.0 | - | Three-to-twelve months after an STI clinic appointment where participants were either diagnosed with CT or not diagnosed with any STI, 3% (n=149) and 0% (n=149), respectively, reported physical violence as related to their CT diagnosis or clinic attendance (O’Farrell). | ⊕⊖⊖⊖  VERY LOW |
| Relationship Break-up | 5  N=1,070 | Observational | - | - | -1.0  (General-risk) | - | - | Findings from three studies (n=895) of screening of high-risk populations suggest that a CT diagnosis may lead to relationship break-up for a small proportion (about 5-10%) of people (Gottlieb, Kangas, O’Farrell). Findings from another study (n=99) appear somewhat contradictory, with mean scores from a scale (1.4 on 5-point Likert) suggesting no break-ups for either sex (Andersson). One study (n=76) of a general-risk population reported that 3 of 76 infected women mentioned the end of a relationship, although all noted that it had been bad anyhow (Gotz). | ⊕⊕⊖⊖  LOW (High-risk)  ⊕⊖⊖⊖  VERY LOW (General-risk) |
| Relationship Distress | 5  553 | Observational | - | - | -0.5 | - | - | The two studies (n=143) of general-risk populations found that many (40% (Gotz) and 62% (Walker) people, mostly females, expected a negative influence on their partnership or felt worried about their partner’s response. Despite this, in one of the studies 92% reported receiving sympathy from their partner (Gotz). Results about overall relationship quality and feelings of betrayal from two studies of high-risk populations were conflicting. Gottlieb et al. (n=71) found that 62% and 70% of their female participants thought the diagnosis changed their relationship or felt betrayed by their partner, respectively, while Andersson et al.’s (n=62) findings suggest that most males and females had not experienced these consequences (1.8 on 5-point Likert; measure of variance not reported). As opposed to feeling betrayed by a partner, two studies suggest that a moderate number of partners will be upset (about 30%), but not necessarily disappointed by the information (n=277) (Kangas), and that many would not assume their partner had cheated on them (1.5 on 5-point Likert; n=62) (Andersson). | ⊕⊕⊖⊖  LOW |

CT: chlamydia trachomatis

**Explanations:**

**Anxiety –Generalized**: Some concerns about **ROB** from duration of follow-up and selection bias in Andersson. Serious concerns about **inconsistency** in magnitude of effects. Some concerns about **indirectness** to general-risk population and **for imprecision** due to small sample size.

**Anxiety – Sexual Aspects of Life**: Some concerns about **inconsistency** between studies. Serious concerns for **indirectness** to general-risk population and to males. Some concerns about **imprecision** from small samples.

**Anxiety about Infertility**: No serious concerns.

**Anxiety – other single item questions**: Serious concerns about **ROB** from selection bias and lack of comparator. Some concern about **imprecision** from sample size.

**Shame/Stigma**: Serious concerns with **indirectness** (use of self-esteem) and **imprecision** (small sample) for generalized stigma. For symptoms, there was some concern with **inconsistency** from the Andersson study.

**Embarrassment**: Some inconsistency but explained by sex. Some concern for **imprecision** from small sample sizes.

**Guilt** Some concerns with imprecision from small samples.

**Relationship Violence**: Some concerns about **lack of consistency** due to one study and about i**ndirectness** with question asked about CT diagnosis or STI clinic attendance. Serious concerns about **imprecision** from small sample.

**Relationship Break-up**: Serious concerns for **indirectness** to general-risk population.

**Relationship Distress**: Some concerns that findings from to studies are about anticipated distress.

**9B GRADE Summary of Findings Table**

| **Outcome**  **No. Participants (studies)** | **Findings** | **Certainty of the evidence (GRADE) (see Explanations below table)** | **What happens?** |
| --- | --- | --- | --- |
| Anxiety –Generalized  277 (2 studies) | Andersson et al. (n=128) found anxiety symptoms or clinically significant anxiety (≥7 or ≥10 out of 20 on Hospital Anxiety and Depression Scale [HADS]) in several people but particularly in females (symptoms: 19% males and 48% females [p<0.001]; clinical: 8.5% males and 31% females [p=0.002]) during their counselling for partner tracing after receiving treatment for CT. Without measurement of their anxiety levels before the diagnosis, the contribution to these levels from the CT diagnosis is unknown for this sample. Moreover, findings from a study using the HADS across timepoints from before a screening invitation to after receiving results indicated that several CT negative females would also have anxiety symptoms or clinical anxiety (HADS score 8.14 ± 3.89) (Campbell). Females with CT in the study by Gottlieb et al. reported low anxiety levels (Brief Symptom Inventory [BSI]; range 0-5 using mean of items for anxiety; mean BSI 1.52 and 1.51) when testing (but unaware of their CT status; n=149) and after receiving their results (n=71), respectively. | ⊕⊖⊖⊖  VERY LOW due to ROB, inconsistency, indirectness, and imprecision | A CT diagnosis may make little to no difference in general anxiety levels. |
| Anxiety about Sexual Aspects of Life  359 (3 studies) | For high-risk females with a CT infection, anxiety about their sexual aspects of life increased between the testing visit (n=149) and one-month follow-up (n=71) (Multidimensional Sexual Self-Concept Questionnaire; 0.34 units higher, p<0.001) (Gottlieb). The increase was greater than for those receiving negative results (0.086 units, p=0.02) but still reflected low overall anxiety for most respondents. A moderate proportion of 82 CT-positive patients reported that they did not feel sexually attractive after first gaining knowledge of their infection (37% and 18% in females and males, respectively); the difference compared with CT negative females was higher (30%, p<0.001) than for CT negative males (8%, p=0.45) (Kangas).  Two studies asked high-risk CT-positive patients, soon after their diagnosis, about whether it would be hard to trust future partners (5-point Likert with 1=strongly disagree; 3=neutral; 5=strongly agree) (Andersson, Gottlieb). One study found that females and males (n=128) were generally neutral or did not think it would be difficult to trust (mean scores 2.6 and 2.3, p=0.24) (Andersson), but findings indicate at least a small proportion would agree. Gottlieb et al. found that 87% of females (n=71) reported some difficulty. | ⊕⊖⊖⊖  VERY LOW due to inconsistency, indirectness (general-risk), and imprecision | A diagnosis of CT may cause one or more symptoms of anxiety about one’s sexual aspects of life in a small-to-moderate (10-30%) number of people at high-risk for CT screening, but the evidence is uncertain particularly for people at general risk and for males. |
| Anxiety about Infertility  428 (6 studies) | Findings across studies of general- and high-risk populations suggest that a moderate number of females (about 40-60%) diagnosed with CT will experience some degree of worry, anxiety, and/or nervousness about their future infertility (Andersson, France, Gottieb, Gotz, Kangas, Walker). Men may have less, but still some, anxiety about their, or their partner’s, ability to have children in the future (Andersson). Because of findings from two studies indicating some of this anxiety in people without CT based on the screening (Gotz, Kangas), full attribution of the findings to the diagnosis is unlikely. Most of the people in these studies would have already received treatment for their CT infection. No study reported providing information about the proportion of females or males that will become infertile from one or more CT infections. | ⊕⊕⊖⊖  LOW | A diagnosis of CT may lead to a moderate-to-large (40-60%) number of people, females more so than males, feel short-term anxiety about their future ability to have children. |
| Anxiety – other single item questions  292 (3 studies) | In a high-risk sample of females (n=71), 80%, 37%, and 75% agreed that they were concerned about the result, worried that the infection would not go away, or worried that they would get the infection again, respectively (Gottlieb). More CT positive females (n=149) were concerned about CT after receiving their diagnosis than during the testing (80% vs 46%, p value not reported). In general-risk populations, 75% of 76 individuals with CT (vs. 12% of 275 without CT) had some worry about the result (Gotz), and 79%, 56%, and 71% of 67 CT-positive females agreed somewhat or strongly that they felt anxious about the result, were worried people would treat them differently, or were afraid of the result, respectively (Walker). | ⊕⊖⊖⊖  VERY LOW from ROB and imprecision | Receiving a diagnosis of CT may cause one or more symptoms related to anxiety for a moderate-to-large (40-80%) proportion of people, but the evidence is uncertain and duration unknown. |
| Shame/Stigma  Generalized stigma: 149 (1 study)  Symptoms: 502 (5 studies) | (Self-esteem) Similar to their sample without a CT infection, overall self-esteem using the Rosensburg Self-Esteem Scale did not change from the testing visit (n=149; 23 on 0-30 point scale; <15 considered low) to 1-month follow-up (n=71)(Gottlieb).  (Symptoms) Findings from three studies generally indicate that a moderate number (about 25-50%) of those infected with CT may feel dirty, shame, or that they did not take care of themselves (Gottlieb, Gotz, Kangas) Another study’s findings indicated that on average people did not feel dirty (2.5 on 5-point Likert with 1=completely agree; no measure of variance)(Andersson). Findings were similar for stigma anticipated from one’s social circle. Two studies reported that a number of participants reported stigma related to telling their friends about the test (38%, n=82)(Kangas) or about worry that people would treat them differently (56%, n=67) (Walker), but another only reported that on average people did not feel that the result changed the way their friends saw them (4.1 on 5-point Likert scale, no measure of variance n=128)(Andersson). | ⊕⊖⊖⊖  VERY LOW (Generalized stigma) from indirectness and imprecision  ⊕⊕⊖⊖  LOW (Symptoms) from inconsistency | A CT diagnosis may make little to no difference for generalized stigma as manifested in low self-esteem, but the evidence is uncertain.  One or more specific aspects of shame or anticipated stigma, a such as feeling dirty or worried about their friends’ reactions, may be felt by a moderate number (20-50%) of individuals upon receiving their positive CT results. |
| Embarrassment  348 (4 studies) | Results across studies indicate that a moderate proportion (40-65%) of female patients will feel some embarrassment about their positive CT result (Andersson, Gottlieb, Kangas, Walker). In one study, 55% of women agreed that they felt “really embarrassed” (Gottlieb). Two studies found that males will also experience this feeling, but with somewhat less frequency than females (Andersson, Kangas). A very small study (n=4) reported that 75% of the females reported feeling embarrassed about the need to trace contacts (France). | ⊕⊕⊖⊖  LOW with some imprecision | A CT diagnosis may cause many females (40-65%), and some males, to feel embarrassed. |
| Guilt  278 (2 studies) | Two studies with participants being at high-risk (n=278) reported results about feelings of guilt (about passing their infection to someone else) shortly after receiving a CT diagnosis (3.2 on 5-point Likert by Andersson, 54% by Gottlieb)**.** At least this number of people may feel not proud of their actions (3.3 on 5-point Likert by Andersson, 74% by Gottlieb). | ⊕⊕⊖⊖  LOW with some imprecision | A moderate-to-large (20-70%) proportion of people with CT will feel some degree of guilt. |
| Relationship Violence  298 (1 study) | Three-to-twelve months after an STI clinic appointment where participants were either diagnosed with CT or not diagnosed with any STI, 3% (n=149) and 0% (n=149), respectively, reported physical violence as related to their CT diagnosis or clinic attendance (O’Farrell). | ⊕⊖⊖⊖  VERY LOW due to lack of consistency, indirectness, and imprecision | The evidence about the effects on relationship violence from screening that results in a diagnosis of CT are uncertain. |
| Relationship Break-up  1,070 (5 studies) | Findings from three studies (n=895) of screening of high-risk populations suggest that a CT diagnosis may lead to relationship break-up for a small proportion (about 5-10%) of people (Gottlieb, Kangas, O’Farrell). Findings from another study (n=99) appear somewhat contradictory, with mean scores from a scale (1.4 on 5-point Likert) suggesting no break-ups for either sex (Andersson). One study (n=76) of a general-risk population reported that 3 of 76 infected women mentioned the end of a relationship, although all noted that it had been bad anyhow (Gotz). | ⊕⊕⊖⊖  LOW (High-risk)  ⊕⊖⊖⊖  VERY LOW (General-risk) due to indirectness | A CT diagnosis may lead to relationship break-up for a small proportion (about 5-10%) of people in high-risk populations. The evidence for the effects in general-risk populations are uncertain. |
| Relationship Distress  553 (5 studies) | The two studies (n=143) of general-risk populations found that many (40% (Gotz) and 62% (Walker) people, mostly females, expected a negative influence on their partnership or felt worried about their partner’s response. Despite this, in one of the studies 92% reported receiving sympathy from their partner (Gotz). Results about overall relationship quality and feelings of betrayal from two studies of high-risk populations were conflicting. Gottlieb et al. (n=71) found that 62% and 70% of their female participants thought the diagnosis changed their relationship or felt betrayed by their partner, respectively, while Andersson et al.’s (n=62) findings suggest that most males and females had not experienced these consequences (1.8 on 5-point Likert; measure of variance not reported). As opposed to feeling betrayed by a partner, two studies suggest that a moderate number of partners will be upset (about 30%), but not necessarily disappointed by the information (n=277) (Kangas), and that many would not assume their partner had cheated on them (1.5 on 5-point Likert; n=62) (Andersson). | ⊕⊕⊖⊖  LOW with some indirectness | A CT diagnosis may cause some relationship distress for a small-to-moderate (100-500 per 1000) number of people. |

CT: chlamydia trachomatis; ROB: risk of bias

**Explanations:**

**Anxiety –Generalized**: Some concerns about **ROB** from duration of follow-up and selection bias in Andersson. Serious concerns about **inconsistency** in magnitude of effects. Some concerns about **indirectness** to general-risk population and **for imprecision** due to small sample size.

**Anxiety – Sexual Aspects of Life**: Some concerns about **inconsistency** between studies. Serious concerns for **indirectness** to general-risk population and to males. Some concerns about **imprecision** from small samples.

**Anxiety about Infertility**: No serious concerns.

**Anxiety – other single item questions**: Serious concerns about **ROB** from selection bias and lack of comparator. Some concern about **imprecision** from sample size.

**Shame/Stigma**: Serious concerns with **indirectness** (use of self-esteem) and **imprecision** (small sample) for generalized stigma. For symptoms, there was some concern with **inconsistency** from the Andersson study.

**Embarrassment**: Some inconsistency but explained by sex. Some concern for **imprecision** from small sample sizes.

**Guilt** Some concerns with imprecision from small samples.

**Relationship Violence**: Some concerns about **lack of consistency** due to one study and about i**ndirectness** with question asked about CT diagnosis or STI clinic attendance. Serious concerns about **imprecision** from small sample.

**Relationship Break-up**: Serious concerns for **indirectness** to general-risk population.

**Relationship Distress**: Some concerns that findings from to studies are about anticipated distress.

**Evidence Set 10 Different screening approaches; Transmission of CT & NG: Incidence of CT & NG**

Included study: Cook 2007 (RCT)

**10A GRADE Evidence Profile Table**

| **Certainty assessment** | | | | | | | **Findings** | **Certainty** |
| --- | --- | --- | --- | --- | --- | --- | --- | --- |
| **№ of studies** | **Study design** | **Risk of bias** | **Inconsistency** | **Indirectness** | **Imprecision** | **Other considerations** |  |  |
| **Incidence of CT & NG in females; offer of 3 screens over 18 mos at home vs. in-clinic to high-risk population** | | | | | | | | |
| 1 | RCT | -0.5 | -0.5 | -1.0 | -1.0 | No concerns | In a small RCT (n=205) comparing home versus clinic screening (3 invites over 18 mos) for CT & NG in high-risk females recruited via community outreach, there was no difference between groups in the incidence of these STIs (11.3 vs 11.2 infections per 100 woman-years; p values or 95% CI not reported) over a follow-up of 18 months. | ⊕⊖⊖⊖  VERY LOW^a^ |

CI: confidence interval; CT: chlamydia trachomatis; NG: neisseria gonorrhea; RCT: randomized controlled trial; STIs: sexually transmitted infections

**Explanation:**

^a^ Some concerns about **ROB** (selection and performance biases) and **lack of consistency** from no additional studies; serious **indirectness** from use of same geographical region for both intervention groups (diminishing potential effects on transmission) and use of non-healthcare setting for recruitment; and serious **imprecision** due to inadequate sample size

**10B GRADE Summary of Findings Table**

| **Outcome**  **No. participants (studies)** | **Findings** | **Certainty of the evidence (GRADE)** | **What happens?** |
| --- | --- | --- | --- |
| Transmission: Incidence of CT & NG in high-risk females  Offer-to-screen design comparing home vs in-clinic screening, 3 invites over 18 mos  Follow-up: 18 months  205 (1 RCT) | In a small RCT (n=205) comparing home versus clinic screening (3 invites over 1 mos) for CT & NG in high-risk females recruited via community outreach, there was no difference between groups in the incidence of these STIs (11.3 vs 11.2 infections per 100 woman-years; p values or 95% CI not reported) over a follow-up of 18 months. | ⊕⊖⊖⊖  VERY LOW due to risk of bias, inconsistency, indirectness, and imprecision^a^ | The evidence on the effects on transmission of CT and NG from moderate-intensity screening using home versus clinic sampling is very uncertain. |

CI: confidence interval; CT: chlamydia trachomatis; NG: neisseria gonorrhea; RCT: randomized controlled trial; ROB: risk of bias; STIs: sexually transmitted infections

**Explanations**

^a^ Some concerns about **ROB** (selection and performance biases) and lack of consistency from no additional studies serious **indirectness** from use of same geographical region for both intervention groups (diminishing potential effects on transmission) and use of non-healthcare setting for recruitment; and serious **imprecision** due to inadequate sample size

**Evidence Set 11 Different screening approaches; Transmission of CT & NG: Treatment as surrogate (indirect) outcome (# treated/# randomized)**

Included studies: Wilson 2017, Regan 2012, Senok 2005 (all RCTs)

Threshold for important effect: >20 per 1000 more [benefit] or fewer [harm]

**11A GRADE Evidence Profile Table**

| **Certainty assessment** | | | | | | | **№ of patients assessed for outcome** | | **Effect (Home vs. clinic)** | | **Certainty** |
| --- | --- | --- | --- | --- | --- | --- | --- | --- | --- | --- | --- |
| **№ of studies** | **Study design** | **Risk of bias** | **Inconsistency** | **Indirectness** | **Imprecision** | **Other considerations** | **Home screening** | **Clinic screening** | **Relative (95% CI)** | **Absolute (95% CI)** |  |
| **Treatment of CT for both sexes;** **offer of 1 screen at home vs. in-clinic to general-risk population** | | | | | | | | | | | |
| 1 | RCT | No concerns | No concerns | -2.0 | -0.5 | No concerns | 1031 | 1032 | Peto OR 1.65 (0.41 to 6.63) | 1.9 more per 1000 (1.7 less to 16.3 more) | ⊕⊕⊖⊖ to ⊕⊖⊖⊖  LOW-TO-VERY LOW |
| **Treatment of CT for females; offer of 1 screen at home vs. in-clinic (opportunistically) to general-risk population** | | | | | | | | | | | |
| 1 | RCT | -0.5 | No concerns | -1.5 | -1.5 | No concerns | 124 | 136 | Peto OR 0.82 (0.18 to 3.68) | 5.2 fewer per 1000 (23.8 fewer to 77.7 more) | ⊕⊖⊖⊖  VERY LOW |
| **Treatment of CT for males; offer of 1 screen at home vs. clinic to general-risk population** | | | | | | | | | | | |
| 1 | RCT | No concerns | No concerns | -2.0 | -1.5 | No concerns | 100 | 100 | Peto OR 0.36 (0.05 to 2.61 | 19.2 fewer per 1000 (28.5 fewer to 48.3 more) | ⊕⊖⊖⊖  VERY LOW |
| **Treatment of NG for both sexes; offer of 1 screen at home vs. clinic to general-risk population** | | | | | | | | | | | |
| 1 | RCT | No concerns | No concerns | -2.0 | -0.5 | No concerns | 1031 | 1032 | Peto OR 1.00 (0.20 to 4.97) | 0 more per 1000 (2.32 fewer to 11.5 more) | ⊕⊕⊖⊖ to ⊕⊖⊖⊖  LOW-TO-VERY LOW |
| **Treatment of NG for males; offer of 1 screen at home vs. clinic to general-risk population** | | | | | | | | | | | |
| 1 | RCT | No concerns | No concerns | -1.5 | -1.5 | No concerns | 100 | 100 | Peto OR 7.54 (0.78 to 73.33) | 0 events in control (clinic) group | ⊕⊖⊖⊖  VERY LOW |

Abbreviations: CI=confidence interval; CT=chlamydia trachomatis; NG=neisseria gonorrhea; OR=odds ratio; RCT=randomized controlled trial

**Explanations:**

**Treatment of CT for both sexes; offer of 1 screen at home vs. in-clinic to general-risk population**: Only study in analysis but some consistency with other studies for this outcome; very serious **indirectness** from use of treatment outcome to serve as proxy for transmission and non-health care setting used for recruitment; some **imprecision** from small sample for rare event.

**Treatment of CT for females; offer of 1 screen at home vs. in-clinic (opportunistically) to general-risk population:** Some concerns with **ROB** from unclear selection, performance and detection biases; serious **indirectness** from use of treatment outcome to serve as proxy for transmission; very serious **imprecision** from wide 95% CI (including important benefit and harm) due to small sample.

**Treatment of CT for males; offer of 1 screen at home vs. clinic to general-risk population:** Very serious **indirectness** from use of treatment outcome to serve as proxy for transmission and non-health care setting used for recruitment; very serious **imprecision** from wide 95% CI (including important benefit and harm) and small sample.

**Treatment of NG for both sexes; offer of 1 screen at home vs. clinic to general-risk population**^:^  Only study in analysis but some consistency with other studies for this outcome; very serious i**ndirectness** from use of treatment outcome to serve as proxy for transmission and non-health care setting; some **imprecision** from small sample for rare event.

**Treatment of NG for males; offer of 1 screen at home vs. clinic to general-risk population**: Very serious **indirectnes**s from use of treatment outcome to serve as proxy for transmission and non-health care setting; very serious **imprecision** from small sample size for this rare outcome.

**11B GRADE Evidence Profile Table**

| **Outcome**  **No. participants (studies)** | **Relative effect (95% CI)** | **Anticipated absolute effects (95% CI)*** | | | **Certainty of the evidence (GRADE)** | **What happens?** |
| --- | --- | --- | --- | --- | --- | --- |
|  |  | **Without screening** | **With a single CT screen** | **Difference** |  |  |
| Transmission: Treatment of CT in both sexes, general-risk  Home vs in-clinic screening; 1 invitation  Follow-up: minimum 10 months  2,063 (1 RCT) | Peto OR 1.65 (0.41 to 6.63) | 2.9 per 1000 | 4.8 per 1000 (1.2 to 19.2) | 1.9 more per 1000 (1.7 less to 16.3 more) | ⊕⊕⊖⊖ to ⊕⊖⊖⊖  LOW-TO-VERY LOW | Offering one home versus clinic STI screening (all treatment via clinics) may make little or no difference in the transmission of CT. |
| Transmission: Treatment of CT in females; general-risk  Home vs in-clinic screening; 1 invitation  Follow-up: 4 months  260 (1 RCT) | Peto OR 0.82 (0.18 to 3.68) | 29 per 1000 | 23.8 per 1000 (5.2 to 106.7) | 5.2 fewer per 1000 (23.8 fewer to 77.7 more) | ⊕⊖⊖⊖  VERY LOW | The evidence on the effects of offering one home versus opportunistic clinic screen on transmission of CT in females is uncertain. |
| Transmission: Treatment of CT in males; general risk  Home vs in-clinic screening; 1 invitation  Follow-up: 3 months  200 (1 RCT) | Peto OR 0.36 (0.05 to 2.61) | 30 per 1000 | 10.8 per 1000 (1.5 to 78.3) | 19.2 fewer per 1000 (28.5 fewer to 48.3 more) | ⊕⊖⊖⊖  VERY LOW | The evidence on the effects of offering one home versus clinic screen on transmission of CT in males is uncertain. |
| Transmission: Treatment of NG in both sexes; general risk  Home vs in-clinic screening; 1 invitation  Follow-up: minimum 10 months  2,063 (1 RCT) | Peto OR 1.00 (0.20 to 4.97) | 2.9 per 1000 | 2.9 per 1000 (0.6 to 14.4) | 0 more per 1000 (2.3 fewer to 11.5 more) | ⊕⊕⊖⊖ to ⊕⊖⊖⊖  LOW-TO-VERY LOW | Offering one home versus clinic STI screening (all treatment via clinics) may make little or no difference in the transmission of NG (. |
| Transmission: Treatment of NG in males; general risk  Home vs in-clinic screening; 1 invitation  Follow-up: 3 months  200 (1 RCT) | Peto OR 7.54 (0.78 to 73.33) | 0 per 1000 | 0 events in control (clinic) group | 0 events in control (clinic) group | ⊕⊖⊖⊖  VERY LOW | The evidence on the effects of offering one home versus clinic screen on transmission of NG in males is uncertain. |

Abbreviations: CI=confidence interval; CT=chlamydia trachomatis; NG=neisseria gonorrhea; OR=odds ratio; RCT=randomized controlled trial

*****The effect with a single CT screen (and its 95% confidence interval) is based on the effect without screening and the relative effect of the intervention (and its 95% CI).

**Explanations:**

**Treatment of CT for both sexes; offer of 1 screen at home vs. in-clinic to general-risk population**: Only study in analysis but some consistency with other studies for this outcome; very serious **indirectness** from use of treatment outcome to serve as proxy for transmission and non-health care setting used for recruitment; some **imprecision** from small sample for rare event.

**Treatment of CT for females; offer of 1 screen at home vs. in-clinic (opportunistically) to general-risk population:** Some concerns with **ROB** from unclear selection, performance and detection biases; serious **indirectness** from use of treatment outcome to serve as proxy for transmission; very serious **imprecision** from wide 95% CI (including important benefit and harm) due to small sample.

**Treatment of CT for males; offer of 1 screen at home vs. clinic to general-risk population:** Very serious **indirectness** from use of treatment outcome to serve as proxy for transmission and non-health care setting used for recruitment; very serious **imprecision** from wide 95% CI (including important benefit and harm) and small sample.

**Treatment of NG for both sexes; offer of 1 screen at home vs. clinic to general-risk population**^:^  Only study in analysis but some consistency with other studies for this outcome; very serious i**ndirectness** from use of treatment outcome to serve as proxy for transmission and non-health care setting; some **imprecision** from small sample for rare event.

**Treatment of NG for males; offer of 1 screen at home vs. clinic to general-risk population**: Very serious **indirectnes**s from use of treatment outcome to serve as proxy for transmission and non-health care setting; very serious **imprecision** from small sample size for this rare outcome.

**11C Forest Plots**

**Treatment of CT for both sexes; offer of 1 screen at home vs in clinic to general-risk population**

**
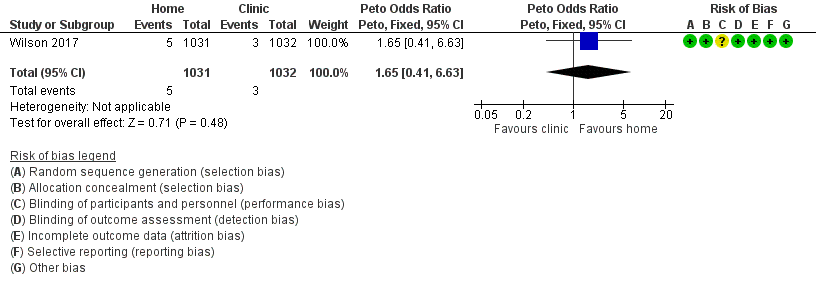
**

**Treatment of CT for females; offer of 1 screen at home vs. in clinic to general-risk population**

**
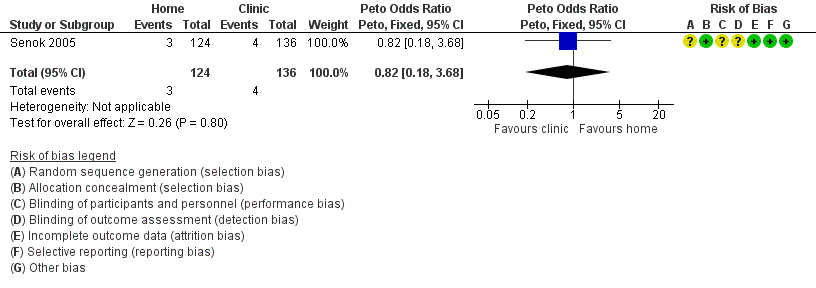
**

**Treatment of CT for males; offer of 1 screen at home vs. in clinic to general-risk population**

**
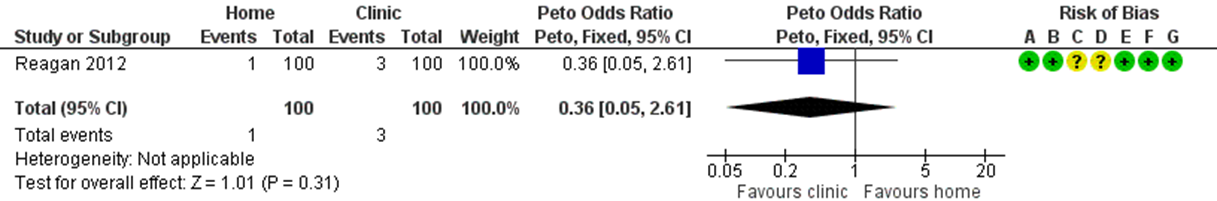
**

**Treatment of NG for both sexes; offer of 1 screen at home vs. in clinic to general-risk population**

**
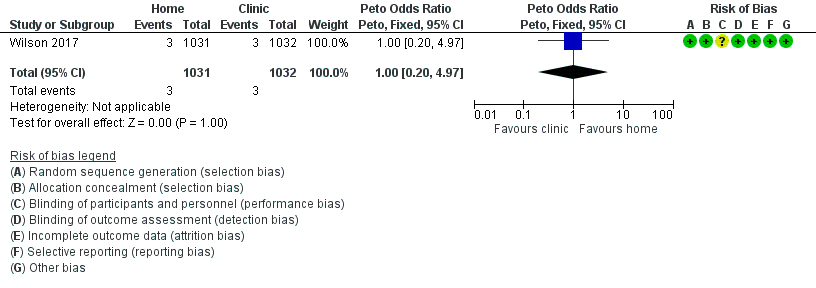
**

**Treatment of NG for males; offer of 1 screen at home vs in clinic in general-risk population**


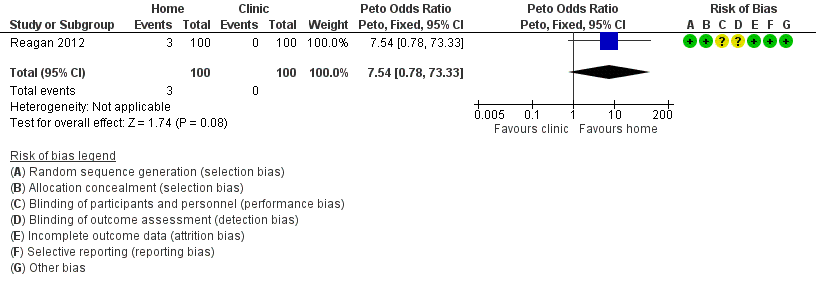


**Evidence Set 12 Patient preferences (relative importance for outcomes): Health state utility values (HSUV)**

Included studies: Kupperman 2007, Smith 2008, Institute of Medicine 1999, Trent 2011 (economic evaluations included and reported on by Jackson 2014 used for durations for QALY calculations)

**12A GRADE Evidence Profile Table**

| **Outcome** | **Certainty assessment** | | | | | | | **Findings** | **Certainty** |
| --- | --- | --- | --- | --- | --- | --- | --- | --- | --- |
|  | **№ of studies** | **Study design** | **Risk of bias** | **Inconsistency** | **Indirectness** | **Imprecision** | **Other considerations** |  |  |
| Infertility HSUV | 3 | Preference- based | Some concerns^b^ | Some concerns^c^ | No serious concerns | No serious concerns | None | Results by method: TTO range: 0.76-0.91 (SD 0.25-0.34); VAS range 0.53-0.68 (SD 0.24-0.29); Indirect 0.82 (variance NR)  Best estimate of utility value^a^ = **0.80 (range 0.76-0.91)** | ⊕⊕⊕⊖  MODERATE |
| Chronic pelvic pain HSUV | 4 | Preference-based | Some concerns^b^ | Some concerns^c^ | No serious concerns | No serious concerns | None | Results by method: TTO Range: 0.69-0.85 (SD 0.29-0.38); VAS Range: 0.45-0.61 (SD 0.29-0.38); Indirect 0.60  Best estimate = **0.76 (range 0.69-0.85)** | ⊕⊕⊕⊖  MODERATE |
| Ectopic pregnancy HSUV | 3 | Preference-based | Serious concerns^b^ | Some concerns^c^ | No serious concerns | No serious concerns | None | Results by method: TTO Range: 0.79-0.91 (SD 0.26-0.34); VAS Range: 0.55-0.73 (SD 0.21-0.25); Indirect: Out-patient 0.58 vs in-patient 0.23 with recuperation 0.60 (added to PID health state)  Best estimate = **0.83 (range 0.79-0.91)** | ⊕⊕⊕⊖-⊕⊕⊖⊖  LOW-TO-MODERATE |
| PID HSUV | 3 | Preference-based | Serious concerns^b^ | Some concerns^c^ | No serious concerns | No serious concerns | None | Results by method:  PID Out-patient: TTO Range: 0.82-0.90 (SD 0.22-0.33); VAS Range: 0.62-0.76 (SD 0.17-0.24); Indirect 0.63  PID In-patient: TTO Range: 0.82-0.88 (SD 0.27-0.36); VAS Range: 0.60-0.74 (SD 0.20-0.25); Indirect IPNS 0.57 vs IPS 0.46 with OPAIP 0.83  Best estimate (majority treated as outpatient) = **0.86 (range 0.82-0.90)** | ⊕⊕⊕⊖-⊕⊕⊖⊖  LOW-TO-MODERATE |
| Cervicitis HSUV | 1 | Preference-based | Some concerns^b^ | No serious concerns | Some concerns^d^ | Some concerns^e^ | None | Indirect method (best estimate): 0.90 (no measure of variance) | ⊕⊕⊕⊖-⊕⊕⊖⊖  LOW-TO-MODERATE |
| Rank-order of outcomes | 4 | Preference-based & economic evaluations from Jackson et al | Some concerns^f^ | Some concerns^f^ | Some concerns^f^ | No serious concerns | None | **Infertility > chronic pelvic pain >> ectopic pregnancy = PID = cervicitis**  Rank order based on range of QALY losses using best estimates ((1- best estimate of utility) x duration in years) for each health state:  Infertility (0.20 x 10-30 years) = 2–6 QALY loss)  **>** chronic pelvic pain (0.24 x 5-10 years = 1.2-2.4 QALY loss)  **>>** ectopic pregnancy (0.17 for 4 weeks =0.013 QALY loss)  **=** cervicitis (0.10 x 4 weeks = 0.008 QALY loss)  **=** PID (0.14 x 10-12 days = 0.004 QALY loss)  Sensitivity analysis using values from indirect utility estimates instead of TTO: Infertility (1.8-5.4 QALY loss) > CPP (2-4 QALY loss) >> Ectopic pregnancy (0.03-0.048 QALY loss) = PID (0.019 QALY loss) = cervicitis (QALY loss 0.008)  Assumptions: 1) utility will be the same for entire duration of health state; 2) those without health state have perfect health (i.e. actual QALY loss may be higher); 3) TTO method is not affected by the duration applied to the scenario (constant proportional trade-off holds) and the value of each health state is not affected by the states that come before or after it (additive separability holds)(Tsuchiya 2004). | ⊕⊕⊕⊖-⊕⊕⊖⊖  LOW-TO-MODERATE |

Abbreviations: HSUV=health state utility value; IPNS=inpatient nonsurgical; IPS=inpatient surgical; OPAIP=outpatient after inpatient; SD=standard deviations; TTO=time-trade off; VAS=visual analogue scale

**Explanations:**

^a^ Best estimates are based on values at mid-range within values provided by TOO methods. Variations between studies in the populations and methods (e.g., scenarios) did not help determine one best estimate within the range: 1) Kupperman et al. in which patients with chronic pelvic pain were not explicitly asked to account for possibility of lessening symptoms (although many had experienced resolution of their symptoms) reported higher utility scores than did Smith & Trent which described that the pain may go away; 2) the time horizon used for the TTO was 10 yrs for Smith and 50 yr for Trent, but Smith reported lower utility values for infertility against expectations, 3) the values between patients who had and had not experienced PID were not different for the TTO values in Smith. VAS scores are lower than those obtained by other measures because the VAS does not involve valuation against an external metric, such as time or risk of death; as such, the VAS is less well accepted for decision analysis and cost-effectiveness analysis. TTO scores have a stronger theoretical basis and are generally accepted as “true” utilities. The values provided by the VAS methods and the indirect methods (using HUI-2 scoring) used in the IOM study are not relied on for the best estimates (except for cervicitis where no TTO was used) but are considered to provide some indication of inconsistency. For the temporary health state of PID, there was also concerns about the use of TTO which may lead to less valid results (e.g. underestimate of utility) because an unrealistic scenario is presented to patients (i.e., health state followed immediately by death); furthermore, the terminal status of the temporary health state, caused by subsequent death, may influence the evaluation of the health state (Locadia 2004-2).

^b^ Some concerns about **ROB** from selection of participants (expert committee members) in IOM study and unknown or possible incomplete data in all studies, but uncertain if impact on findings used for best estimate (from TTO); serious concerns for ectopic pregnancy from additional concerns about the outcome presentations/scenarios underestimating the health and psychosocial impacts of this condition. Concerns were also serious for the temporary heath state of PID, from use of TTO, which may lead to less valid results (e.g., underestimate of utility) because an unrealistic scenario is presented to patients; furthermore, the terminal status of the temporary health state, caused by subsequent death, may influence the evaluation of the health state (Locadia 2004-2).

^c^Some concerns about **inconsistency** in utility values that was unexplained by participant characteristics (age, experience of outcome) or TTO methods (see a above).

^d^Some concerns about **indirectness** for cervicitis because of use of indirect methods (based on population tariffs) for valuing this outcome.

^e^Some concerns about sample size of IOM study (not reported).

^f^Some concerns about **ROB** for suboptimal outcome descriptions for ectopic pregnancy that may place this outcome worse in terms of QALY loss compared with PID and cervicitis; some concerns about **inconsistency** for order of infertility relative to chronic pelvic pain (i.e., if both experienced for the same time frame QALY loss would be similar; use of indirect utility estimates suggests these outcomes may have similar importance in some cases); some concerns about **indirectness** when using a range of durations created by expert opinion although also accounted for in inconsistency. Sensitivity analyses using different TTO utilities (across the range of the best estimate) indicated no influence on rank order.

**12B GRADE Summary of Findings Table**

| **Outcome**  **No. participants (studies)** | **Findings** | **Certainty of the evidence (GRADE)** | **What does the evidence say?** |
| --- | --- | --- | --- |
| Infertility HSUV  461 plus IOM committee members (3 studies) | Results by method: TTO range: 0.76-0.91 (SD 0.25-0.34); VAS range 0.53-0.68 (SD 0.24-0.29); Indirect 0.82  Best estimate of utility value^a^ = **0.80 (range 0.76-0.91)** | ⊕⊕⊕⊖  MODERATE^b,c^ | Based on utility values, the outcomes are probably of similar importance to people. |
| Chronic pelvic pain HSUV  733 plus IOM committee members (4 studies) | Results by method: TTO Range: 0.69-0.85 (SD 0.29-0.38); VAS Range: 0.45-0.61 (SD 0.29-0.38); Indirect 0.60  Best estimate = **0.76 (range 0.69-0.85)** | ⊕⊕⊕⊖  MODERATE^b,c^ |  |
| Ectopic pregnancy HSUV  461 plus IOM committee members (3 studies) | Results by method: TTO Range: 0.79-0.91 (SD 0.26-034); VAS Range: 0.55-0.73 (SD 0.21-0.25); Indirect : Out-patient 0.58 vs in-patient 0.23 with recuperation 0.60 (added to PID health state)  Best estimate = **0.83 (range 0.79-0.91)** | ⊕⊕⊕⊖-⊕⊕⊖⊖  LOW-TO-MODERATE^b,c^ |  |
| PID HSUV  461 plus IOM committee members (3 studies) | Results by method:  PID Out-patient: TTO Range: 0.82-0.90 (SD 0.22-0.33); VAS Range: 0.62-0.76 (SD 0.17-0.24); Indirect 0.63  PID In-patient: TTO Range: 0.82-0.88 (SD 0.27-0.36); VAS Range: 0.60-0.74 (SD 0.20-0.25); Indirect IPNS 0.57 vs IPS 0.46 with OPAIP 0.83  Best estimate (majority treated as outpatient) = **0.86 (range 0.82-0.90)** | ⊕⊕⊕⊖-⊕⊕⊖⊖  LOW-TO-MODERATE^b,c^ |  |
| Cervicitis HSUV  NR (IOM committee members( (1 study) | Indirect methods: 0.90 (no measure of variance) | ⊕⊕⊕⊖-⊕⊕⊖⊖  LOW-TO-MODERATE^b,d,e^ |  |
| Rank order of outcomes | **Infertility > chronic pelvic pain >> ectopic pregnancy = PID = cervicitis**  Rank order based on range of QALY losses using best estimates ((1- best estimate of utility) x duration in years) for each health state:  Infertility (0.20 x 10-30 years) = 2–6 QALY loss)  **>** chronic pelvic pain (0.24 x 5-10 years = 1.2-2.4 QALY loss)  **>>** ectopic pregnancy (0.17 for 4 weeks =0.013 QALY loss)  **=** cervicitis (0.10 x 4 weeks = 0.008 QALY loss)  **=** PID (0.14 x 10-12 days = 0.004 QALY loss)  Sensitivity analysis using values from indirect utility estimates instead of TTO: Infertility (1.8-5.4 QALY loss) > CPP (2-4 QALY loss) >> Ectopic pregnancy (0.03-0.048 QALY loss) = PID (0.019 QALY loss) = cervicitis (QALY loss 0.008)  Assumptions: 1) utility will be the same for entire duration of health state; 2) those without health state have perfect health (i.e. actual QALY loss may be higher); 3) TTO method is not affected by the duration applied to the scenario (constant proportional trade-off holds) and the value of each health state is not affected by the states that come before or after it (additive separability holds)(Tsuchiya 2004). | ⊕⊕⊕⊖-⊕⊕⊖⊖  LOW-TO-MODERATE^f^ | The rank-order of importance for the benefit outcomes, when considering the duration of effects, may be infertility > chronic pelvic pain >> ectopic pregnancy = cervicitis = PID, although there are several assumptions that are being made. |

Abbreviations: HSUV=health state utility value; IPNS=inpatient nonsurgical; IPS=inpatient surgical; OPAIP=outpatient after inpatient; ROB=risk of bias; SD=standard deviations; TTO=time-trade off; VAS= visual analogue scale

**Explanations:**

^a^ Best estimates are based on values at mid-range within values provided by TOO methods. Variations between studies in the populations and methods (e.g., scenarios) did not help determine one best estimate within the range: 1) Kupperman et al. in which patients with chronic pelvic pain were not explicitly asked to account for possibility of lessening symptoms (although many had experienced resolution of their symptoms) reported higher utility scores than did Smith & Trent which described that the pain may go away; 2) the time horizon used for the TTO was 10 yrs for Smith and 50 yr for Trent, but Smith reported lower utility values for infertility against expectations, 3) the values between patients who had and had not experienced PID were not different for the TTO values in Smith. VAS scores are lower than those obtained by other measures because the VAS does not involve valuation against an external metric, such as time or risk of death; as such, the VAS is less well accepted for decision analysis and cost-effectiveness analysis. TTO scores have a stronger theoretical basis and are generally accepted as “true” utilities. The values provided by the VAS methods and the indirect methods (using HUI-2 scoring) used in the IOM study are not relied on for the best estimates (except for cervicitis where no TTO was used) but are considered to provide some indication of inconsistency. For the temporary health state of PID, there was also concerns about the use of TTO which may lead to less valid results (e.g. underestimate of utility) because an unrealistic scenario is presented to patients (i.e., health state followed immediately by death); furthermore, the terminal status of the temporary health state, caused by subsequent death, may influence the evaluation of the health state (Locadia 2004-2).

^b^ Some concerns about **ROB** from selection of participants in IOM study and unknown or possible incomplete data in all studies, but uncertain if impact on findings used for best estimate (from TTO); serious concerns for ectopic pregnancy from additional concerns about the outcome presentations underestimating the health and psychosocial impacts of this condition

^c^Some concerns about **inconsistency** in utility values that was unexplained by participant characteristics (age, experience of outcome) or TTO methods (see a above).

^d^Serious concerns about **indirectness** for cervicitis because of use of indirect methods for valuing this outcome.

^e^Some concerns about sample size of IOM study (not reported).

^f^Some concerns about **ROB** for suboptimal outcome descriptions for ectopic pregnancy that may place this outcome worse in terms of QALY loss compared with PID and cervicitis; some concerns about **inconsistency** for order of infertility relative to chronic pelvic pain (i.e., if both experienced for the same time frame QALY loss would be similar); some concerns about **indirectness** when using a range of durations created by expert opinion although also accounted for in inconsistency. Sensitivity analyses using different utilities (across the range of the best estimate) indicated no influence on rank order.

**Evidence Set 13 Patient preferences (relative importance on benefits versus harms): non-utility studies**

Included studies: Balfe 2010, Barth 2002, Booth 2012, Booth 2015, Chako 2008, Reed 2017, Theunissen 2015 (patients considering screening); Duncan 2001, Mills 2006, Nielsen 2017 (patients who have undergone screening)

**13A GRADE Evidence Profile Table**

| **Outcome** | **Certainty assessment** | | | | | | | **Findings** | **Certainty** |
| --- | --- | --- | --- | --- | --- | --- | --- | --- | --- |
|  | **№ of studies** | **Study design** | **Risk of bias** | **Inconsistency** | **Indirectness** | **Imprecision** | **Other considerations** |  |  |
| **^Patients mainly considering rather than undergoing CT & NG screening^** | | | | | | | | | |
| Relative importance of benefits vs harms | 7 | Qualitative (6); cross –sectional (1) | Serious concerns^a^ | Serious concerns^a^ | Serious concerns^a^ | No serious concerns | None | Two studies of general-risk populations found that harms from stigma of a diagnosis and (less so) anxiety from testing may outweigh the potential benefits on their reproductive health (unspecified outcomes) and transmission (Balfe, Barth). One study’s findings indicated that a fine balance may exist between a large potential for reduced transmission and several harms, from stigma from testing, anxiety about CT, and relationship distress (Booth 2012). The remaining four studies suggested that the potential benefits from reduced transmission and (less so) improved future reproductive health will outweigh the harms from anxiety and stigma when making decision about screening (Booth 2015, Chako, Reed, Theunissin). The relative importance placed on benefits may be higher for women. Ages in the studies were 14-29 years, with most 18-21.  **Summary**: Patients considering screening (especially females) may place more importance on the potential benefits for future reproductive health and transmission than the harms from stigma and anxiety. | ⊕⊖⊖⊖  VERY LOW |
| **^Patients who have undergone CT screening^** | | | | | | | | | |
| Relative importance of benefits vs harms | 3 | Qualitative | Serious concerns^b^ | No serious concerns | Serious concerns^b^ | Serious concerns^b^ | None | The potential benefits for reducing infertility and/or transmission may outweigh any (transient and mild) harms from anxiety or stigma experienced from screening, except in those getting a diagnosis where the stigma (e.g., about transmitting to others in social network) and anxiety about infertility will likely become relatively more important (Duncan, Mills, Nielsen). It is unclear if the harms from a diagnosis would deter people in these studies from future screening. Because of being told about the uncertain course of CT infections and duration required to cause infertility (Duncan, Mills), many women who tested positive in two studies were significantly concerned about the possibility of being infertile, and distressed by their unanswered questions. One of the studies found that the harm from stigma after a diagnosis (or an anticipated one) was the main driver for regular repeat testing, to alleviate the feelings (Nielsen). Ages of study participants were 16-39.  **Summary**: Patients who have undergone screening, and are not diagnosed with CT, may place more importance infertility and transmission than on harms from anxiety and stigma. | ⊕⊖⊖⊖  VERY LOW |

CT: chlamydia trachomatis; NG: neisseria gonorrhea

**Explanations:**

^a^ Very low certainty due to serious concerns about **ROB** with respect to our research interest (uninformed decisions with few participants having awareness of specific health outcomes and none about their risk magnitudes, and little direct relation of outcomes to screening decisions), **inconsistency** between studies in direction of relative importance, **and indirectness** (most harms were anticipated rather than experienced, studies did not consider specific benefit outcomes and in only one did patients mention infertility).

^b^ Very low certainty because of **ROB** (uninformed findings with few participants having awareness of specific health outcomes and none about their risk magnitudes, and use of selective samples), **indirectness** (largely related to those having a diagnosis or repeatedly screening), and **imprecision** (three small studies when wanting to quantify findings as much as possible).

**13B. GRADE Evidence Profile Table**

| **Outcome**  **No. participants (studies)** | **Findings** | **Certainty of the evidence (GRADE)** | **What happens?** |
| --- | --- | --- | --- |
| Relative importance of benefits vs harms  Patients mainly considering rather than undergoing CT & NG screening  777 (7 studies) | Two studies of general-risk populations found that harms from stigma of a diagnosis and (less so) anxiety from testing may outweigh the potential benefits on their reproductive health (unspecified outcomes) and transmission (Balfe, Barth). One study’s findings indicated that a fine balance may exist between a large potential for reduced transmission and several harms, from stigma from testing, anxiety about CT, and relationship distress (Booth 2012). The remaining four studies suggested that the potential benefits from reduced transmission and (less so) improved future reproductive health will outweigh the harms from anxiety and stigma when making decision about screening (Booth 2015, Chako, Reed, Theunissin). The relative importance placed on benefits may be higher for women. | ⊕⊖⊖⊖  VERY LOW due to ROB, inconsistency, and indirectness^a^ | Patients considering screening (especially females) may place more importance on the potential benefits than on the harms from screening, but the evidence is uncertain.  Transmission as the only benefit considered may still lead to the same assessment as would consideration of both transmission and future reproductive health. |
| Relative importance of benefits vs harms  Patients who have undergone CT screening  77 (3 studies) | The potential benefits for reducing infertility and/or transmission may outweigh any (transient and mild) harms from anxiety or stigma experienced from screening, except in those getting a diagnosis where the stigma (e.g., about transmitting to others in social network) and anxiety about infertility will likely become relatively more important (Duncan, Mills, Nielsen). It is unclear if the harms from a diagnosis would deter people in these studies from future screening. Because of being told about the uncertain course of CT infections and duration required to cause infertility (Duncan, Mills), many women who tested positive in two studies were significantly concerned about the possibility of being infertile, and distressed by their unanswered questions. One of the studies found that the harm from stigma after a diagnosis (or an anticipated one) was the main driver for regular repeat testing, to alleviate the feelings (Nielsen). | ⊕⊖⊖⊖  VERY LOW due to ROB, indirectness, and imprecision^b^ | Patients who have undergone screening, and are not diagnosed with CT, may place more importance on the benefits than on the harms, but the evidence is uncertain. |

Abbreviations: CT= chlamydia trachomatis; NG=neisseria gonorrhea; ROB=risk of bias

**Explanations:**

^a^Very low certainty due to serious concerns about **ROB** with respect to our research interest (uninformed decisions with few participants having awareness of specific health outcomes and none about their risk magnitudes, and little direct relation of outcomes to screening decisions), **inconsistency** between studies in direction of relative importance, **and indirectness** (most harms were anticipated rather than experienced, studies did not consider specific benefit outcomes and in only one did patients mention infertility).

^b^ Very low certainty because of **ROB** (uninformed findings with few participants having awareness of specific health outcomes and none about their risk magnitudes, and use of selective samples), **indirectness** (largely related to those having a diagnosis or repeatedly screening), and **imprecision** (three small studies when wanting to quantify findings as much as possible).
